# Supplementary material for: Red-light-mediated copper-catalyzed photoredox catalysis promotes regioselectivity switch in the difunctionalization of alkenes
Source: Nat Commun. 2024 Jun 18;15:5208. doi: 10.1038/s41467-024-49514-4 (PMC11189478; doi:10.1038/s41467-024-49514-4)
Supplement: Supplementary file 1 — Supplementary Information [file 41467_2024_49514_MOESM1_ESM.pdf]

Supplementary Information

Red Light-Mediated Copper-Catalyzed Photoredox Catalysis Promotes Regioselectivity Switch in the Difunctionalization of Alkenes

Tong Zhang[1], Jabor Rabeah[2,3], and Shoubhik Das*[1,4]

1. Department of Chemistry, University of Antwerp, 2020 Antwerp, Belgium.
2. Leibniz-Institut für Katalyse e.V. an der Universität Rostock (LIKAT), 18059 Rostock, Germany.
3. State Key Laboratory of Low Carbon Catalysis and Carbon Dioxide Utilization, Lanzhou Institute of Chemical Physics (LICP), Chinese Academy of Sciences, Lanzhou 730000, P. R. China.
4. Department of Chemistry, University of Bayreuth, 95447 Bayreuth, Germany.

Contents

Supplementary Information **1**

Supplementary Notes **3**

1.1 Chemicals and solvents 3

1.2 Analysis 3

1.2.1 Nuclear magnetic resonance 3

1.2.2 Chromatography 3

1.2.3 Electron paramagnetic resonance spectroscopy (EPR) 4

Supplementary Methods and Discussions **4**

1.3 General procedure and optimizations 4

1.4 Mechanistic investigation 10

1.4.1 Electrochemical measurements 10

1.4.2 Quenching experiment and radical probe experiment 12

1.4.3 Analysis of Cu-CF_3 complex 14

1.4.4 Quantum yield (QY) measurement 21

1.4.5 Examples of unsuccessful substrates 21

1.5 Experiments 22

1.5.1 Photocatalytic reaction setup 22

1.5.2 Procedure for synthesis of TT-$CF_3^+OTF^-$ 22

1.5.3 Procedure for synthesis of Os(bptpy)$_2$(PF_6)$_2$ 23

1.5.4 Procedure for the synthesis of olefins from phenols 23

1.5.5 Procedure for synthesis of olefins from acids 24

1.5.6 Procedure for sulfonyltrifluoromethylation of olefins 25

1.6 Characterization 26

1.7 Spectra 43

Supplementary References **109**

1.8 Reference 109

Supplementary Notes

1.1 Chemicals and solvents

All reagents and solvents were purchased from certified chemical vendors and used without prior purification. Demineralized water was obtained through deionization of tap water using a EUROTEC L4 reverse osmosis installation. This instrument filters out salts by means of a semi-permeable membrane. Water that was used never exceeded a conductivity of 0.5 µS/cm. Gases were purchased in high-pressured cylinders (200 bar) and converted to the desired pressure by means of a pressure regulator.

1.2 Analysis

1.2.1 Nuclear magnetic resonance

^{1}H NMR (400 MHz) and ^{13}C NMR (101 MHz) spectra were recorded by using a Bruker Avance III Fourier Transform NMR spectrometer at 300 K unless explicitly stated otherwise, using deuterated solvents as internal standard (^{1}H: δ 7.26 ppm and ^{13}C {^{1}H}: δ 77.2 ppm in $CDCl_3$; ^{1}H: δ 2.50 ppm and 13C {^{1}H}: δ 39.52 in DMSO-d6). Chemical shifts (δ) were expressed in ppm and coupling constants (*J*) in Hertz (Hz). Splitting patterns are reported as: s (singlet), d (doublet), t (triplet), q (quadruplet), p (pentuplet), m (multiplet) or combinations thereof. Integration of the signals is presented as the number of hydrogen atoms.

1.2.2 Chromatography

Thin-layer chromatography was performed by using a heptane/ethyl acetate (EtOAc) solvent system as mobile phase and a 0.20 mm silica gel on an aluminium plate (Machery-Nagel Precoated TLC sheets Alugram® SIL G/UV254) as stationary phase. Visualization of the ‘spots’ was enhanced by illumination with UV-light (254 nm).
Flash chromatography was performed by using an automatic chromatographic system (Biotage® or Combiflash®) with on-line UV-detection (254 nm and 280 nm unless explicitly stated otherwise). A heptane/EtOAc solvent system was used as mobile phase and commercial silica cartridges (12-80 g, Grace®) as stationary phase.

1.2.3 Electron paramagnetic resonance spectroscopy (EPR)

EPR spectra of the reaction mixture were recorded at room temperature on a Bruker cw spectrometer ELEXSYS 500-10/12 (X-band, $\nu \approx$ 9.88 GHz) with a microwave power of 6.6 mW, a modulation frequency of 100 kHz and modulation amplitude of 5 G.

Supplementary Methods and Discussions

1.3 General procedure and optimizations

General procedure for optimizations:

A dried 8 mL reaction vial with magnetic stirring bar was charged with photocatalyst, CF_3-source, co-catalyst, sodium sulfinate salts (RSO_2Na) and 4-vinylbiphenyl (0.05 mmol, 1 equiv.) after which the vessel was evacuated using Schlenk techniques and flushed with N_2 three times. Dry DCM was added by using a syringe flushed with inert gas. The resulting mixture was stirred for 3-4 h under irradiation of blue LED (40 W, 456 nm) or red light (EvoluChem™ LED 650PF, 650 nm, 20 mW/cm^2) with the EvoluChem PhotoRedOx Box at room temperature. After 3-4 h, trifluorotoluene (prepared in stock solution) was added as an internal standard. After shaking the vial, the mixture was filtered by celite and was submitted to the NMR to determine the yield through ^{19}F NMR.

1.3.1 Optimizations based on red light system:

Supplementary Table 1. Investigation of the amount of sulfinate salts and CF_3 reagent and addition of ligands.

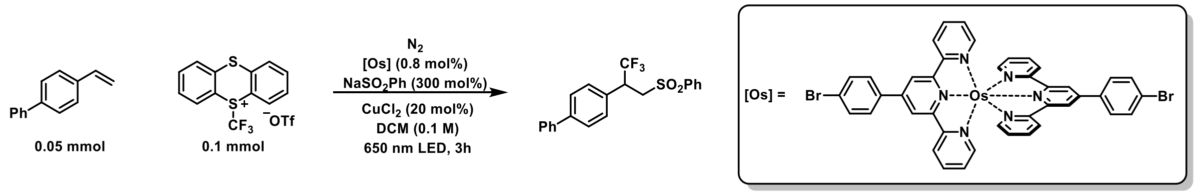

Entry	Variations	Yield (%)[a]
**1**	none	76(73[b])
**2**	2 equiv. $NaSO_2Ph$	50
**3**	1.5 equiv. CF_3-reagent	66
**4**	DCM (0.05 M)	43
**5**	30 mol% bpy ligand	0
**6**	30 mol% 1,10-phen ligand	0

[a]Yield was determined by ^{19}F NMR with trifluorotoluene as internal standard. [b]Isolated yields.

The amount of each of the component was evaluated: Lower concentration or equivalent of -CF_3 reagent and sulfinate salts provided comparably lower yield of product, which can be attributed to the kinetic effect. Since the presence of Cu-salts, commonly used ligands such as 2,2′-bipyridine (bpy) and 1,10-phenanthroline (1,10-phen) were added in the reactions, however, both of the ligands did not provide any product.

Supplementary Table 2. Investigations of -CF_3 reagents by the irradiation of red light.

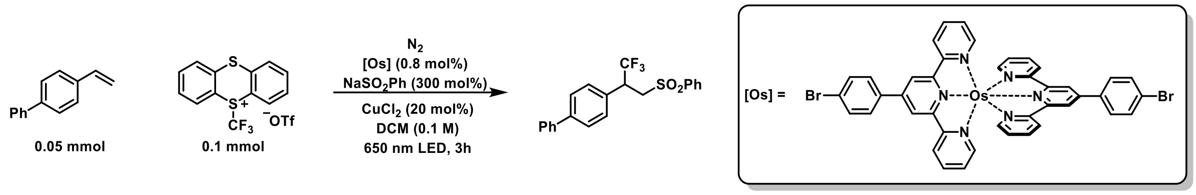

Entry	Variations	Yield (%)[a]
**1**	2.0 equiv. Togni I	trace
**2**	2.0 equiv. Togni II	8
**3**	2.0 equiv. Umemoto reagent	20
**4**	2.0 equiv. Mes-Umemoto reagent	trace
**5**	2.0 equiv. bpyCu(CF_3)$_3$	0

[a]Yield was determined by ^{19}F NMR with trifluorotoluene as internal standard.

When alternative electrophilic -CF_3 sources such as Togni reagents, Umemoto reagents, and Cu(CF_3)$_3$bpy were exploired, we observed substantially lower or negligible yields of the desired product. For Togni's reagents, Cu-salts directly reacted with them to form the Cu−CF_3 complexes, which caused lower selectivity and reactivity in the reaction. When Umemoto reagent was used as the -CF_3 source, the Os-catalyst at the excited state directly reduced it to the corresponding -CF_3 radical, which generated the free -CF_3 radical and caused the formation of side products, β-trifluoromethylated product. Compared to Umemoto reagent, Mes-Umemoto reagent was more difficult to be reduced by the Os-catalyst. Therefore, only trace quantity of product was detected. At last, Cu(CF_3)$_3$bpy was also examined and no product was obtained. From the ^{19}F NMR spectra, Cu(CF_3)$_3$bpy was intact under this reaction conditions.

Supplementary Table 3. Investigation of Cu-salts and solvents in the difunctionalization reaction.

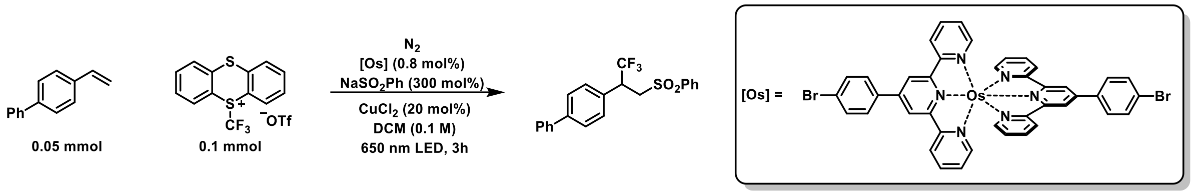

Entry	Variations	Yield (%)[a]
**1**	20 mol% CuCl	51
**2**	20 mol% Cu powder	0
**3**	MeCN (0.1 M)	26
**4**	EtOAc (0.1 M)	30
**5**	6 h	61
**6**	No $CuCl_2$	0
**7**	No [Os]	0
**8**	No light	0
**9**	40 W NIR Kessil lamp (740 nm)	24
**10**	w/o EvoluChem PhotoRedOx Box	52

[a]Yield was determined by ^{19}F NMR with trifluorotoluene as internal standard. [b]Isolated yields.

Investigations were conducted to find out whether variations in the valence state of Cu, specifically by involving Cu^{I} salt (such as CuCl) and Cu^{0} in the form of fresh copper powder, had an influence in the reaction. The results indicated that the presence of CuCl promoted the reaction to obtain the product, albeit resulting in a slightly reduced yield of approximately 20%. We assumed that the concentration of chloride was also crucial for achieving high photocatalytic activity (more details is found in **Supplementary Figure 8**). However, Cu-powder was not efficient under these conditions, giving no product. Additionally, two frequently used solvents in photocatalytic functionalization of -CF_3 group, namely MeCN and EtOAc, were also evaluated, however, no improvement was found. At last, to further increase the yield of product, we prolonged the reaction time to 6 hours, however, it revealed that extending the reaction time resulted in a marginal reduction in yield. To assess the necessity of the photocatalyst, Cu-salts, and light, a series of control experiments were meticulously conducted, elucidating their necessity in propelling the reaction.

1.3.2 Optimizations based on blue light system:

Investigation of the photocatalysts

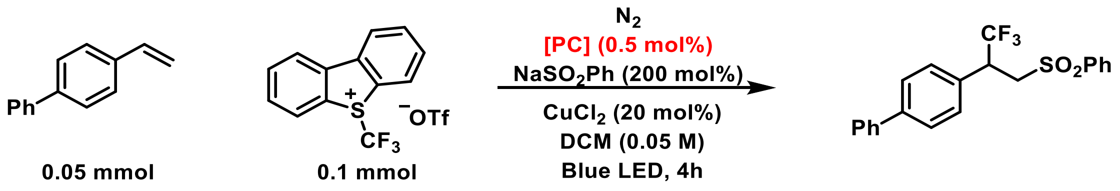

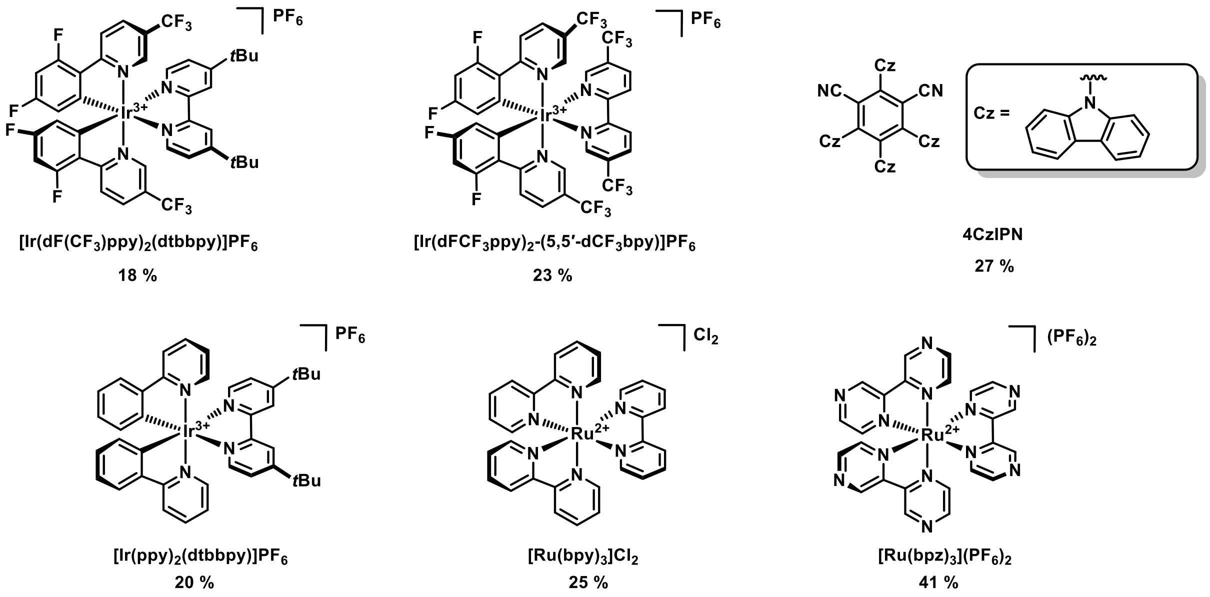

To firstly realize the reaction under blue light, 4-vinyl-1,1'-biphenyl, Umemoto's reagent and sodium benzenesulfinate ($NaSO_2Ph$) were initially chosen as the model substrate, electrophilic CF_3 source and sulfinate, respectively. Several commercially available Ru- and Ir-based photocatalysts and organic dye, 4-CzIPN, were investigated and all of them offered the desired product in 18-41% yield.

Investigation of CF_3-sources

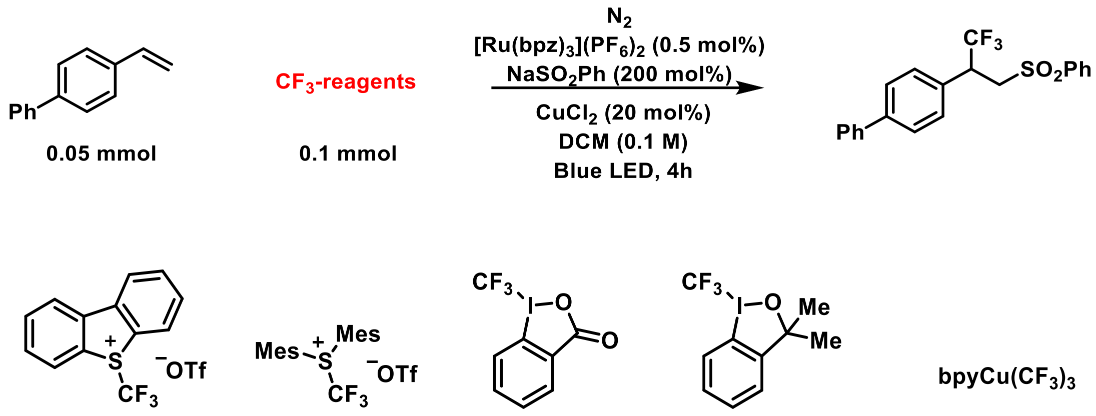

41 % | trace | 8 % | n.ob. | n.ob.

Since the $[Ru(bpz)_3](PF_6)_2$ has provided the best result, we continuously used it as the photocatalyst and further examined the CF_3 source. Various electrophilic CF_3 sources such as Mes-Umemoto's reagent, Togni reagent I/II and $Cu(CF_3)_3$bpy were utilized under the conditions, however, none of them offered the better yield. The Mes-Umemoto's reagent has more negative reduction potential compared to that of Umemoto's reagent, therefore, the reduction process of CF_3 source was probably not efficient by $[Ru(bpz)_3](PF_6)_2$. It is worth noting that both Togni's reagents are able to react with Cu-salts (here was $CuCl_2$) to form the Cu−CF_3 complexes, which can directly proceed the addition reaction with olefins. Therefore, main side-products were β-CF_3-difunctionalized products. The use of complex bpy$Cu(CF_3)_3$ did not show any conversion of the substrate under this conditions.

Investigations of co-catalysts

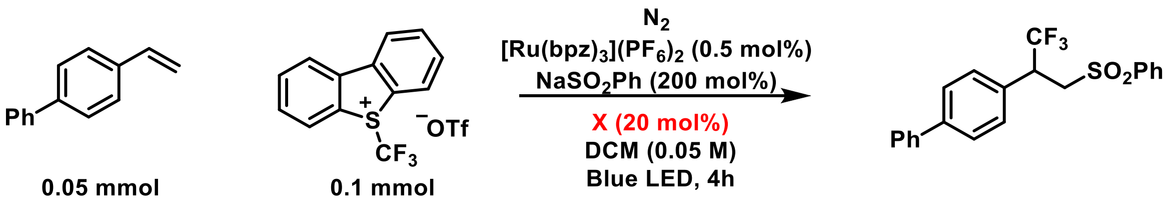

X	Yield (%)
$CuCl_2$	41
CuCl	30
$Cu(CH_3CN)PF_6$	0
$Cu(OTf)_2$	0
CuCN	31
$FeCl_2$	0
$MnCl_2$	0

Investigation of solvents

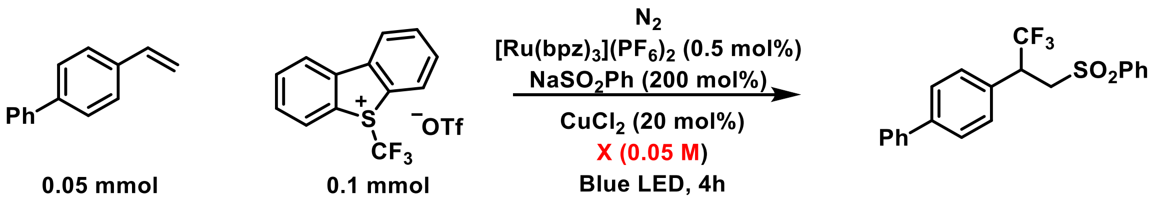

X	Yield (%)
MeCN	34
DMSO	trace
DCM	41
1,2-DCE	28
DMF	0
Acetone	trace
EtOAc	27
DCM **(0.1 M)**	42

1.4 Mechanistic investigation

1.4.1 Electrochemical measurements

The electrochemical redox potentials were obtained by using a Metrohm Autolab potentiostat-galvanostat PGSTAT204 fitted with a glassy carbon (GC) working electrode (diameter = 3 mm), an Ag/AgCl (3 M KCl) reference electrode and a Pt-foil counter electrode, attached to a PC by using Nova v2.1.5 software. 0.1 mmol samples were dissolved in 15 mL 0.1 M tetra-*n*-butylammonium hexafluorophosphate ($^{n}Bu_4NPF_6$) in dry, degassed DCM. Reductions were measured by scanning potentials in the negative direction and oxidations in the positive direction. The obtained value was referenced to Ag/AgCl (3 M KCl).

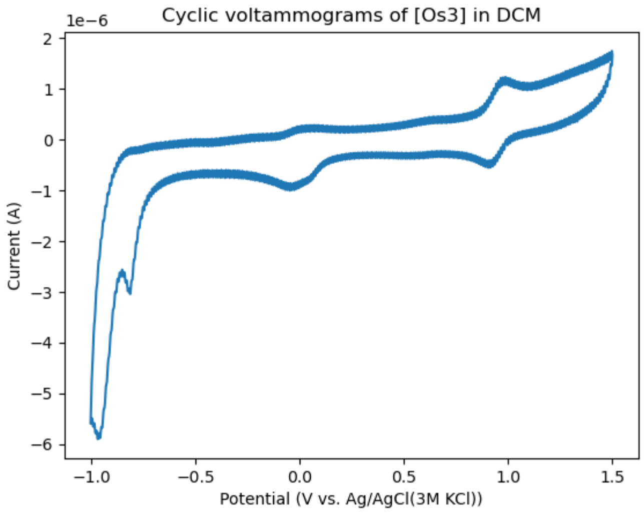

Supplementary Figure 1. Redox potential analysis of [Os]-catalyst *via* cyclic voltammetry.

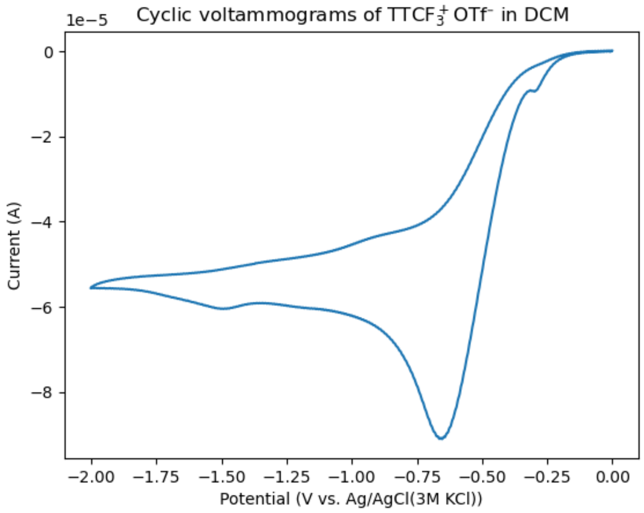

Supplementary Figure 2. Redox potential analysis of TT-$CF_3^{+}OTf^{-}$ *via* cyclic voltammetry.

The redox potentials of Os-photocatalyst and $TTCF_3^+OTF^-$ were measured in DCM (0.1 M). The E_{ox} and E_{red} of [Os] were 1.05 V and -0.82 V *vs.* Ag/AgCl (3 M KCl), respectively. Although the solvent was different from the reference, those values were consistent with the reference.[1] Therefore, the redox potential of the excited state was estimated same as the reference. The reduction potential of $TTCF_3^+OTF^-$ was ca. -0.69 V *vs.* Ag/AgCl (3 M KCl).

According to these redox potentials, we demonstrated that the excited state of photocatalyst $[Os^{II}]^*$ would exclusively undergo reduction by **I** to form the sulfonyl radical **II** (*Path A*) rather than oxidation by **IV** to generate the free $-CF_3$ radical **V** (*Path B*). *We highlighted the aspect of mechanism we verified in this part.*

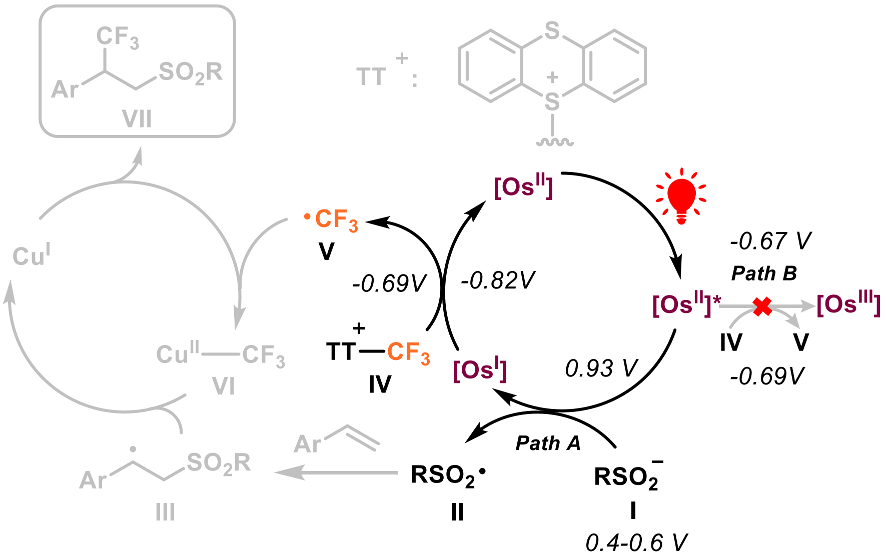

The obtained value was referenced to Ag/AgCl (3 M KCl).

1.4.2 Quenching experiment and radical probe experiment

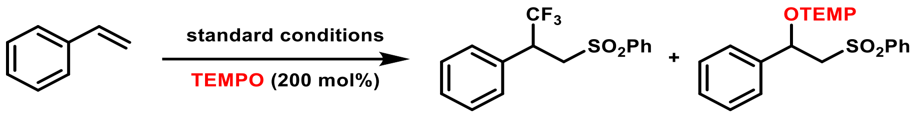

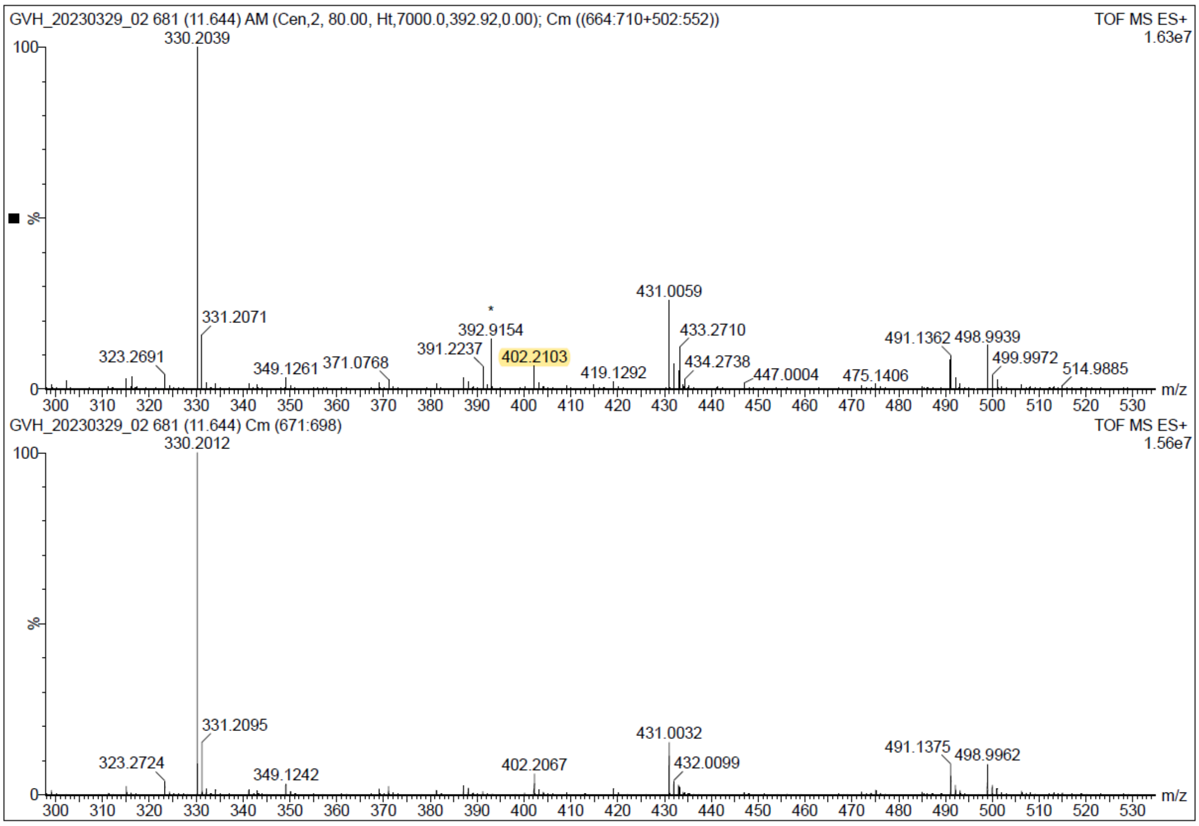

Supplementary Figure 3. HRMS analysis of carbon-centered radical (**III**) trapped by TEMPO.

The quenching experiment was carried out with 2 equiv. TEMPO and the carbon-centered radical **III** was captured by TEMPO which was detected by HRMS.

Radical probe experiment via ring-opening reaction

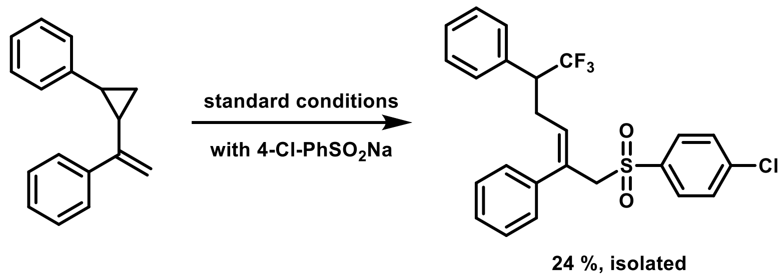

Fluorescence quenching experiments

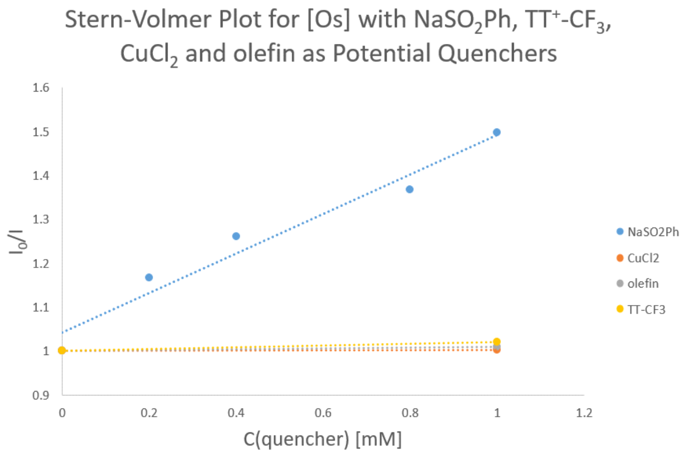

The radical probe experiment and Stern-Volmer analysis were also carried out. These results verified that the excited state of the photocatalyst $[Os^{II}]^*$ was quenched by the sulfinate anion and the following steps involved radical process. *We highlighted the aspect of mechanism that we had verified in this part.*

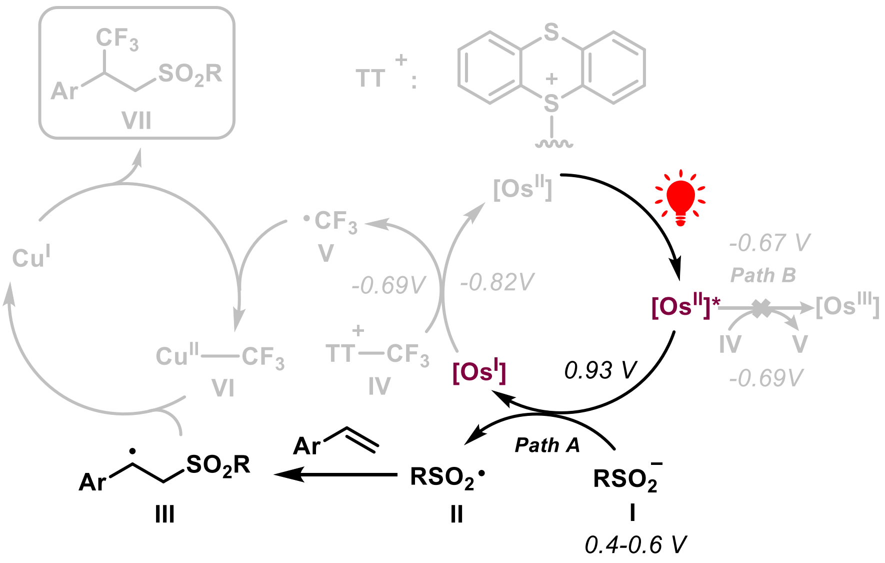

The obtained value was referenced to Ag/AgCl (3 M KCl).

1.4.3 Analysis of Cu-CF_3 complex

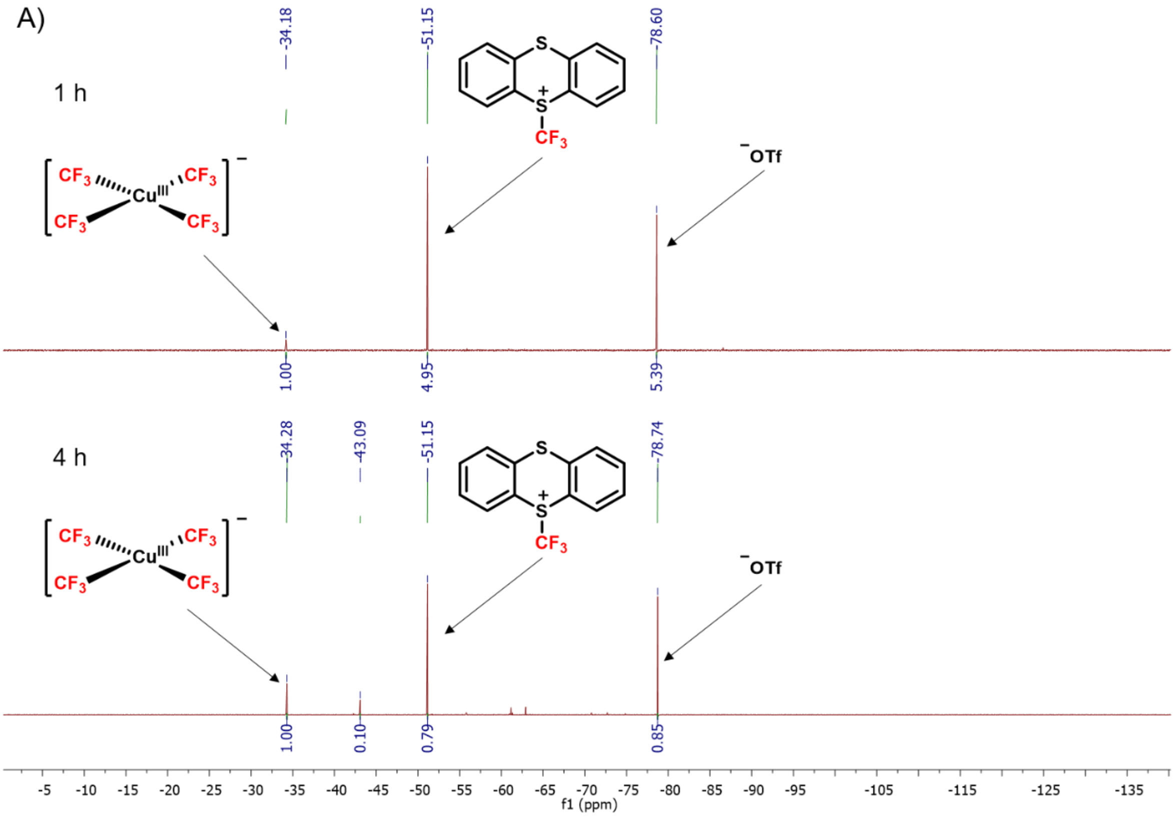

Supplementary Figure 4. ^{19}F NMR analysis of the reaction in the absence of styrene in 1 and 4 h.

To analyze the possible Cu-CF_3 active species, various control experiments were carried out. Initially, we attempted to detect the active species in the absence of any styrene. While no new peak corresponding to Cu^{II}-CF_3 was observed, we observed the presence of the $Cu^{III}(CF_3)_4$ anion peak.

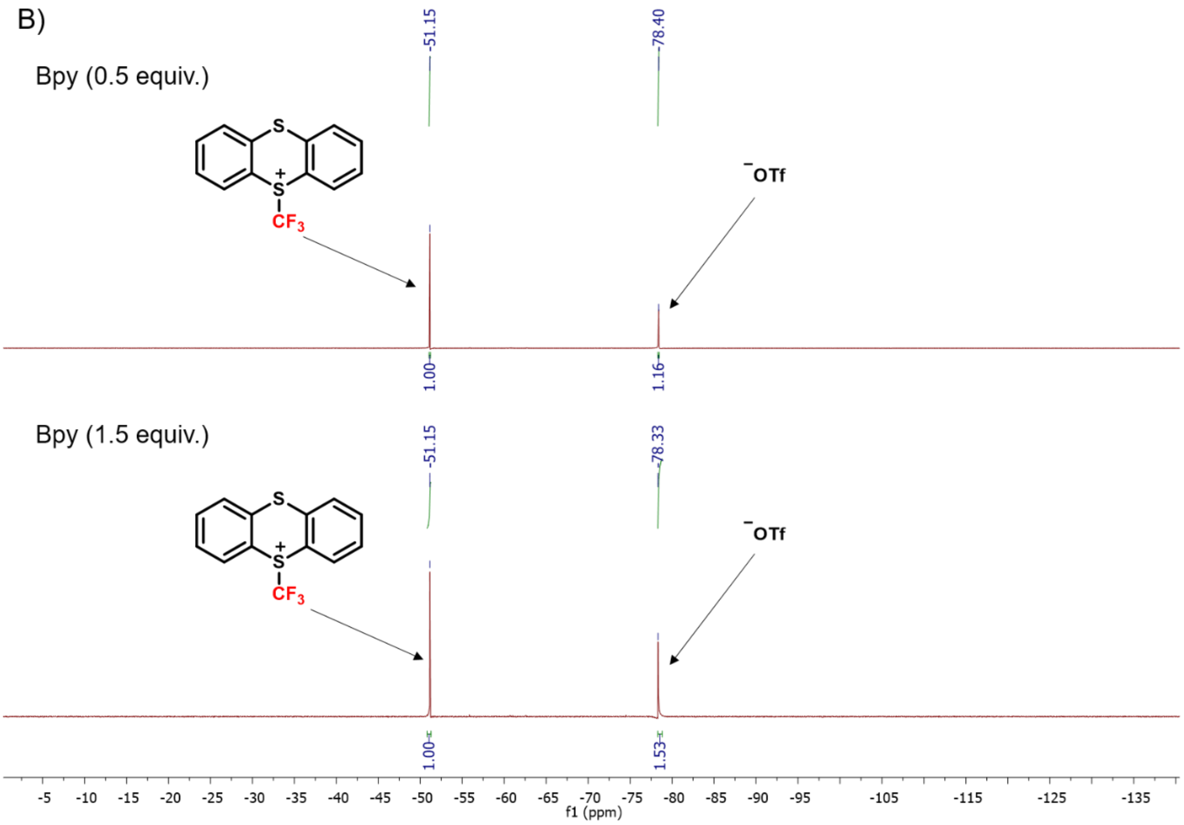

Supplementary Figure 5. ^{19}F NMR analysis of the reaction in the absence of styrene and adding 2,2'-bipyridine (bpy) ligand.

To capture the active species, the 2,2'-bipyridine (bpy) ligand was added into the system. Unfortunately, only peak of $TTCF_3^+OTF^-$ was observed in ^{19}F NMR. Therefore, it was necessary to examine whether the $Cu^{III}(CF_3)_4$ anion complex was the active species.

C)

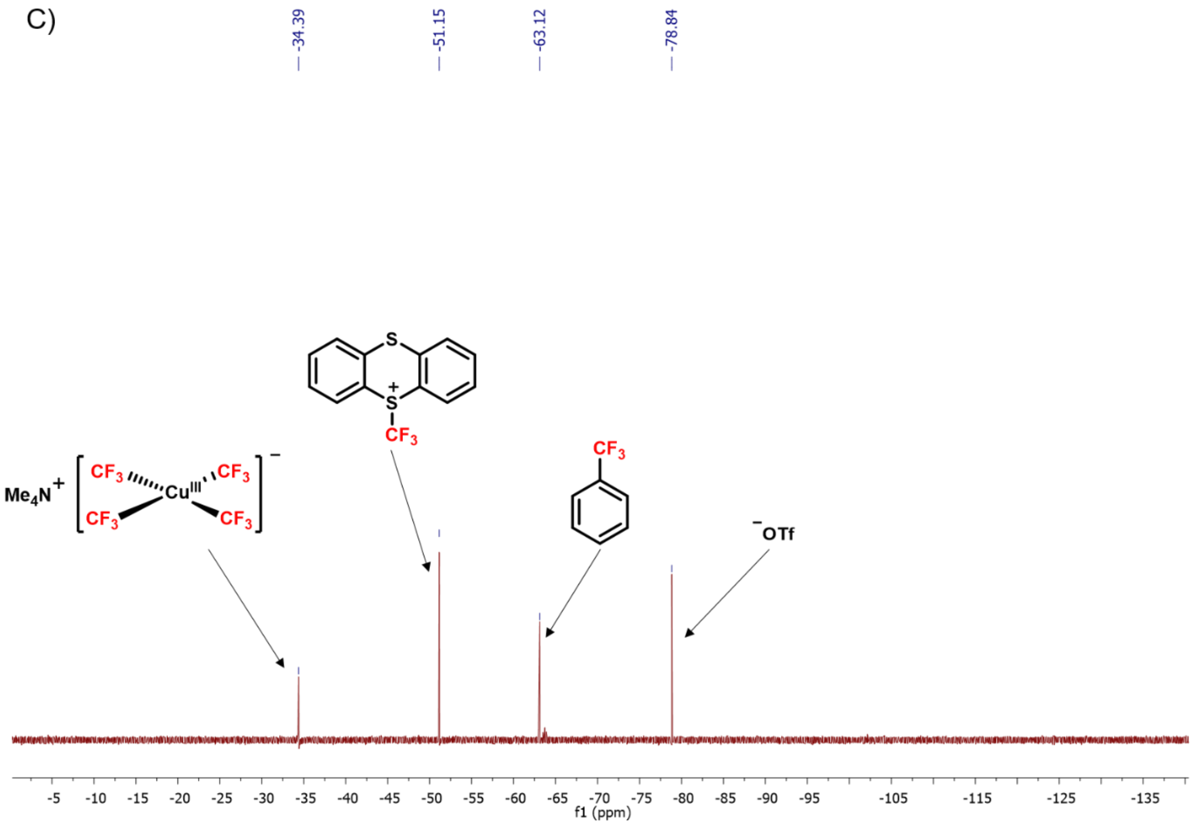

Supplementary Figure 6. ^{19}F NMR analysis of the reaction with $Me_4NCu^{III}(CF_3)_4$ complex instead of $CuCl_2$.

The styrene was added to system, however, no desired sulfonyltrifluoromethylated product was obtained. To further examine the $Cu^{III}(CF_3)_4$ anion complex, we synthesized stable $Me_4NCu^{III}(CF_3)_4$ complex according to the reference.[2] As expected, no product was obtained by using $Me_4NCu^{III}(CF_3)_4$ complex instead of $CuCl_2$.

D)

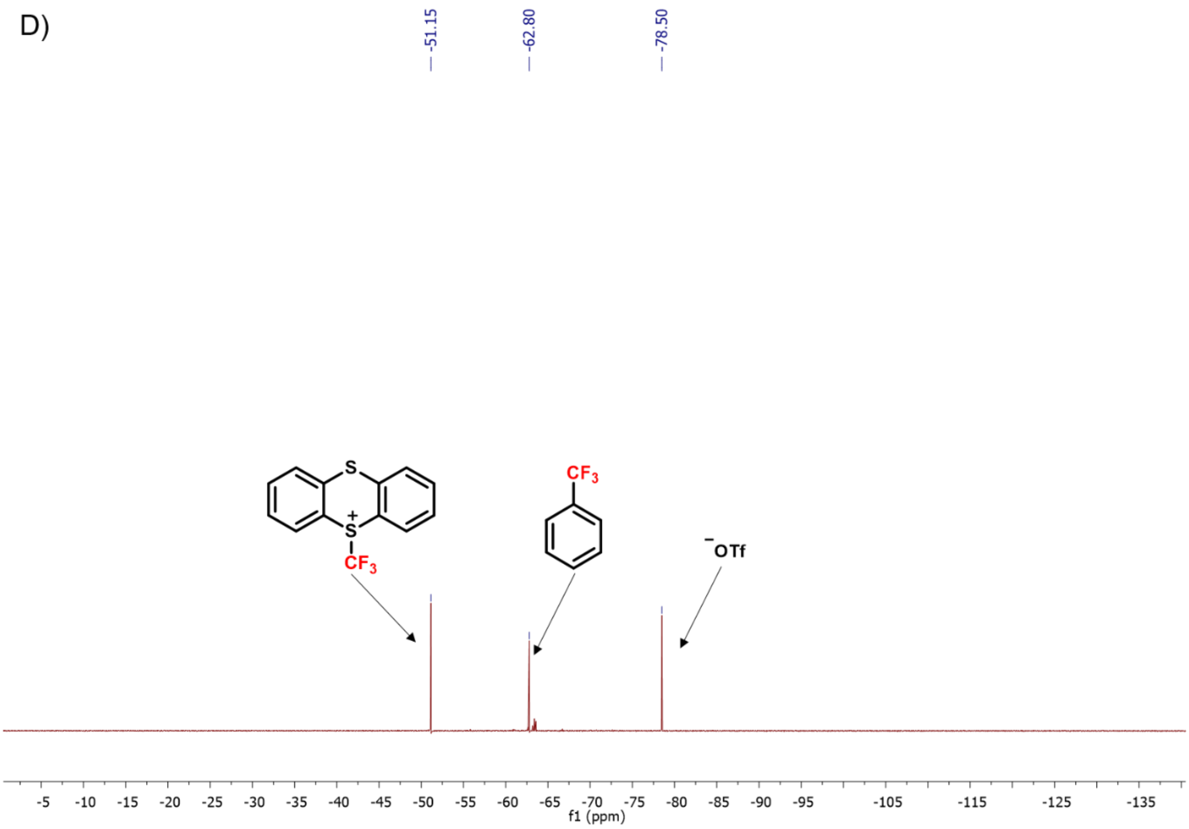

Supplementary Figure 7. ^{19}F NMR analysis of the reaction with fresh Cu^0 powder instead of $CuCl_2$.

Fresh Cu^0 powder was also used as catalyst instead of $CuCl_2$, and no product was found in the system.

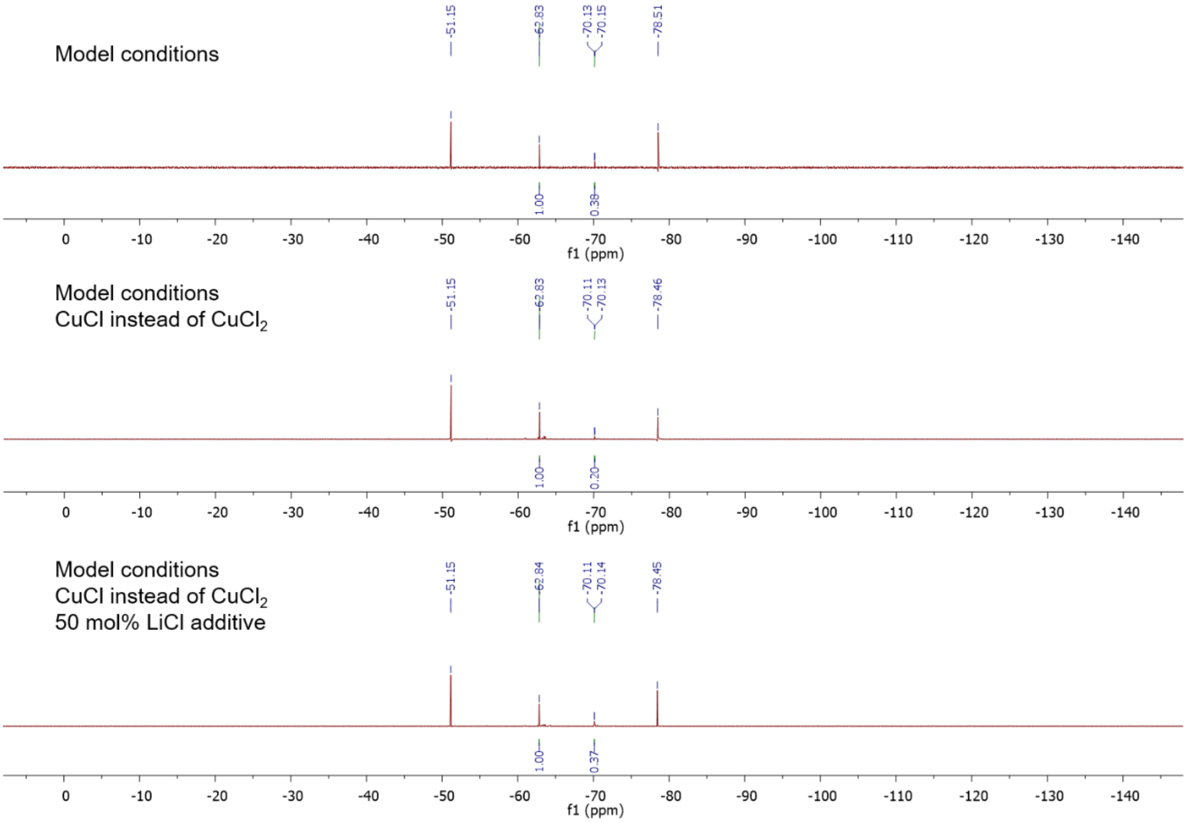

Supplementary Figure 8. Top: ^{19}F NMR analysis of the model reaction. Middle: ^{19}F NMR analysis of the reaction with CuCl instead of $CuCl_2$. Bottom: ^{19}F NMR analysis of the reaction with CuCl with 50 mol% LiCl instead of $CuCl_2$.

As elucidated in the optimization table, the introduction of CuCl instead of $CuCl_2$ resulted in a decrease in yield by approximately 20%. As expected, if Cu^{I} effectively captured the $-CF_3$ radical to form the active species, the yield should have been comparable to that obtained with Cu^{II} salts, which generated Cu^{I} *in situ*. We hypothesized that the concentration of chloride anion (Cl^-) might also play a crucial role in the reaction, as reported in the literature.[3]

Consequently, the addition of 50 mol% of LiCl was incorporated as an additive to the system, resulting in an increased yield comparable to that of the model reaction.

EPR analysis

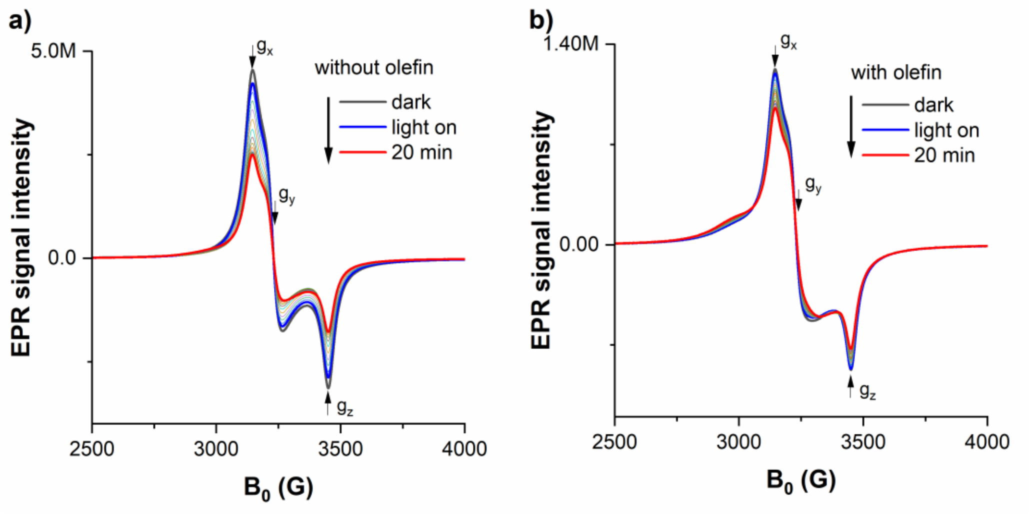

Supplementary Figure 9. Reaction conditions: $CuCl_2$ (2.7 mg), $TTCF_3^+OTF^-$ (9.5 mg), $NaSO_2Ph$ (5.9 mg) and $Os(bptpy)_2(PF_6)_2$ (0.6 mg) in 1 mL DCM in dark and under the irradiation at room temperature; a) without olefin; b) in the presence of 5.1 mg model olefin.

The EPR spectrum of the reaction mixture recorded in dark is characterized by three g values (g_x = 2.244 ; g_y = 2.187 ; g_z =2.047). The g values ($g_x > g_y > g_z$) are characteristic for pentacoordination Cu^{II} ion with a geometry intermediate between the square pyramid and trigonal bipyramid. Illuminating the mixture resulted in a decreasing of the Cu^{II} EPR signal (**Supplementary Figure 9**) due to the formation of EPR silent species, probably to Cu^I as suggested in **Figure 5d**. However, this effect is less pronounced in the presence of olefin, more probably due to reoxidation of Cu^I to Cu^{II} during the photocatalytic reaction. It seems to be that the reduction step of Cu^{II} to Cu^I is higher than reoxidation of Cu^I to Cu^{II} as the signal of Cu^{II} decreased with time.

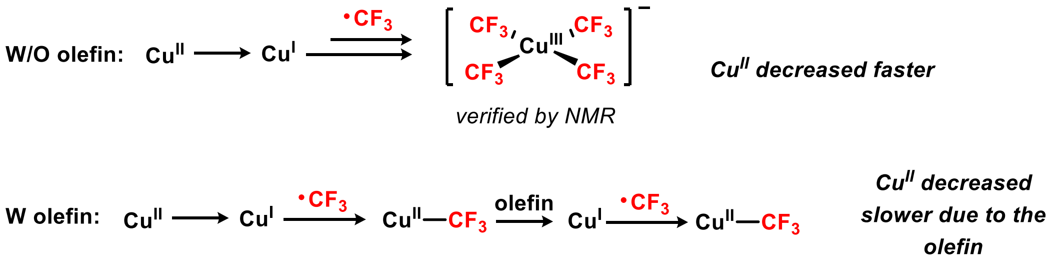

Based on the EPR and NMR analysis, we proposed that $Cu^{III}(CF_3)_4^-$complex would be formed in the absence of the olefin, which was also verified by NMR. Therefore, the transformation process of Cu^{II} to Cu^I is much faster. In contrast, due to the presence of the olefin, we propose Cu^{II}-CF_3 would transfer the CF_3 to the olefin and regenerate the Cu^I, indicating that it probably has additional transformation processes of Cu^I to Cu^{II}, which made the Cu^{II} EPR signal dropped slower.

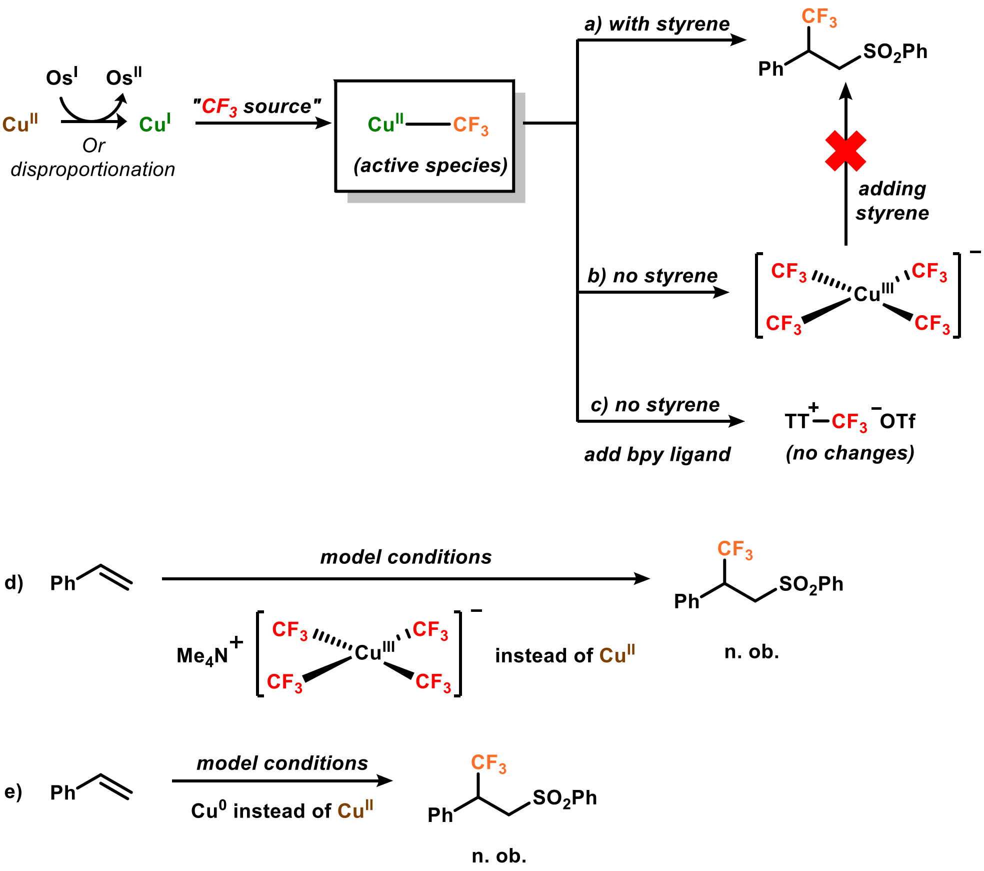

Supplementary Figure 10. The comprehensive analysis of Cu-CF_3 complexes.

Based on this analysis, we assumed that Cu^{II}-CF_3 (**VI**) was the possible active species for conducting the desired product *via* cross-coupling with **III**. The total mechanism of this reaction is proposed as follow:

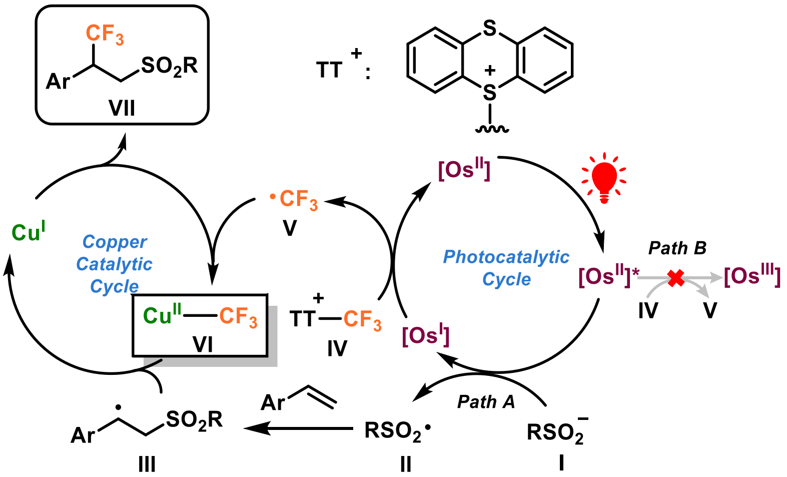

1.4.4 Quantum yield (QY) measurement

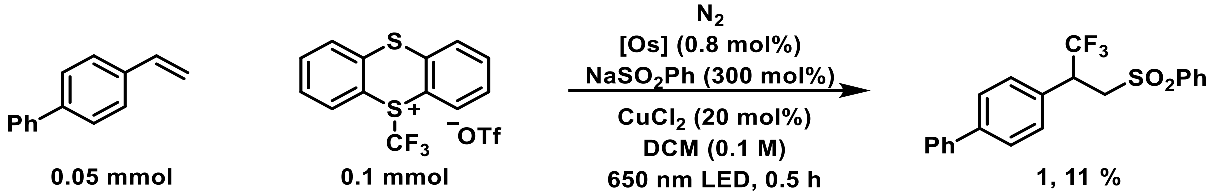

The light intensity of LED (λ = 650 nm) was 0.02 W/cm^2. QY for photocatalytic production of **1** was calculated using the following formula[4]:

$$\Phi = \frac{N_e}{N_p} \times 100\ \% = \frac{2 \times M \times N_a}{\frac{S \times P \times t}{h \times \frac{c}{\lambda}}} \times 100\ \% = \frac{2 \times M \times N_a \times h \times c}{S \times P \times t \times \lambda} \times 100\ \%$$

Where, M represents the amount of formed **1** (mol), N_a is the Avogadro constant 6.02×10^{23} mol^{-1}, h is the Planck constant 6.63×10^{-34} J·s, c is the light speed 3×10^{8} m·s^{-1}, S is the irradiation area, which is 0.88 cm^2, P is the light intensity (W·cm^{-2}), t is irradiation time (s), λ is the wavelength of light (m).

After calculation, the quantum yield (Φ) is 6.4%, which is much lower than 1. Therefore, this reaction process is a closed catalytic cycle.[5]

1.4.5 Examples of unsuccessful substrates

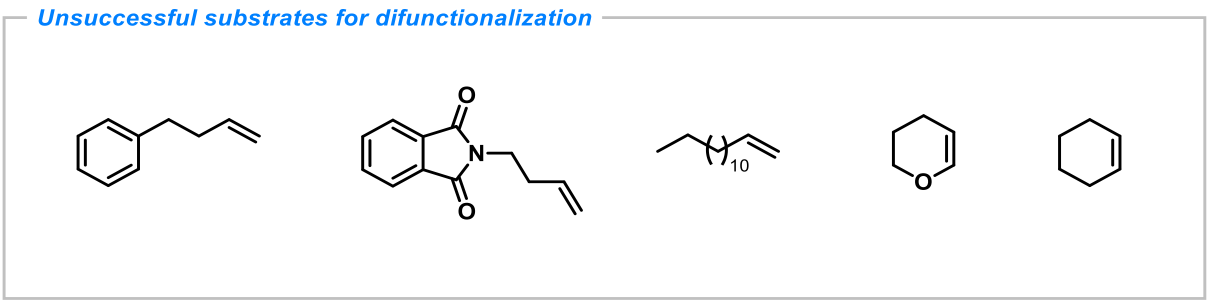

The examples of unsuccessful unactivated alkenes. After the addition of -SO_2R radical to the alkenes to form the corresponding carbon-centered radical, the unsuccessful cross-coupling between carbon-centered radical and Cu-CF_3 could be the cause of this limitation because hydrosulfonylated products could be observed in certain instances.

1.5 Experiments

1.5.1 Photocatalytic reaction setup

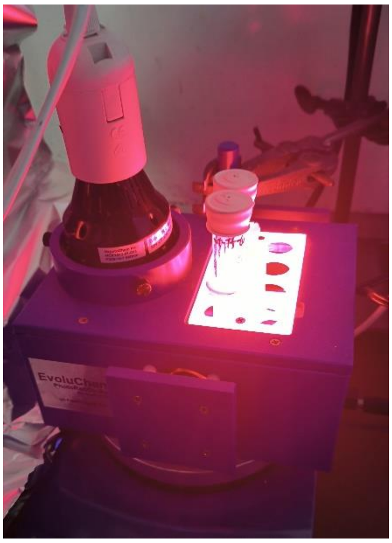

The reaction was carried out with the red LED light (EvoluChem™ LED 650PF HCK1012-XX-014 650 nm 20 mW/cm^2) in the EvoluChem PhotoRedOx Box. In the box, it containd an electric fan to cool down the reaction. The reaction holder supported 8 reaction vials simultaneously. The reaction mixture was stirred by using a magnetic stirring plate and stirring bar at 800 rpm on average.

1.5.2 Procedure for synthesis of TT-$CF_3^+OTF^-$

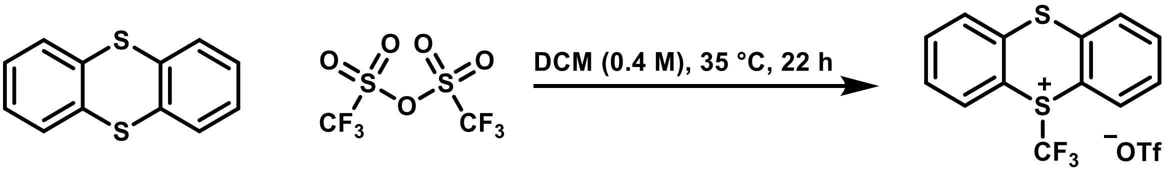

The S-(trifluoromethyl)thianthrenium triflate was prepared according to the literature.[6] A dried 250 mL round-bottom flask with magnetic stirring bar was charged with thianthrene (40 mmol, 1 equiv.) and DCM (100 mL, 0.4 M) under an ambient atmosphere. Subsequently, triflic anhydride (44 mmol, 1.1 equiv.) was added in one portion. The reaction mixture was stirred at 35 °C for 22 h. Followed by this, a saturated aqueous $NaHCO_3$ solution (50 mL) was added carefully. The organic layer was separated and concentrated in vacuo. Et_2O (50 mL) was added to the residue and the suspension was stirred vigorously at room temperature for 30 min. The mixture was allowed to stand for 5 minutes, and the solvent was decanted carefully. The resulting yellow slurry was concentrated to dryness in vacuo and TT-$CF_3^+OTF^-$ salts can be used without further purification.

1.5.3 Procedure for synthesis of Os(bptpy)$_2$(PF$_6$)$_2$

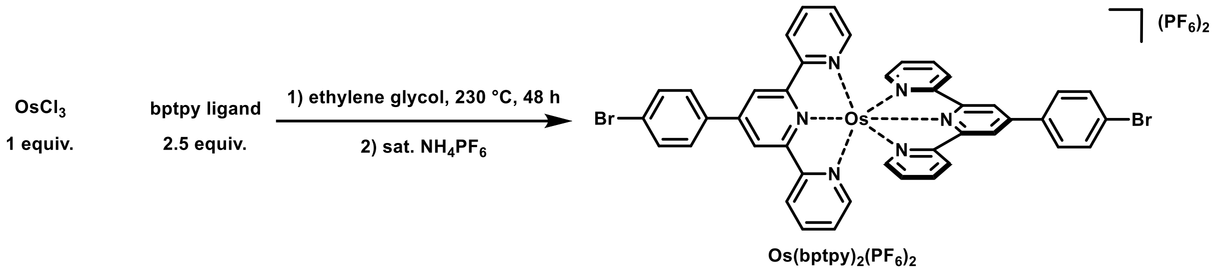

A dried 50 mL pressure tube with magnetic stirring bar was charged with $OsCl_3$ (1 equiv.) and bptpy ligand (2.5 equiv.), followed by the addition of anhydrous ethylene glycol (0.075 M) in glovebox. The tube was sealed and heated at 230 °C in a sand bath (*Caution*: a blast shield is required for all sealed tube reactions since pressure develops in the tube.). After 48 h, the reaction was allowed to cool to 60 °C. Then, NH_4PF_6 (10 equiv.) was added to the flask under an ambient atmosphere, followed by adding equal volume of distilled water. After 45 minutes, a dark precipitate was collected on filter paper and was washed with water, methanol, DCM and Et_2O to obtain the pure catalyst. The catalyst was dried under vacuum and was used without further purification.[1]

1.5.4 Procedure for the synthesis of olefins from phenols

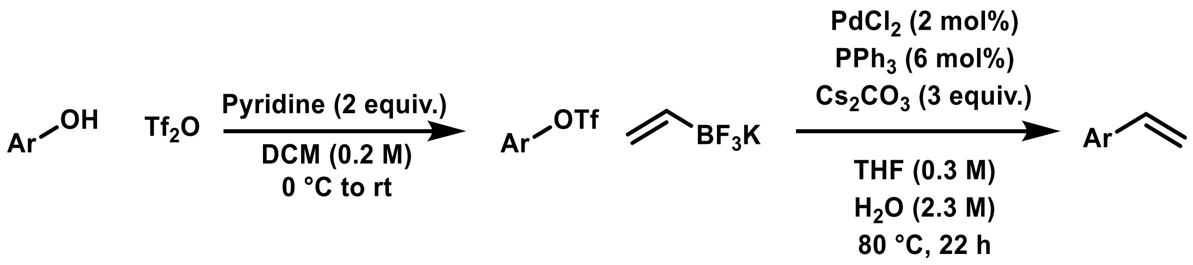

The olefin substrates were synthesized according to a reported procedure.[7] A dried Schlenk flask with magnetic stirring bar was charged with phenol (1.0 mmol, 1.0 equiv), after which the vessel was evacuated by using Schlenk techniques and was flushed with N_2 for three times. Under nitrogen atmosphere, DCM (5.0 mL, 0.20 M) and pyridine (2.0 mmol, 2.0 equiv) were added. The resulting mixture was cooled to 0 °C. Subsequently, Tf_2O (1.5 mmol, 1.5 equiv) was added dropwise over 5 minutes. The reaction mixture was then warmed to room temperature and stirred for 5 hours. After 5 h, the reaction mixture was quenched by adding distilled water (15 mL). The organic

phase was extracted by DCM (3 × 20 mL) and was concentrated in vacuo. Purification proceeded to obtain *triflate substrate via* flash column chromatography.

A dried two-necked flask with stirring bar was charged with *triflate substrate* (0.5 mmol, 1.00 equiv) from previous step, potassium vinyltrifluoroborate (0.6 mmol, 1.2 equiv), $PdCl_2$ (0.01 mmol, 2.00 mol%), PPh_3 (0.03 mmol, 6.00 mol%) and Cs_2CO_3 (1.5 mmol, 3.00 equiv), after which the vessel was evacuated by using Schlenk techniques and flushed with N_2 for three times. Distilled THF (2.00 mL) and degassed distilled water (0.25 mL) were added. After stirring at 80 °C for 22 h, the reaction mixture was quenched by adding DCM (15.0 mL) and water (20.0 mL). The layers were separated, and the aqueous layer was extracted with DCM (3 × 15 mL). The organic phase was extracted and concentrated in vacuo. The residue was purified by flash column chromatography.

1.5.5 Procedure for synthesis of olefins from acids

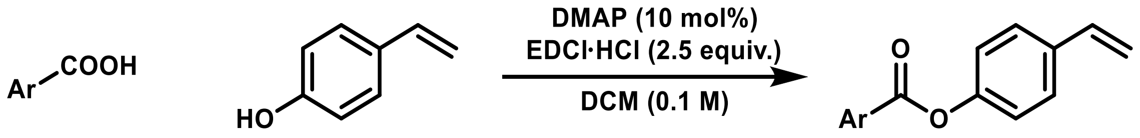

These olefins were prepared by following reported literature procedure.[8] A dried two-necked flask with stirring bar was charged with acid (2 mmol, 1 equiv.), DMAP (0.2 mmol, 10 mol%) and EDCI•HCl (5 mmol, 2.5 equiv.), after which the vessel was evacuated by using Schlenk techniques and flushed with N_2 for three times. Under nitrogen gas flow, 4-vinylphenol (2 mmol, 1 equiv.) and DCM (0.1 M) were added by using a syringe flushed with inert gas. The mixture was stirred at room temperature under N_2 until the reaction was completed which was checked by TLC monitoring. After the completion of the reaction, the mixture was diluted with water and the organic layer was separated, dried over anhydrous Na_2SO_4 and concentrated. Purification proceeded *via* flash column chromatography.

1.5.6 Procedure for sulfonyltrifluoromethylation of olefins

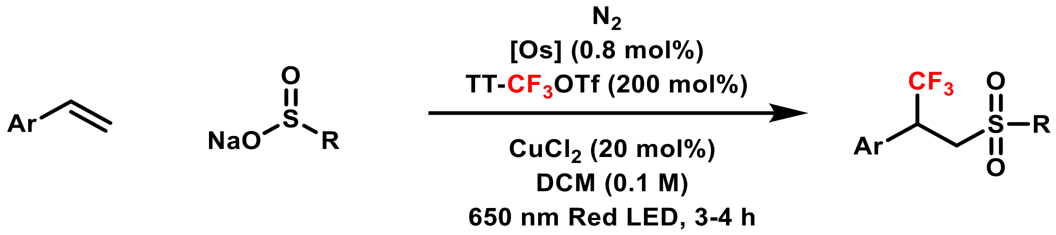

A dried reaction vial with magnetic stirring bar was charged with $Os(bptpy)_2(PF_6)_2$ (0.0008 mmol, 0.8 mol%), $CuCl_2$ (0.02 mmol, 20 mol%), TT-$CF_3^+OTF^-$ (0.2 mmol, 2 equiv.) and sodium sulfinate (0.3 mmol, 3 equiv.), after which the vessel was evacuated by using Schlenk techniques and flushed with N_2 for three times. Under nitrogen gas flow, olefin (0.1 mmol, 1 equiv.) (if liquid, otherwise added before flushing cycle) and dry DCM (0.1 M) were added by using a syringe flushed with inert gas. The resulting mixture was stirred for 3-4 h under the irradiation of red LED light (EvoluChem™ LED 650PF HCK1012-XX-014 650 nm 20 mW/cm²) in the EvoluChem PhotoRedOx Box. After completion of the reaction, the reaction mixture was quenched by adding distilled water (2 mL). The organic phase was extracted and concentrated in vacuo. 1,1,1-Trifluorotoluene was added as an internal standard to determine the NMR yield of the functionalised product through ^{19}F NMR. Purification proceeded *via* flash column chromatography.

1.6 Characterization

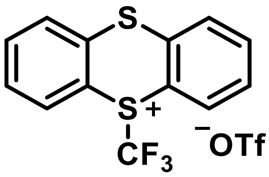

TT-CF_3^+OTF$^-$

^{1}H NMR (400 MHz, $CDCl_3$) δ 8.58 (dd, *J* = 8.1, 1.2 Hz, 1H), 7.93 – 7.87 (m, 1H), 7.82 (dd, *J* = 8.0, 1.3 Hz, 1H), 7.79 – 7.74 (m, 1H).

^{19}F NMR (376 MHz, $CDCl_3$) δ -51.11 (s), -78.39 (s).

^{13}C NMR (101 MHz, $CDCl_3$) δ 136.90, 136.81, 136.65, 130.33, 129.41, 124.43 (q, *J* = 337.2 Hz), 120.68 (q, *J* = 320.3 Hz), 108.95.

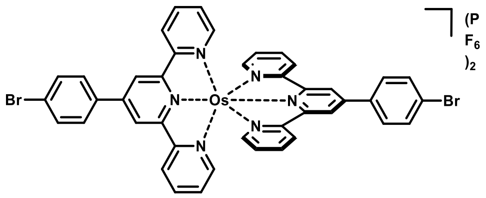

Os(bptpy)$_2$(PF$_6$)$_2$

^{1}H NMR (400 MHz, DMSO) δ 9.40 (s, 4H), 8.97 (d, *J* = 8.2 Hz, 4H), 8.27 (d, *J* = 8.5 Hz, 4H), 7.88 (d, *J* = 8.5 Hz, 4H), 7.81 (t, *J* = 7.8 Hz, 4H), 7.31 (d, *J* = 5.6 Hz, 4H), 7.09 (t, *J* = 6.5 Hz, 4H).

^{19}F NMR (376 MHz, DMSO) δ -70.18 (d, *J* = 711.2 Hz).

^{13}C NMR (101 MHz, DMSO) δ 160.12, 155.18, 152.96, 152.75, 138.30, 135.09, 132.58, 130.43, 128.47, 125.49, 124.64, 120.10.

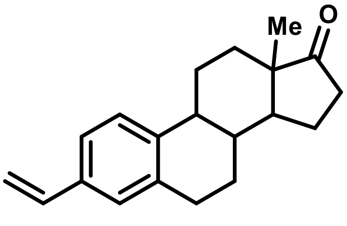

S1

^{1}H NMR (400 MHz, $CDCl_3$) δ 7.21 (dd, *J* = 17.6, 8.2 Hz, 2H), 7.12 (s, 1H), 6.68 – 6.55 (m, 1H), 5.69 (d, *J* = 17.6 Hz, 1H), 5.18 (d, *J* = 10.9 Hz, 1H), 2.90 (dd, *J* = 8.6, 3.9 Hz, 2H), 2.44 (ddd, *J* = 17.4, 16.0, 6.8 Hz, 2H), 2.25 (dd, *J* = 13.6, 7.1 Hz, 1H), 2.19 – 1.89 (m, 4H), 1.67 – 1.33 (m, 6H), 0.89 (s, 3H).

^{13}C NMR (101 MHz, $CDCl_3$) δ 220.9, 139.57, 136.64, 136.57, 135.24, 126.90, 125.55, 123.64, 113.16, 50.55, 47.98, 44.47, 38.20, 35.86, 31.65, 29.41, 26.53, 25.76, 21.62, 13.88.

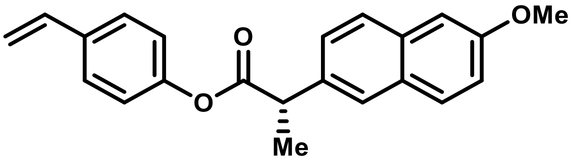

S2

^{1}H NMR (400 MHz, $CDCl_3$) δ 7.80 – 7.69 (m, 3H), 7.50 (dd, *J* = 8.5, 1.9 Hz, 1H), 7.40 – 7.31 (m, 2H), 7.16 (dt, *J* = 7.2, 2.4 Hz, 2H), 7.00 – 6.88 (m, 2H), 6.67 (dd, *J* = 17.6, 10.9 Hz, 1H), 5.67 (dd, *J* = 17.6, 0.8 Hz, 1H), 5.21 (dd, *J* = 10.9, 0.7 Hz, 1H), 4.09 (q, *J* = 7.1 Hz, 1H), 3.93 (s, 3H), 1.69 (d, *J* = 7.1 Hz, 3H).
^{13}C NMR (101 MHz, $CDCl_3$) δ 173.10, 157.80, 150.42, 135.91, 135.31, 135.14, 133.85, 129.33, 129.03, 127.38, 127.07, 126.15, 126.13, 121.45, 119.11, 113.93, 105.67, 55.34, 45.61, 18.52.

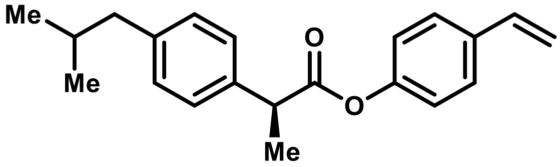

S3

^{1}H NMR (400 MHz, $CDCl_3$) δ 7.41 – 7.34 (m, 2H), 7.32 – 7.23 (m, 2H), 7.15 (d, *J* = 8.1 Hz, 2H), 6.99 – 6.93 (m, 2H), 6.68 (dd, *J* = 17.6, 10.9 Hz, 1H), 5.68 (dd, *J* = 17.6, 0.7 Hz, 1H), 5.22 (dd, *J* = 10.9, 0.6 Hz, 1H), 3.93 (q, *J* = 7.1 Hz, 1H), 2.48 (d, *J* = 7.2 Hz, 2H), 1.97 – 1.79 (m, 1H), 1.61 (d, *J* = 7.2 Hz, 3H), 0.92 (d, *J* = 6.6 Hz, 6H).
^{13}C NMR (101 MHz, $CDCl_3$) δ 173.16, 150.47, 140.83, 137.24, 135.94, 135.27, 129.51, 127.22, 127.07, 121.46, 113.90, 45.29, 45.07, 30.18, 22.39, 18.53.

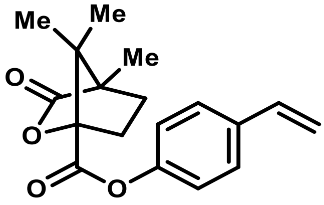

S4

¹H NMR (400 MHz, $CDCl_3$) δ 7.47 – 7.37 (m, 2H), 7.15 – 7.06 (m, 2H), 6.71 (dd, *J* = 17.6, 10.9 Hz, 1H), 5.72 (dd, *J* = 17.6, 0.5 Hz, 1H), 5.27 (d, *J* = 10.9 Hz, 1H), 2.57 (ddd, *J* = 13.5, 10.8, 4.3 Hz, 1H), 2.20 (ddd, *J* = 13.6, 9.3, 4.6 Hz, 1H), 1.99 (ddd, *J* = 13.2, 10.8, 4.6 Hz, 1H), 1.77 (ddd, *J* = 13.4, 9.4, 4.3 Hz, 1H), 1.16 (d, *J* = 7.6 Hz, 6H), 1.11 (s, 3H).
¹³C NMR (101 MHz, $CDCl_3$) δ 177.79, 166.08, 149.49, 135.71, 127.31, 121.36, 114.49, 90.83, 54.91, 54.68, 30.78, 28.99, 16.89, 9.73.

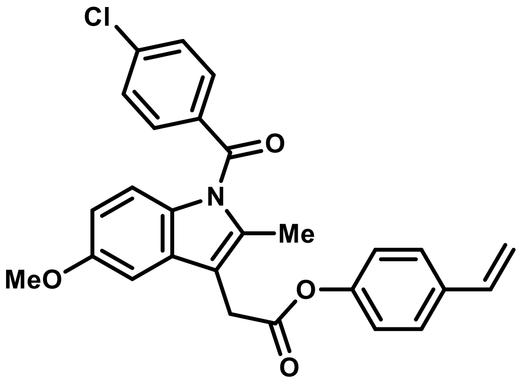

S5

¹H NMR (400 MHz, $CDCl_3$) δ 7.74 – 7.62 (m, 2H), 7.55 – 7.45 (m, 2H), 7.41 – 7.35 (m, 2H), 7.04 (ddd, *J* = 11.2, 6.9, 2.5 Hz, 3H), 6.91 (d, *J* = 9.0 Hz, 1H), 6.75 – 6.62 (m, 2H), 5.69 (dd, *J* = 17.6, 0.6 Hz, 1H), 5.24 (dd, *J* = 10.9, 0.5 Hz, 1H), 3.90 (s, 2H), 3.84 (s, 3H), 2.46 (s, 3H).
¹³C NMR (101 MHz, $CDCl_3$) δ 169.24, 168.31, 156.18, 150.26, 139.37, 136.22, 135.83, 135.52, 133.88, 131.21, 130.90, 130.52, 129.16, 127.16, 121.44, 115.03, 114.15, 112.00, 111.85, 101.28, 55.75, 30.59, 13.42.

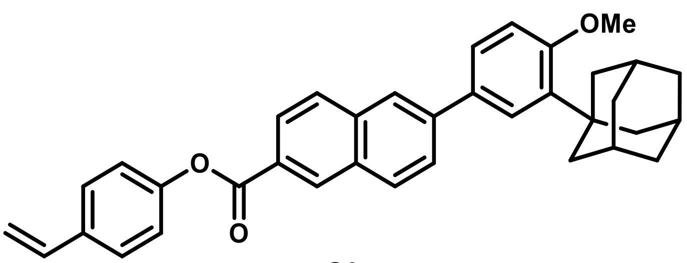

S6

¹H NMR (400 MHz, $CDCl_3$) δ 8.78 (s, 1H), 8.20 (d, *J* = 8.6 Hz, 1H), 8.01 (dd, *J* = 23.4, 9.1 Hz, 3H), 7.83 (d, *J* = 8.5 Hz, 1H), 7.62 (s, 1H), 7.57 (d, *J* = 8.4 Hz, 1H), 7.50 (d, *J* = 7.6 Hz, 1H), 7.24 (d, *J* = 8.4 Hz, 2H), 7.01 (d, *J* = 8.5 Hz, 1H), 6.75 (dd, *J* = 17.7,

10.8 Hz, 1H), 5.75 (d, J = 17.5 Hz, 1H), 5.28 (d, J = 10.8 Hz, 1H), 3.92 (d, J = 1.1 Hz, 3H), 2.20 (s, 6H), 2.11 (s, 3H), 1.81 (s, 6H).

^{13}C NMR (101 MHz, $CDCl_3$) δ 165.37, 159.05, 150.69, 141.83, 139.10, 136.31, 136.01, 135.45, 132.48, 131.72, 131.27, 129.84, 128.46, 127.29, 126.68, 126.23, 126.02, 125.82, 125.77, 124.78, 121.85, 114.03, 112.18, 55.19, 40.66, 37.26, 37.16, 29.15.

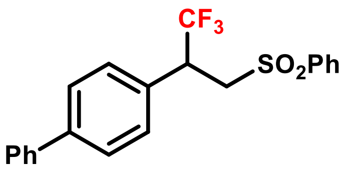

1

^{1}H NMR (400 MHz, $CDCl_3$) δ 7.61 – 7.57 (m, 2H), 7.52 – 7.42 (m, 5H), 7.40 – 7.35 (m, 3H), 7.34 – 7.28 (m, 2H), 7.17 (d, J = 8.2 Hz, 2H), 4.08 – 3.98 (m, 1H), 3.80 (qd, J = 14.7, 6.8 Hz, 2H).

^{19}F NMR (376 MHz, $CDCl_3$) δ -69.95 (d, J = 9.1 Hz).

^{13}C NMR (101 MHz, $CDCl_3$) δ 141.84, 140.18, 139.12, 133.47, 129.84, 129.56, 129.04, 128.88, 127.88, 127.72, 127.42, 127.02, 126.80, 55.48, 45.67 (q, J = 28.6 Hz).

HRMS (ESI$^+$, *m/z*) [M+H$^+$] calcd. for $C_{21}H_{17}SO_2F_3$ 391.0974, found 391.0976.

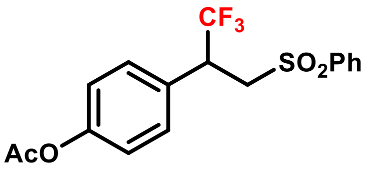

2

^{1}H NMR (400 MHz, $CDCl_3$) δ 7.62 – 7.48 (m, 3H), 7.43 – 7.30 (m, 2H), 7.11 (d, J = 8.5 Hz, 2H), 6.96 – 6.86 (m, 2H), 4.08 – 3.92 (m, 1H), 3.74 (m, 2H), 2.29 (s, 3H).

^{19}F NMR (376 MHz, $CDCl_3$) δ -70.06 (d, J = 9.0 Hz).

^{13}C NMR (101 MHz, $CDCl_3$) δ 168.81, 151.11, 138.91, 133.84, 130.16, 129.24, 128.49, 127.77, 126.66, 121.95, 55.50, 45.39 (q, J = 29.0 Hz), 21.10.

HRMS (ESI$^+$, *m/z*) [M+H$^+$] calcd. for $C_{17}H_{15}SO_4F_3$ 373.0716, found 373.0718.

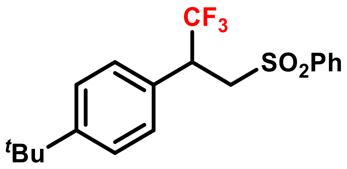

3

^{1}H NMR (400 MHz, $CDCl_3$) δ 7.59 – 7.52 (m, 2H), 7.48 – 7.39 (m, 1H), 7.33 – 7.22 (m, 2H), 7.20 – 7.08 (m, 2H), 6.99 (d, *J* = 8.3 Hz, 2H), 3.96 (pd, *J* = 9.2, 3.3 Hz, 1H), 3.85 – 3.68 (m, 2H), 1.27 (s, 9H).
^{19}F NMR (376 MHz, $CDCl_3$) δ -70.14 (d, *J* = 9.1 Hz).
^{13}C NMR (101 MHz, $CDCl_3$) δ 151.77, 139.13, 133.45, 128.92, 128.74, 127.87, 127.75, 126.86, 125.64, 55.38, 45.54 (q, *J* = 28.5 Hz), 34.49, 31.20.
HRMS (ESI^+, *m/z*) [$M+H^+$] calcd. for $C_{19}H_{21}SO_2F_3$ 371.1287, found 371.1292. [$M+Na^+$] 393.1107, found 393.1114.

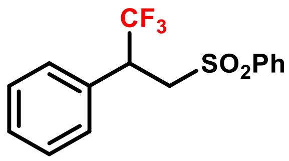

4

^{1}H NMR (400 MHz, $CDCl_3$) δ 7.62 – 7.58 (m, 2H), 7.50 (ddd, *J* = 8.7, 2.4, 1.2 Hz, 1H), 7.38 – 7.32 (m, 2H), 7.25 – 7.17 (m, 3H), 7.13 (d, *J* = 7.4 Hz, 2H), 3.98 (pd, *J* = 9.1, 3.9 Hz, 1H), 3.83 – 3.69 (m, 2H).
^{19}F NMR (376 MHz, $CDCl_3$) δ -70.02 (d, *J* = 9.1 Hz).
^{13}C NMR (101 MHz, $CDCl_3$) δ 139.07, 133.65, 131.10, 129.12, 129.08, 128.91, 128.77, 127.85, 126.80, 55.42, 45.86 (q, *J* = 28.7 Hz).
HRMS (ESI^+, *m/z*) [$M+H^+$] calcd. for $C_{15}H_{13}SO_2F_3$ 315.0661, found 315.0668.

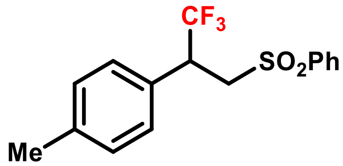

5

^{1}H NMR (400 MHz, $CDCl_3$) δ 7.65 – 7.58 (m, 1H), 7.57 – 7.46 (m, 1H), 7.41 – 7.29 (m, 1H), 7.03 – 6.98 (m, 2H), 3.93 (pt, *J* = 8.4, 4.1 Hz, 1H), 3.81 – 3.65 (m, 1H), 2.31 – 2.25 (m, 2H).
^{19}F NMR (376 MHz, $CDCl_3$) δ -70.16 (d, *J* = 9.1 Hz).
^{13}C NMR (101 MHz, $CDCl_3$) δ 139.16, 138.79, 133.45, 129.44, 129.01, 128.98, 128.06, 127.88, 124.07, 55.54, 45.44 (q, J = 28.6 Hz), 21.04.
HRMS (ESI^+, *m/z*) [$M+H^+$] calcd. for $C_{16}H_{15}SO_2F_3$ 329.0818, found 329.0827.

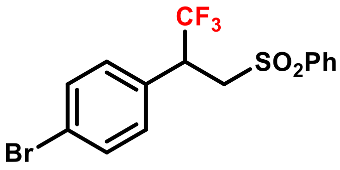

6

^{1}H NMR (400 MHz, $CDCl_3$) δ 7.62 – 7.52 (m, 3H), 7.45 – 7.36 (m, 2H), 7.35 – 7.29 (m, 2H), 7.00 (d, *J* = 8.4 Hz, 2H), 4.04 – 3.87 (m, 1H), 3.78 – 3.64 (m, 2H).

^{19}F NMR (376 MHz, $CDCl_3$) δ -70.04 (d, *J* = 9.0 Hz).

^{13}C NMR (101 MHz, $CDCl_3$) δ 138.99, 133.74, 131.97, 130.76, 130.03, 129.22, 127.81, 126.47, 123.34, 55.25, 45.40.

HRMS (ESI^+, *m/z*) [$M+H^+$] calcd. for $C_{15}H_{12}SO_2F_3Br$ 392.9766, found 392.9778. [$M+Na^+$] 414.9586, found 414.9603.

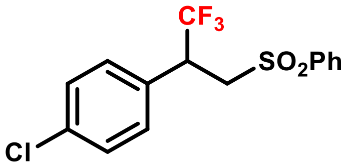

7

^{1}H NMR (400 MHz, $CDCl_3$) δ 7.67 – 7.50 (m, 1H), 7.45 – 7.33 (m, 1H), 7.22 – 7.13 (m, 1H), 7.07 (d, *J* = 8.4 Hz, 1H), 4.03 – 3.89 (m, 1H), 3.72 (d, *J* = 7.0 Hz, 1H).

^{19}F NMR (376 MHz, $CDCl_3$) δ -70.07 (d, *J* = 8.9 Hz).

^{13}C NMR (101 MHz, $CDCl_3$) δ 139.01, 135.17, 133.76, 130.47, 129.55, 129.20, 129.01, 127.82, 126.55, 55.31, 45.30.

HRMS (ESI^+, *m/z*) [$M+H^+$] calcd. for $C_{15}H_{12}SO_2F_3Cl$ 349.0271, found 349.0275. [$M+Na^+$] 371.0091, found 371.0099.

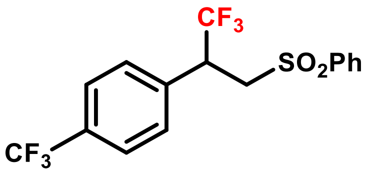

8

^{1}H NMR (400 MHz, $CDCl_3$) δ 7.60 – 7.55 (m, 2H), 7.55 – 7.49 (m, 1H), 7.45 (d, *J* = 8.2 Hz, 2H), 7.34 (dd, *J* = 10.8, 4.9 Hz, 2H), 7.27 (m, 2H), 4.07 (pd, *J* = 8.9, 4.9 Hz, 1H), 3.83 – 3.71 (m, 2H).

^{19}F NMR (376 MHz, $CDCl_3$) δ -63.05, -69.78 (d, *J* = 8.8 Hz).

^{13}**C NMR** (101 MHz, $CDCl_3$) δ 138.88, 134.96, 134.06, 133.91, 129.68, 129.22, 127.76, 125.73, 125.69, 123.65, 55.08, 45.75 (d, *J* = 28.8 Hz).

HRMS (ESI$^+$, *m/z*) [M+Na$^+$] calcd. for $C_{16}H_{12}SO_2F_6$ 405.0354, found 405.0351.

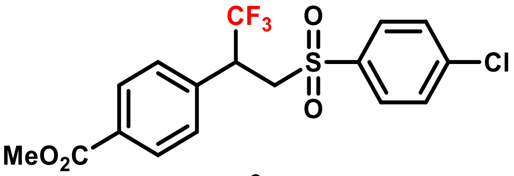

9

^{1}**H NMR** (400 MHz, $CDCl_3$) δ 7.93 – 7.86 (m, 2H), 7.56 – 7.48 (m, 2H), 7.35 – 7.29 (m, 2H), 7.22 (d, *J* = 8.2 Hz, 2H), 4.04 (pd, *J* = 8.8, 5.0 Hz, 1H), 3.93 (s, 3H), 3.80 – 3.69 (m, 2H).

^{19}**F NMR** (376 MHz, $CDCl_3$) δ -69.68 (d, *J* = 8.8 Hz).

^{13}**C NMR** (101 MHz, $CDCl_3$) δ 166.12, 140.90, 137.26, 135.70, 130.97, 130.01, 129.50, 129.32, 126.41, 126.13, 55.29, 52.32, 45.77 (d, *J* = 29.0 Hz).

HRMS (ESI$^+$, *m/z*) [M+H$^+$] calcd. for $C_{17}H_{14}SO_4F_3Cl$ 407.0326, found 407.0330.

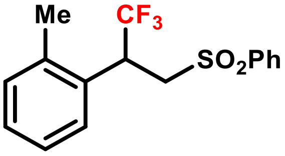

10

^{1}**H NMR** (400 MHz, $CDCl_3$) δ 7.60 – 7.52 (m, 2H), 7.51 – 7.44 (m, 1H), 7.36 – 7.28 (m, 2H), 7.11 (ddd, *J* = 11.3, 9.2, 4.5 Hz, 2H), 6.90 (dt, *J* = 7.9, 4.4 Hz, 2H), 4.40 (pd, *J* = 9.1, 3.5 Hz, 1H), 3.79 (qd, *J* = 14.7, 6.8 Hz, 2H), 2.45 (s, 3H).

^{19}**F NMR** (376 MHz, $CDCl_3$) δ -69.97 (d, *J* = 9.0 Hz).

^{13}**C NMR** (101 MHz, $CDCl_3$) δ 139.20, 138.15, 133.55, 130.82, 129.46, 129.00, 128.65, 127.54, 127.17, 127.05, 126.27, 55.69, 40.38 (d, *J* = 29.8 Hz), 19.84.

HRMS (ESI$^+$, *m/z*) [M+H$^+$] calcd. for $C_{16}H_{15}SO_2F_3$ 329.0818, found 329.0821.

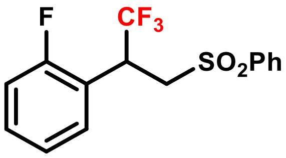

11

^{1}H NMR (400 MHz, $CDCl_3$) δ 7.68 (dt, *J* = 8.6, 1.6 Hz, 2H), 7.57 – 7.48 (m, 1H), 7.41 – 7.36 (m, 2H), 7.28 – 7.21 (m, 1H), 7.14 (t, *J* = 7.4 Hz, 1H), 7.04 – 6.98 (m, 1H), 6.97 – 6.88 (m, 1H), 4.36 – 4.25 (m, 1H), 3.87 (dd, *J* = 14.7, 10.9 Hz, 1H), 3.71 (dd, *J* = 14.7, 2.6 Hz, 1H).

^{19}F NMR (376 MHz, $CDCl_3$) δ -70.04 (dd, *J* = 8.9, 6.0 Hz), -115.26.

^{13}C NMR (101 MHz, $CDCl_3$) δ 162.31, 159.83, 138.56, 133.89, 130.91/130.83, 129.95, 129.18, 127.94, 124.49/ 124.45, 118.48/118.36, 116.13/115.91, 54.11, 39.19 (q, *J* = 30.0 Hz).

HRMS (ESI$^+$, *m/z*) [M+H$^+$] calcd. for $C_{15}H_{12}SO_2F_4$ 333.0567, found 333.0572.

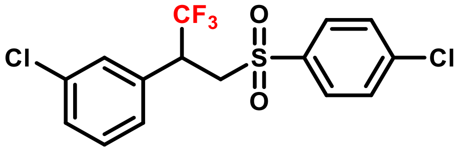

12

^{1}H NMR (400 MHz, $CDCl_3$) δ 7.54 – 7.46 (m, 2H), 7.38 – 7.31 (m, 2H), 7.26 (ddd, *J* = 8.0, 1.9, 1.1 Hz, 1H), 7.17 (t, *J* = 7.8 Hz, 2H), 7.09 – 6.97 (m, 2H), 4.00 – 3.87 (m, 1H), 3.80 – 3.66 (m, 2H).

^{19}F NMR (376 MHz, $CDCl_3$) δ -69.93 (d, *J* = 8.8 Hz).

^{13}C NMR (101 MHz, $CDCl_3$) δ 140.89, 137.21, 134.87, 132.62, 130.14, 129.43, 129.22, 127.59, 126.38, 123.59, 55.13, 45.67 (q, *J* = 29.0 Hz).

HRMS (ESI$^+$, *m/z*) [M+Na$^+$] calcd. for $C_{15}H_{11}SO_2F_3Cl_2$ 404.9701, found 404.9704.

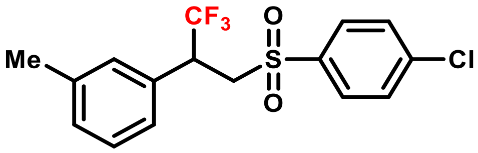

13

^{1}H NMR (400 MHz, $CDCl_3$) δ 7.49 – 7.38 (m, 2H), 7.25 (dt, *J* = 4.4, 1.9 Hz, 2H), 7.12 – 7.03 (m, 2H), 6.92 (d, *J* = 7.2 Hz, 1H), 6.76 (s, 1H), 3.98 – 3.84 (m, 1H), 3.82 – 3.70 (m, 2H), 2.21 (s, 3H).

^{19}F NMR (376 MHz, $CDCl_3$) δ -70.07 (d, *J* = 8.9 Hz).

^{13}C NMR (101 MHz, $CDCl_3$) δ 140.36, 138.57, 137.44, 130.47, 130.25, 129.66, 129.60, 129.26, 129.11, 128.76, 126.51, 55.38, 45.98 (dd, J = 57.6, 28.9 Hz), 21.10.
HRMS (ESI$^+$, *m/z*) [M+Na$^+$] calcd. for $C_{16}H_{14}SO_2F_3Cl$ 385.0247, found 385.0253.

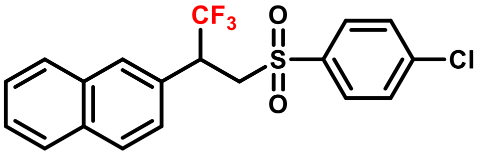

14

^{1}H NMR (400 MHz, $CDCl_3$) δ 7.82 – 7.76 (m, 1H), 7.67 (ddd, J = 19.1, 9.4, 6.0 Hz, 2H), 7.57 – 7.47 (m, 3H), 7.39 – 7.32 (m, 2H), 7.14 (d, J = 7.9 Hz, 1H), 7.00 – 6.89 (m, 2H), 4.20 – 4.05 (m, 1H), 3.86 (ddd, J = 17.9, 14.8, 6.9 Hz, 2H).
^{19}F NMR (376 MHz, $CDCl_3$) δ -69.75 (d, J = 8.9 Hz).
^{13}C NMR (101 MHz, $CDCl_3$) δ 140.35, 137.35, 133.18, 132.81, 129.69, 129.48, 129.19, 129.10, 128.95, 128.80, 127.71, 127.55, 127.09, 126.89, 125.51, 55.46, 46.24.
HRMS (ESI$^+$, *m/z*) [M+H$^+$] calcd. for $C_{19}H_{14}SO_2F_3Cl$ 399.0428, found 399.0427. [M+Na$^+$] 421.0247, found 421.0248.

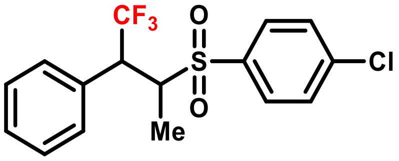

15

^{1}H NMR (400 MHz, $CDCl_3$) δ 7.35 – 7.30 (m, 2H), 7.18 (m, 6H), 4.19 (d, J = 9.8 Hz, 1H), 3.51 – 3.34 (m, 1H), 1.79 (d, J = 7.0 Hz, 3H).
^{19}F NMR (376 MHz, $CDCl_3$) δ -68.40 (d, J = 8.0 Hz).
^{13}C NMR (101 MHz, $CDCl_3$) δ 140.16, 136.74, 132.67, 130.08, 129.73, 129.02, 128.71, 128.61, 71.43, 40.82 (dd, J = 52.3, 26.1 Hz), 30.89, 12.84.
HRMS (ESI$^+$, *m/z*) [M+Na$^+$] calcd. for $C_{16}H_{14}SO_2F_3Cl$ 385.0247, found 385.0252.

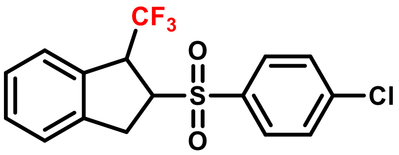

16

^{1}H NMR (400 MHz, $CDCl_3$) δ 7.84 – 7.80 (m, 2H), 7.54 – 7.48 (m, 2H), 7.35 – 7.17 (m, 4H), 4.32 (qd, *J* = 9.0, 2.4 Hz, 1H), 4.06 – 4.01 (m, 1H), 3.57 – 3.43 (m, 2H).
^{19}F NMR (376 MHz, $CDCl_3$) δ -70.74 (d, *J* = 9.1 Hz).
^{13}C NMR (101 MHz, $CDCl_3$) δ 141.19, 141.06, 135.24, 133.52, 130.29, 129.71, 129.56, 127.70, 125.84, 124.68, 63.55, 50.96 (q, *J* = 29.5 Hz), 33.20, 29.69.
HRMS (ESI^+, *m/z*) [$M+H^+$] calcd. for $C_{16}H_{12}SO_2F_3Cl$ 361.0271, found 361.0278.

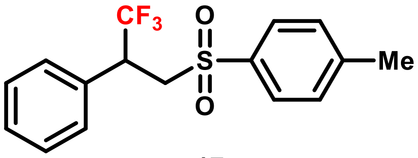

17

^{1}H NMR (400 MHz, $CDCl_3$) δ 7.53 – 7.43 (m, 2H), 7.29 – 7.18 (m, 3H), 7.13 (dd, *J* = 7.6, 4.2 Hz, 4H), 3.94 (pd, *J* = 9.1, 4.2 Hz, 1H), 3.80 – 3.65 (m, 2H), 2.37 (s, 3H).
^{19}F NMR (376 MHz, $CDCl_3$) δ -70.03 (d, *J* = 9.0 Hz).
^{13}C NMR (101 MHz, $CDCl_3$) δ 150.45, 144.76, 136.04, 131.31, 130.00, 129.70, 129.14, 128.71, 127.91, 55.48, 45.85, 21.53.

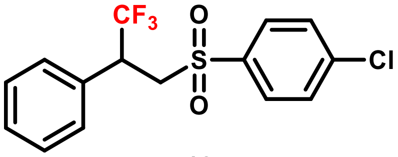

18

^{1}H NMR (400 MHz, $CDCl_3$) δ 7.52 – 7.42 (m, 2H), 7.32 – 7.24 (m, 3H), 7.20 (dd, *J* = 10.3, 4.8 Hz, 2H), 7.10 (d, *J* = 7.6 Hz, 2H), 3.96 (pd, *J* = 9.1, 4.5 Hz, 1H), 3.84 – 3.68 (m, 2H).
^{19}F NMR (376 MHz, $CDCl_3$) δ -70.05 (d, *J* = 9.1 Hz).
^{13}C NMR (101 MHz, $CDCl_3$) δ 140.50, 137.44, 130.81, 129.31, 129.16, 128.97, 128.85, 126.69, 123.90, 55.50, 45.98 (q, *J* = 28.6 Hz).
HRMS (ESI^+, *m/z*) [$M+Na^+$] calcd. for $C_{15}H_{12}SO_2F_3Cl$ 371.0091, found 371.0097.

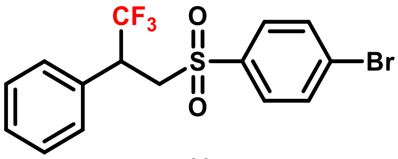

19

^{1}H NMR (400 MHz, $CDCl_3$) δ 7.46 – 7.36 (m, 4H), 7.31 – 7.26 (m, 1H), 7.23 – 7.17 (m, 2H), 7.09 (d, *J* = 7.6 Hz, 2H), 3.95 (pt, *J* = 8.0, 3.9 Hz, 1H), 3.82 – 3.68 (m, 2H).

^{19}F NMR (376 MHz, $CDCl_3$) δ -70.05 (d, *J* = 9.0 Hz).

^{13}C NMR (101 MHz, $CDCl_3$) δ 137.95, 132.32, 130.78, 129.34, 129.16, 129.10, 128.95, 128.87, 126.68, 55.49, 45.97 (q, *J* = 28.7 Hz).

HRMS (ESI$^+$, *m/z*) [M+H$^+$] calcd. for $C_{15}H_{12}SO_2F_3Br$ 392.9766, found 392.9759. [M+Na$^+$] 414.9586, found 414.9589.

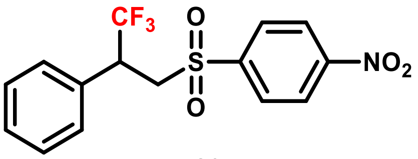

20

^{1}H NMR (400 MHz, $CDCl_3$) δ 8.12 – 8.07 (m, 2H), 7.71 – 7.66 (m, 2H), 7.24 (dt, *J* = 2.6, 1.7 Hz, 1H), 7.18 – 7.13 (m, 2H), 7.08 (d, *J* = 7.6 Hz, 2H), 4.07 – 3.94 (m, 1H), 3.90 – 3.77 (m, 2H).

^{19}F NMR (376 MHz, $CDCl_3$) δ -70.02 (d, *J* = 8.8 Hz).

^{13}C NMR (101 MHz, $CDCl_3$) δ 150.47, 144.52, 130.43, 130.41, 129.31, 129.26, 128.92, 126.52, 123.98, 55.56, 46.05 (q, *J* = 29.0 Hz).

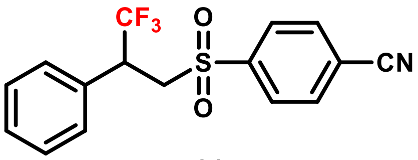

21

^{1}H NMR (400 MHz, $CDCl_3$) δ 7.65 – 7.52 (m, 4H), 7.30 – 7.23 (m, 1H), 7.21 – 7.14 (m, 2H), 7.07 (d, *J* = 7.6 Hz, 2H), 3.99 (pd, *J* = 8.9, 4.8 Hz, 1H), 3.89 – 3.75 (m, 2H).

^{19}F NMR (376 MHz, $CDCl_3$) δ -70.03 (d, *J* = 8.8 Hz).

^{13}C NMR (101 MHz, $CDCl_3$) δ 143.10, 132.59, 130.44, 129.24, 129.21, 128.93, 128.53, 126.54, 123.75, 117.30, 116.84, 55.45, 46.02 (q, *J* = 28.9 Hz).

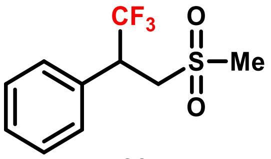

22

^{1}H NMR (400 MHz, $CDCl_3$) δ 7.47 – 7.38 (m, 5H), 4.13 – 3.93 (m, 1H), 3.69 – 3.53 (m, 2H), 2.36 (s, 3H).

^{19}F NMR (376 MHz, $CDCl_3$) δ -69.61 (d, *J* = 9.0 Hz).

^{13}C NMR (101 MHz, $CDCl_3$) δ 129.62, 129.49, 129.30, 129.20, 127.14, 54.94, 46.13 (d, *J* = 28.9 Hz), 42.27.

HRMS (ESI$^+$, *m/z*) [M+Na$^+$] calcd. for $C_{10}H_{11}SO_2F_3$ 275.0324, found 275.0326.

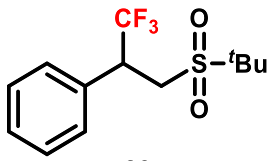

23

^{1}H NMR (400 MHz, $CDCl_3$) δ 7.43 – 7.34 (m, 5H), 4.12 (pd, *J* = 9.4, 3.1 Hz, 1H), 3.51 (qd, *J* = 13.6, 6.4 Hz, 2H), 1.38 (s, 9H).

^{19}F NMR (376 MHz, $CDCl_3$) δ -69.53 (d, *J* = 9.3 Hz).

^{13}C NMR (101 MHz, $CDCl_3$) δ 132.64, 129.08, 129.00, 128.91, 124.49, 60.03, 45.61, 44.14, 23.24.

HRMS (ESI$^+$, *m/z*) [M+Na$^+$] calcd. for $C_{13}H_{17}SO_2F_3$ 317.0794, found 317.0793.

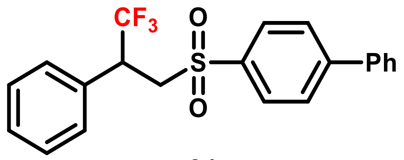

24

^{1}H NMR (400 MHz, $CDCl_3$) δ 7.66 – 7.62 (m, 2H), 7.58 – 7.45 (m, 7H), 7.27 – 7.13 (m, 5H), 4.02 (pd, *J* = 9.1, 3.4 Hz, 1H), 3.82 (qd, *J* = 14.7, 6.7 Hz, 2H).

^{19}F NMR (376 MHz, $CDCl_3$) δ -70.03 (d, *J* = 9.0 Hz).

^{13}C NMR (101 MHz, $CDCl_3$) δ 146.63, 139.09, 137.40, 131.09, 129.20, 129.11, 128.77, 128.75, 128.73, 128.38, 127.67, 127.31, 126.80, 55.48, 45.98 (q, *J* = 28.7 Hz).

HRMS (ESI$^+$, *m/z*) [M+H$^+$] calcd. for $C_{21}H_{17}SO_2F_3$ 391.0974, found 391.0980.

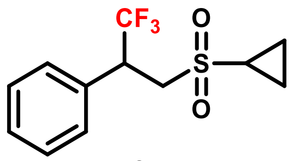

25

^{1}**H NMR** (400 MHz, $CDCl_3$) δ 7.45 – 7.36 (m, 5H), 4.12 – 3.95 (m, 1H), 3.65 (ddd, *J* = 17.6, 14.7, 6.8 Hz, 2H), 1.71 – 1.58 (m, 1H), 1.20 – 1.11 (m, 1H), 1.08 – 0.97 (m, 1H), 0.82 – 0.63 (m, 2H).

^{19}**F NMR** (376 MHz, $CDCl_3$) δ -69.77 (d, *J* = 9.0 Hz).

^{13}**C NMR** (101 MHz, $CDCl_3$) δ 132.16, 129.37, 129.32, 129.15, 126.95, 53.79, 45.79, 30.95, 5.51, 5.33.

HRMS (ESI$^+$, *m/z*) [M+H$^+$] calcd. for $C_{12}H_{13}SO_2F_3$ 301.0481, found 301.0485.

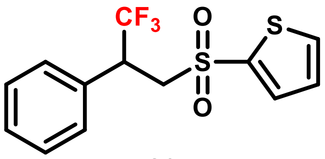

26

^{1}**H NMR** (400 MHz, $CDCl_3$) δ 7.57 (dd, *J* = 5.0, 1.3 Hz, 1H), 7.33 – 7.16 (m, 6H), 6.91 (dd, *J* = 4.9, 3.8 Hz, 1H), 4.08 – 3.93 (m, 1H), 3.93 – 3.79 (m, 2H).

^{19}**F NMR** (376 MHz, $CDCl_3$) δ -70.00 (d, *J* = 8.9 Hz).

^{13}**C NMR** (101 MHz, $CDCl_3$) δ 139.88, 134.71, 134.49, 131.22, 129.07, 128.97, 128.84, 127.78, 126.75, 56.8, 46.11 (q, *J* = 28.6 Hz).

HRMS (ESI$^+$, *m/z*) [M+H$^+$] calcd. for $C_{13}H_{11}S_2O_2F_3$ 321.0225, found 321.0231. [M+Na$^+$] 343.0045, found 343.0053.

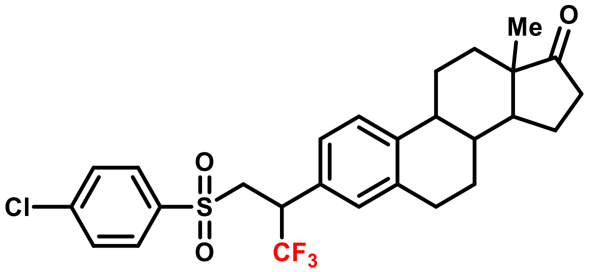

27

^{1}**H NMR** (400 MHz, $CDCl_3$) δ 7.44 – 7.37 (m, 2H), 7.23 – 7.18 (m, 2H), 7.09 (2d, *J* = 8.1 Hz, 1H), 6.88 (2d, *J* = 8.1 Hz, 1H), 6.62 (2s, 1H), 3.95 – 3.83 (m, 1H), 3.80 – 3.66 (m, 2H), 2.82 – 2.46 (m, 3H), 2.43 – 2.30 (m, 1H), 2.25 – 1.93 (m, 5H), 1.70 – 1.33 (m, 6H), 0.95 (2s, 3H).

^{19}**F NMR** (376 MHz, $CDCl_3$) δ -70.12 (d, *J* = 8.8 Hz), -70.17 (d, *J* = 8.8 Hz).

^{13}**C NMR** (101 MHz, $CDCl_3$) δ 220.38, 140.92/140.87, 139.96/139.90, 137.66/137.63, 136.91/136.89, 129.72, 129.31/129.29, 129.06, 128.86, 127.67/127.56, 127.21, 126.55, 125.83/125.81, 55.29/55.15, 53.40, 50.53/50.52, 47.94/47.89, 45.74 (q, *J* =

28.8 Hz), 44.30/44.24, 37.99/37.97, 35.80, 31.58/31.56, 29.20/29.18, 26.24, 25.74/25.58, 21.56, 14.01/13.90.

HRMS (ESI$^+$, *m/z*) [M+H$^+$] calcd. for $C_{27}H_{28}SO_4F_3Cl$ 525.1473, found 525.1482.

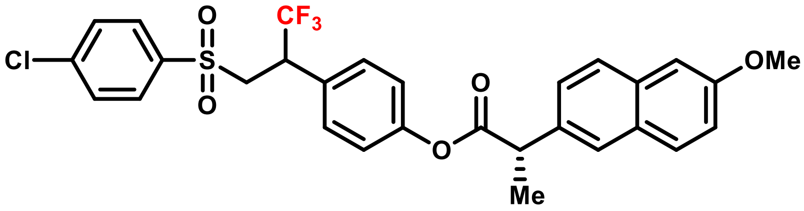

28

^{1}H NMR (400 MHz, $CDCl_3$) δ 7.81 – 7.69 (m, 3H), 7.49 (dt, *J* = 8.6, 1.6 Hz, 1H), 7.44 – 7.37 (m, 2H), 7.33 – 7.26 (m, 2H), 7.20 – 7.12 (m, 2H), 7.03 (d, *J* = 8.6 Hz, 2H), 6.87 – 6.79 (m, 2H), 4.16 – 4.04 (m, 1H), 4.00 – 3.86 (m, 4H), 3.76 – 3.61 (m, 2H), 1.71 (d, *J* = 7.1 Hz, 3H).

^{19}F NMR (376 MHz, $CDCl_3$) δ -70.13 (d, *J* = 8.8 Hz).

^{13}C NMR (101 MHz, $CDCl_3$) δ 172.59, 157.86, 151.40, 140.63, 137.34, 134.86, 134.81, 133.91, 130.09, 129.52, 129.32, 129.15, 129.02, 128.05, 127.43, 126.17, 126.06, 126.04, 121.87, 119.18, 105.68, 55.56, 55.35, 45.56, 18.36.

HRMS (ESI$^+$, *m/z*) [M+H$^+$] calcd. for $C_{29}H_{24}SO_5F_3Cl$ 577.1058, found 577.1053.

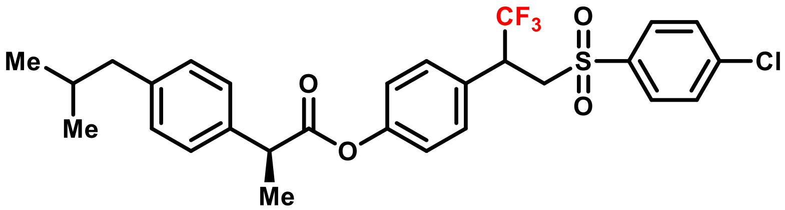

29

^{1}H NMR (400 MHz, $CDCl_3$) δ 7.48 – 7.38 (m, 2H), 7.30 (ddd, *J* = 6.9, 4.6, 1.7 Hz, 4H), 7.15 (d, *J* = 7.9 Hz, 2H), 7.04 (d, *J* = 8.5 Hz, 2H), 6.85 (d, *J* = 8.5 Hz, 2H), 4.02 – 3.87 (m, 2H), 3.76 – 3.65 (m, 2H), 2.48 (d, *J* = 7.2 Hz, 2H), 1.87 (dp, *J* = 13.5, 6.7 Hz, 1H), 1.62 (d, *J* = 7.2 Hz, 3H), 0.92 (d, *J* = 6.6 Hz, 6H).

^{19}F NMR (376 MHz, $CDCl_3$) δ -70.13 (dd, *J* = 8.9, 4.9 Hz).

^{13}C NMR (101 MHz, $CDCl_3$) δ 172.63, 151.44, 140.96, 140.64, 138.40, 137.37, 137.35, 136.97, 136.93, 130.08, 129.54, 129.15, 127.97, 127.20, 121.87, 55.59, 45.26, 45.06, 30.18, 22.38, 18.39.

HRMS (ESI$^+$, *m/z*) [M+H$^+$] calcd. for $C_{28}H_{28}SO_4F_3Cl$ 553.1422, found 553.1413.

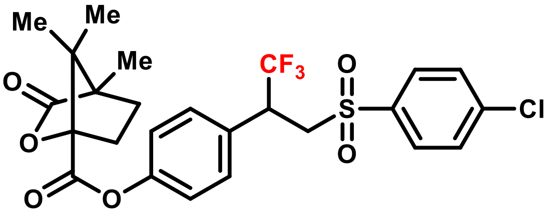

30

^{1}H NMR (400 MHz, $CDCl_3$) δ 7.47 (d, *J* = 8.7 Hz, 2H), 7.34 (dd, *J* = 8.5, 1.4 Hz, 2H), 7.14 (d, *J* = 8.5 Hz, 2H), 7.00 (d, *J* = 8.6 Hz, 2H), 4.07 – 3.92 (m, 1H), 3.75 (m, 2H), 2.63 – 2.50 (m, 1H), 2.20 (dddd, *J* = 13.6, 9.3, 4.5, 2.2 Hz, 1H), 2.06 – 1.94 (m, 1H), 1.78 (ddd, *J* = 13.4, 9.3, 4.2 Hz, 1H), 1.17 (d, *J* = 5.7 Hz, 6H), 1.11 (s, 3H).

^{19}F NMR (376 MHz, $CDCl_3$) δ -70.06 (d, *J* = 8.8 Hz).

^{13}C NMR (101 MHz, $CDCl_3$) δ 177.68, 165.67/165.64, 150.46, 140.65, 137.43, 137.40, 130.41, 129.58, 129.21, 128.93, 121.79, 90.70, 55.50, 54.92, 54.72, 45.89, 45.60, 45.31, 45.02, 30.82/30.78, 28.96, 16.89/16.86, 9.72.

HRMS (ESI^+, *m/z*) [$M+H^+$] calcd. for $C_{25}H_{24}SO_6F_3Cl$ 545.1007, found 545.1004.

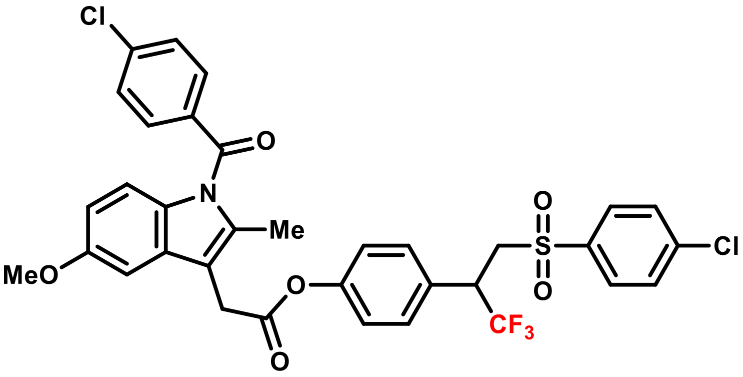

31

^{1}H NMR (400 MHz, $CDCl_3$) δ 7.73 – 7.65 (m, 2H), 7.51 – 7.41 (m, 4H), 7.33 – 7.28 (m, 2H), 7.07 (dd, *J* = 12.2, 5.5 Hz, 3H), 6.96 – 6.84 (m, 3H), 6.71 (dd, *J* = 9.0, 2.5 Hz, 1H), 3.96 (ddd, *J* = 15.8, 7.8, 4.5 Hz, 1H), 3.91 (s, 2H), 3.85 (s, 3H), 3.71 (m, 2H), 2.47 (s, 3H).

^{19}F NMR (376 MHz, $CDCl_3$) δ -70.08 (d, *J* = 8.9 Hz).

^{13}C NMR (101 MHz, $CDCl_3$) δ 168.67, 168.30, 156.19, 151.22, 140.64, 139.44, 137.38, 136.35, 133.81, 131.21, 130.91, 130.46, 130.20, 129.55, 129.19, 128.37, 121.89, 115.04, 111.72, 101.39, 55.78, 55.55, 45.40 (d, *J* = 29.0 Hz), 30.52, 13.38.

HRMS (ESI^+, *m/z*) [$M+H^+$] calcd. for $C_{34}H_{26}NSO_6F_3Cl_2$ 704.0883, found 704.0882.

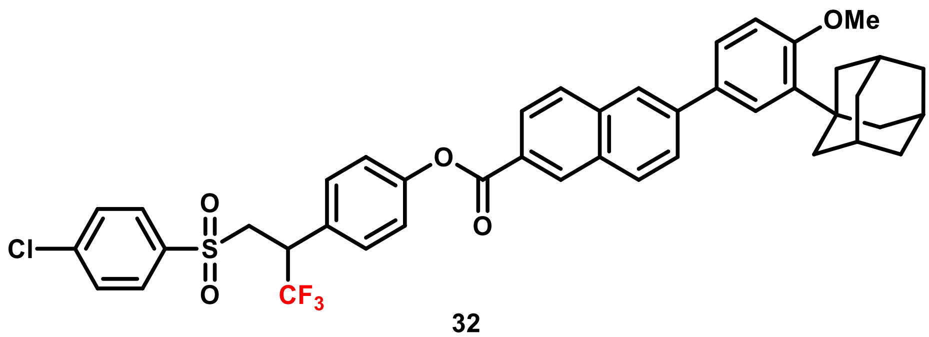

32

^{1}H NMR (400 MHz, $CDCl_3$) δ 8.79 (d, *J* = 10.8 Hz, 1H), 8.19 (dd, *J* = 8.6, 1.7 Hz, 1H), 8.02 (dd, *J* = 20.9, 9.2 Hz, 3H), 7.85 (dd, *J* = 8.5, 1.7 Hz, 1H), 7.63 (d, *J* = 2.3 Hz, 1H), 7.57 (dd, *J* = 8.4, 2.3 Hz, 1H), 7.53 – 7.49 (m, 2H), 7.43 – 7.38 (m, 2H), 7.21 – 7.13 (m, 4H), 7.02 (d, *J* = 8.5 Hz, 1H), 4.10 – 3.98 (m, 1H), 3.92 (s, 3H), 3.79 (d, *J* = 6.9 Hz, 2H), 2.20 (d, *J* = 2.4 Hz, 6H), 2.12 (s, 3H), 1.82 (s, 6H).

^{19}F NMR (376 MHz, $CDCl_3$) δ -70.01 (d, *J* = 8.9 Hz).

^{13}C NMR (101 MHz, $CDCl_3$) δ 164.80, 159.09, 152.14, 151.71, 141.99, 140.74, 139.13, 137.45, 136.39, 132.40, 131.83, 131.24, 130.31, 129.86, 129.63, 129.27, 128.57, 126.78, 126.01, 125.86, 125.78, 125.71, 124.78, 122.31, 112.19, 55.19, 40.66, 37.26, 37.15, 29.70, 29.14.

HRMS (ESI^+, *m/z*) [$M+Na^+$] calcd. for $C_{43}H_{38}SO_5F_3Cl$ 781.1973, found 781.1963.

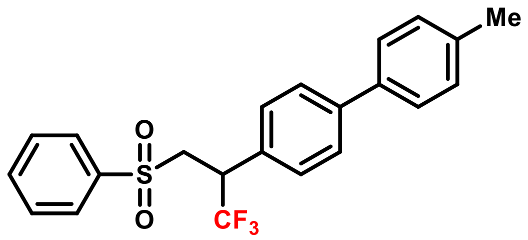

33

^{1}H NMR (400 MHz, $CDCl_3$) δ 7.60 – 7.56 (m, 2H), 7.49 – 7.44 (m, 1H), 7.42 – 7.38 (m, 2H), 7.37 – 7.32 (m, 2H), 7.29 (m, 4H), 7.14 (d, *J* = 8.2 Hz, 2H), 4.07 – 3.96 (m, 1H), 3.80 (qd, *J* = 14.7, 6.8 Hz, 2H), 2.41 (s, 3H).

^{19}F NMR (376 MHz, $CDCl_3$) δ -69.98 (d, *J* = 9.0 Hz).

^{13}C NMR (101 MHz, $CDCl_3$) δ 141.50, 140.23, 139.12, 133.43, 129.60, 129.02, 127.88, 127.19, 126.84, 106.88, 97.48, 92.65, 90.67, 55.07, 45.05, 21.11.

TLC-MS (ESI$^+$, *m/z*) [M+Na$^+$] calcd. for $C_{22}H_{19}SO_2F_3$ 427.4370, found 427.4378.

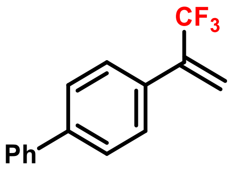

34

^{1}H NMR (400 MHz, $CDCl_3$) δ 7.61 – 7.55 (m, 4H), 7.52 (d, *J* = 8.2 Hz, 2H), 7.43 (t, *J* = 7.5 Hz, 2H), 7.34 (t, *J* = 7.3 Hz, 1H), 5.95 (d, *J* = 1.1 Hz, 1H), 5.79 (d, *J* = 1.6 Hz, 1H).
^{19}F NMR (376 MHz, $CDCl_3$) δ -64.78.
^{13}C NMR (101 MHz, $CDCl_3$) δ 141.90, 140.30, 138.67 (q, *J* = 30.0 Hz), 132.51, 128.91, 127.78, 127.73, 127.30, 127.11, 123.46 (q, *J* = 274.1 Hz), 120.20 (q, *J* = 5.8 Hz).
GCMS (EI, *m/z*) [M$^+$] calcd. for $C_{15}H_{11}F_3$ 248.0807, found 248.0804.

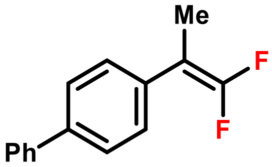

35

^{1}H NMR (400 MHz, $CDCl_3$) δ 7.64 – 7.57 (m, 4H), 7.48 – 7.40 (m, 4H), 7.40 – 7.30 (m, 1H), 2.02 (t, *J* = 3.4 Hz, 3H).
^{19}F NMR (376 MHz, $CDCl_3$) δ -89.82 (dq, *J* = 42.9, 3.3 Hz), 90.20 (dq, *J* = 42.8, 3.3 Hz).
^{13}C NMR (101 MHz, $CDCl_3$) δ 153.59 (dd, *J* = 290.7, 286.3 Hz), 140.60, 139.88, 133.84 (t, *J* = 4.2 Hz), 128.81, 127.83 (dd, *J* = 4.7, 3.4 Hz), 127.38, 127.04, 127.01, 87.23 (dd, *J* = 22.6, 14.1 Hz), 13.15 (t, *J* = 1.8 Hz).
GCMS (EI, *m/z*) [M$^+$] calcd. for $C_{15}H_{12}F_2$ 230.0902, found 230.0899.

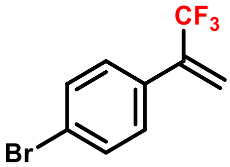

36

^{1}H NMR (400 MHz, $CDCl_3$) δ 7.56 – 7.50 (m, 2H), 7.32 (d, *J* = 8.4 Hz, 2H), 5.98 (q, *J* = 1.3 Hz, 1H), 5.78 (q, *J* = 1.6 Hz, 1H).
^{19}F NMR (376 MHz, $CDCl_3$) δ -64.95 (s).
^{13}C NMR (101 MHz, $CDCl_3$) δ 138.28 (q, *J* = 30.5 Hz), 132.67, 131.95, 129.16, 123.54, 122.04 (q, *J* = 273.9 Hz), 121.03 (q, *J* = 5.7 Hz).

GCMS (EI, *m/z*) [M^+] calcd. for $C_9H_6F_3Br$ 251.9579, found 251.9577.

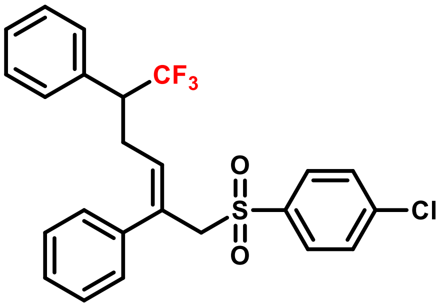

41

^{1}H NMR (400 MHz, $CDCl_3$) δ 7.59 (dd, *J* = 8.9, 2.1 Hz, 2H), 7.37 (m, 4H), 7.31 – 7.27 (m, 4H), 7.15 – 7.11 (m, 2H), 6.95 – 6.88 (m, 2H), 5.78 (t, *J* = 7.4 Hz, 1H), 4.26 (s, 2H), 3.49 – 3.37 (m, 1H), 2.98 – 2.88 (m, 1H), 2.84 – 2.73 (m, 1H).
^{19}F NMR (376 MHz, $CDCl_3$) δ -69.30 (d, *J* = 9.2 Hz).
^{13}C NMR (101 MHz, $CDCl_3$) δ 140.48, 140.17, 137.35, 133.53, 130.29, 129.85, 129.18, 128.98, 128.92, 128.86, 128.48, 128.36, 128.20, 127.55, 126.37, 57.90, 49.77, 29.23.
HRMS (ESI$^+$, *m/z*) [$M+H^+$] calcd. for $C_{24}H_{20}SO_2F_3Cl$ 465.0897, found 465.0911.

1.7 Spectra

^{1}H NMR (400 MHz, $CDCl_3$) of TT-CF_3^+OTF$^-$

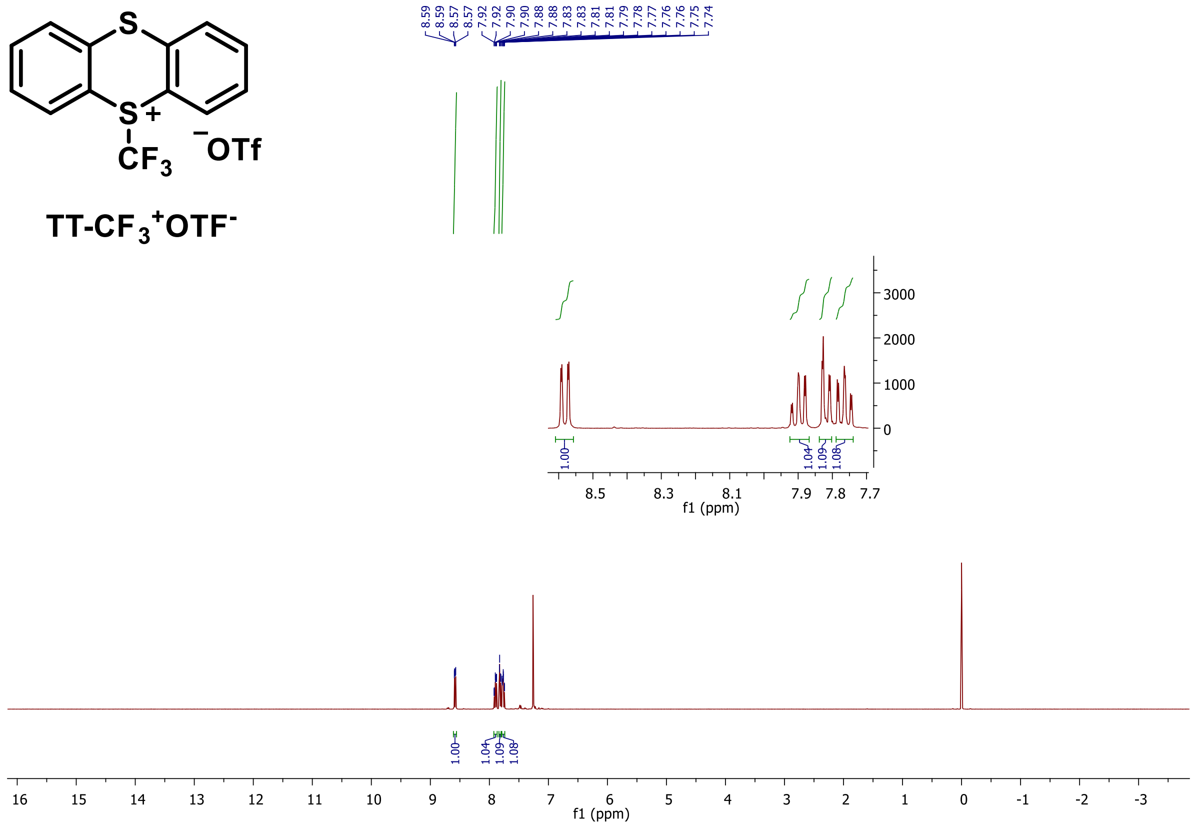

^{19}F NMR (376 MHz, $CDCl_3$) of TT-$CF_3$$^+OTF^-$

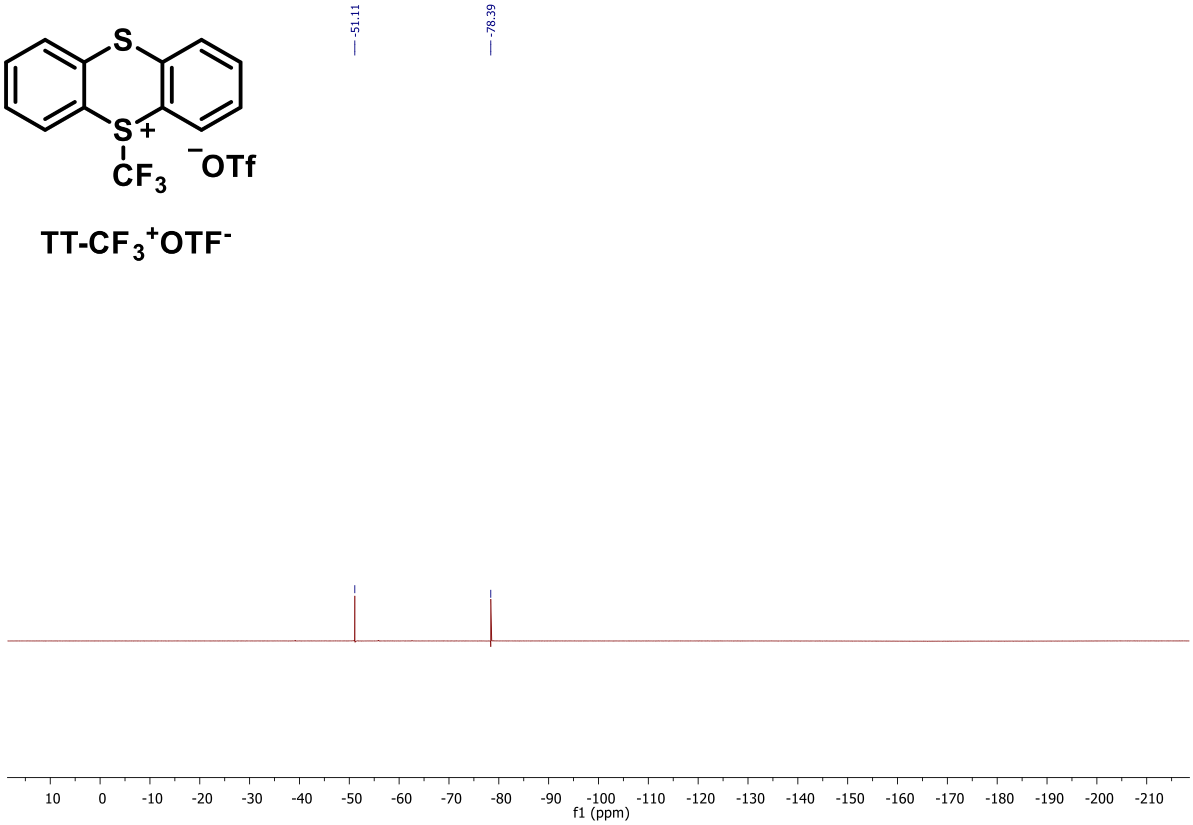

^{13}C NMR (101 MHz, $CDCl_3$) of TT-$CF_3$$^+OTF^-$

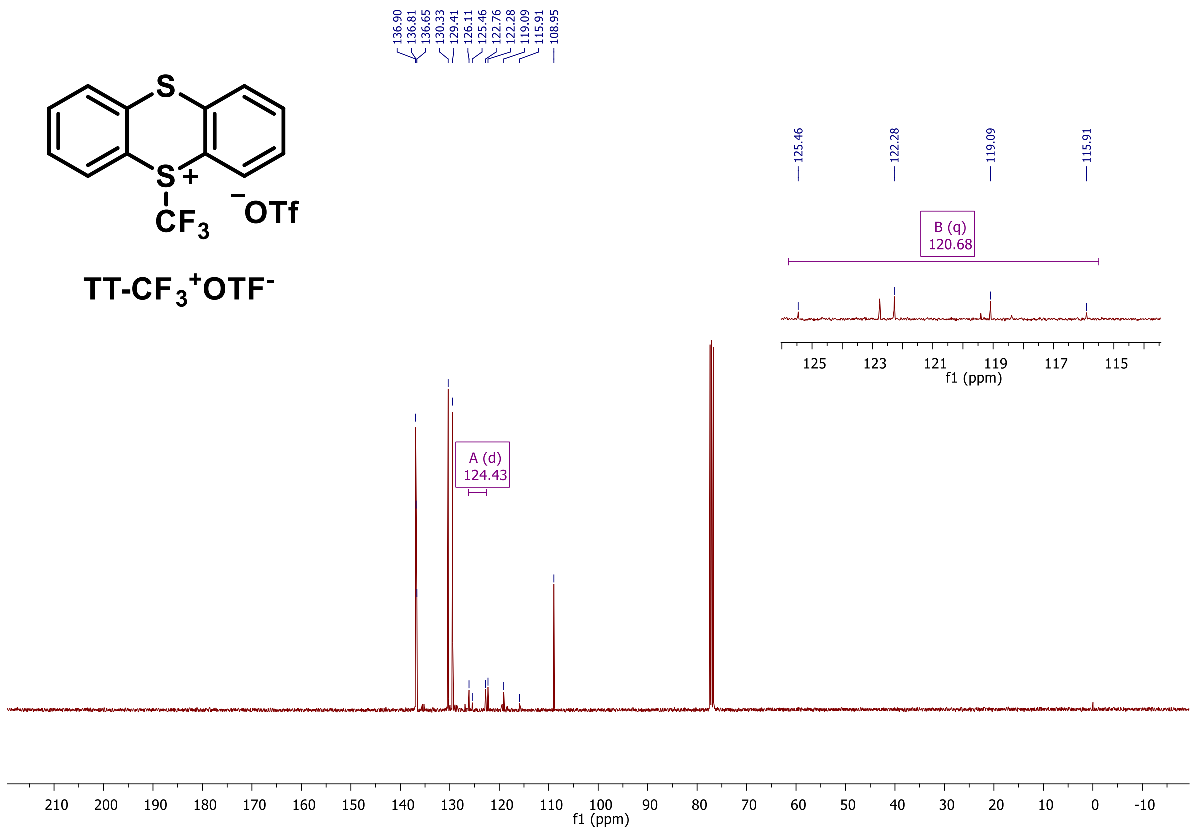

^{1}H NMR (400 MHz, $CDCl_3$) of $Os(bptpy)_2(PF_6)_2$

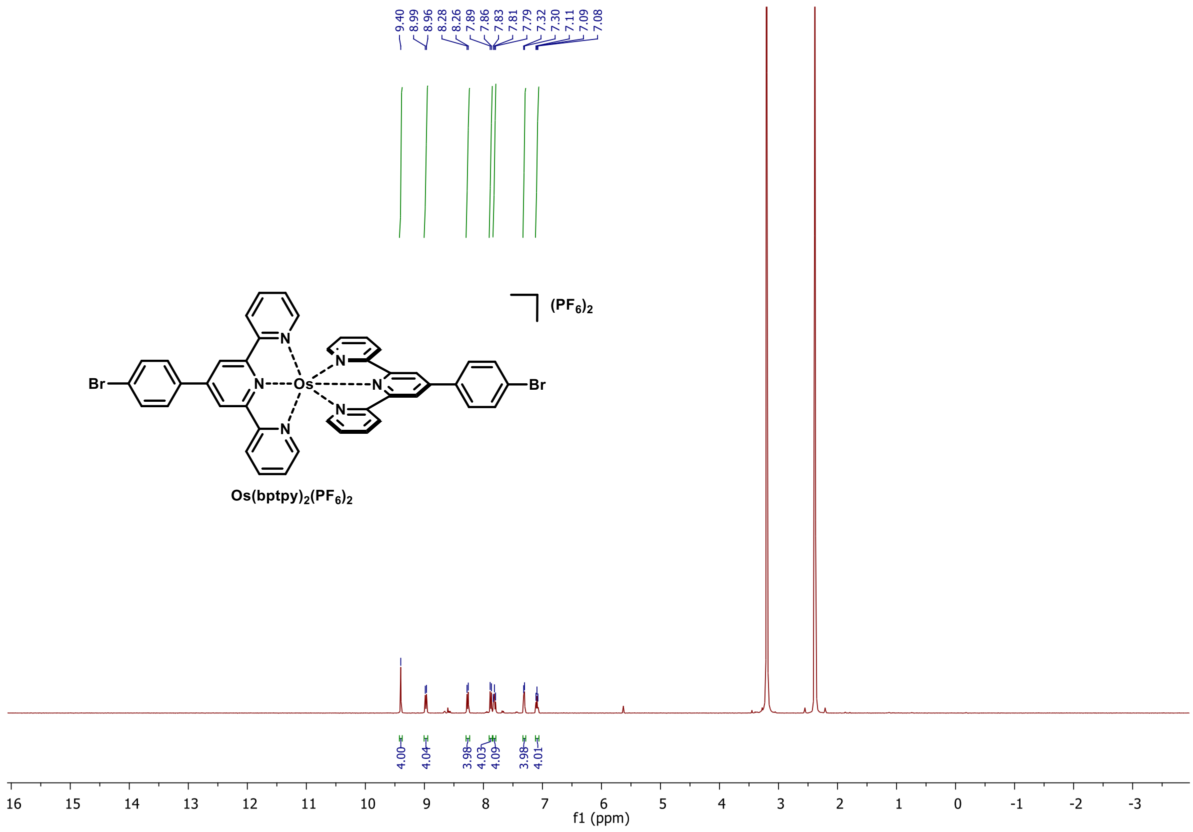

^{19}F NMR (376 MHz, $CDCl_3$) of $Os(bptpy)_2(PF_6)_2$

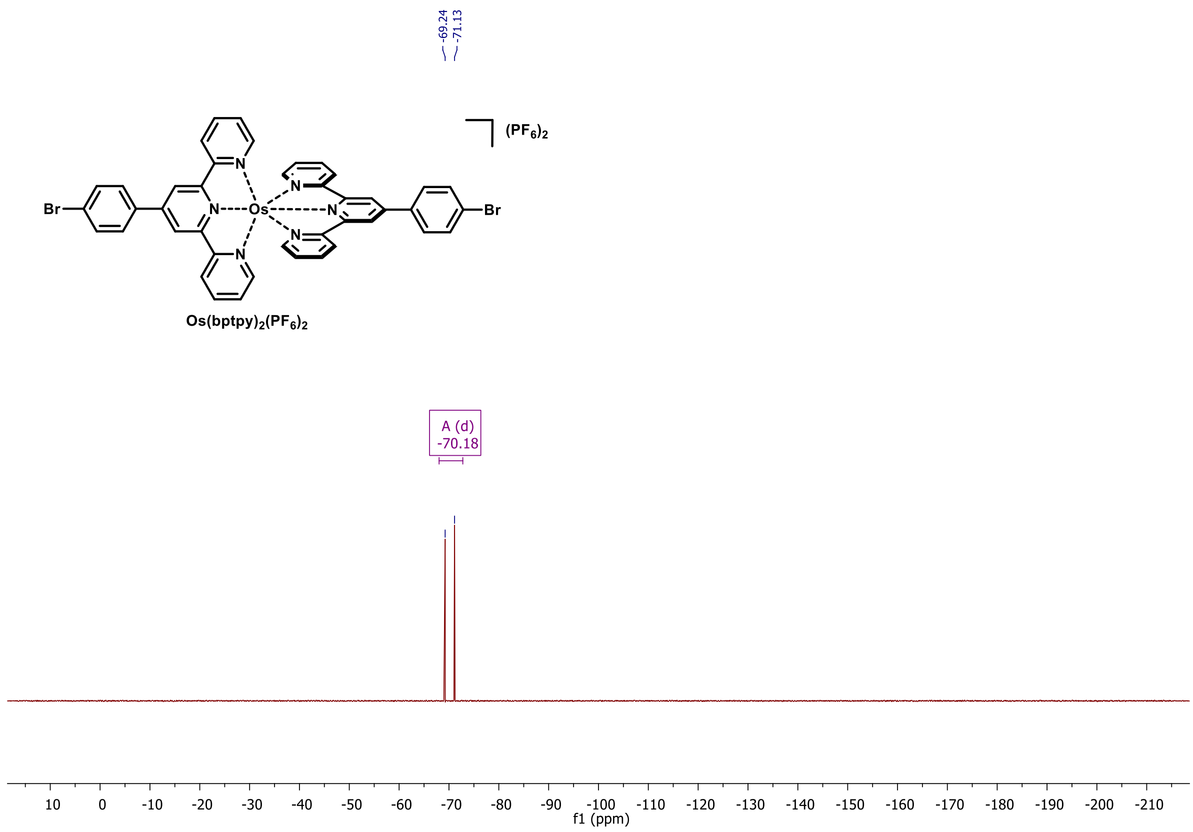

^{13}**C NMR** (101 MHz, $CDCl_3$) of $Os(bptpy)_2(PF_6)_2$

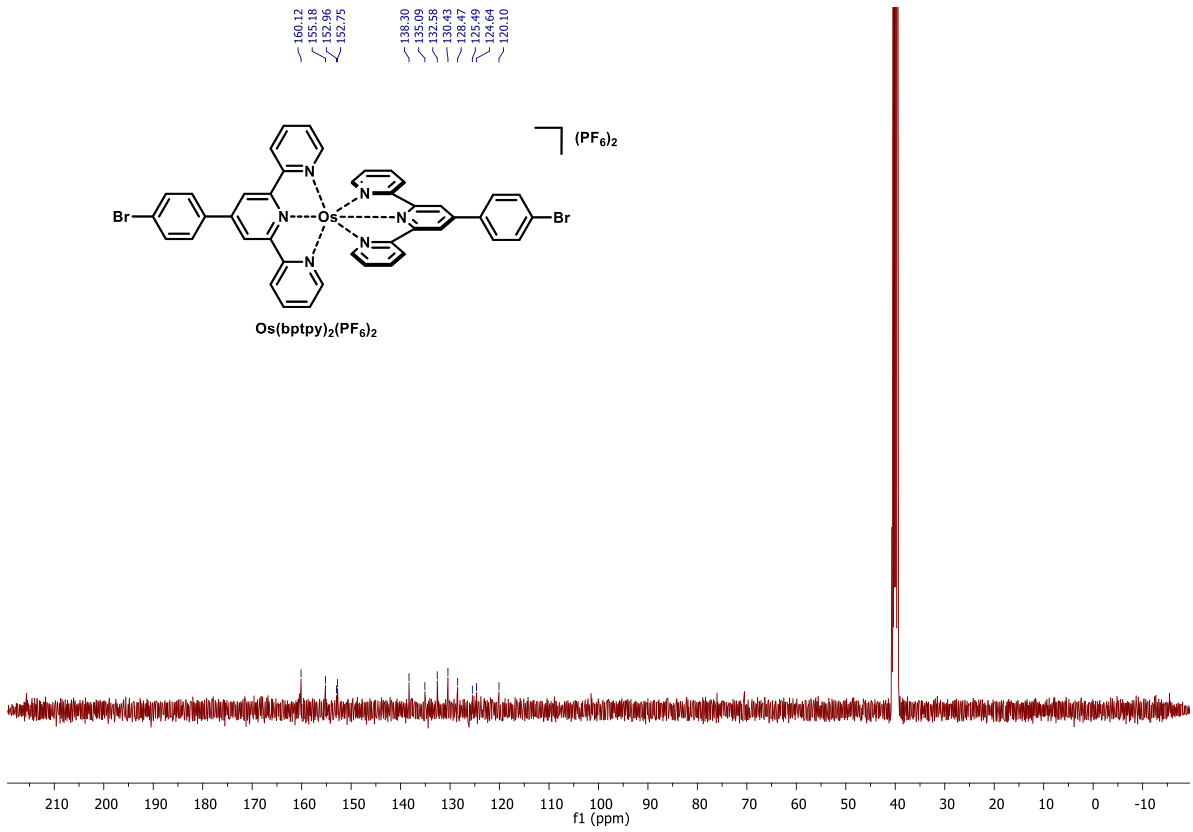

^{1}**H NMR** (400 MHz, $CDCl_3$) of **S1**

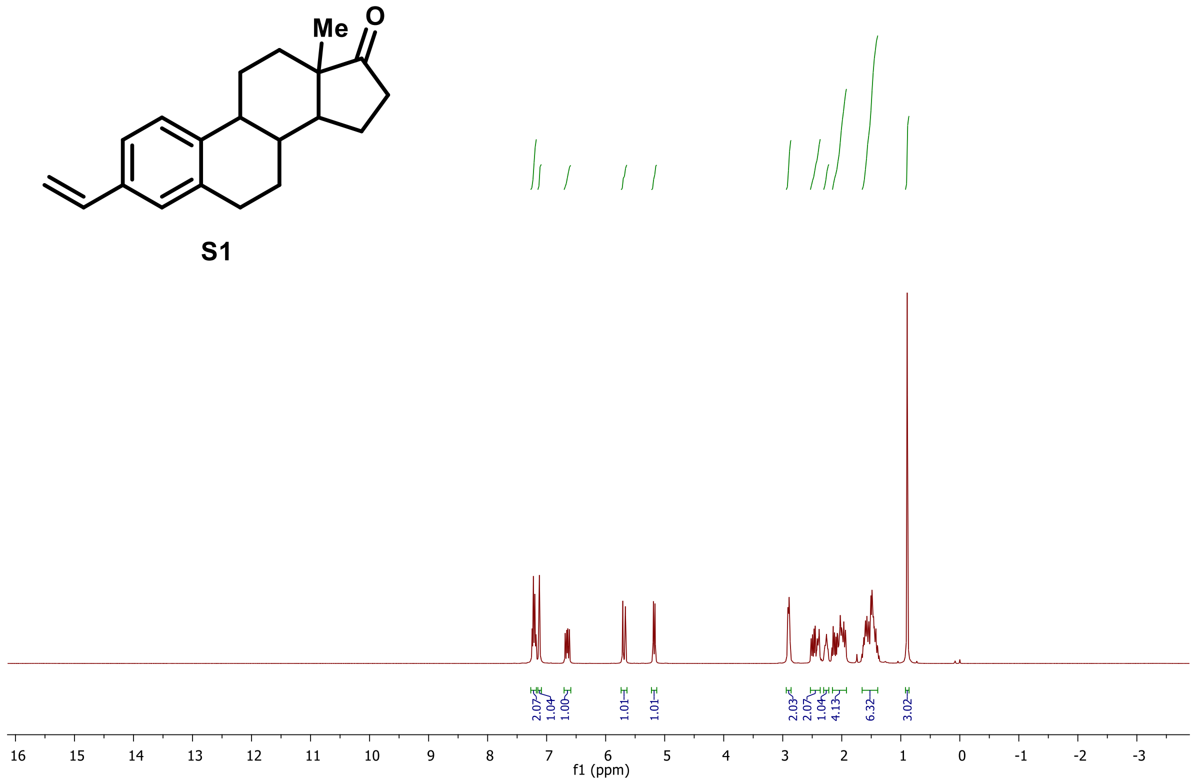

^{13}C NMR (101 MHz, $CDCl_3$) of **S1**

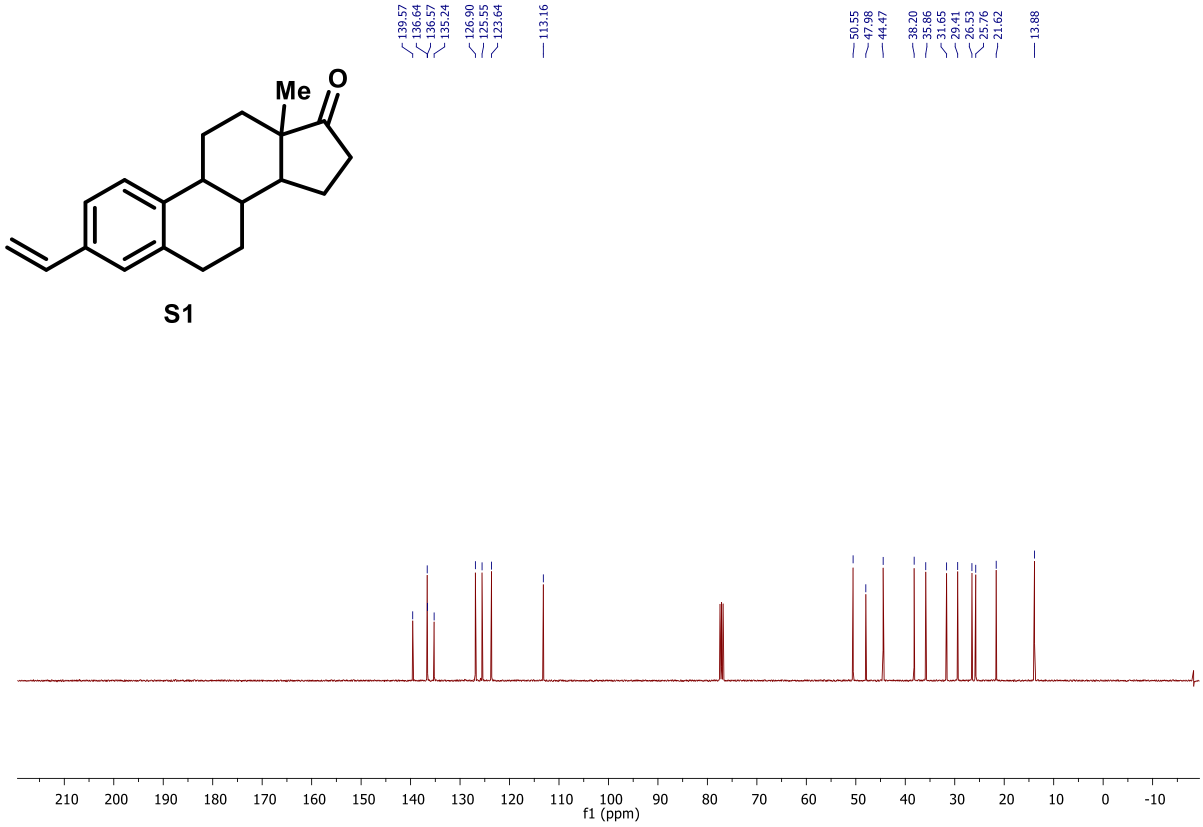

^{1}H NMR (400 MHz, $CDCl_3$) of **S2**

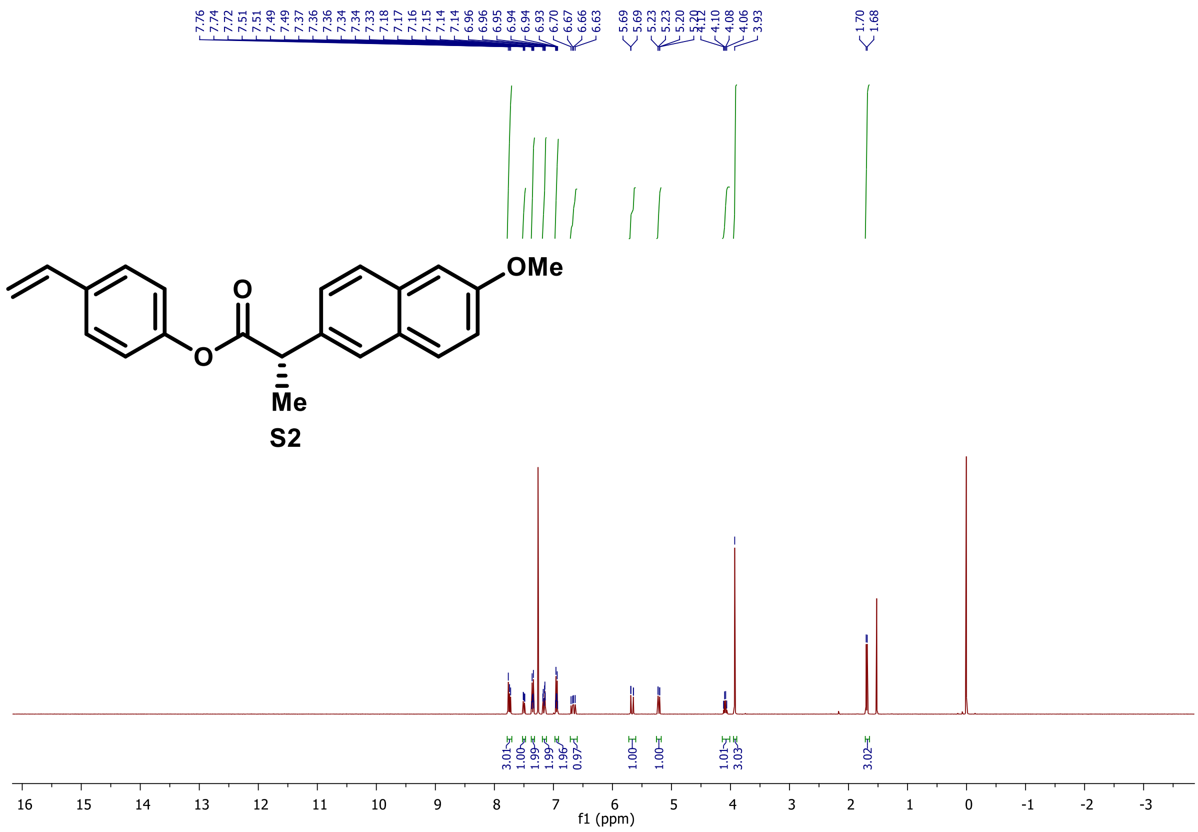

^{13}C NMR (101 MHz, $CDCl_3$) of **S2**

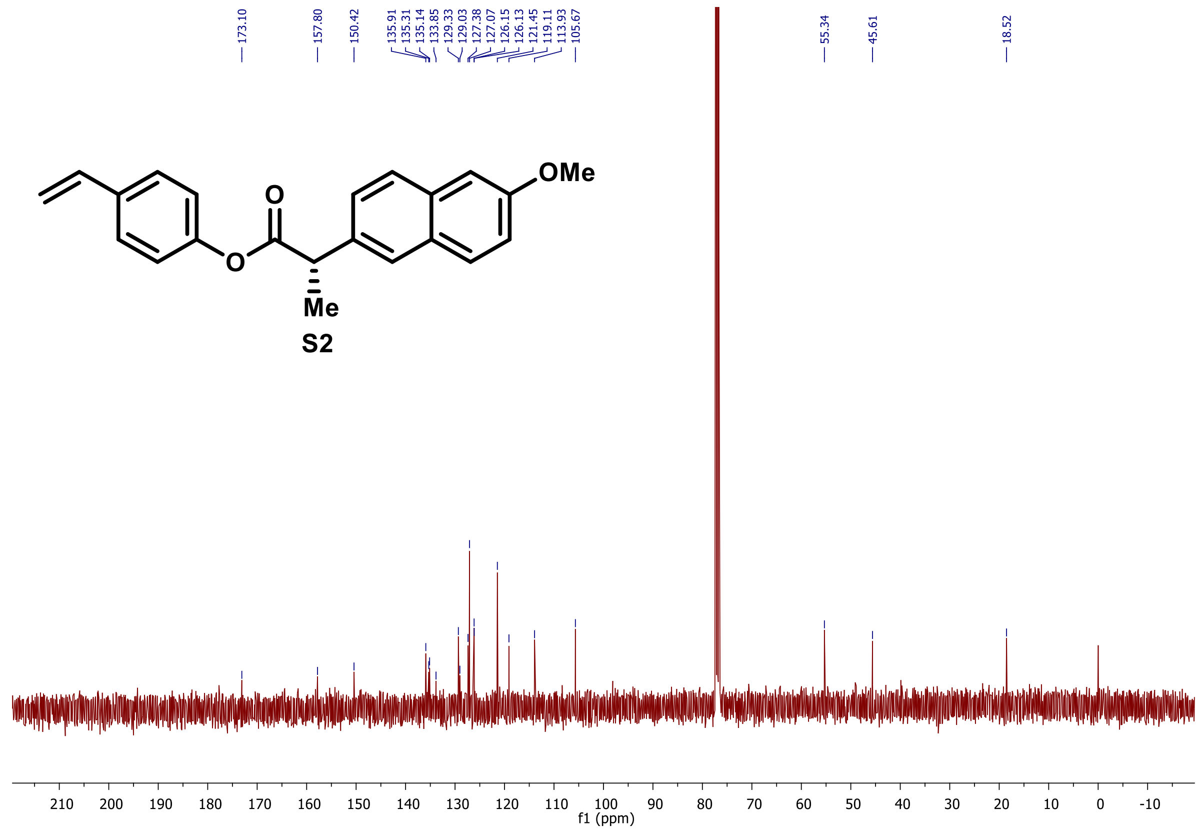

^{1}H NMR (400 MHz, $CDCl_3$) of **S3**

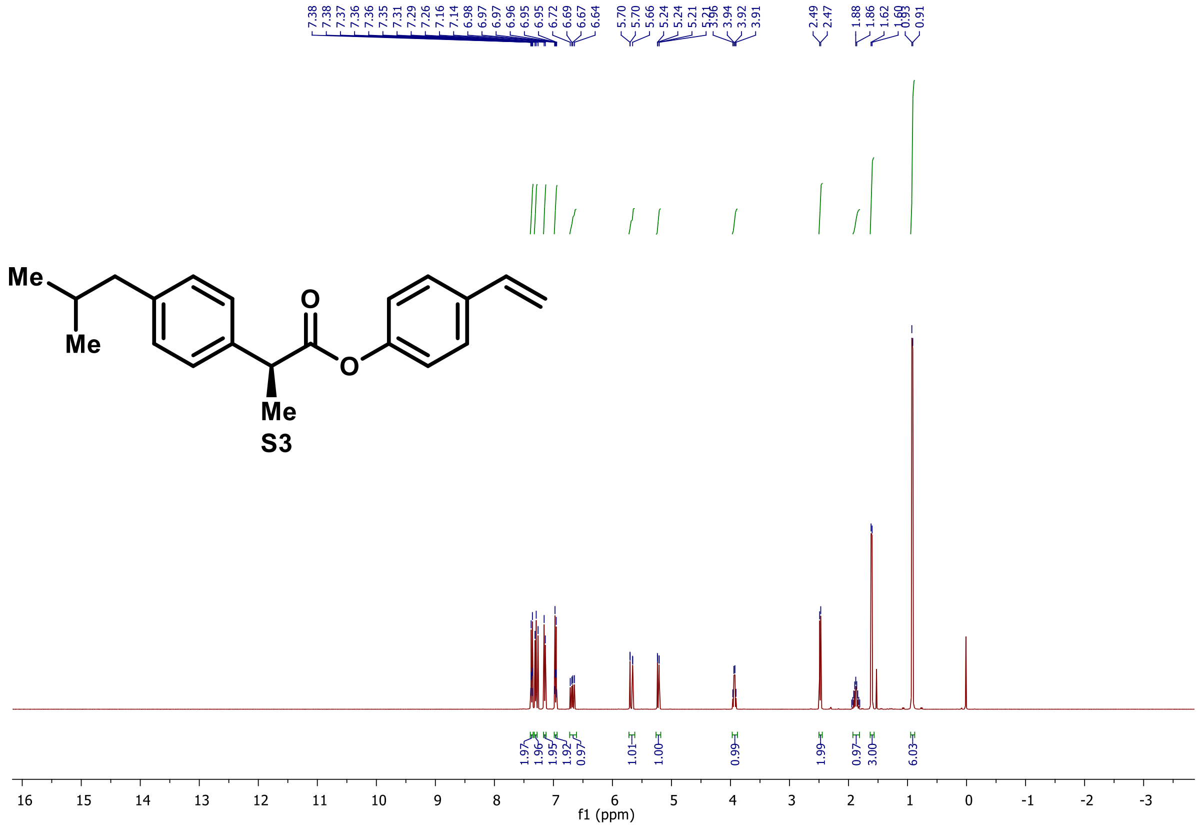

^{13}C NMR (101 MHz, $CDCl_3$) of **S3**

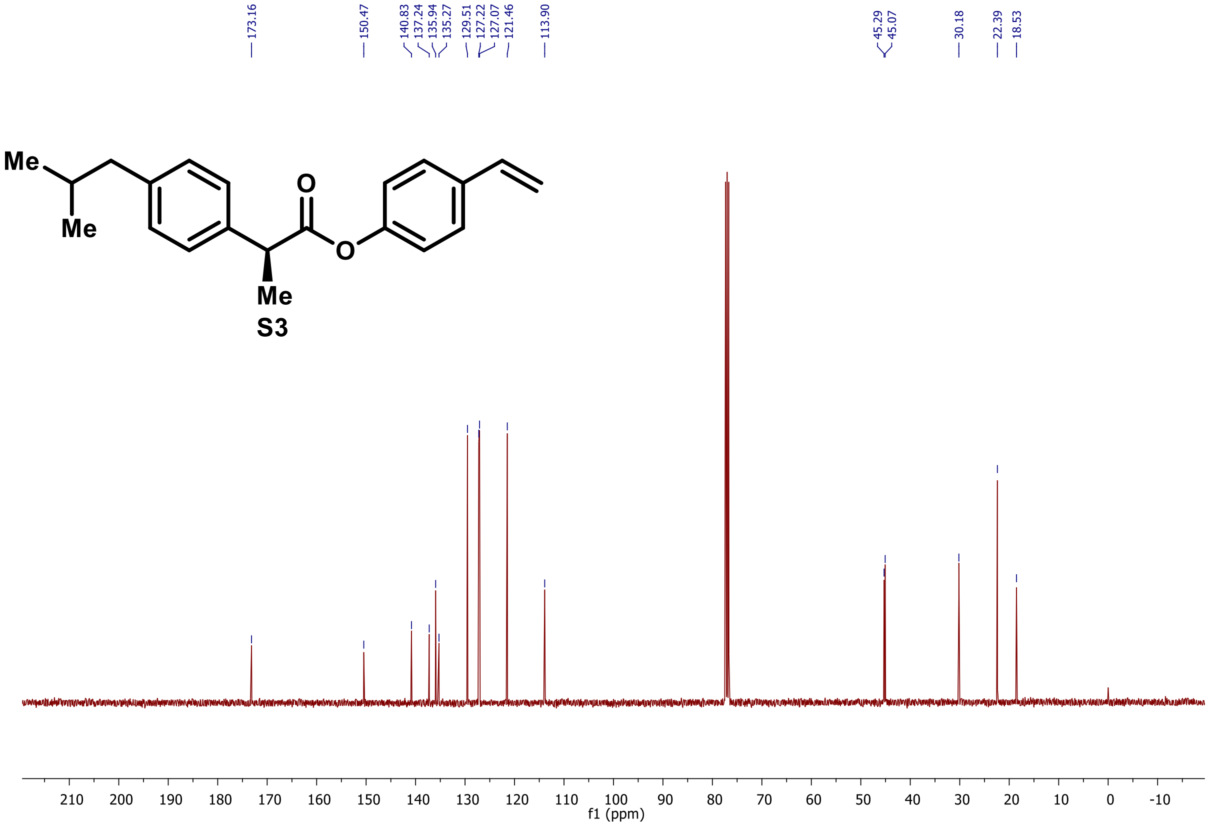

^{1}H NMR (400 MHz, $CDCl_3$) of **S4**

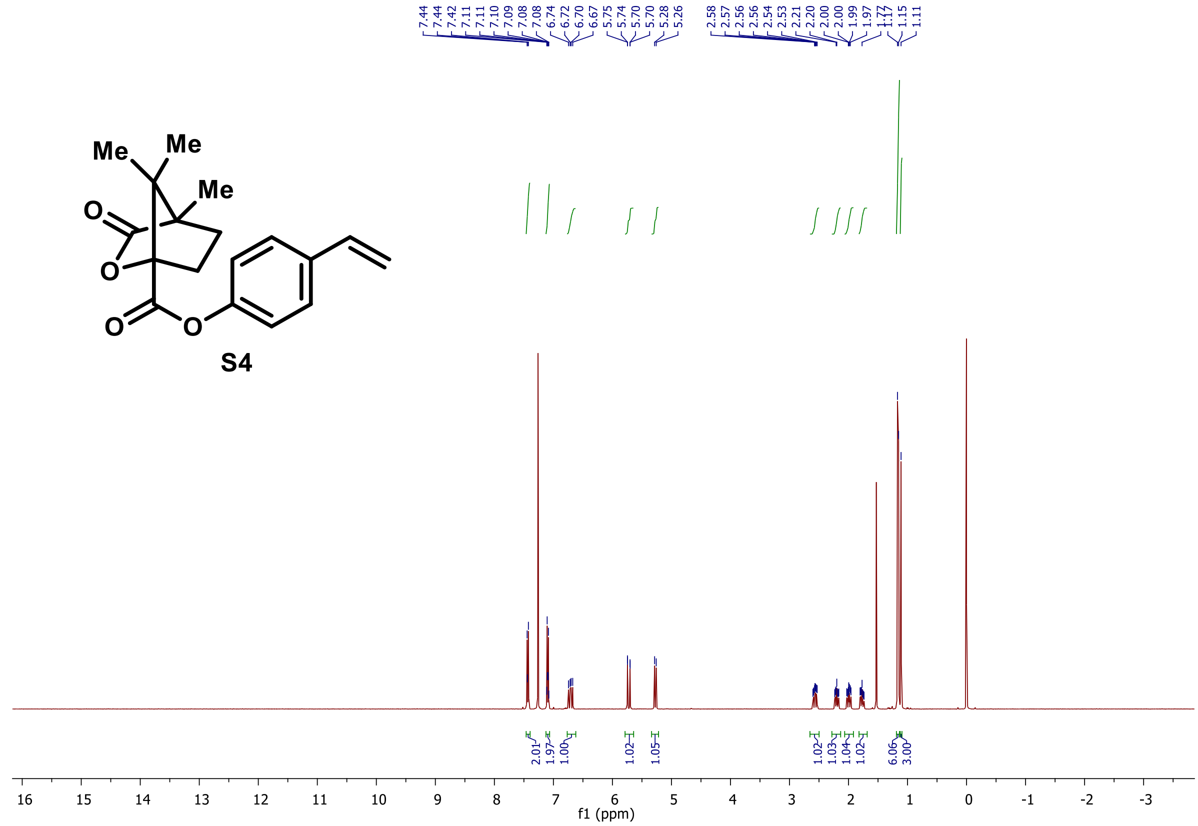

^{13}C NMR (101 MHz, $CDCl_3$) of **S4**

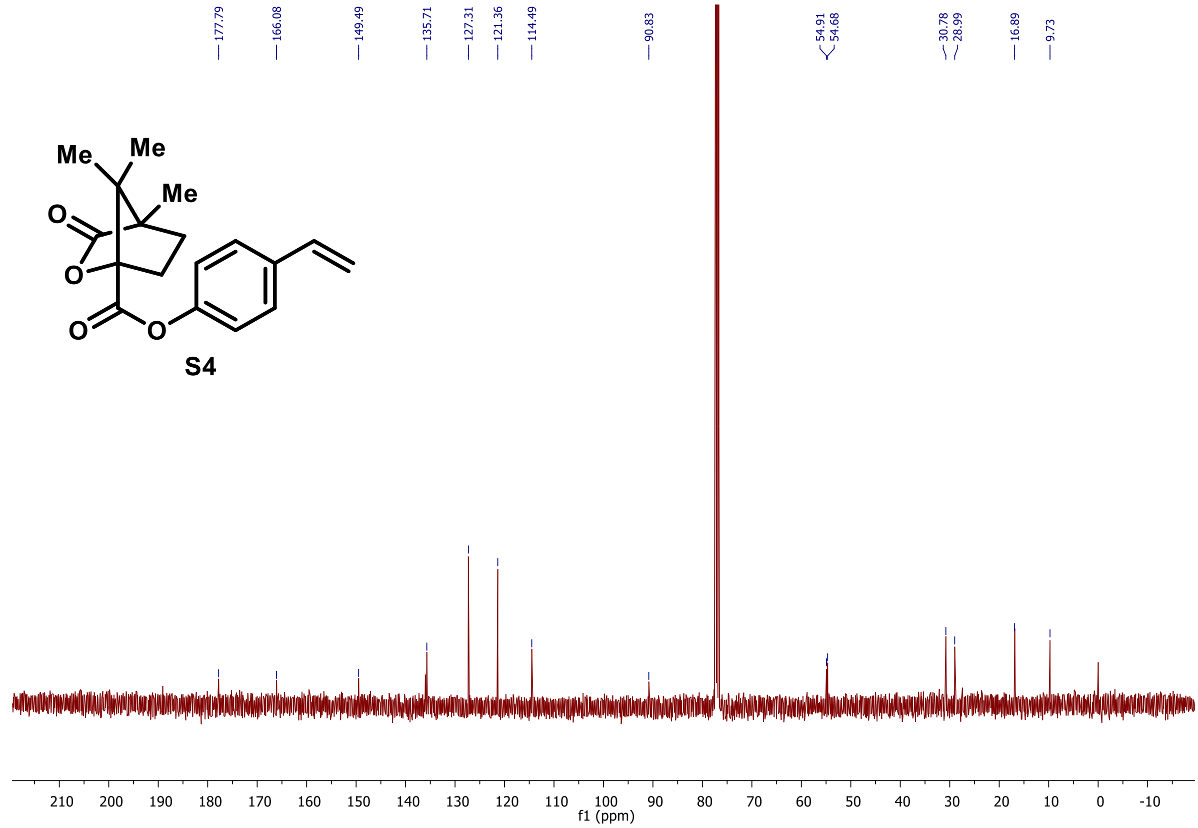

^{1}H NMR (400 MHz, $CDCl_3$) of **S5**

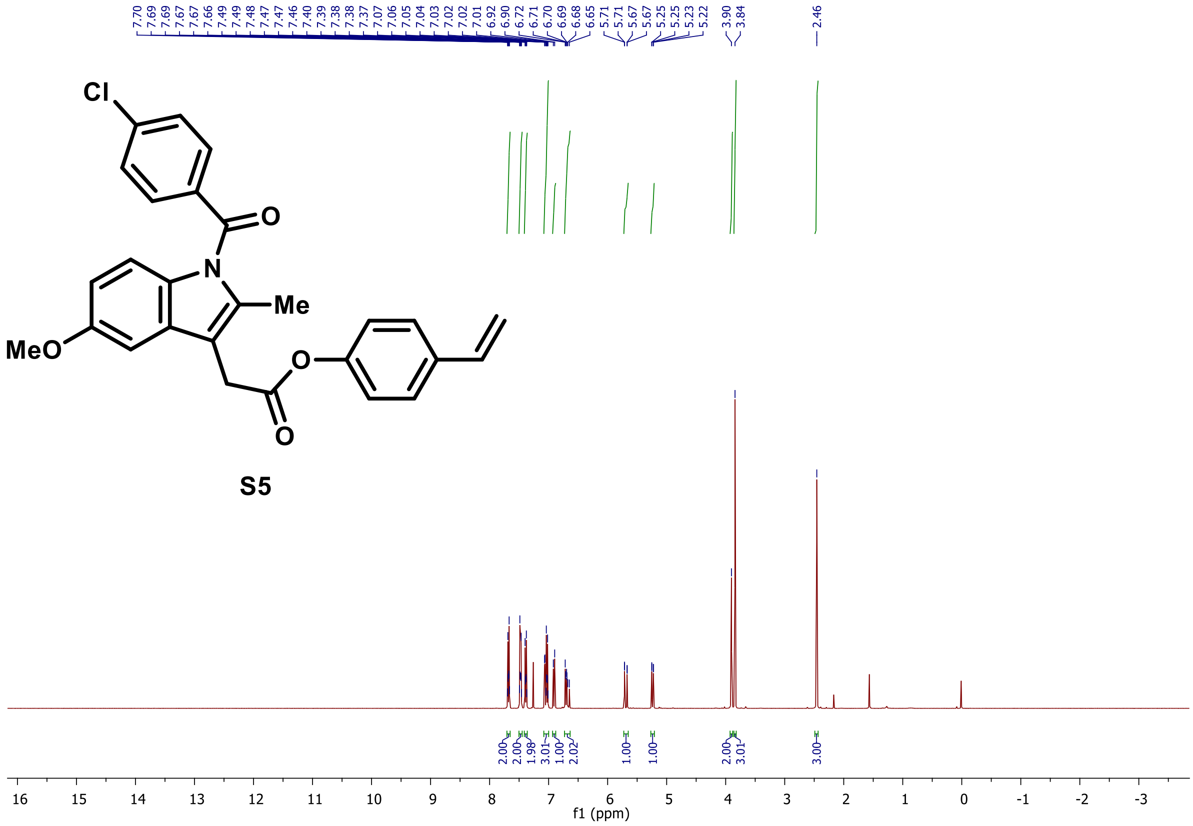

^{13}C NMR (101 MHz, $CDCl_3$) of **S5**

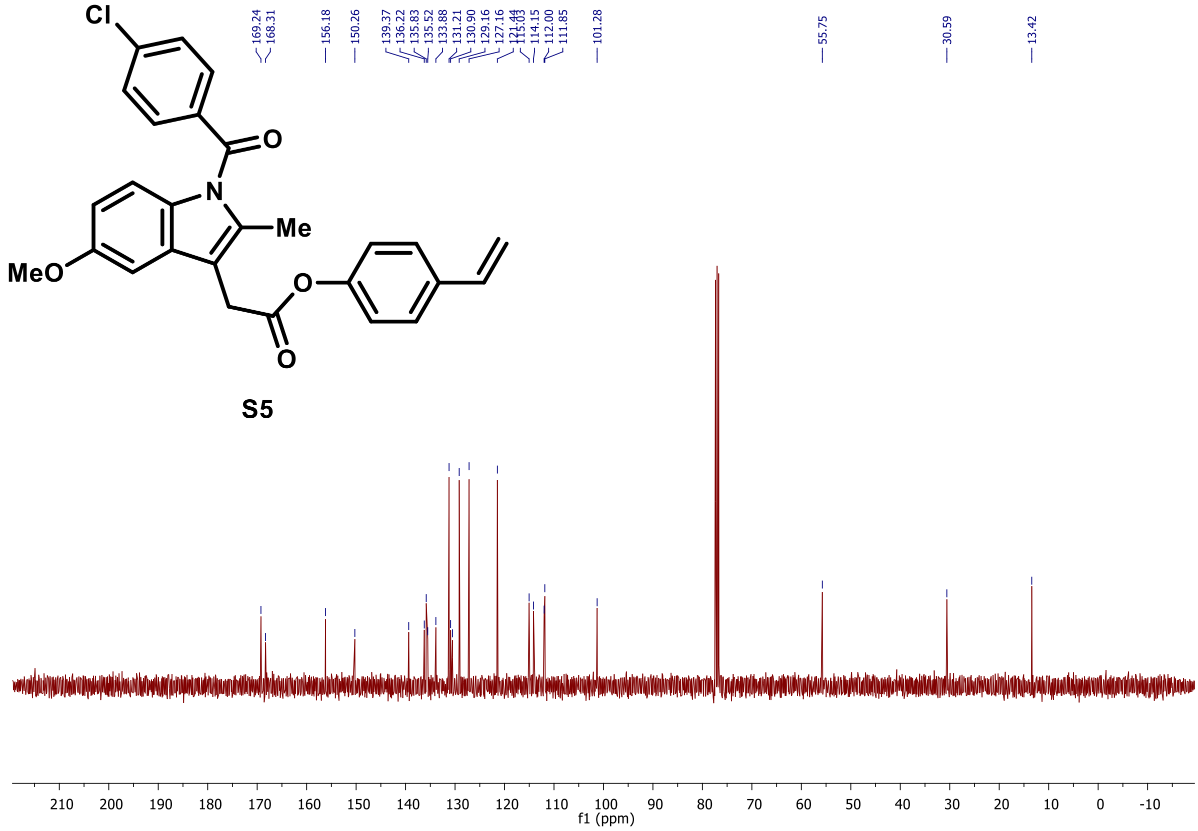

^{1}H NMR (400 MHz, $CDCl_3$) of **S6**

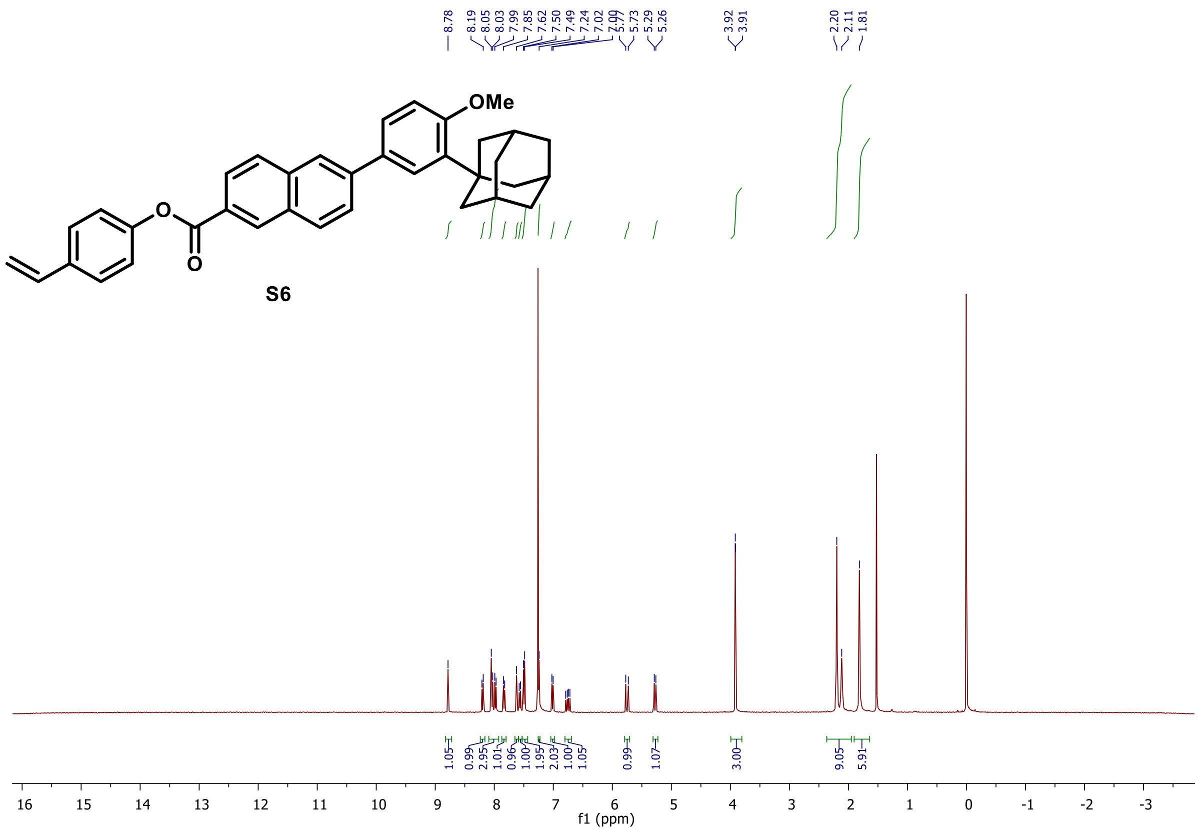

^{13}C NMR (101 MHz, $CDCl_3$) of **S6**

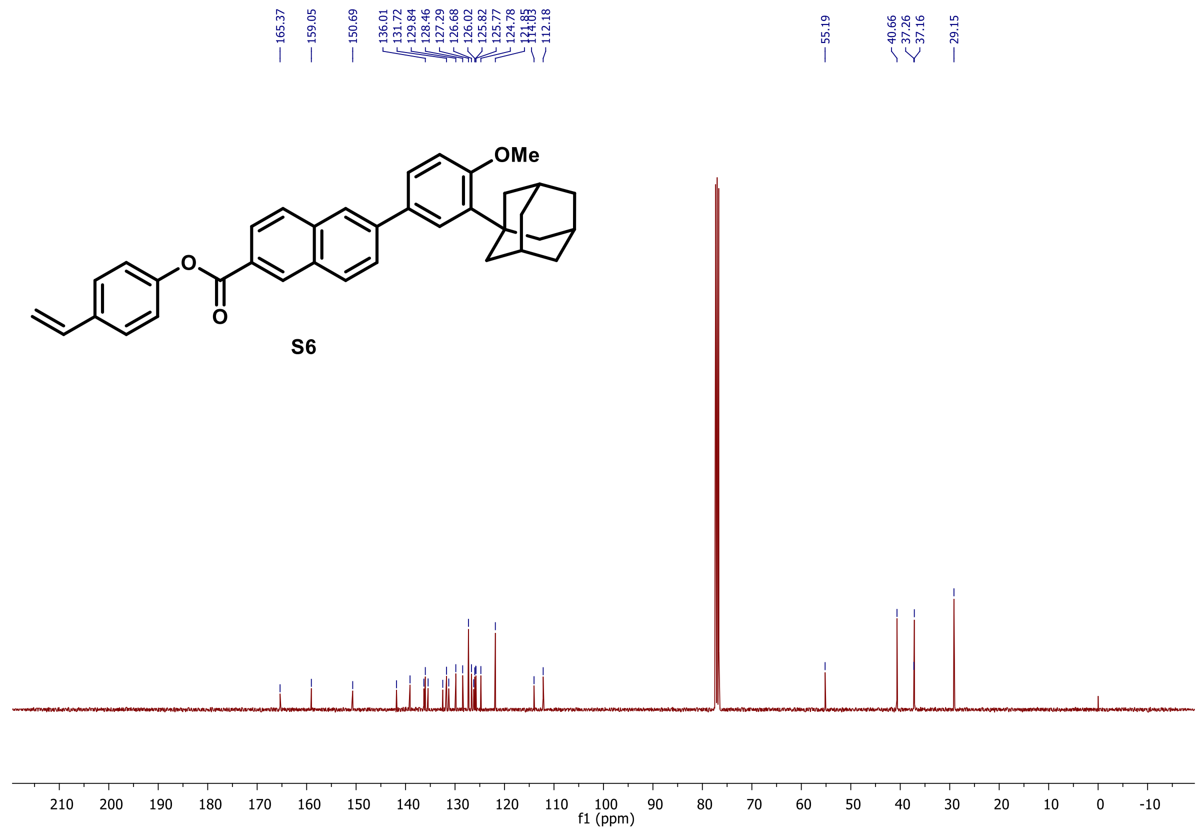

^{1}H NMR (400 MHz, $CDCl_3$) of **1**

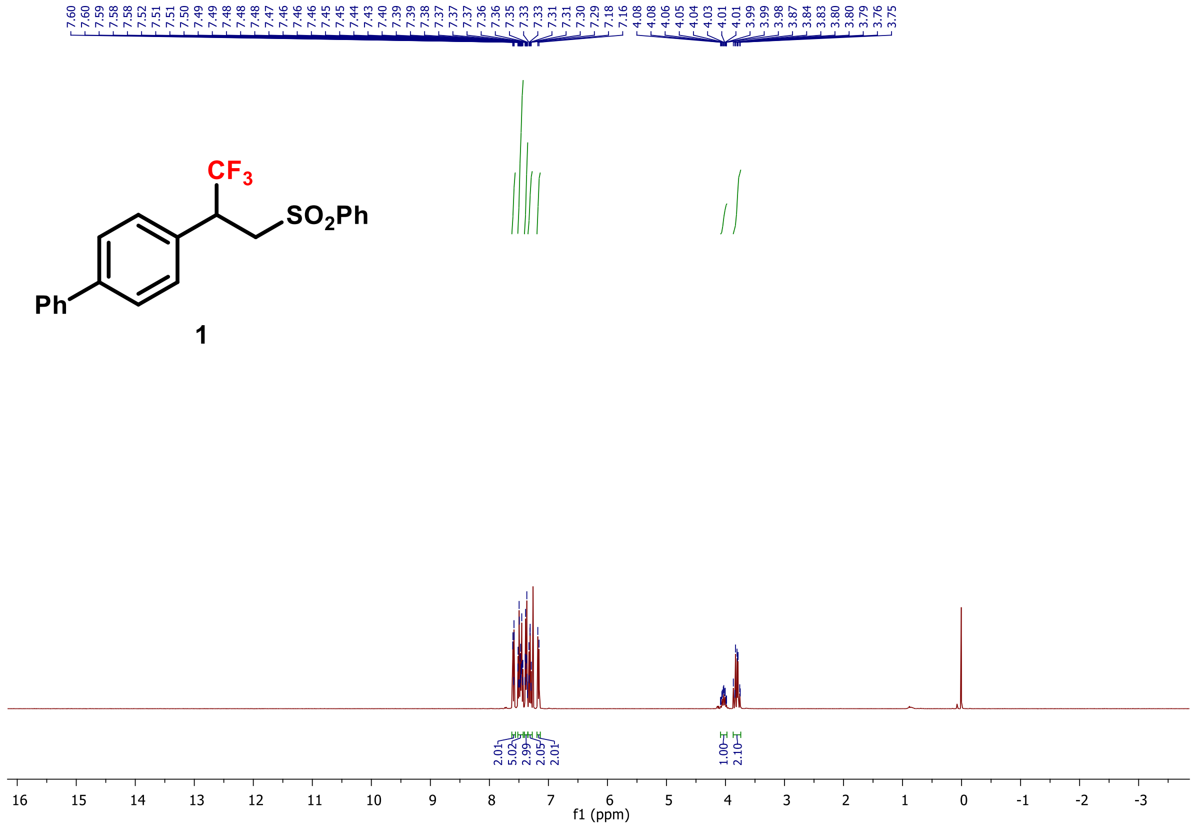

^{19}F NMR (376 MHz, $CDCl_3$) of **1**

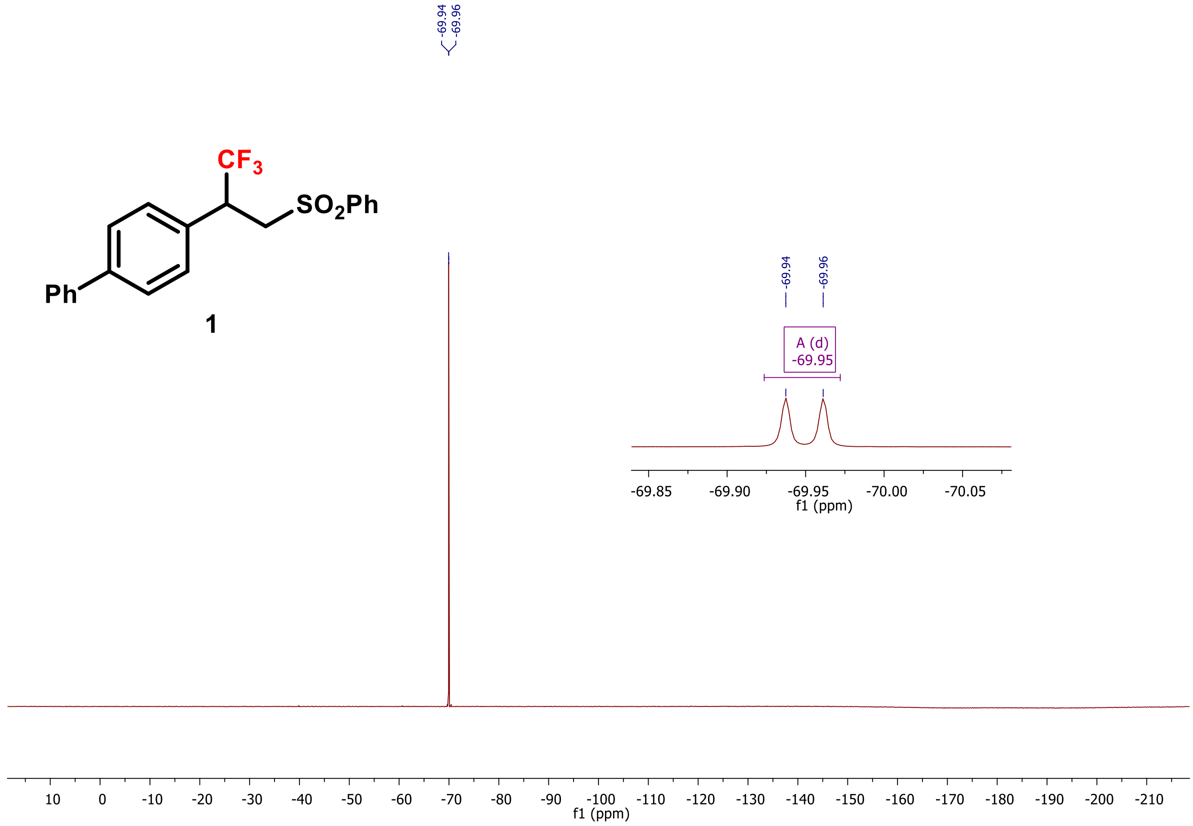

^{13}C NMR (101 MHz, $CDCl_3$) of **1**

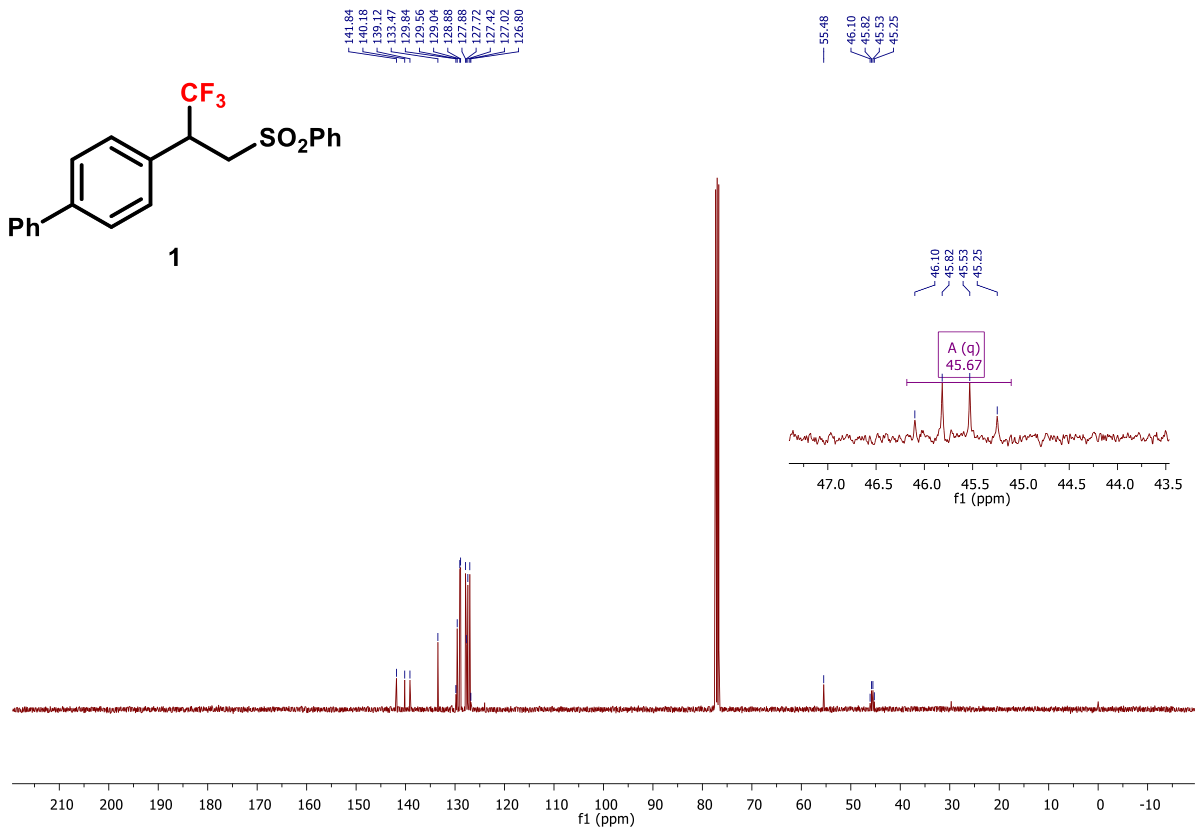

^{1}H NMR (400 MHz, $CDCl_3$) of **2**

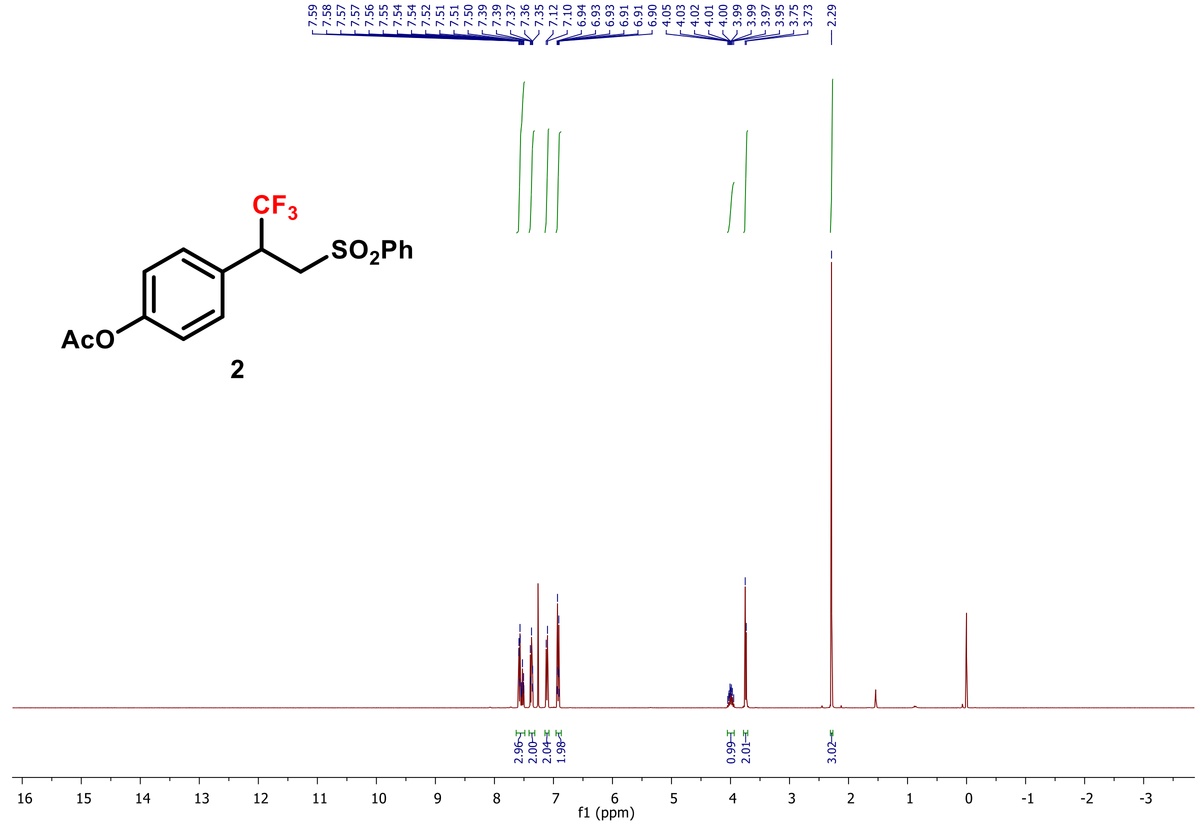

^{19}F NMR (376 MHz, $CDCl_3$) of **2**

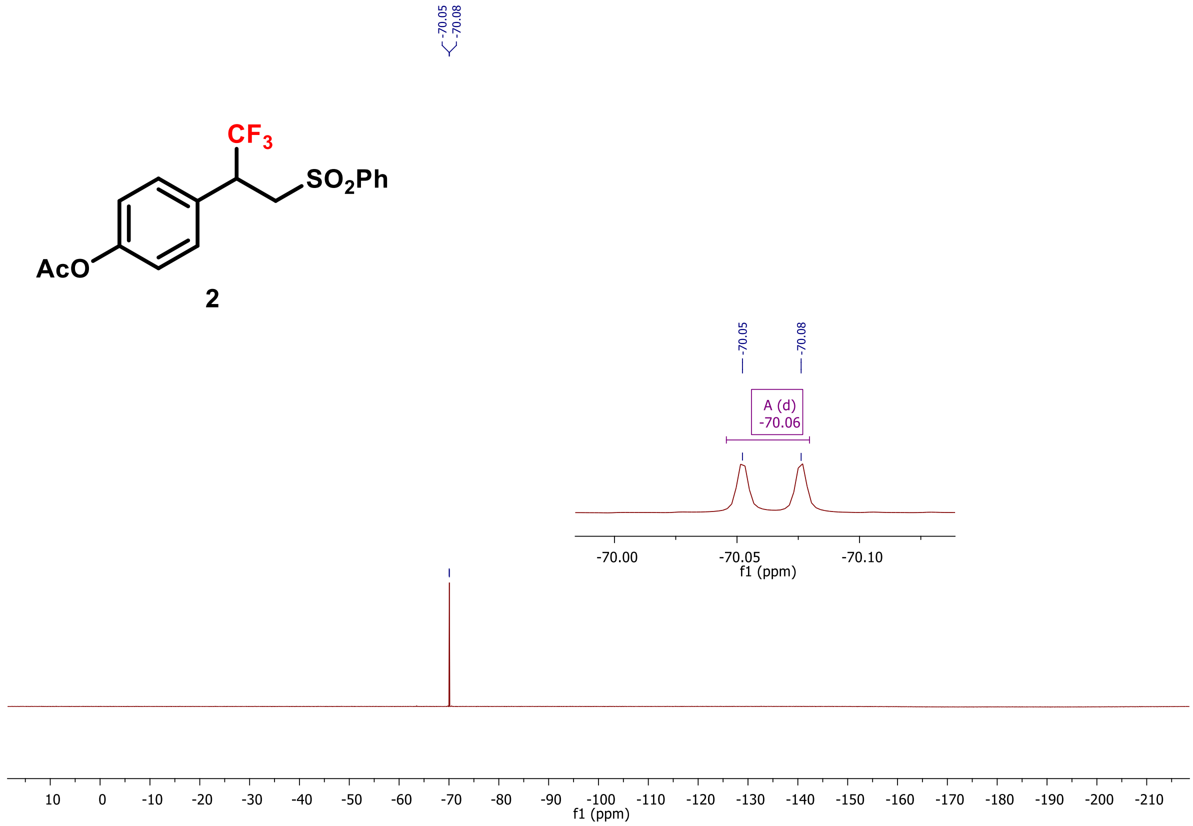

^{13}**C NMR** (101 MHz, $CDCl_3$) of **2**

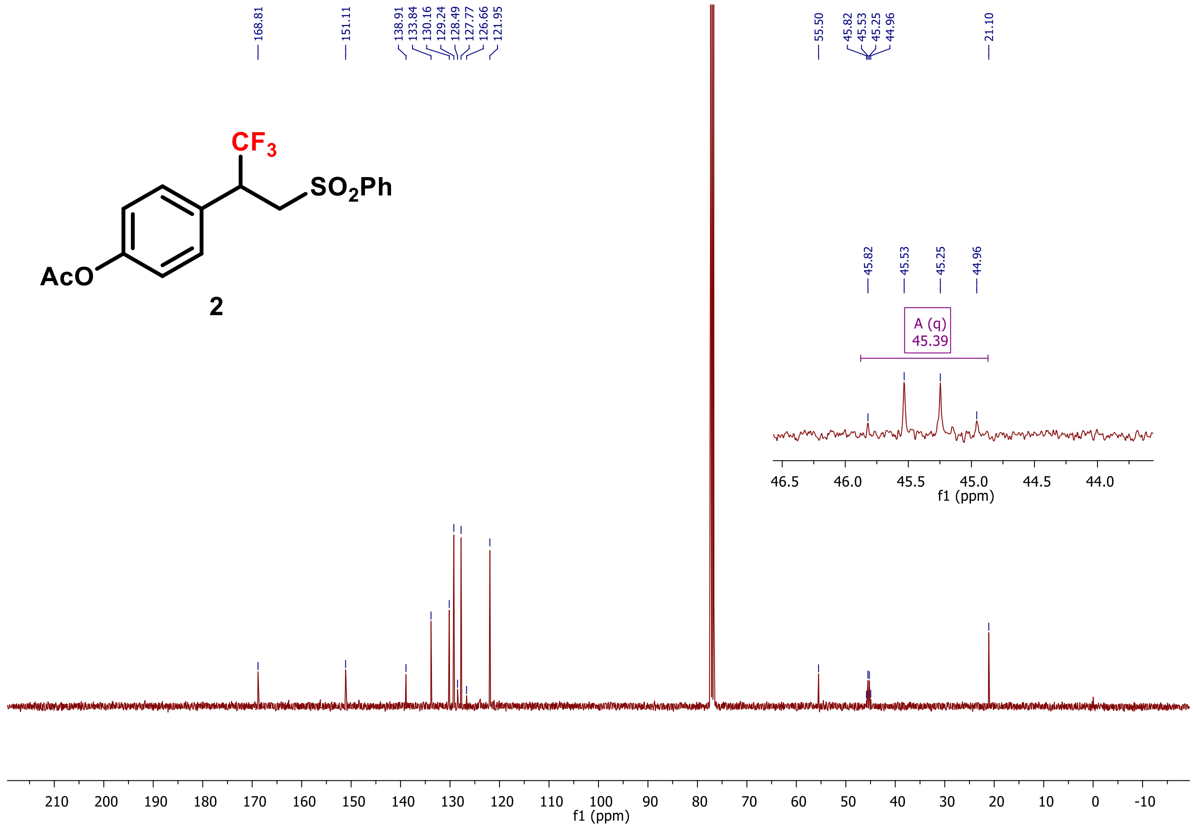

^{1}**H NMR** (400 MHz, $CDCl_3$) of **3**

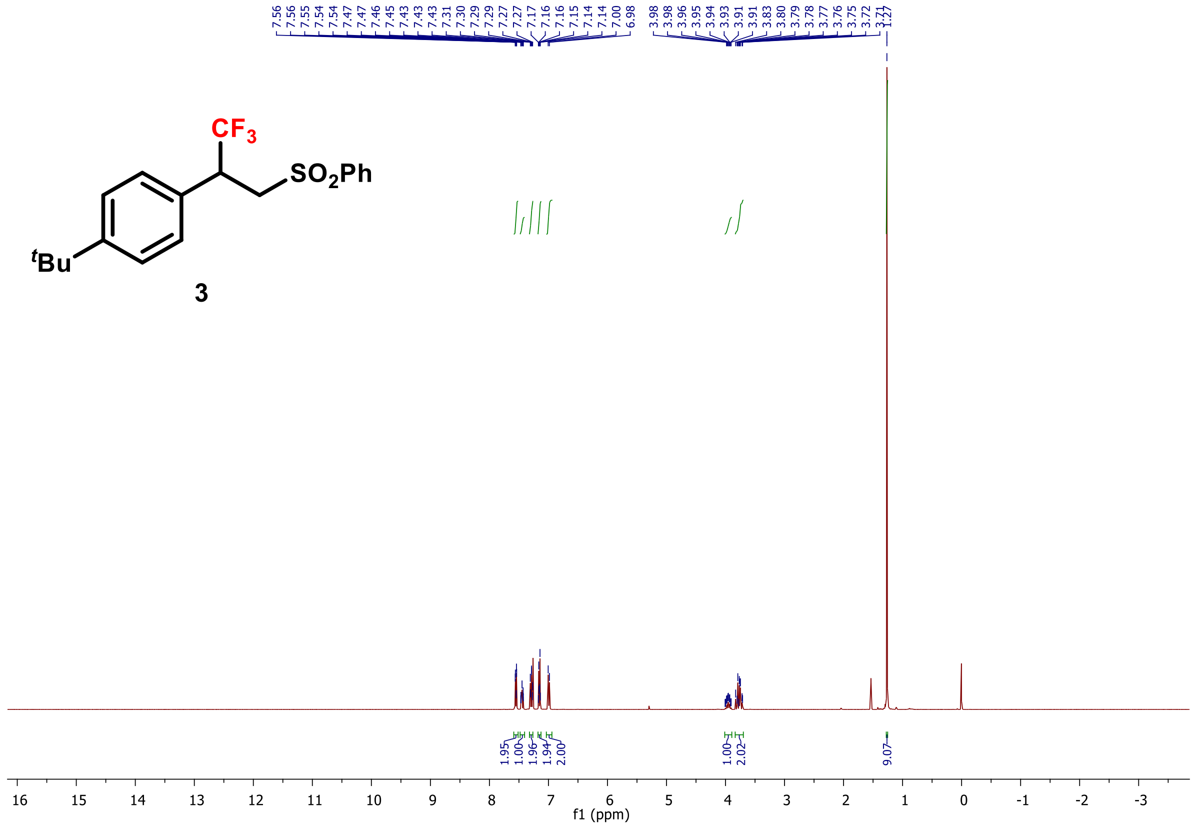

^{19}F NMR (376 MHz, $CDCl_3$) of **3**

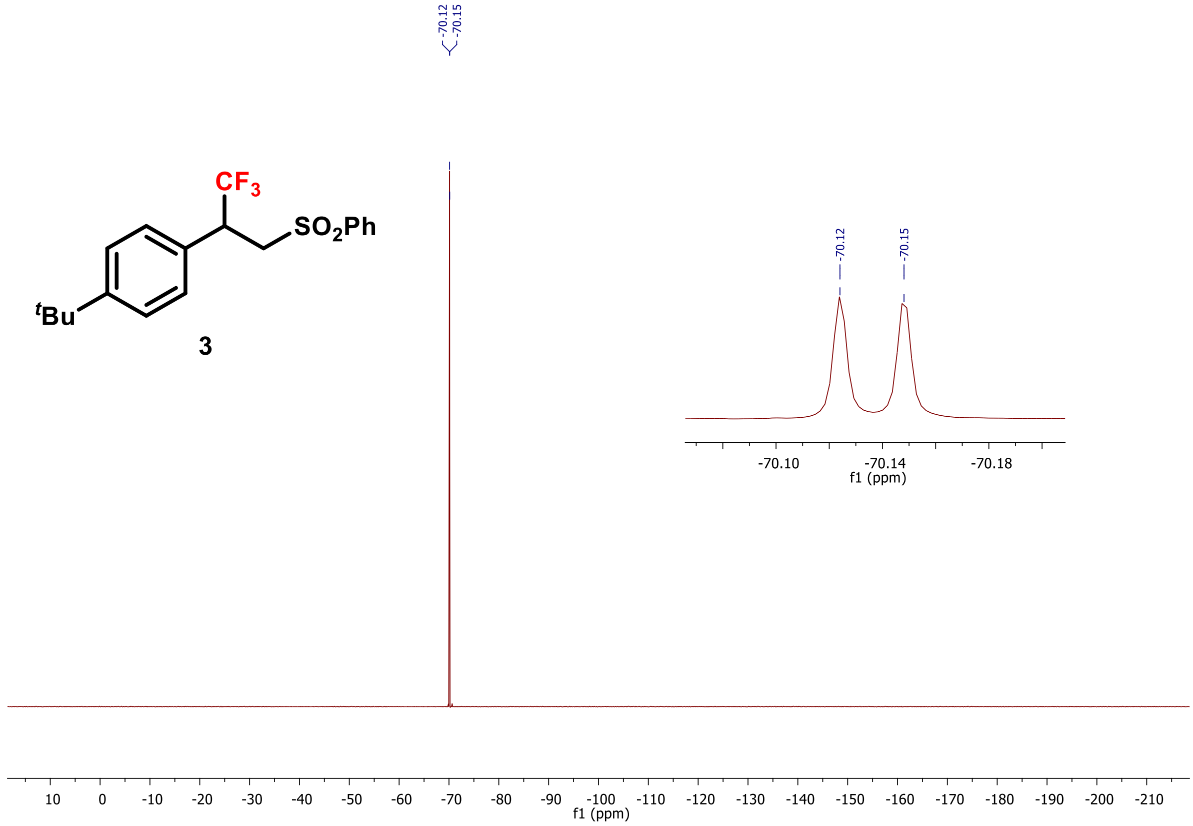

^{13}C NMR (101 MHz, $CDCl_3$) of **3**

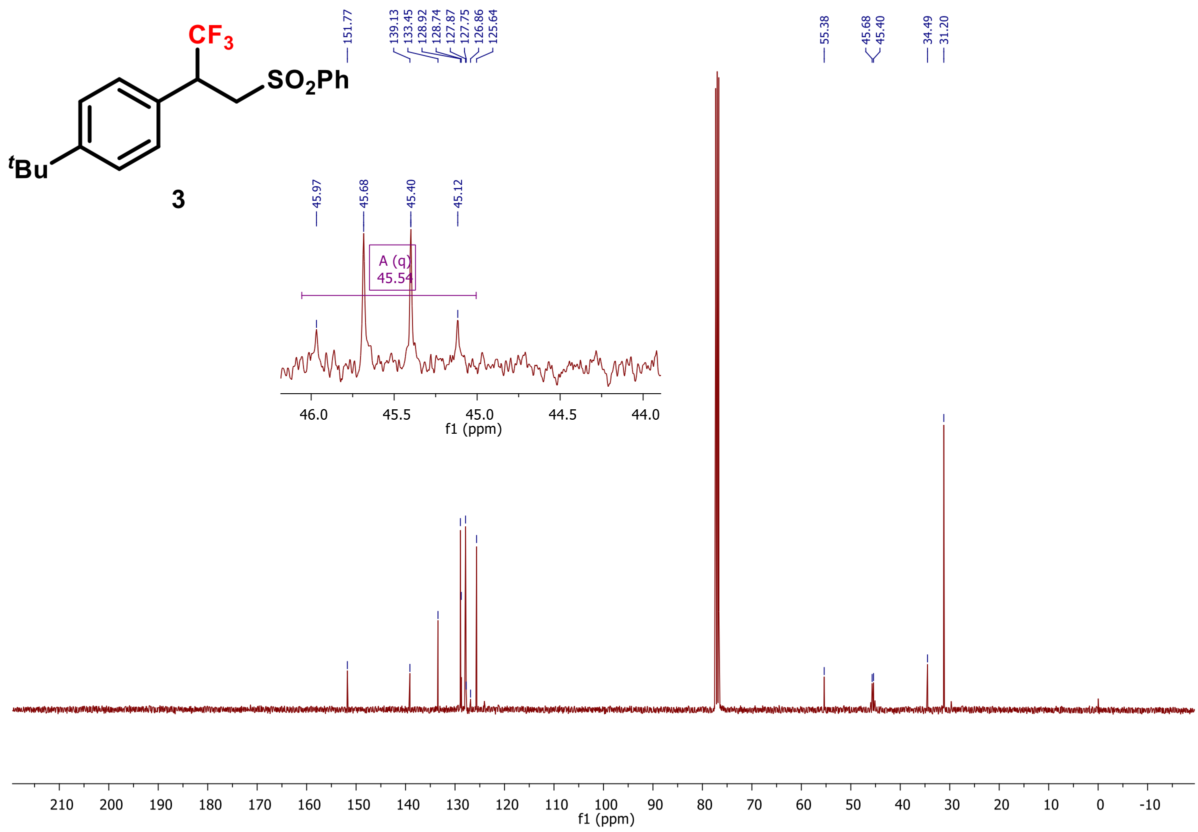

^{1}H NMR (400 MHz, $CDCl_3$) of 4

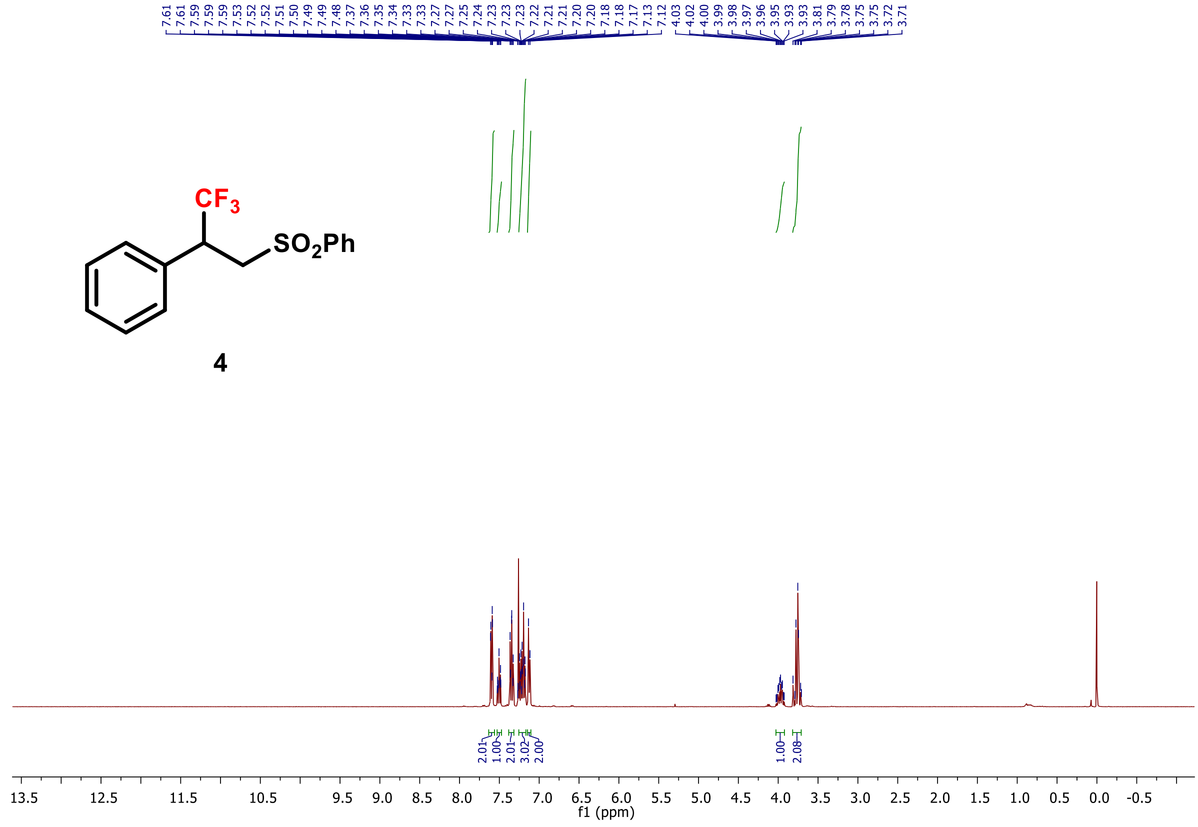

^{19}F NMR (376 MHz, $CDCl_3$) of 4

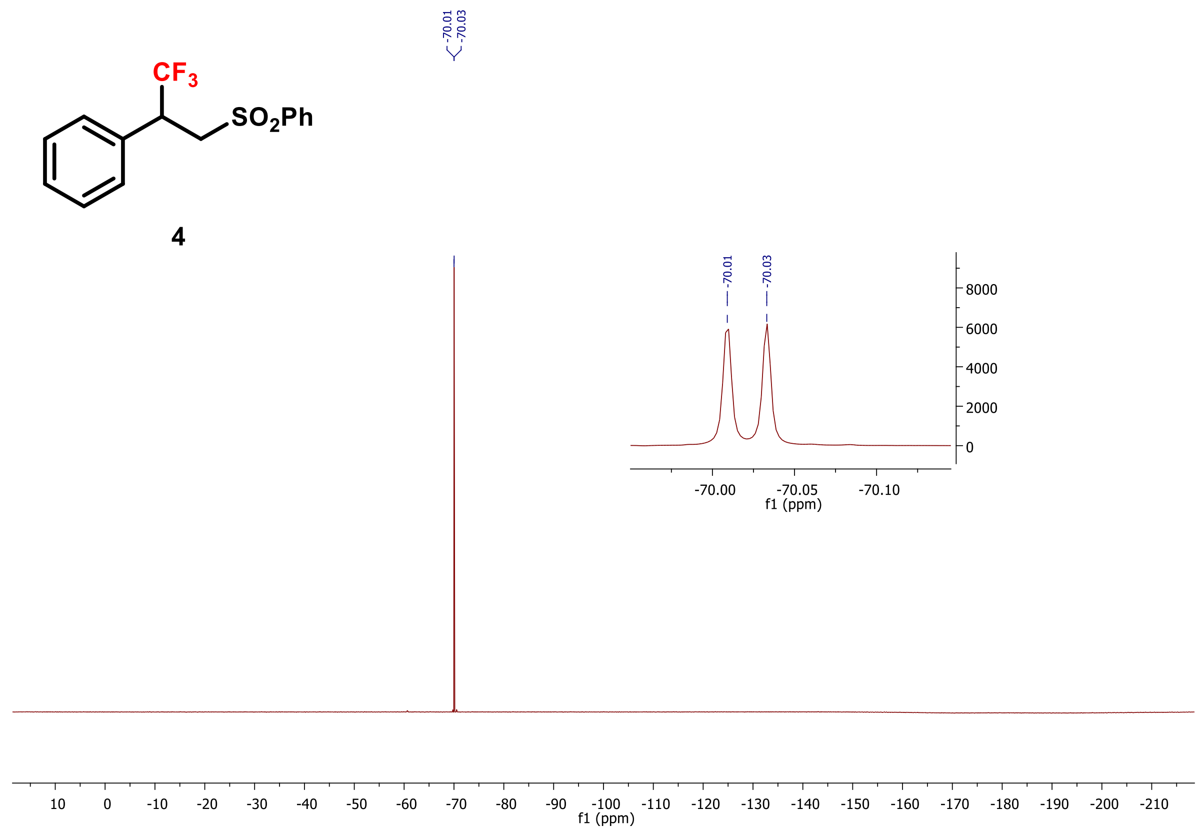

^{13}C NMR (101 MHz, $CDCl_3$) of **4**

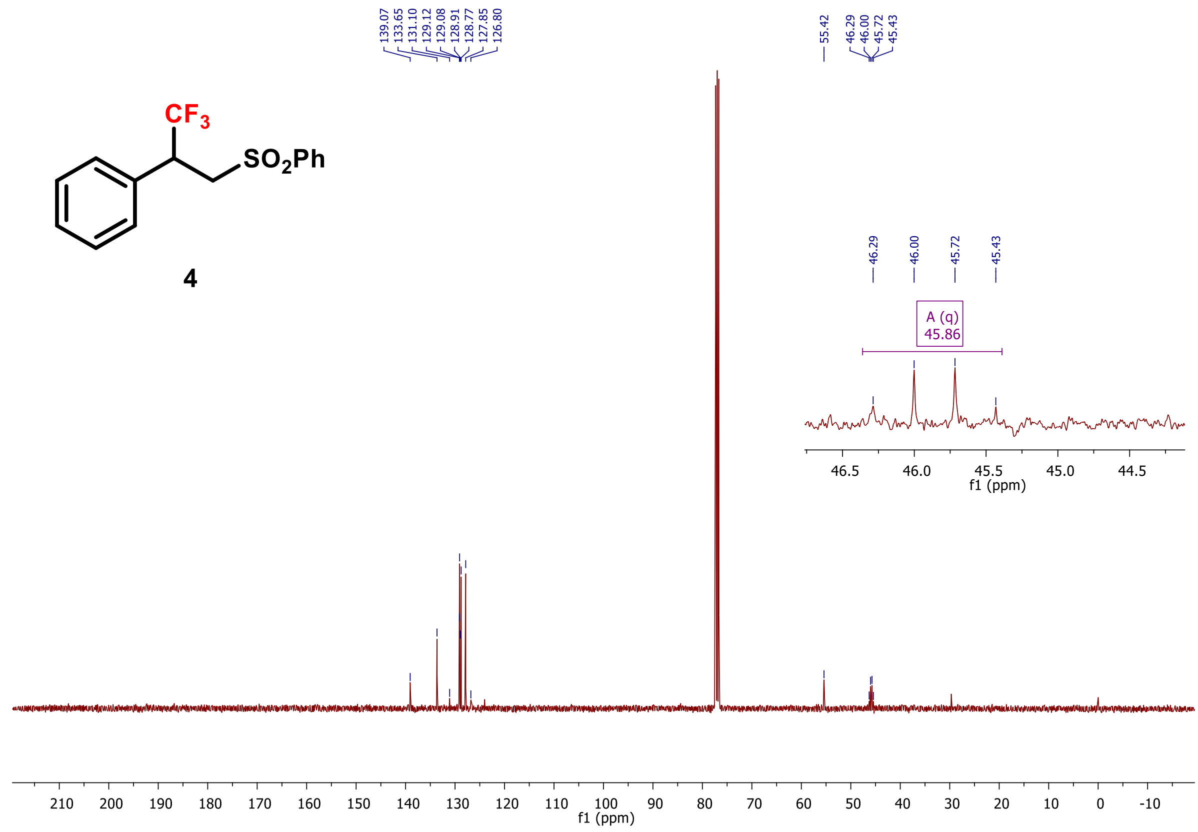

^{1}H NMR (400 MHz, $CDCl_3$) of **5**

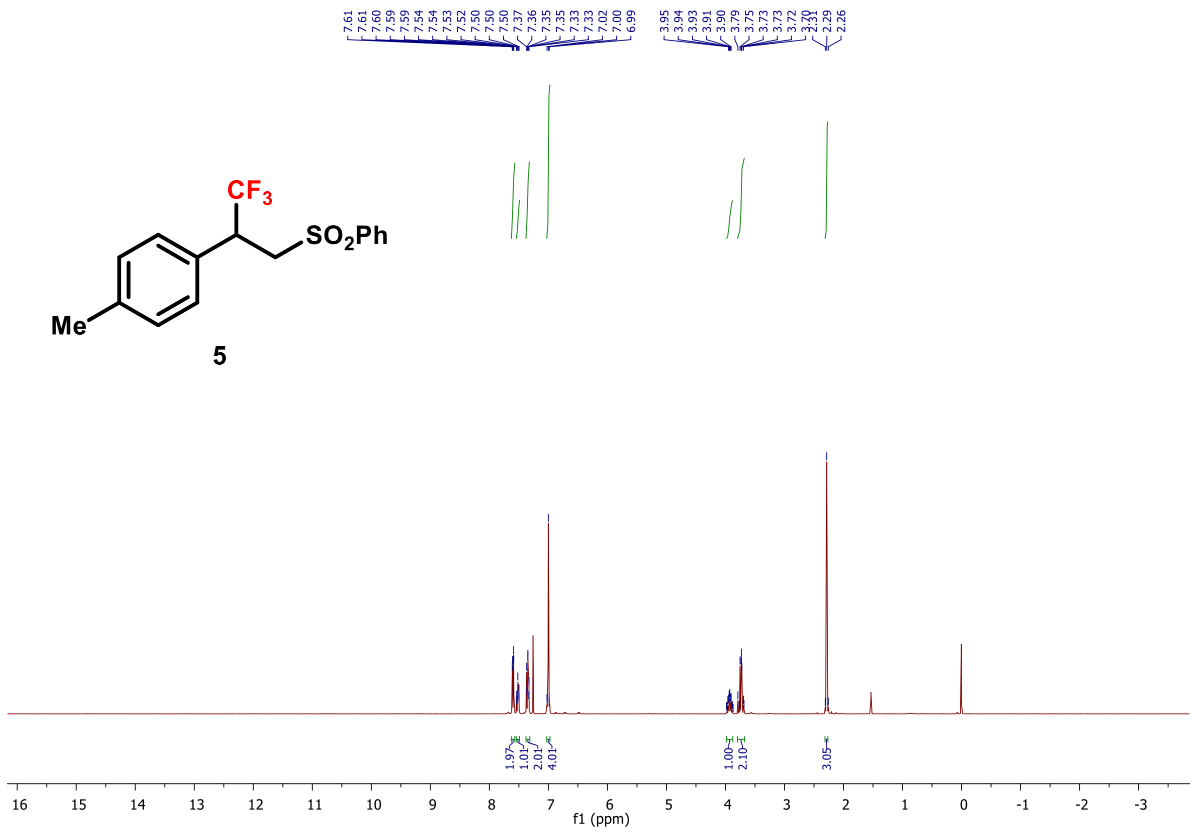

^{19}F NMR (376 MHz, $CDCl_3$) of **5**

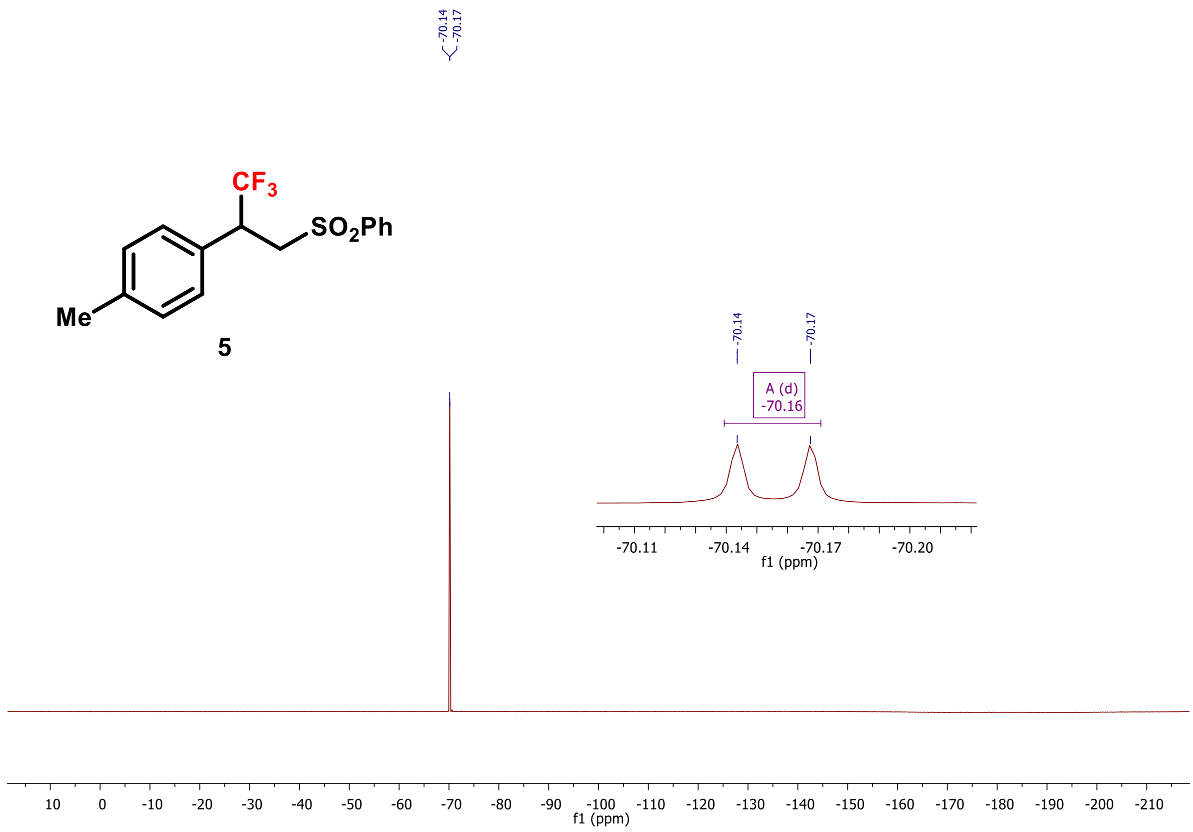

^{13}C NMR (101 MHz, $CDCl_3$) of **5**

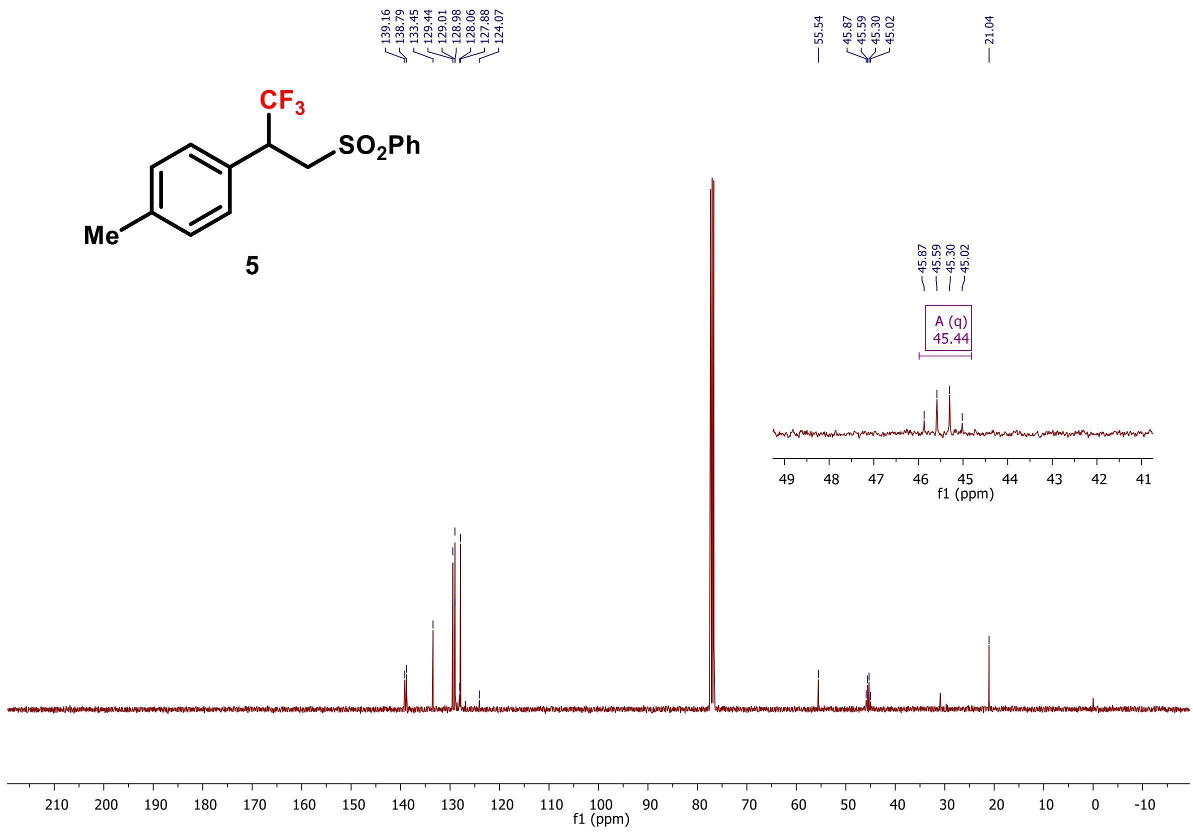

^{1}H NMR (400 MHz, $CDCl_3$) of **6**

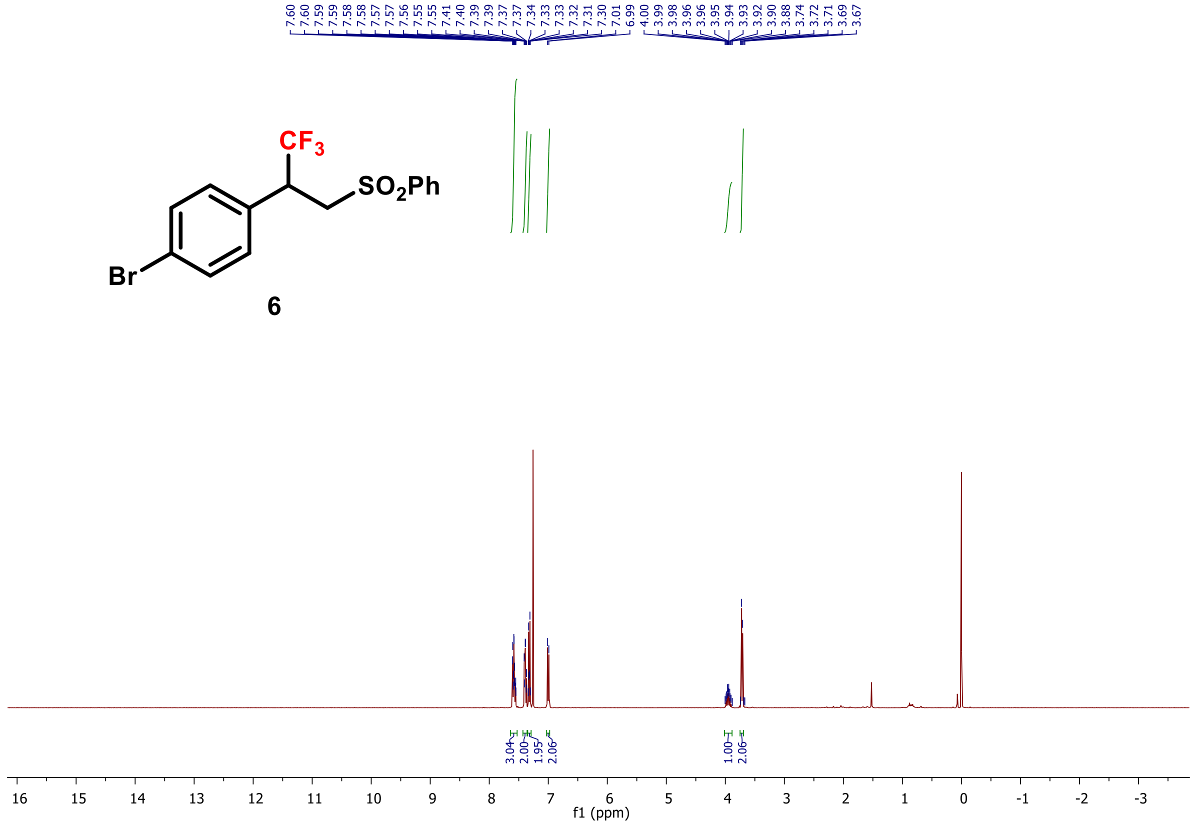

^{19}F NMR (376 MHz, $CDCl_3$) of **6**

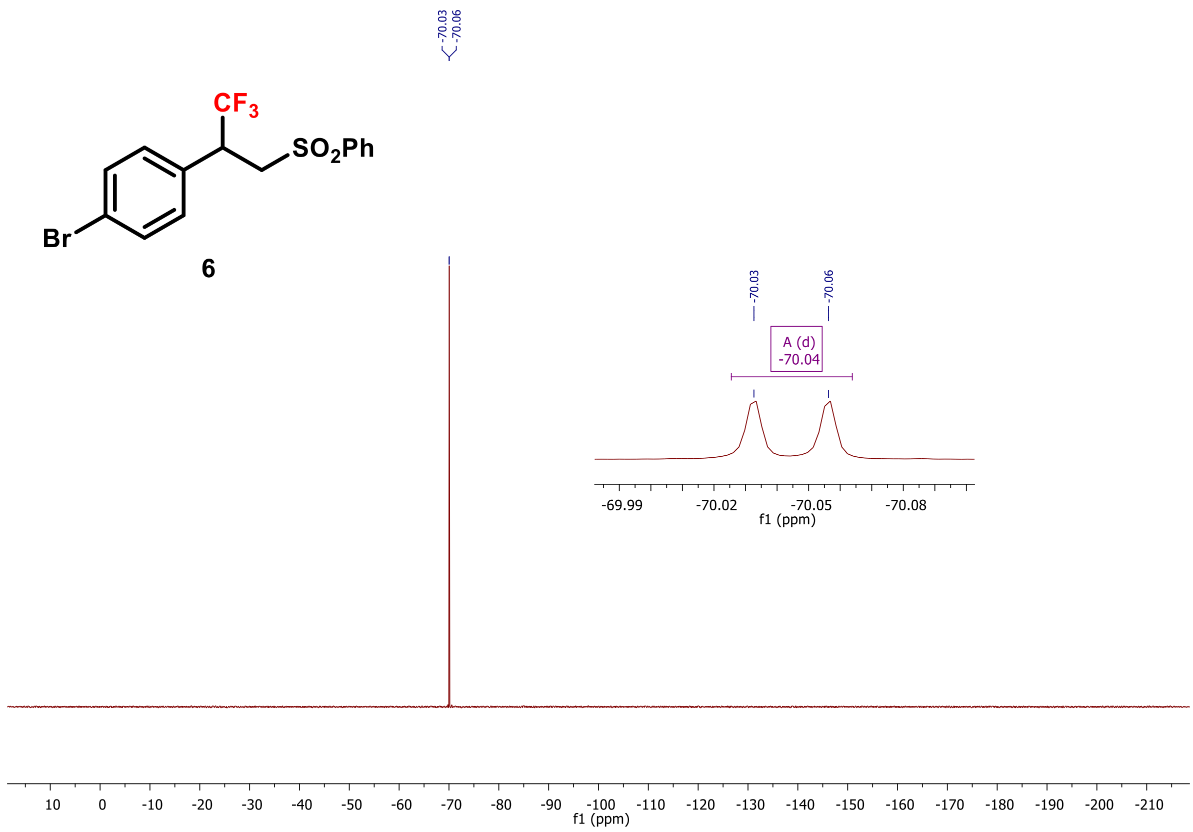

^{13}C NMR (101 MHz, $CDCl_3$) of **6**

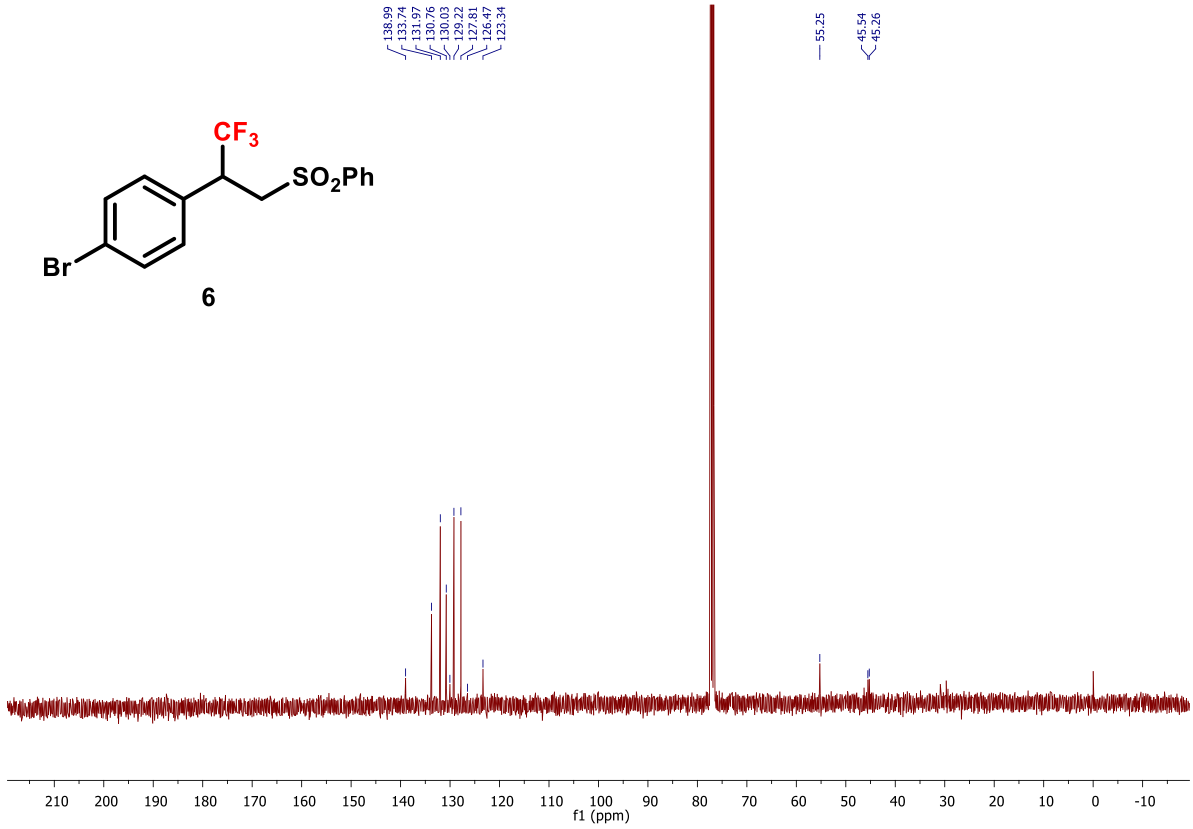

^{1}H NMR (400 MHz, $CDCl_3$) of **7**

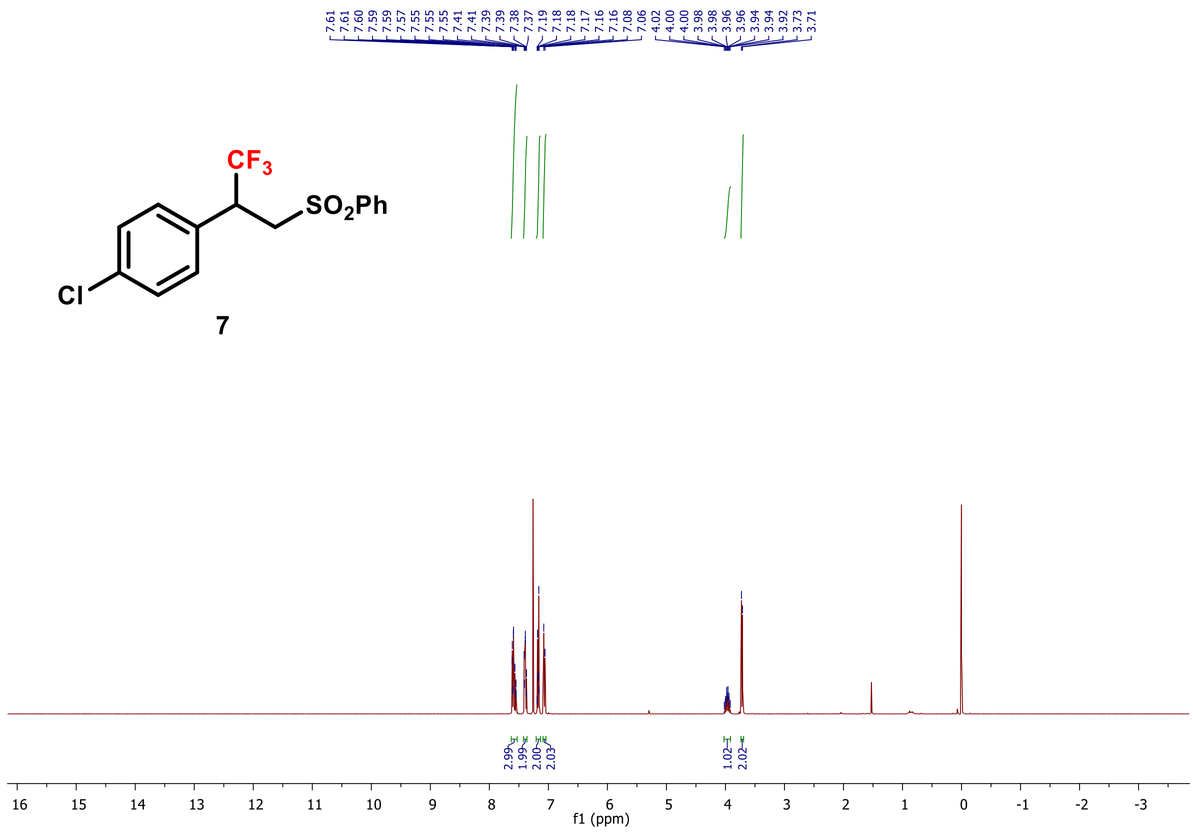

^{19}F NMR (376 MHz, $CDCl_3$) of **7**

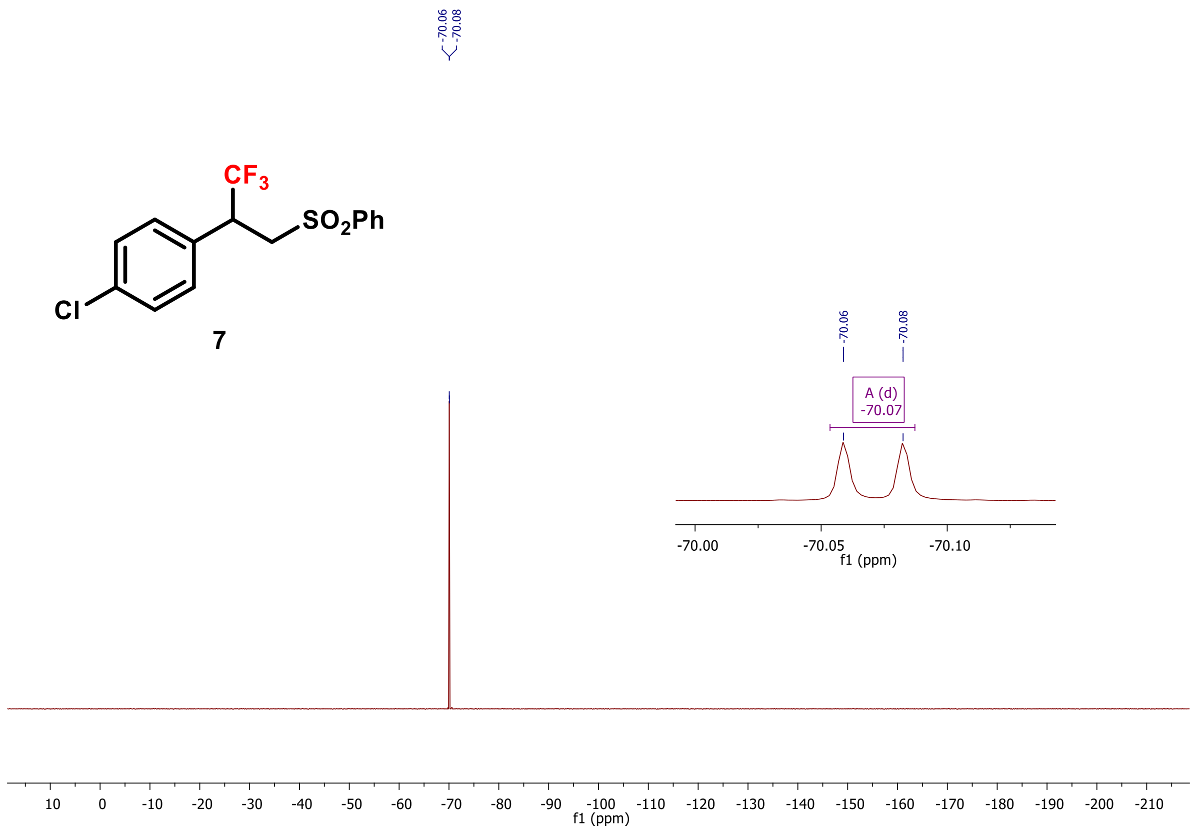

^{13}C NMR (101 MHz, $CDCl_3$) of **7**

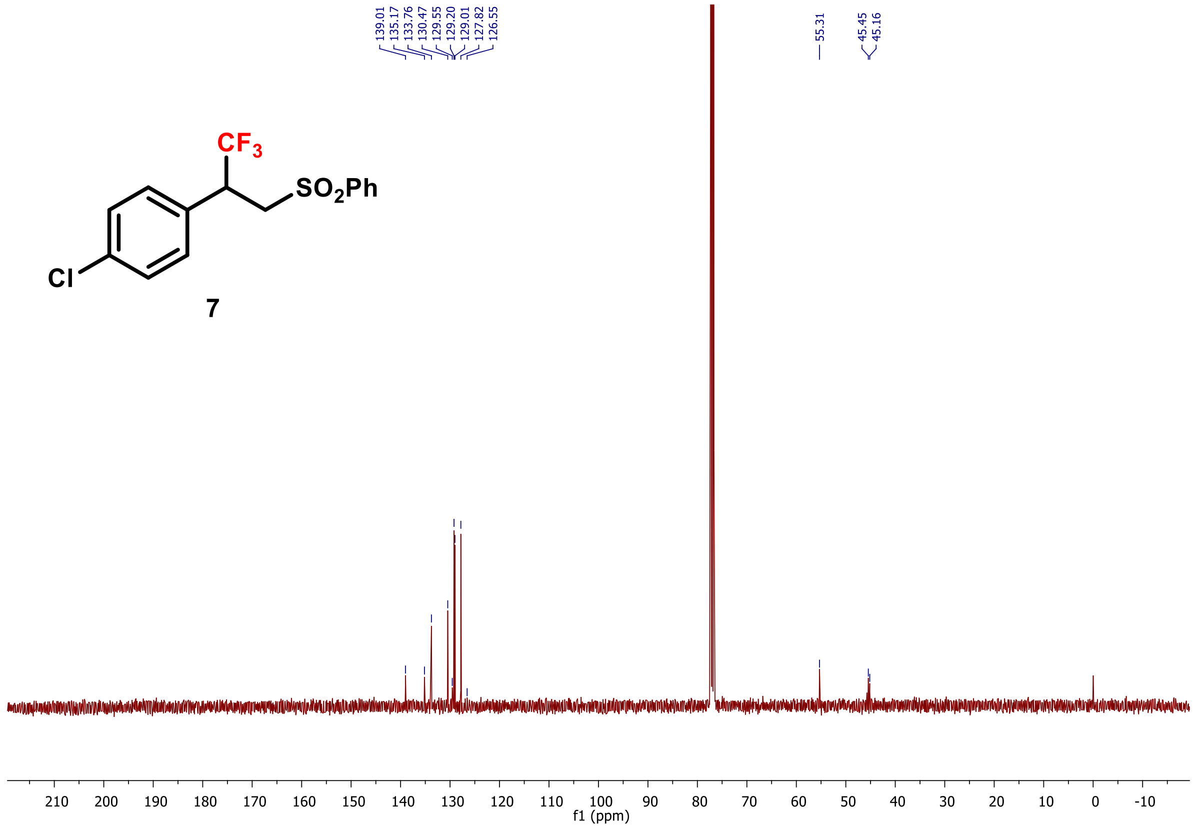

^{1}H NMR (400 MHz, $CDCl_3$) of **8**

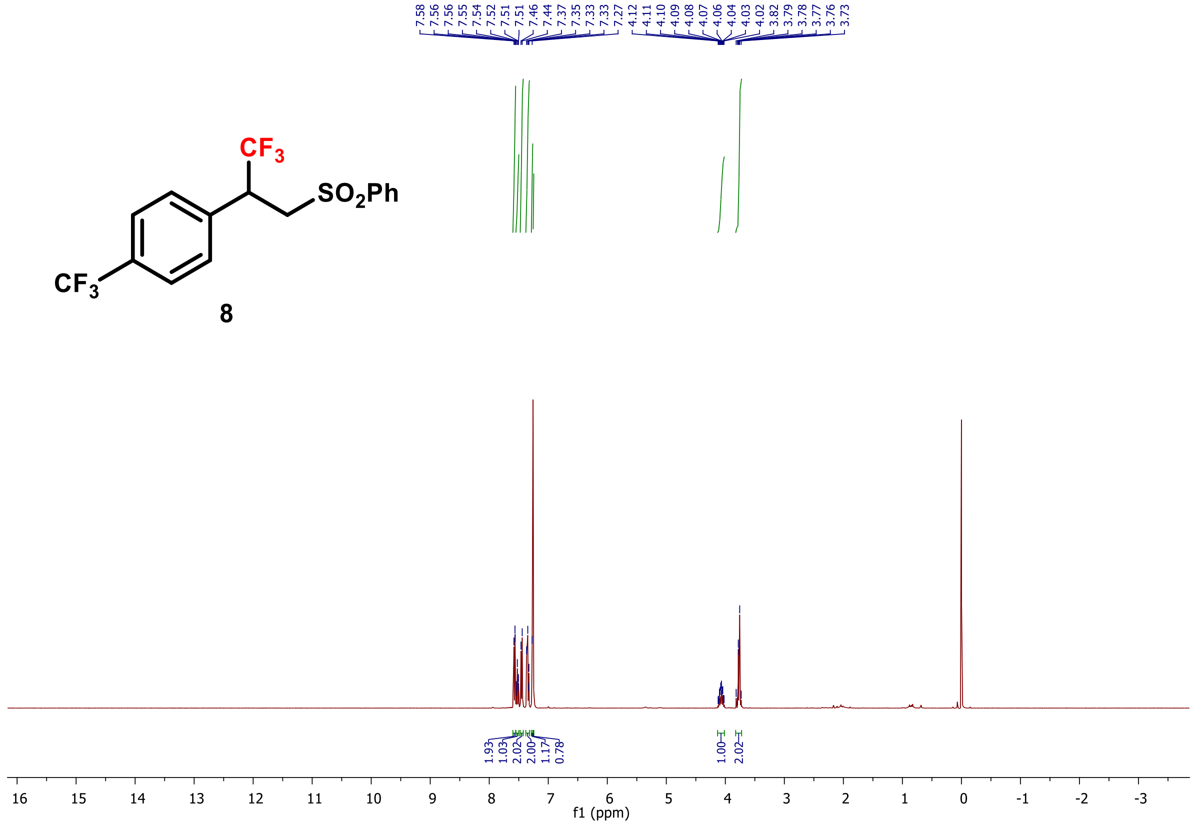

^{19}F NMR (376 MHz, $CDCl_3$) of **8**

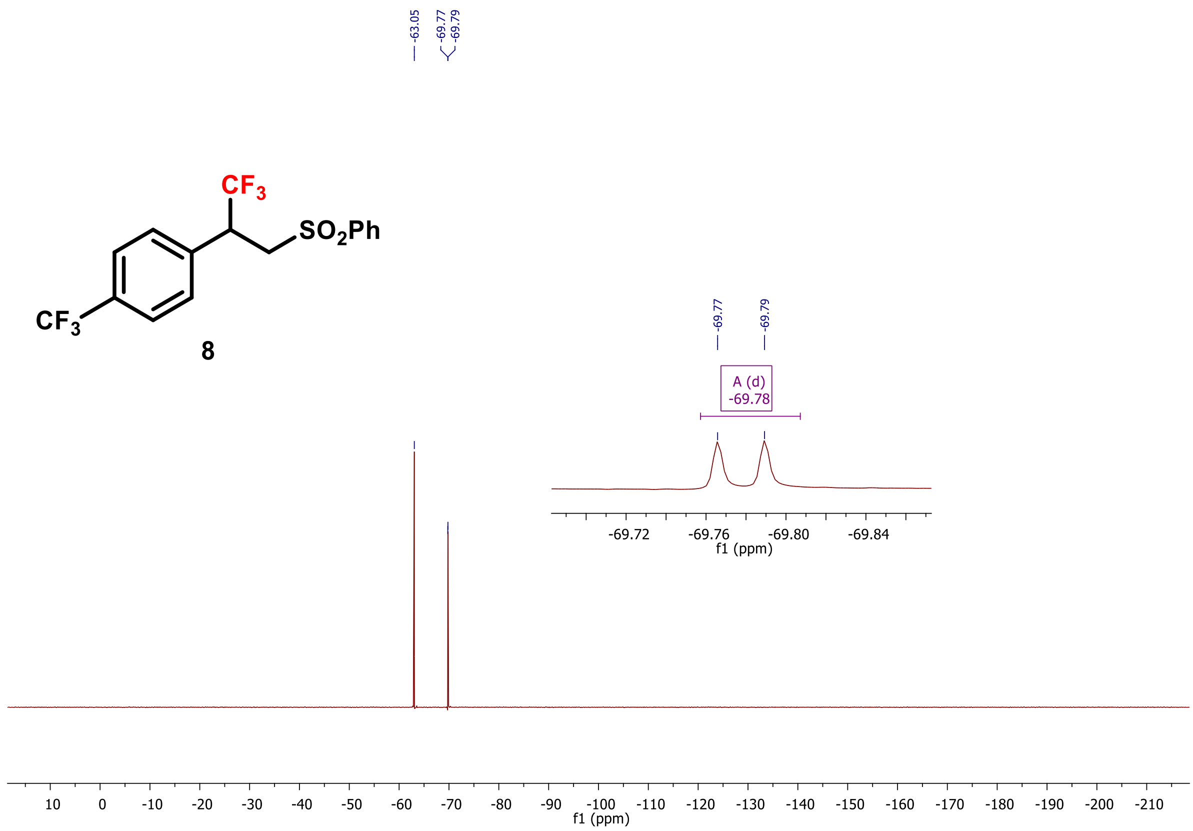

^{13}**C NMR** (101 MHz, $CDCl_3$) of **8**

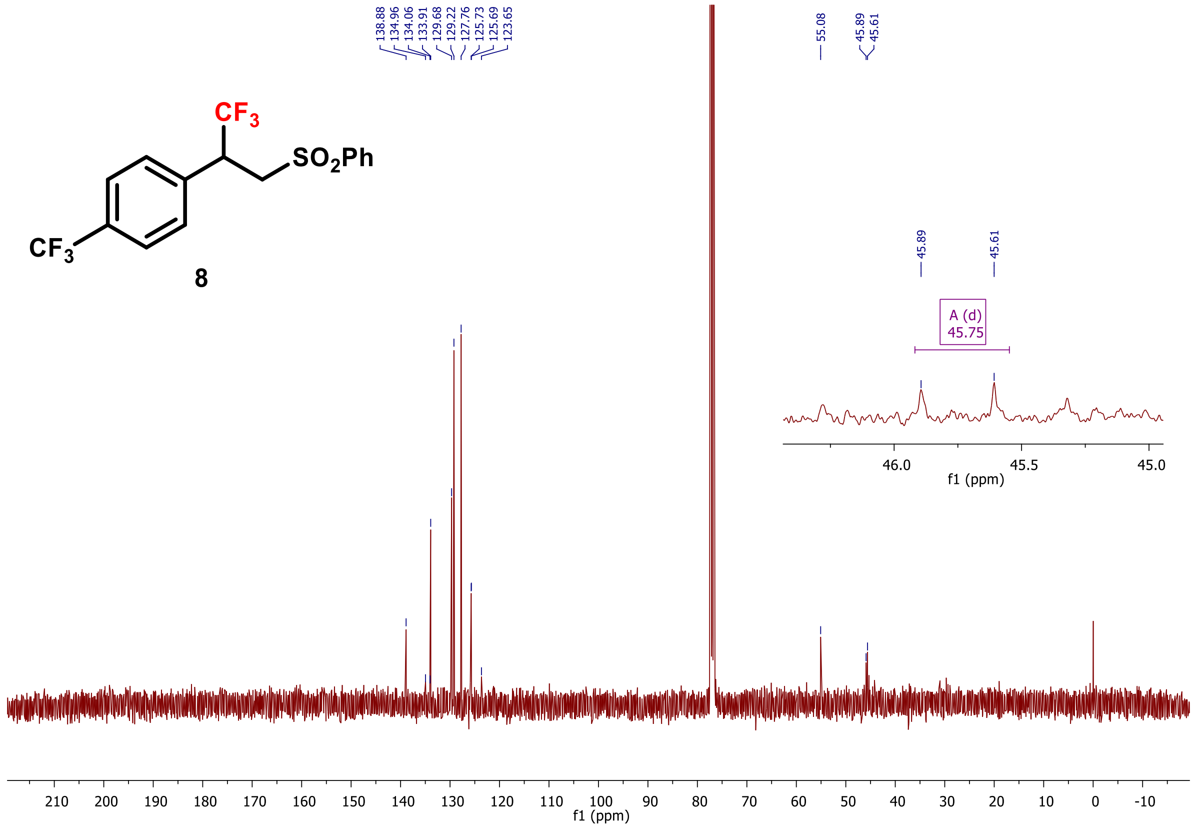

^{1}**H NMR** (400 MHz, $CDCl_3$) of **9**

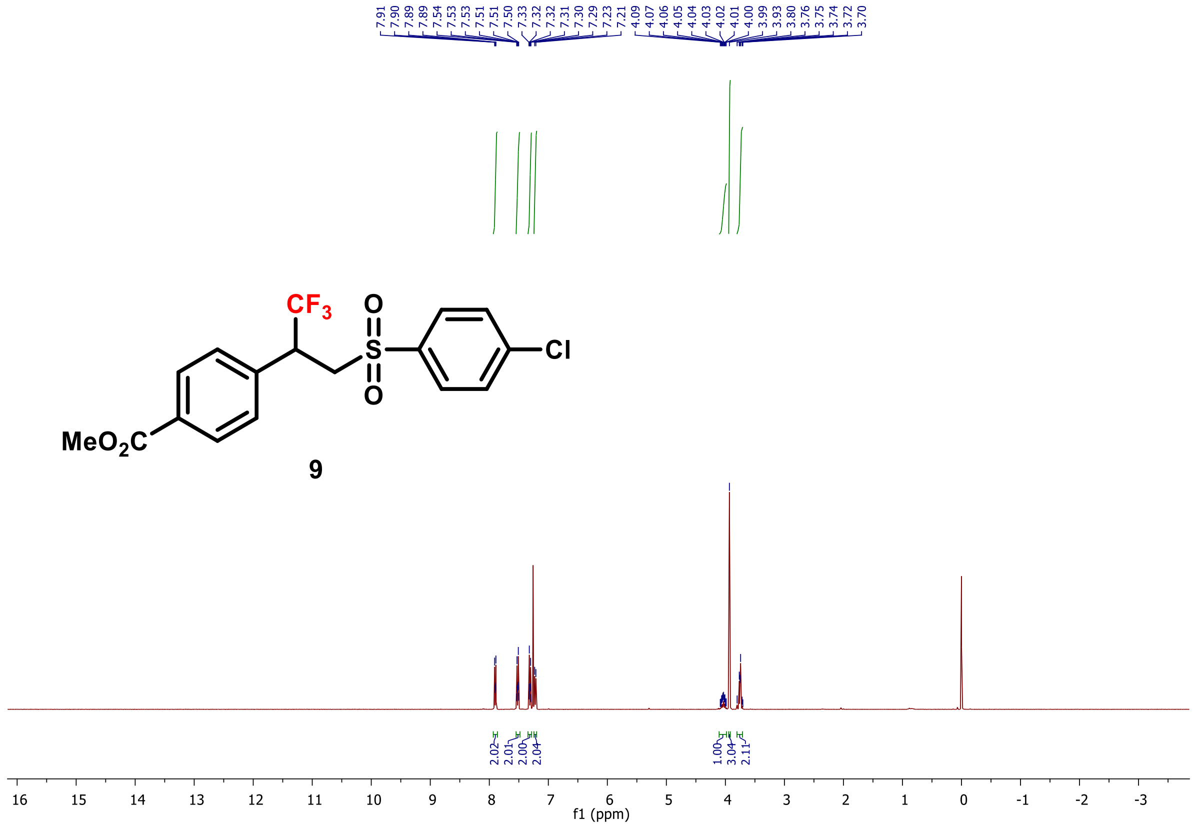

^{19}F NMR (376 MHz, $CDCl_3$) of **9**

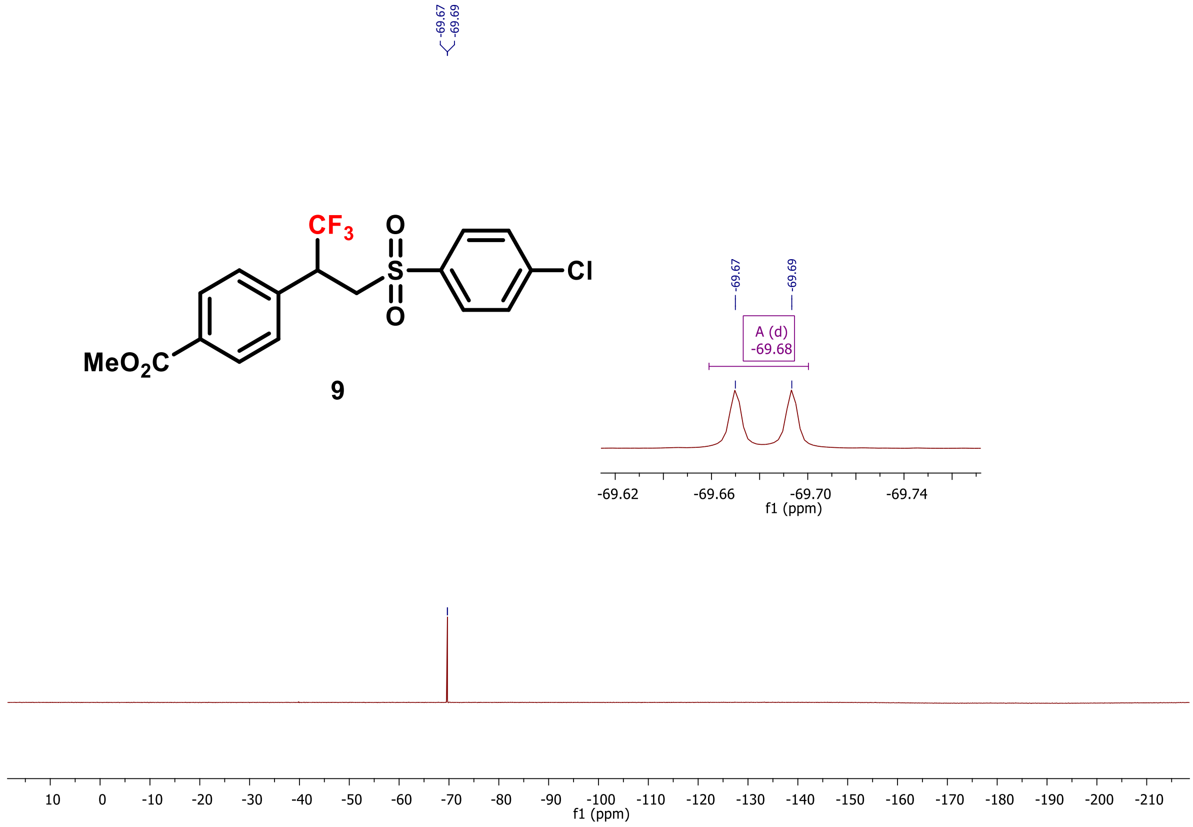

^{13}C NMR (101 MHz, $CDCl_3$) of **9**

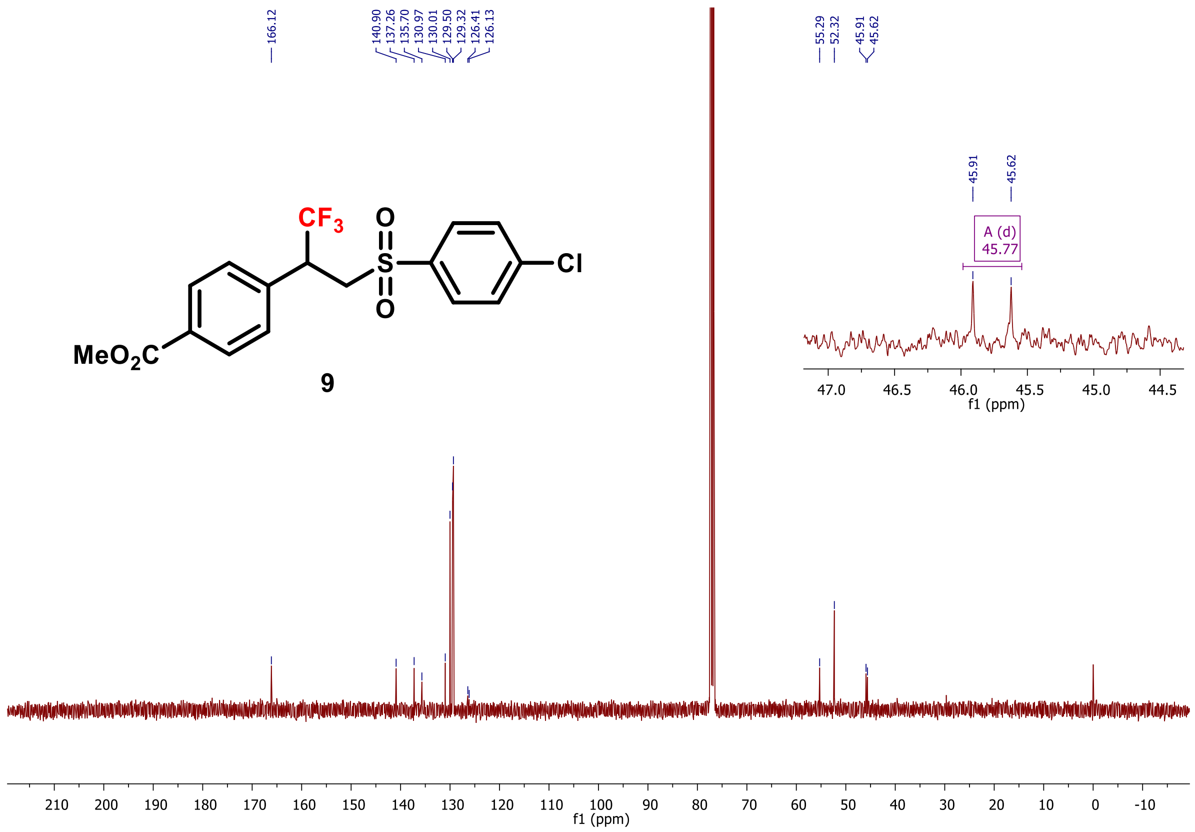

^{1}H NMR (400 MHz, $CDCl_3$) of **10**

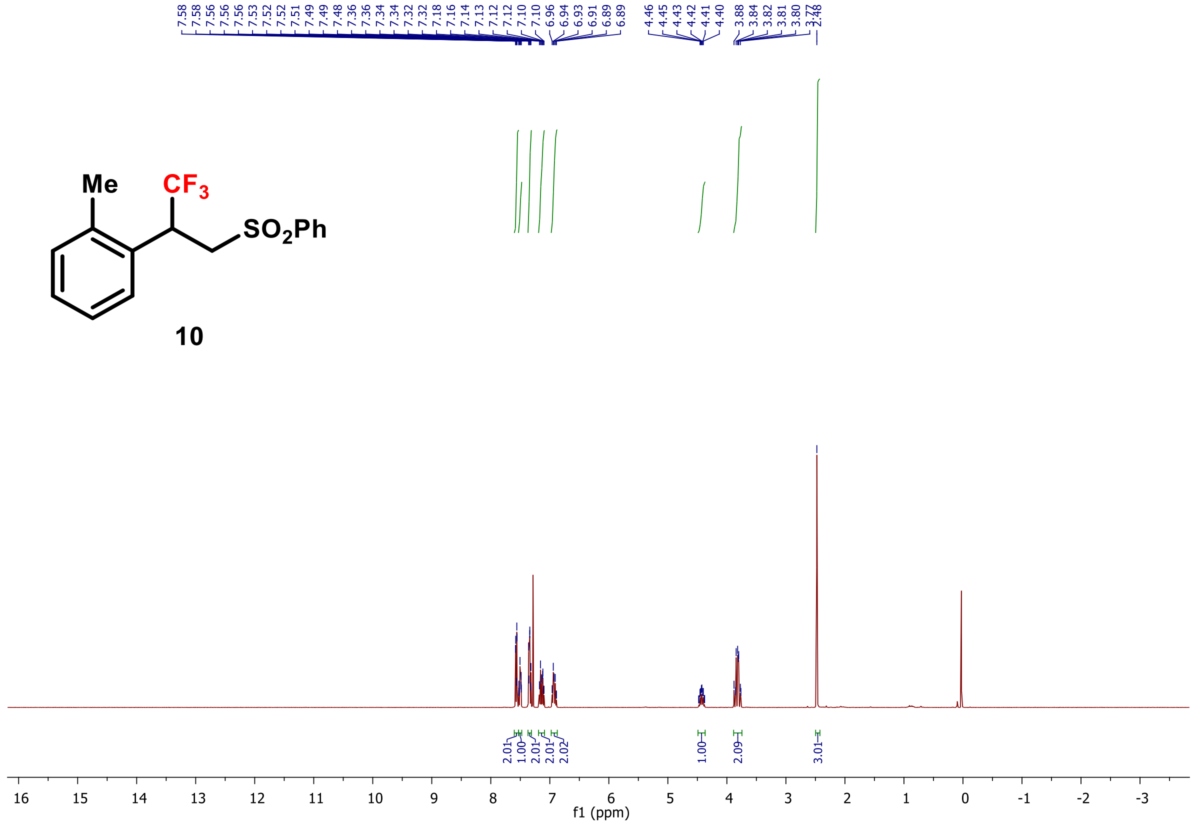

^{19}F NMR (376 MHz, $CDCl_3$) of **10**

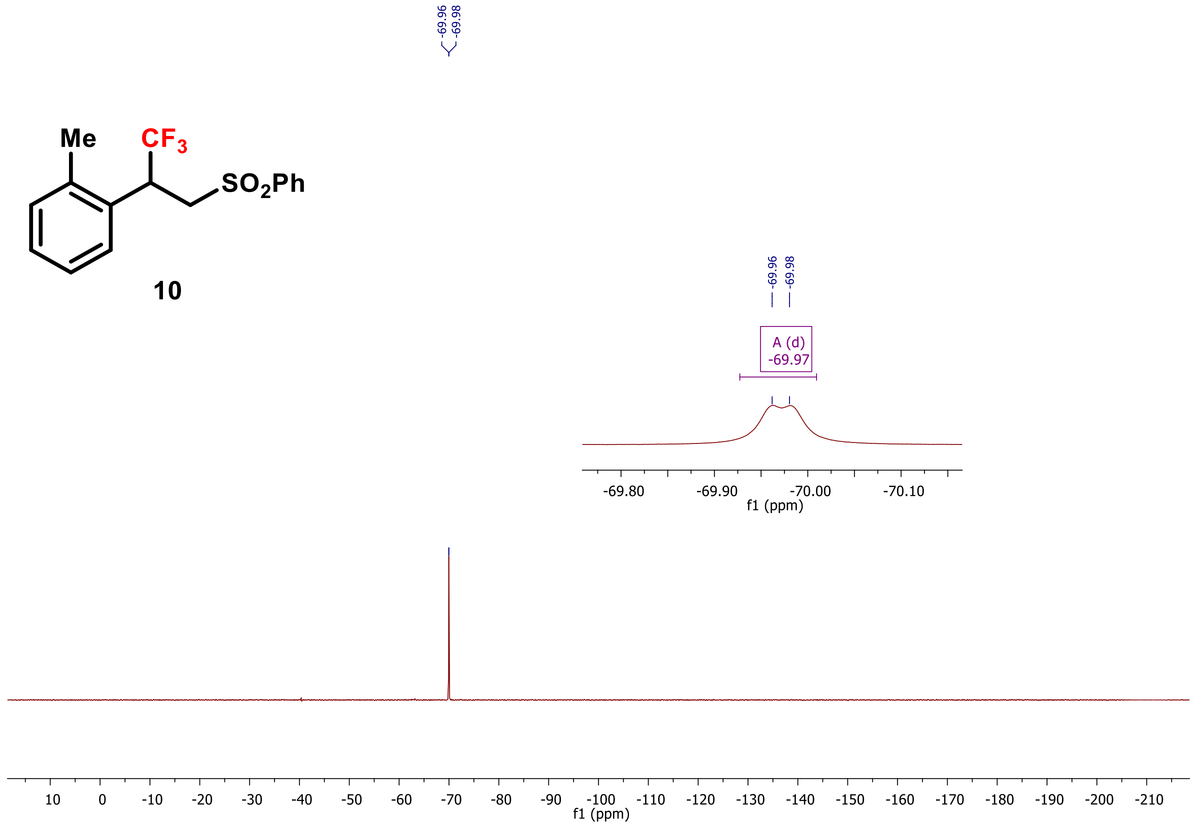

^{13}C NMR (101 MHz, $CDCl_3$) of **10**

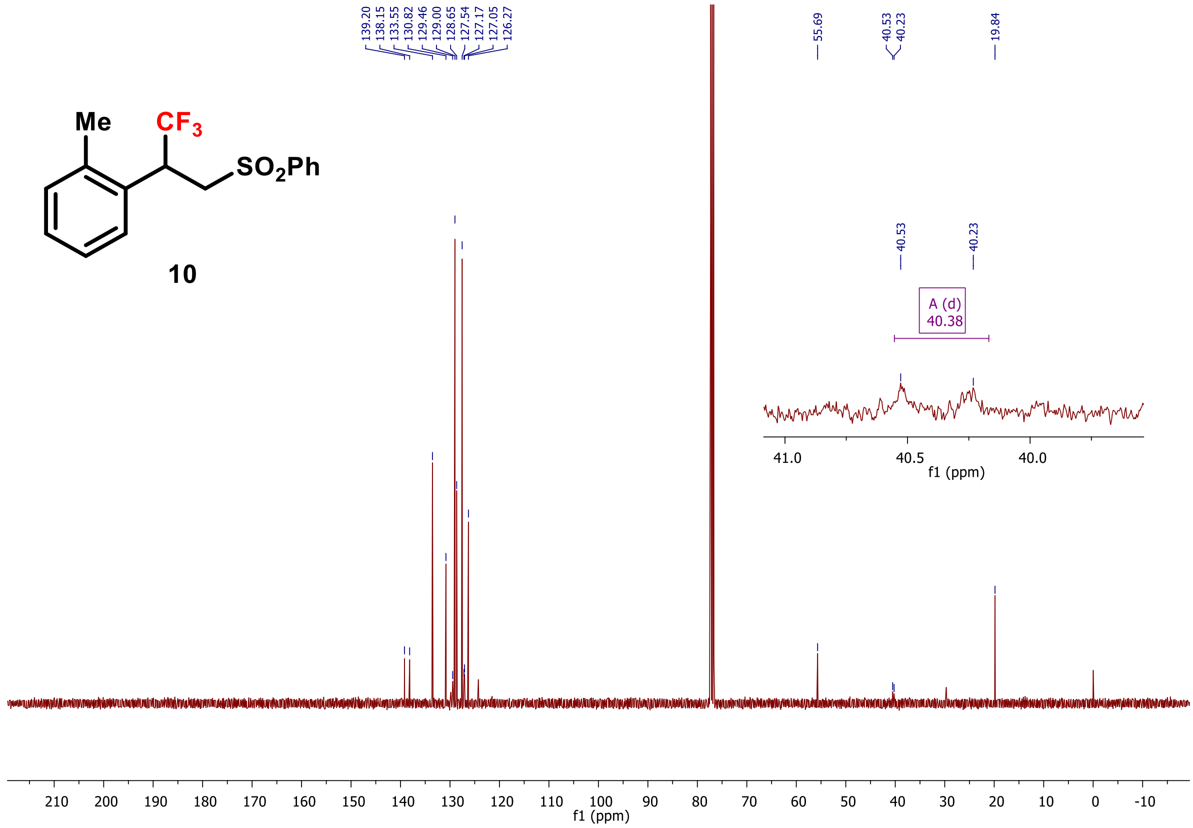

^{1}H NMR (400 MHz, $CDCl_3$) of **11**

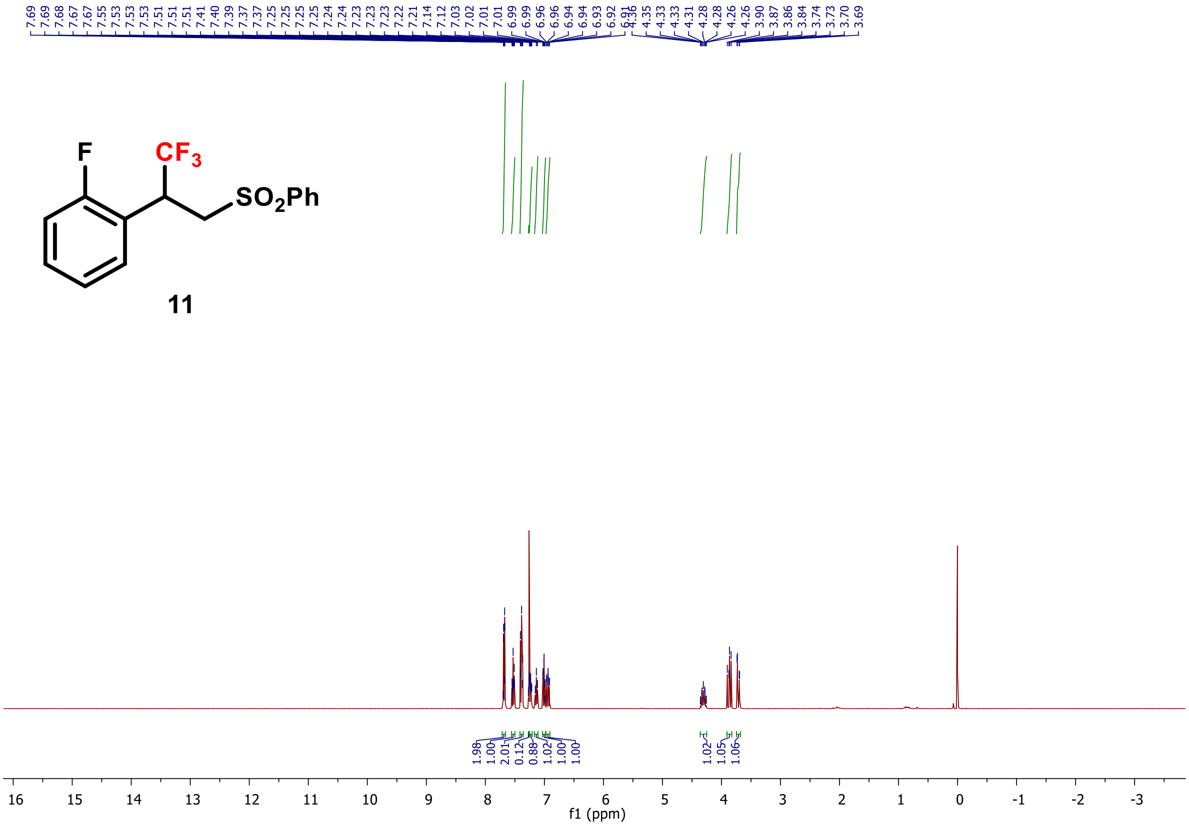

^{19}F NMR (376 MHz, $CDCl_3$) of **11**

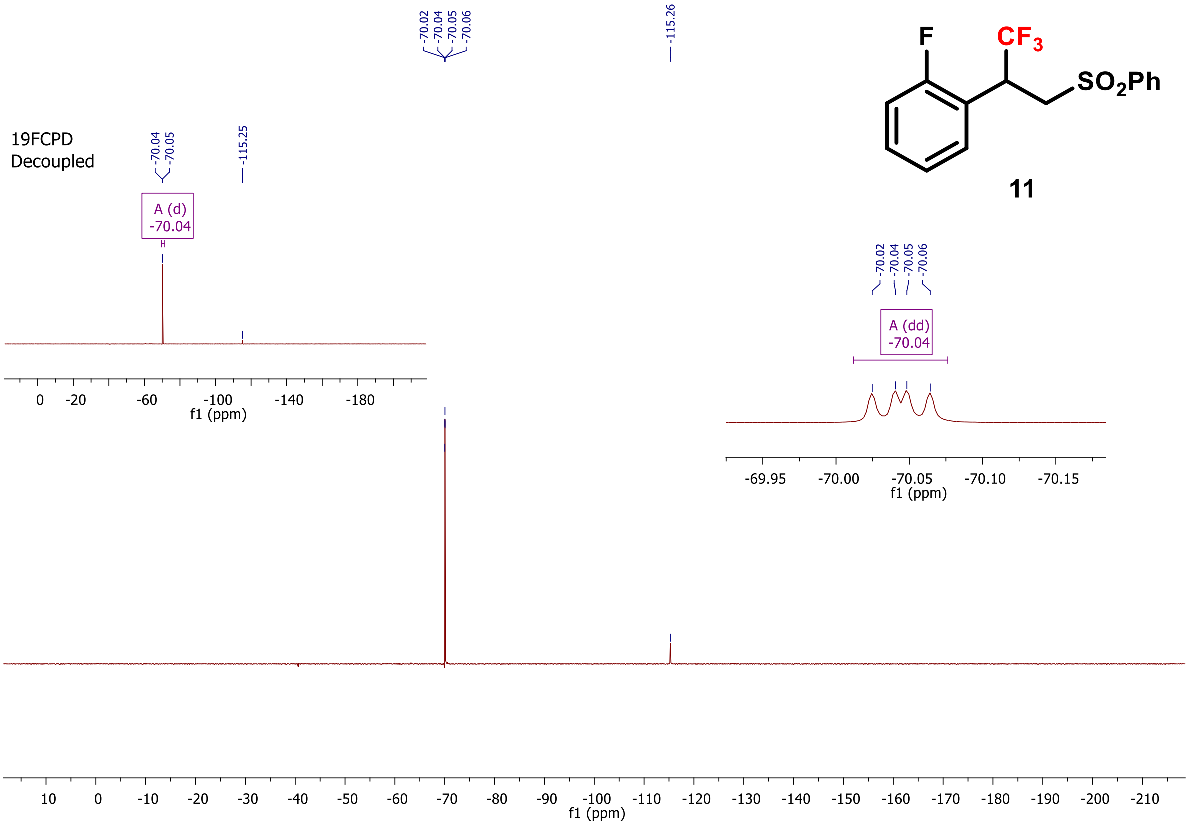

^{13}C NMR (101 MHz, $CDCl_3$) of **11**

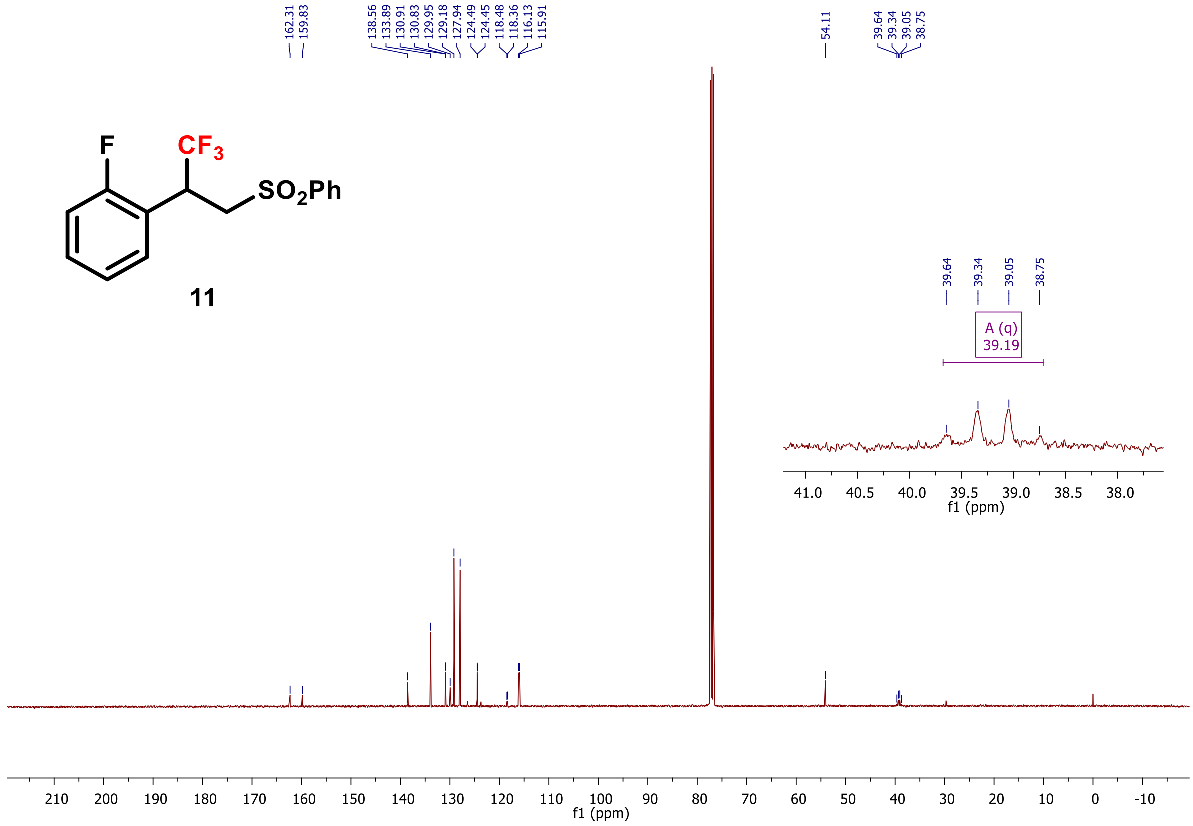

^{1}H NMR (400 MHz, $CDCl_3$) of **12**

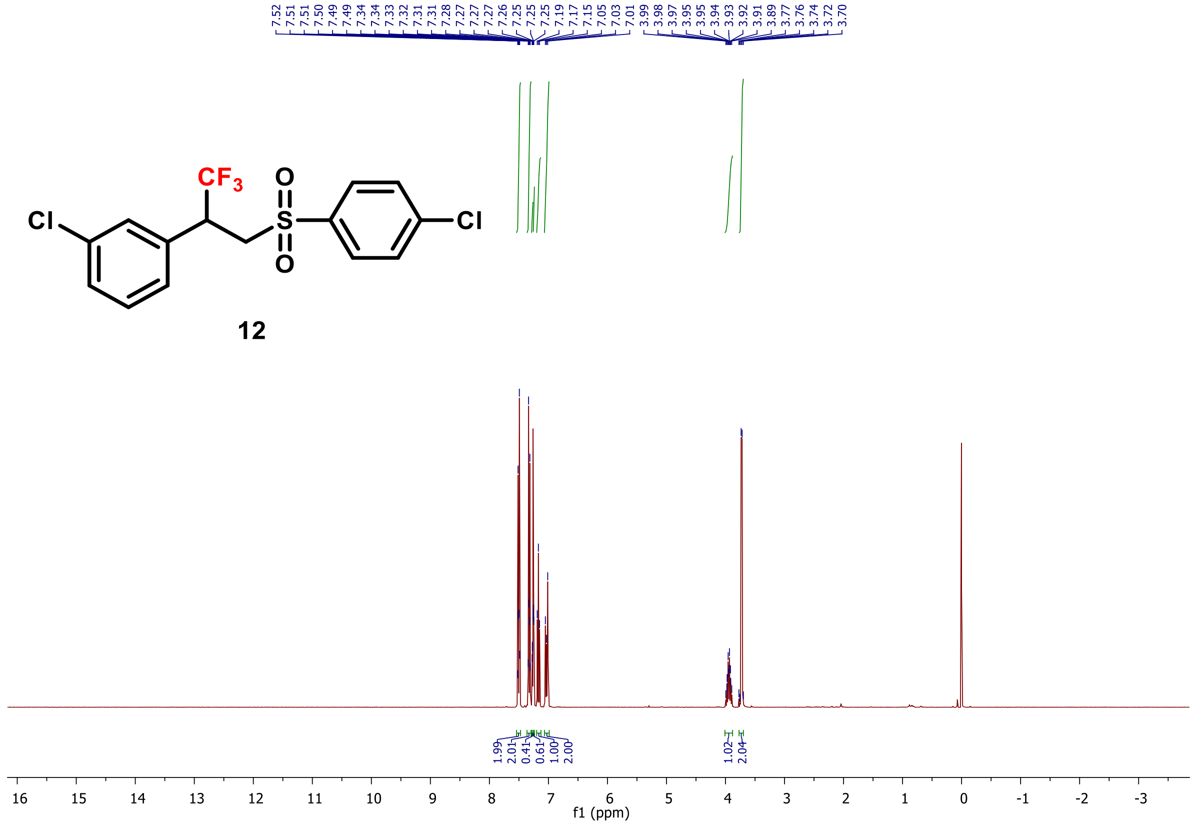

^{19}F NMR (376 MHz, $CDCl_3$) of **12**

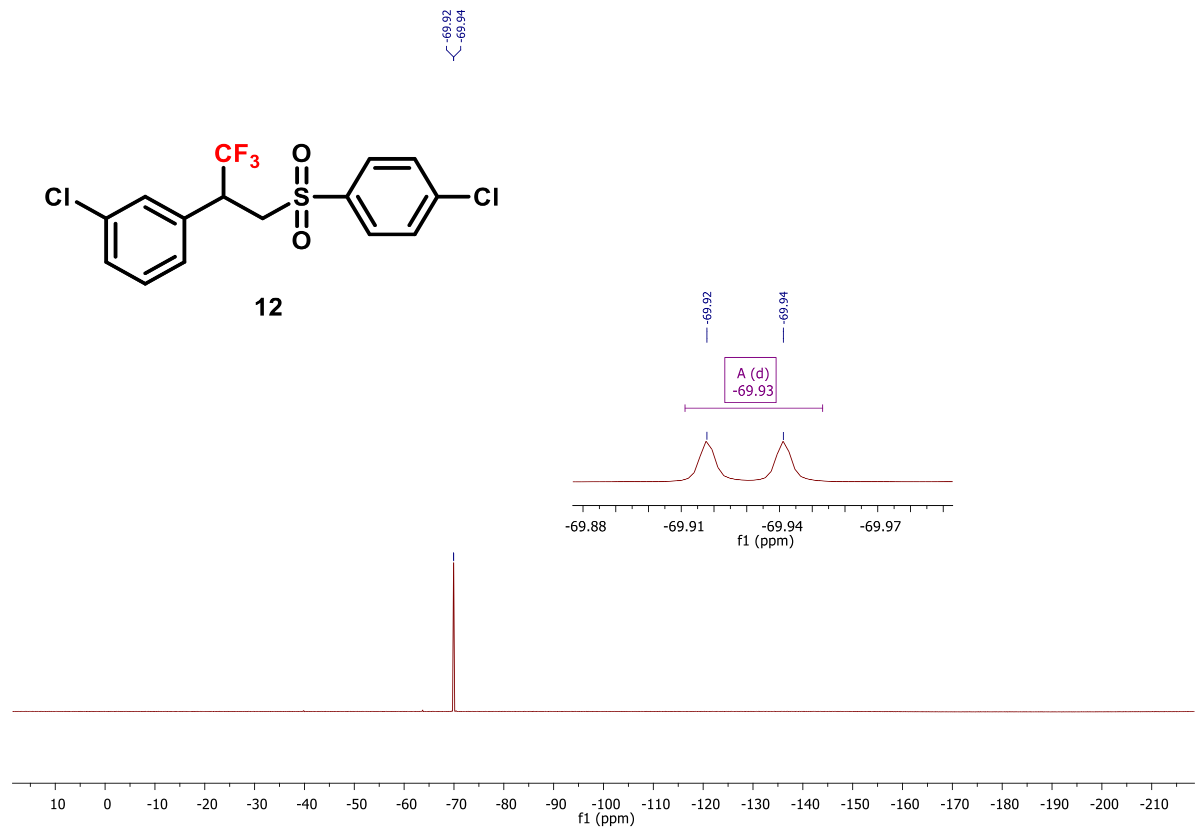

^{13}C NMR (101 MHz, $CDCl_3$) of **12**

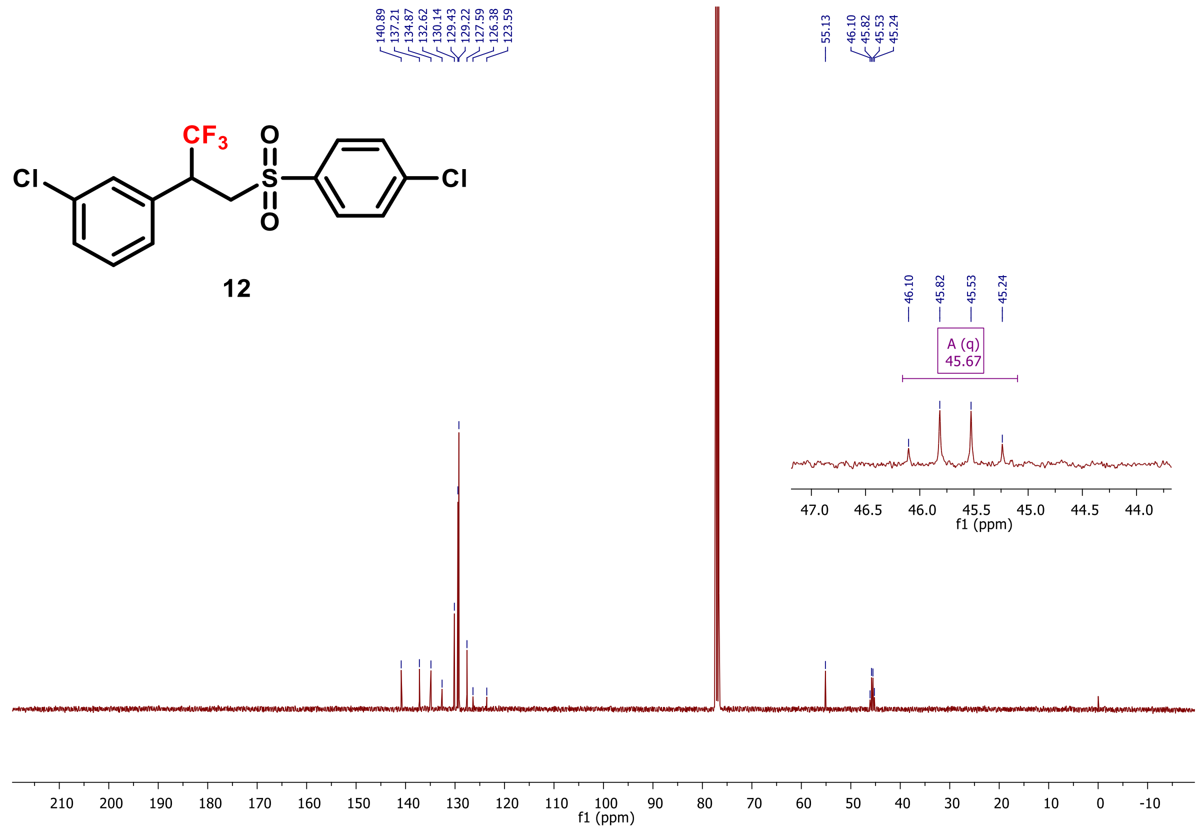

^{1}H NMR (400 MHz, $CDCl_3$) of **13**

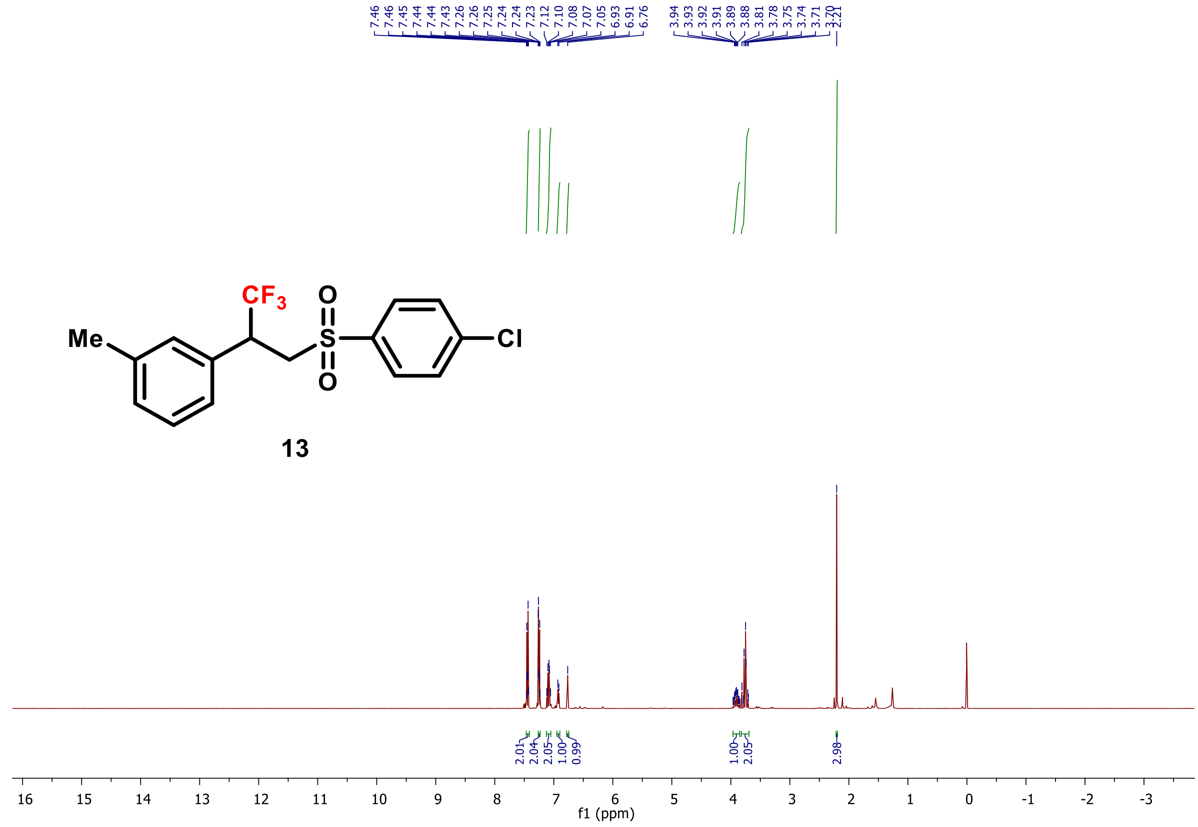

^{19}F NMR (376 MHz, $CDCl_3$) of **13**

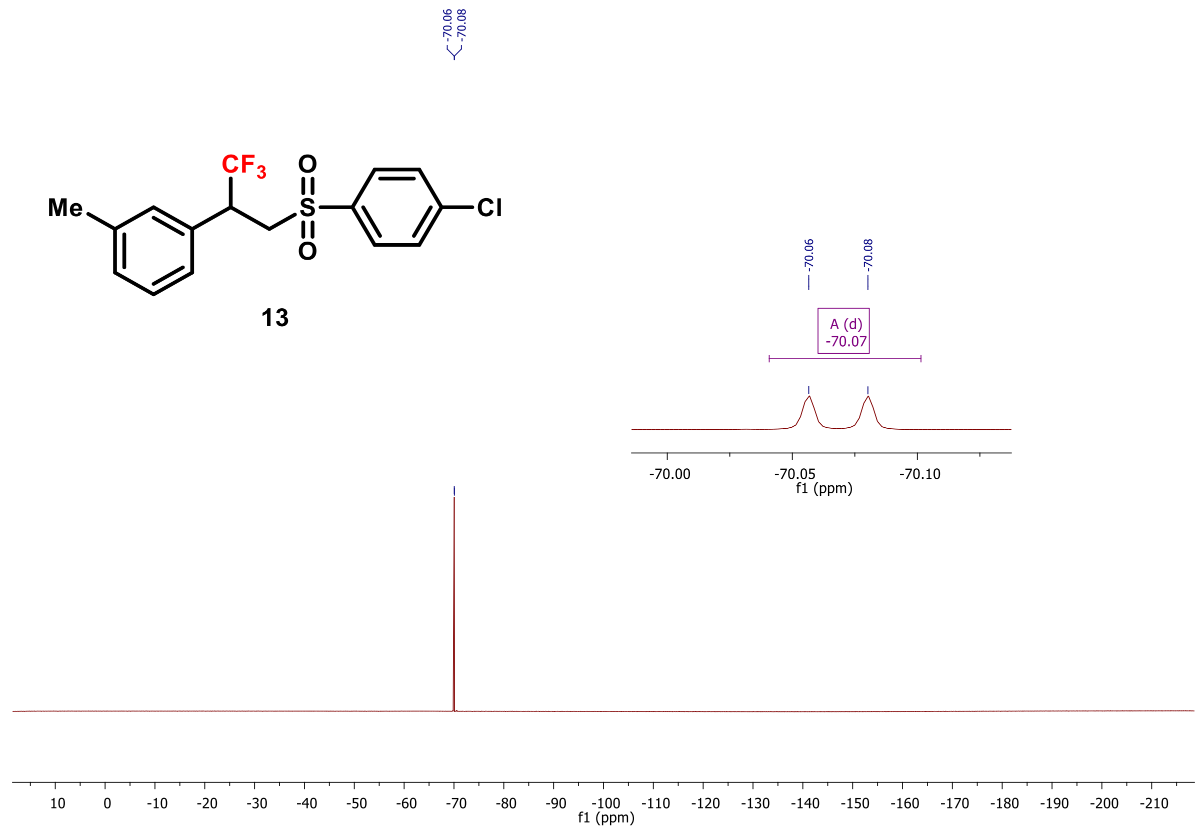

^{13}C NMR (101 MHz, $CDCl_3$) of **13**

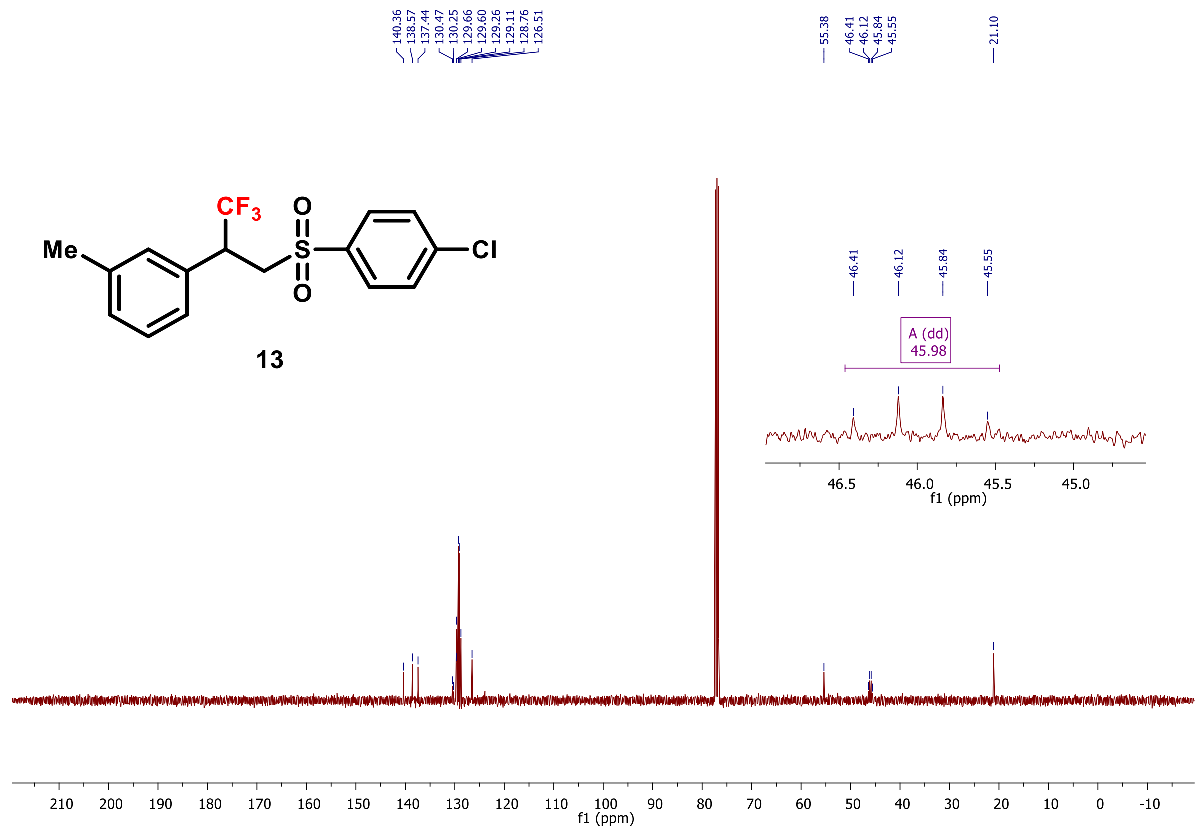

¹H NMR (400 MHz, $CDCl_3$) of **14**

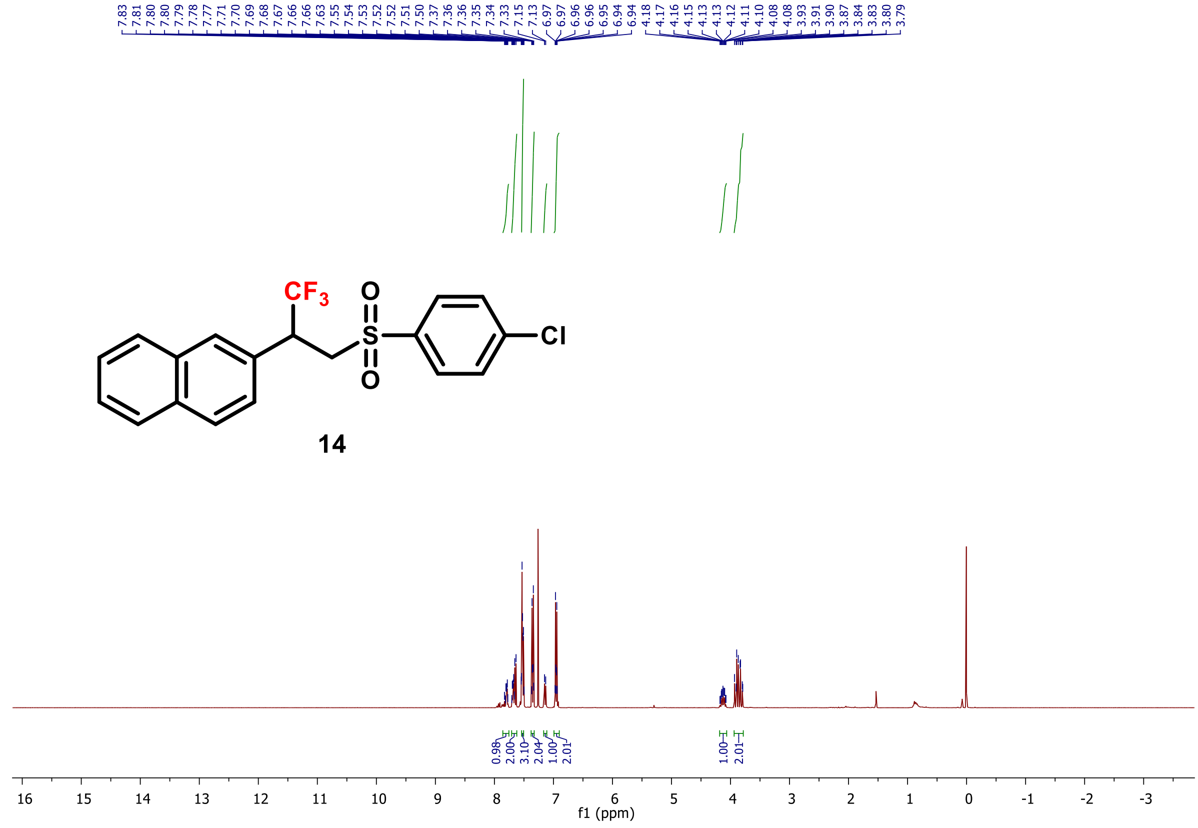

¹⁹F NMR (376 MHz, $CDCl_3$) of **14**

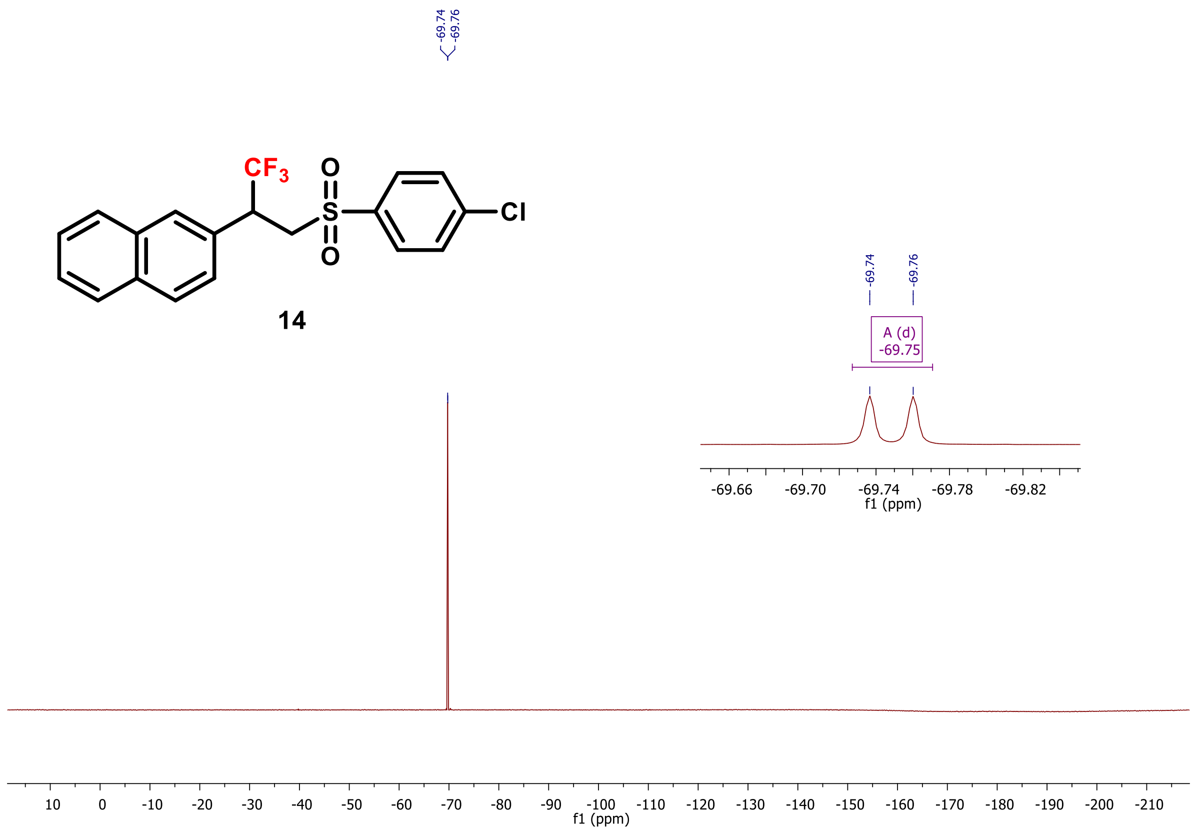

^{13}C NMR (101 MHz, $CDCl_3$) of **14**

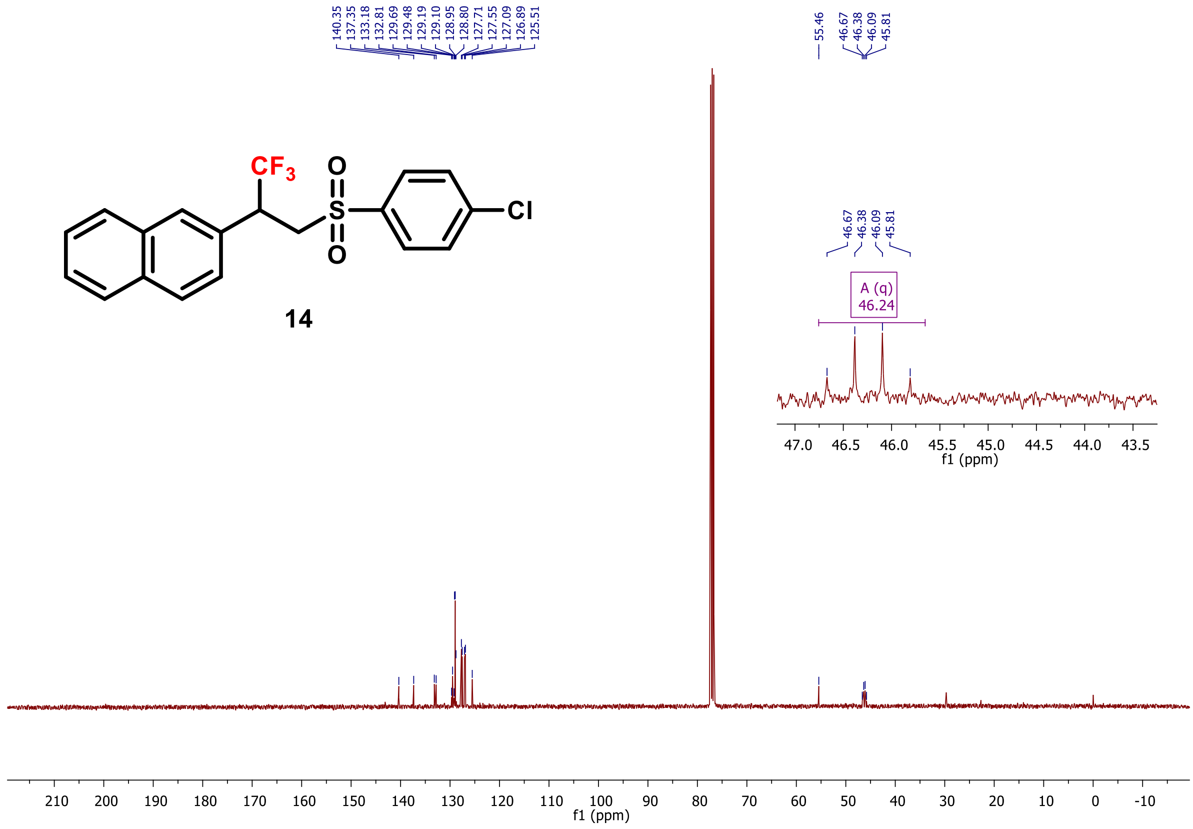

^{1}H NMR (400 MHz, $CDCl_3$) of **15**

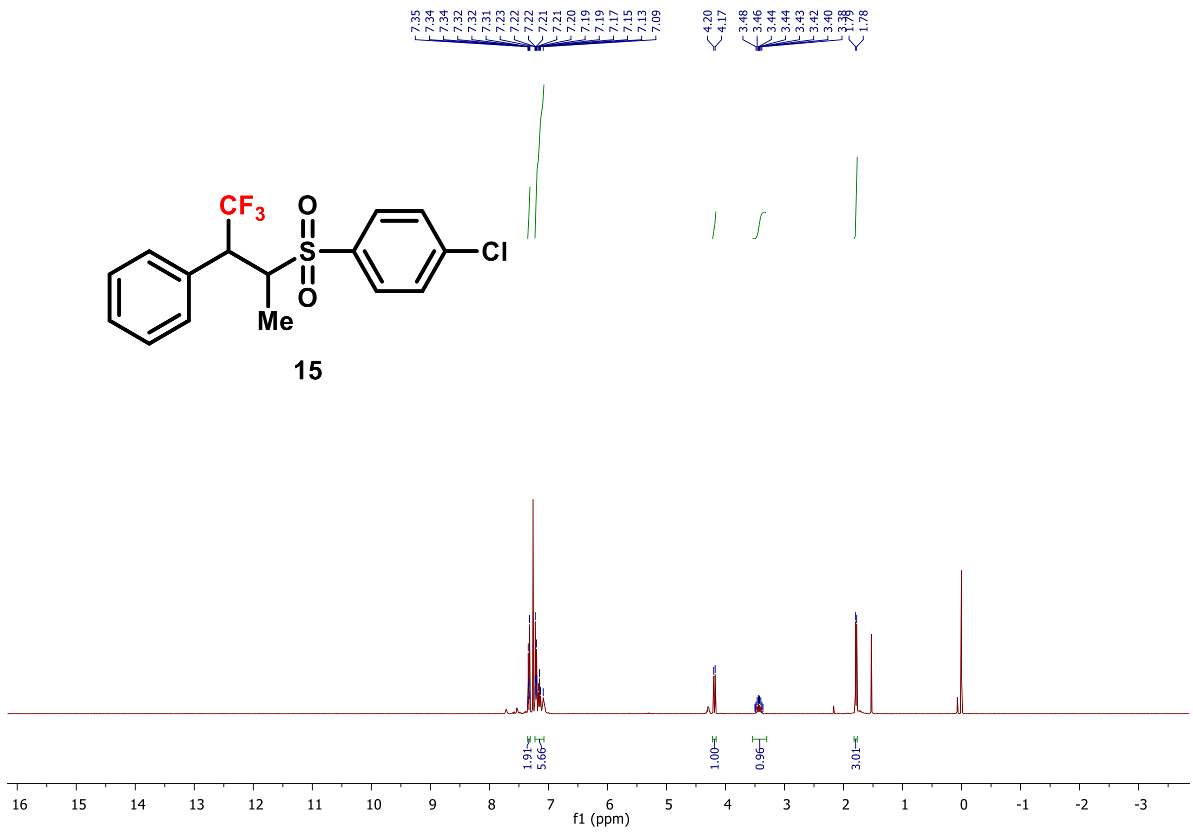

19F NMR (376 MHz, CDCl3) of **15**

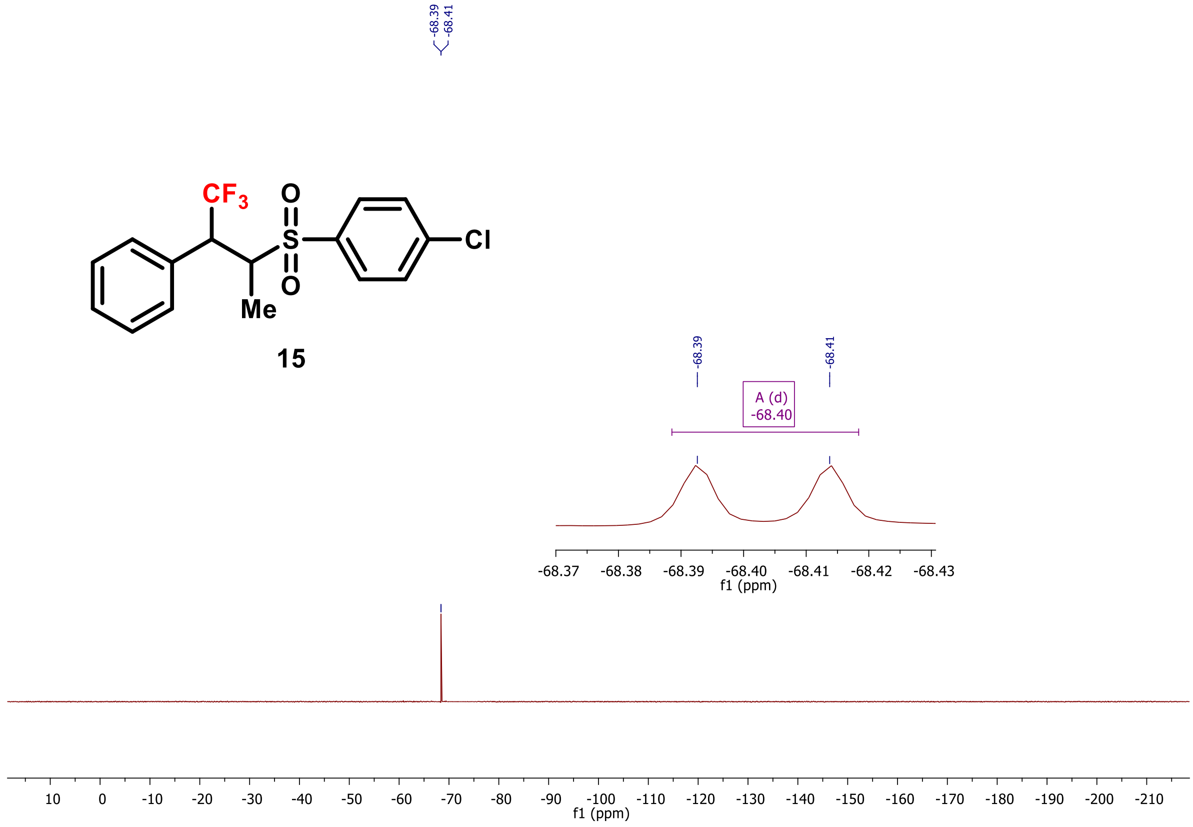

13C NMR (101 MHz, CDCl3) of **15**

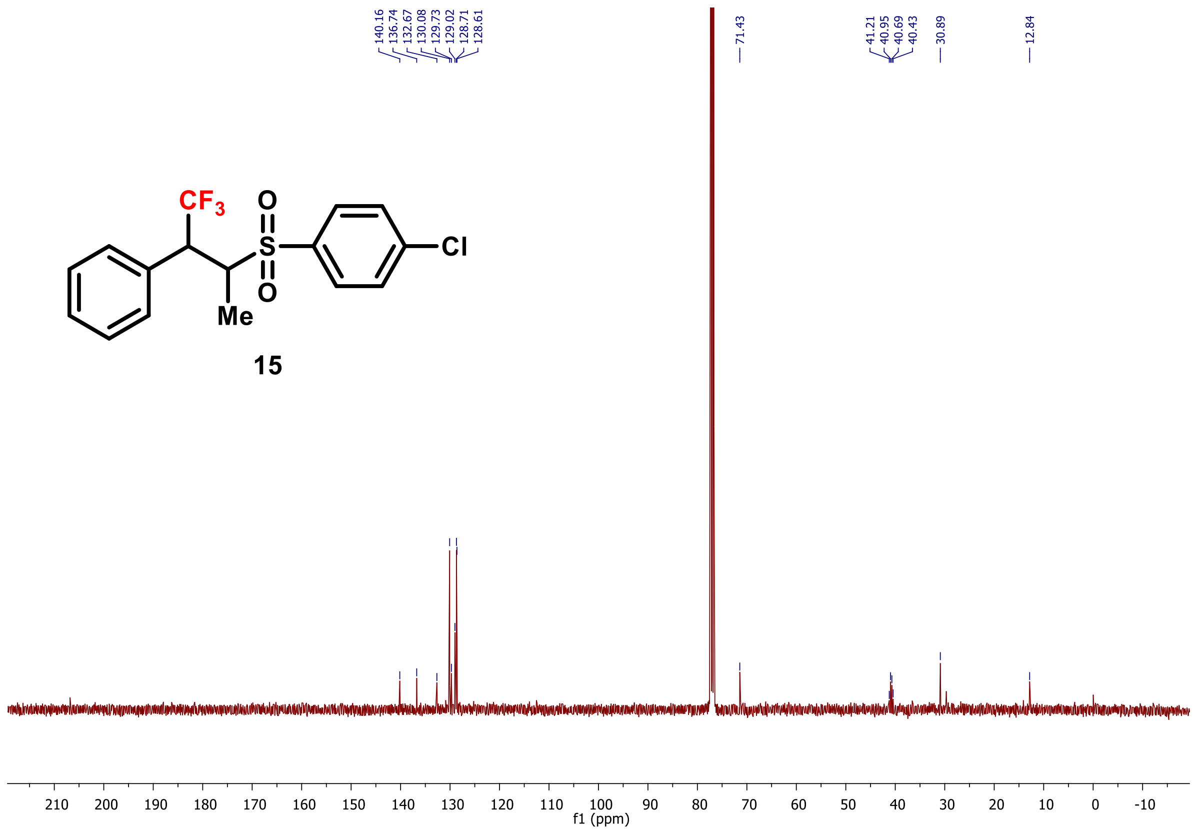

^{1}H NMR (400 MHz, $CDCl_3$) of **16**

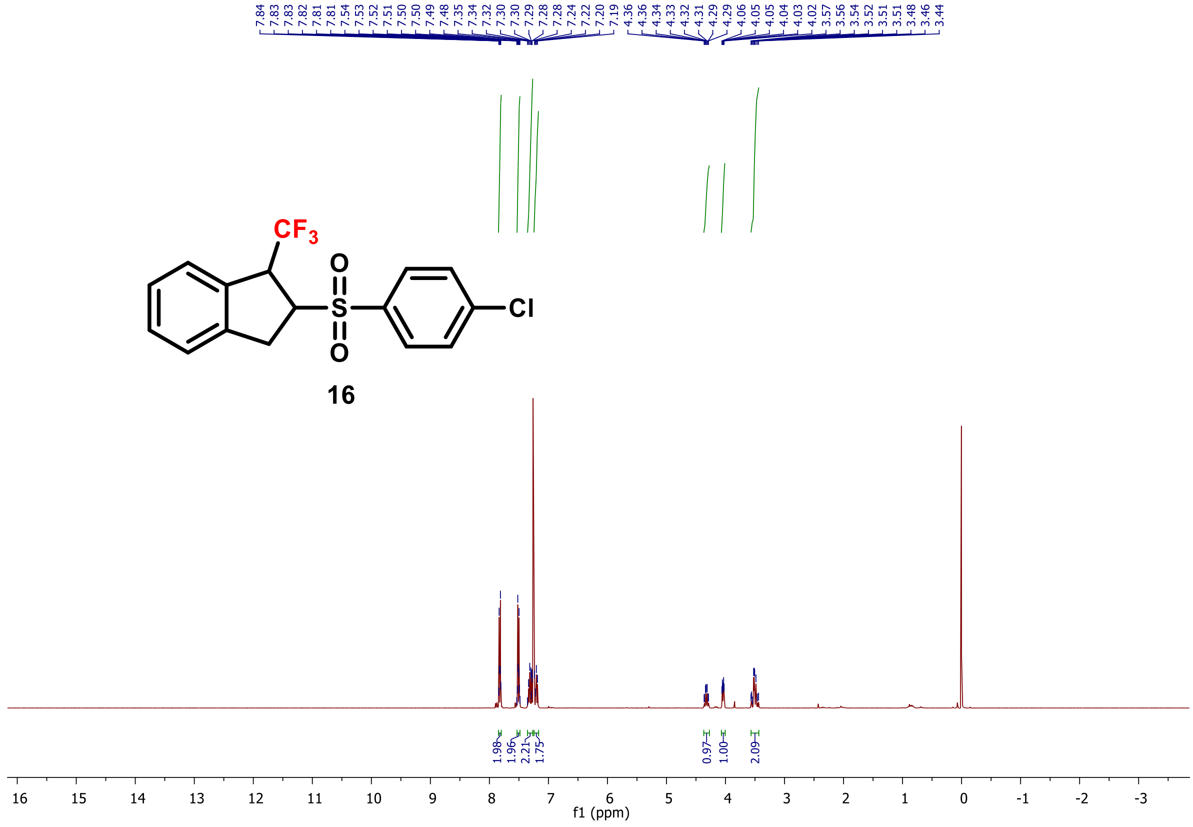

^{19}F NMR (376 MHz, $CDCl_3$) of **16**

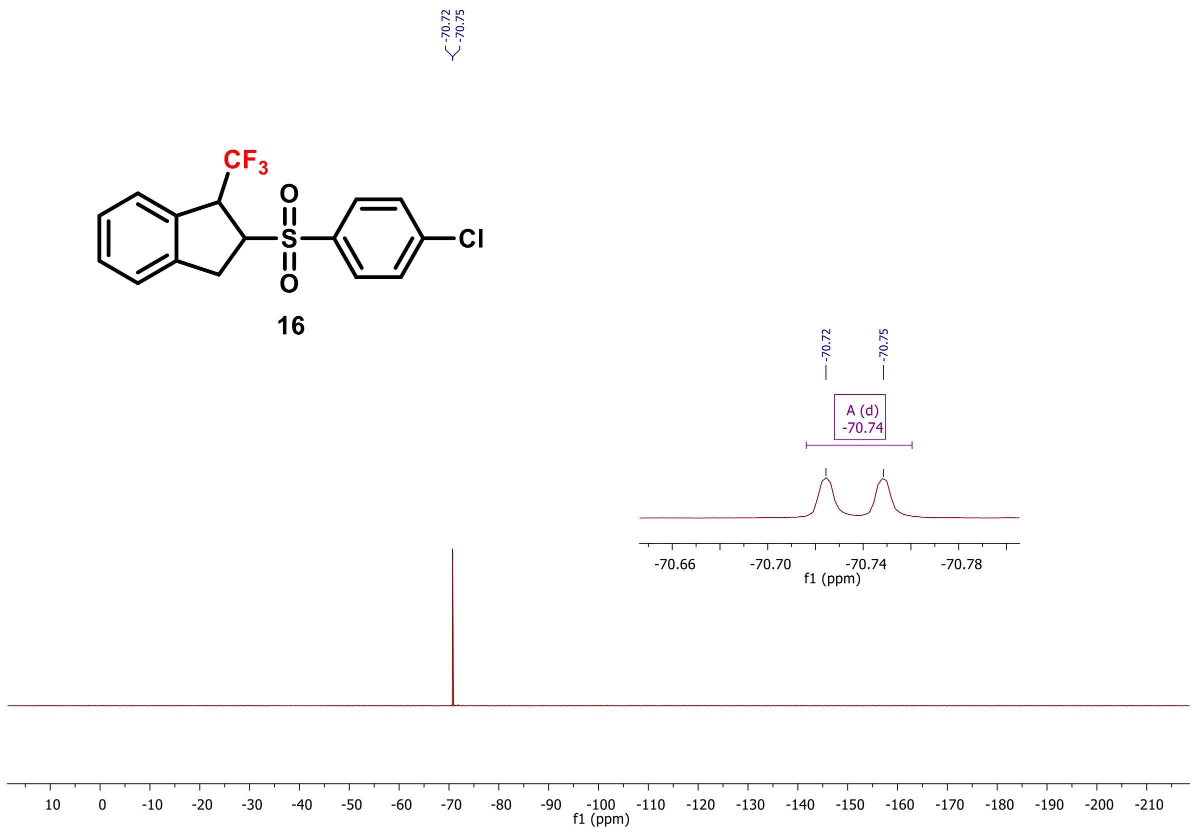

^{13}C NMR (101 MHz, $CDCl_3$) of **16**

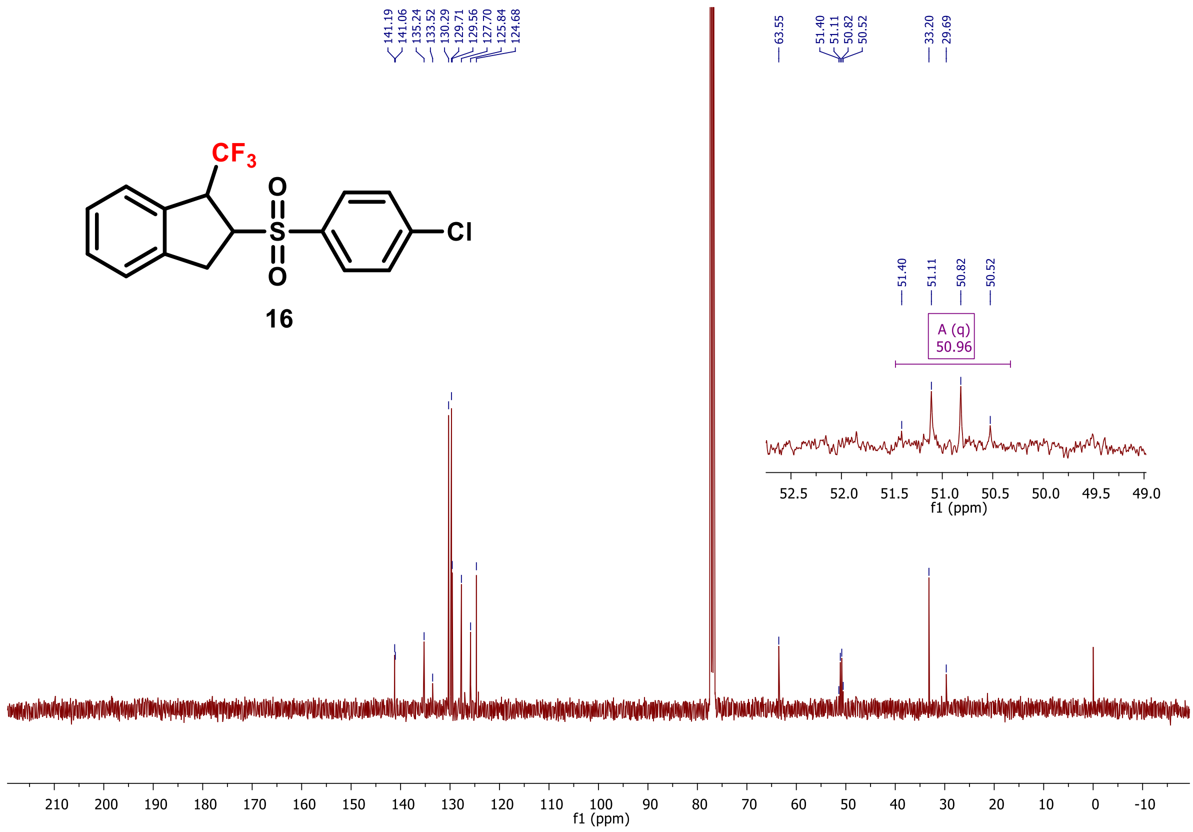

^{1}H NMR (400 MHz, $CDCl_3$) of **17**

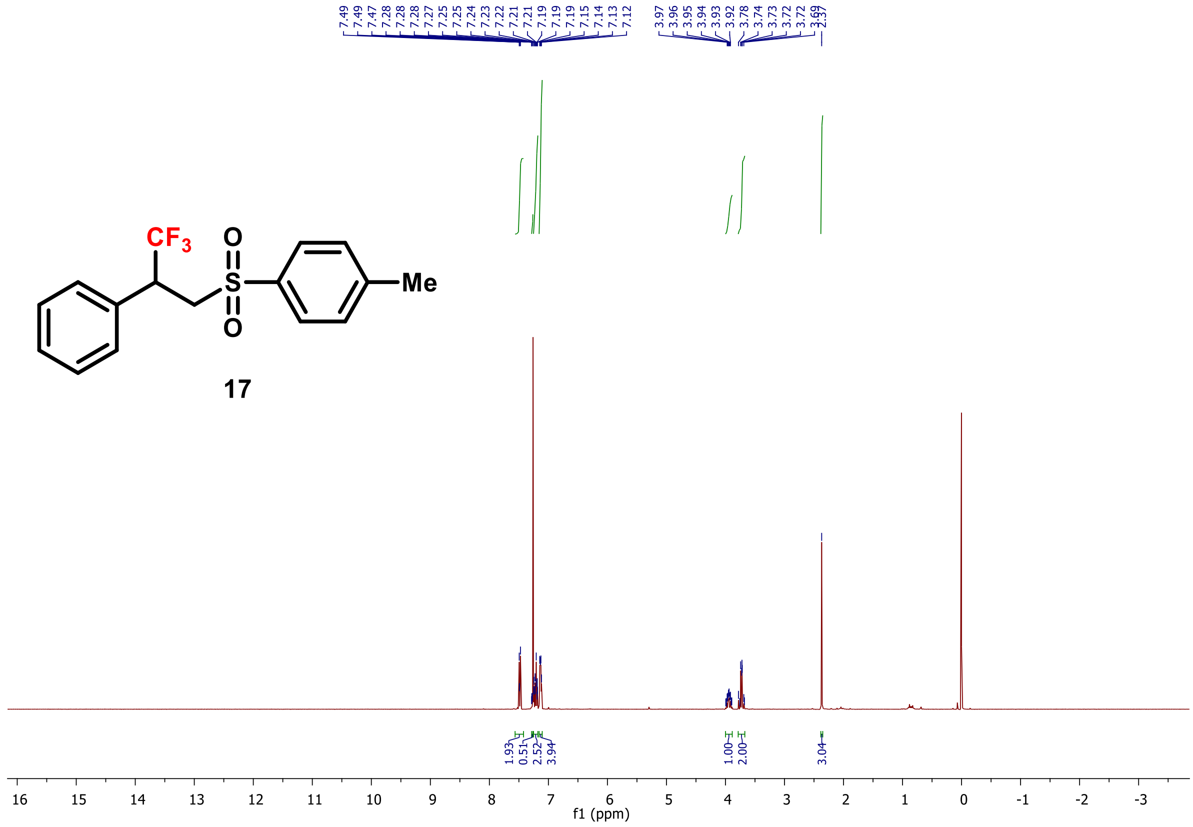

^{19}**F NMR** (376 MHz, $CDCl_3$) of **17**

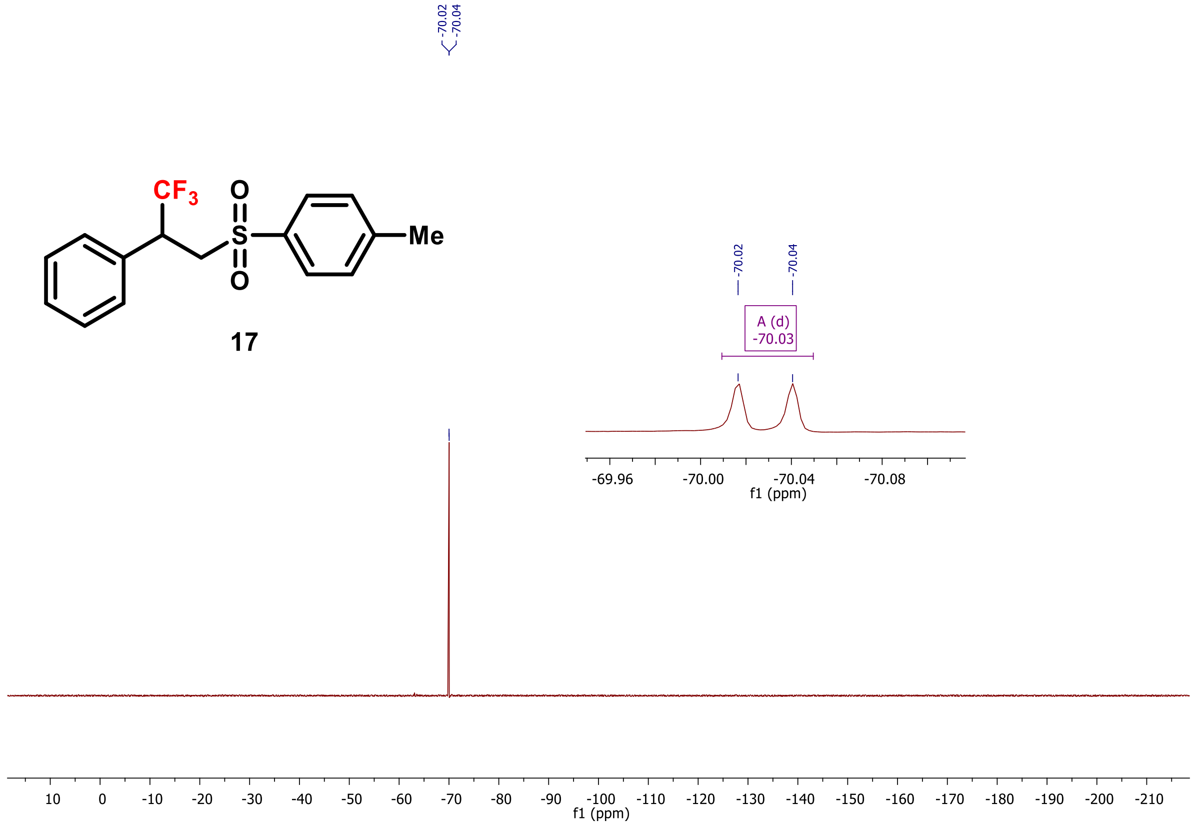

^{13}**C NMR** (101 MHz, $CDCl_3$) of **17**

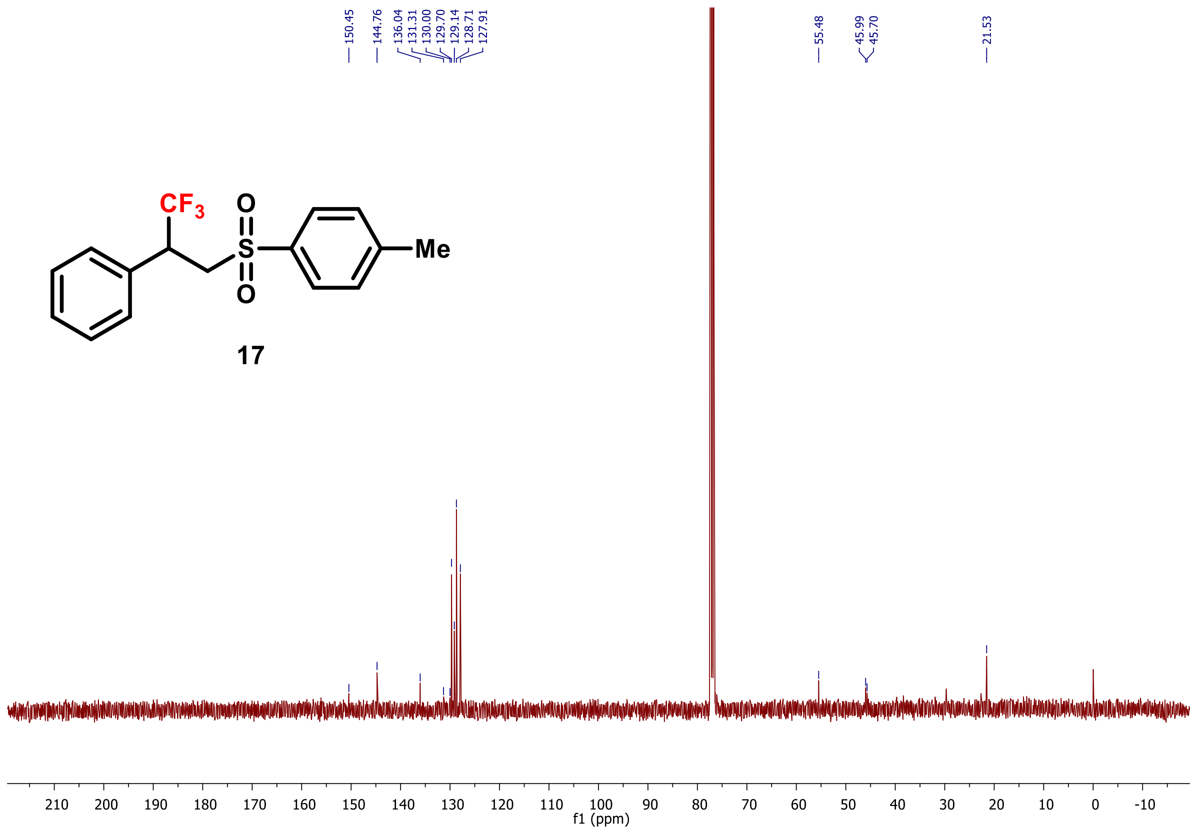

^{1}H NMR (400 MHz, $CDCl_3$) of **18**

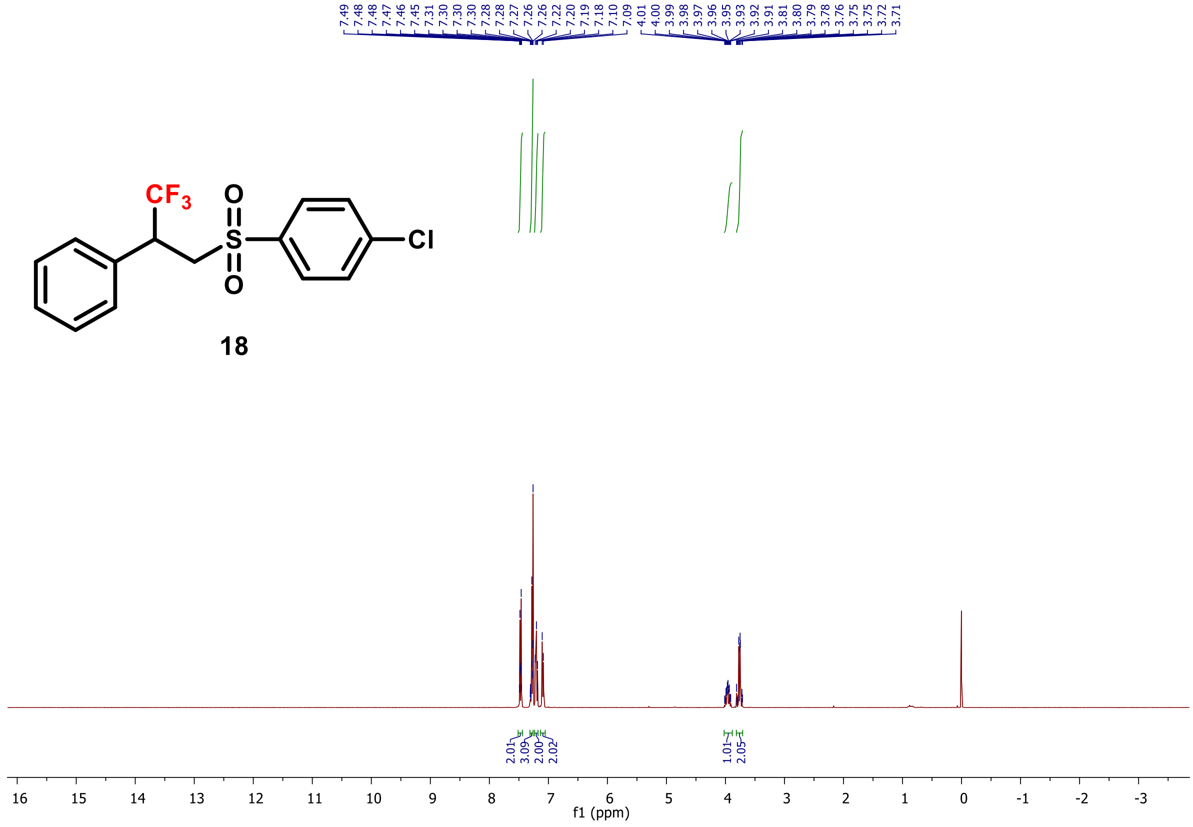

^{19}F NMR (376 MHz, $CDCl_3$) of **18**

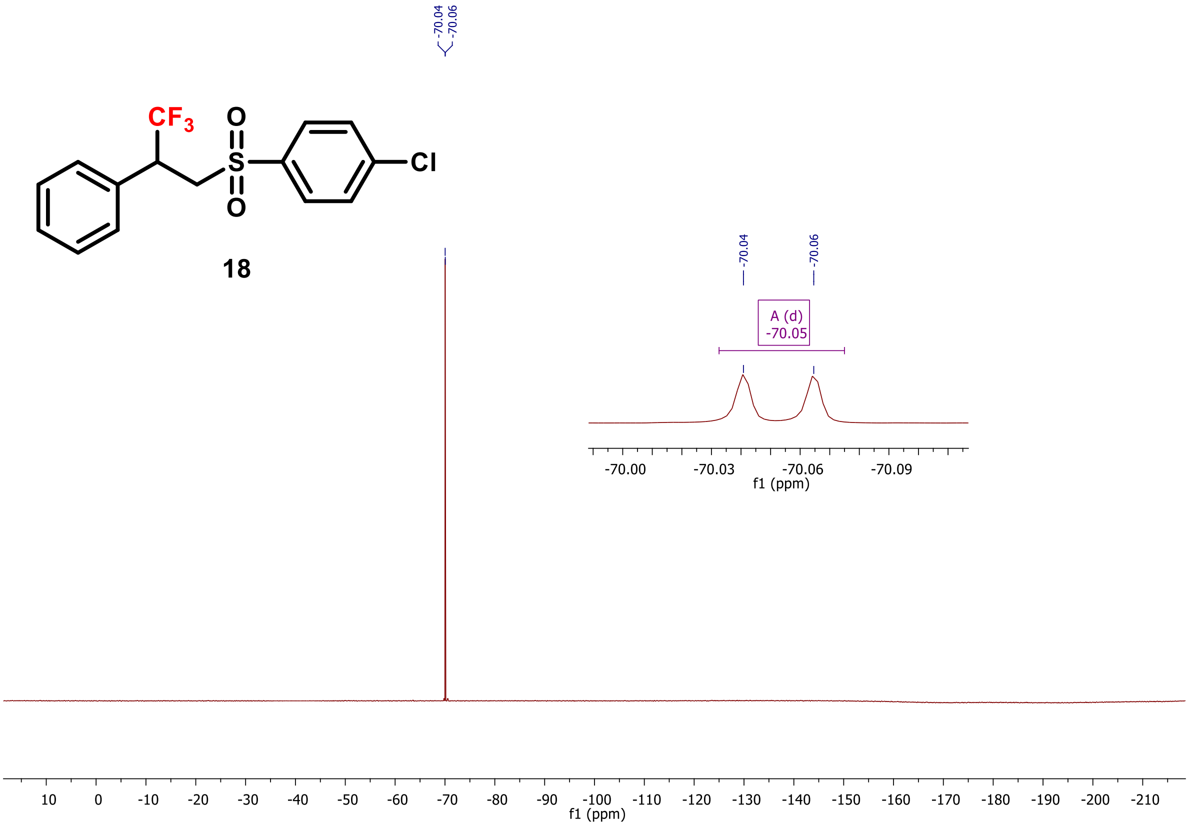

^{13}C NMR (101 MHz, $CDCl_3$) of **18**

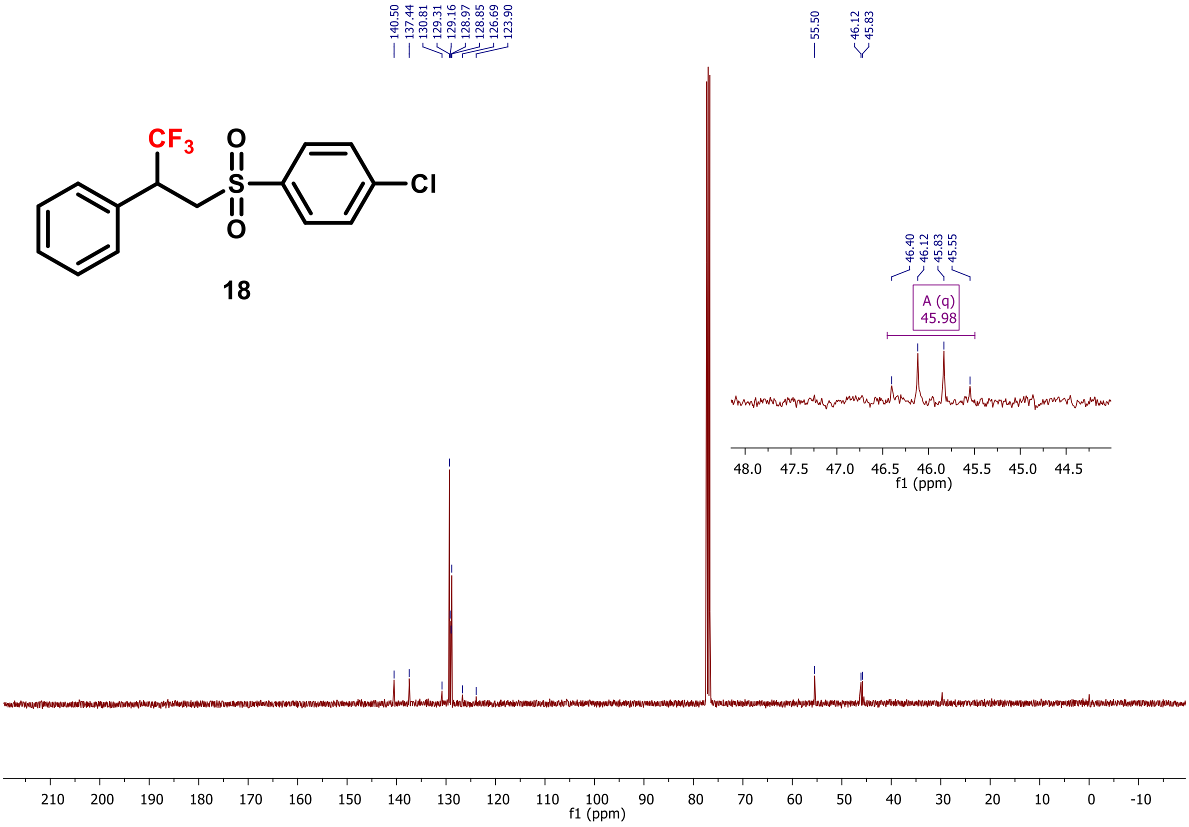

^{1}H NMR (400 MHz, $CDCl_3$) of **19**

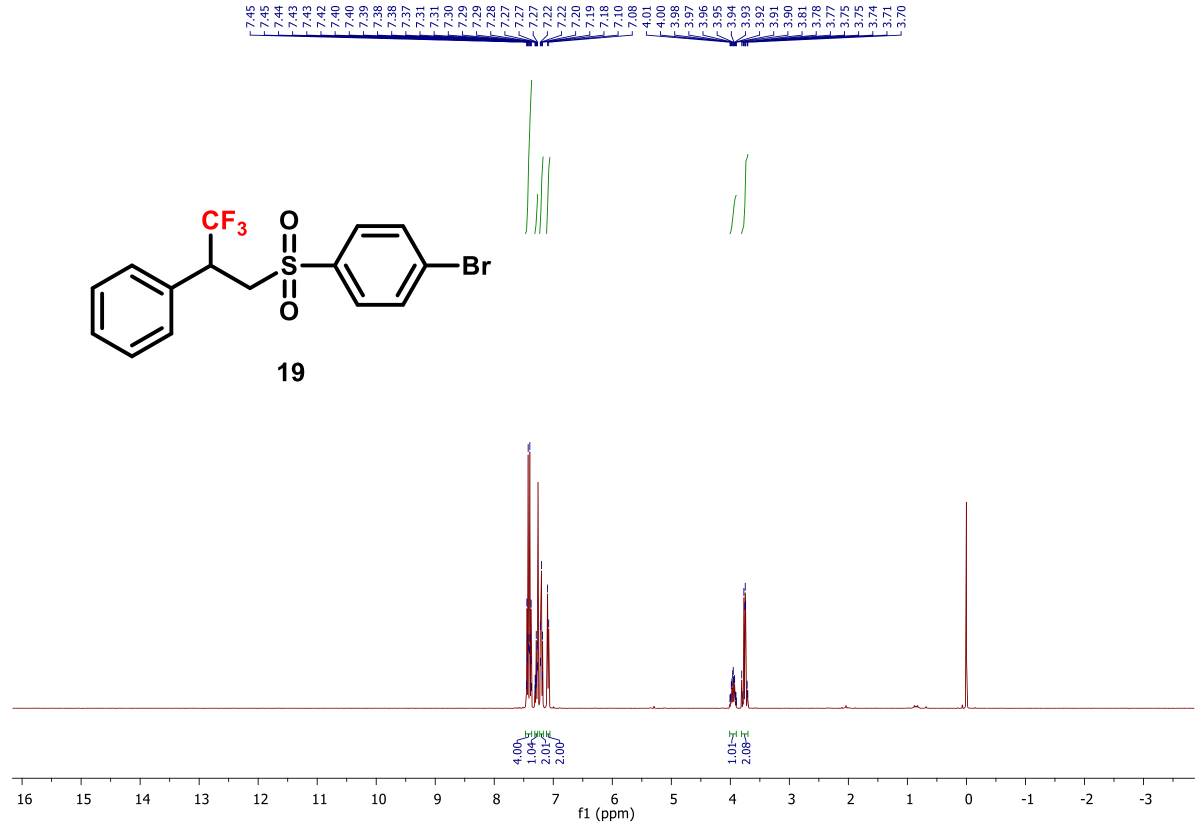

^{19}F NMR (376 MHz, $CDCl_3$) of **19**

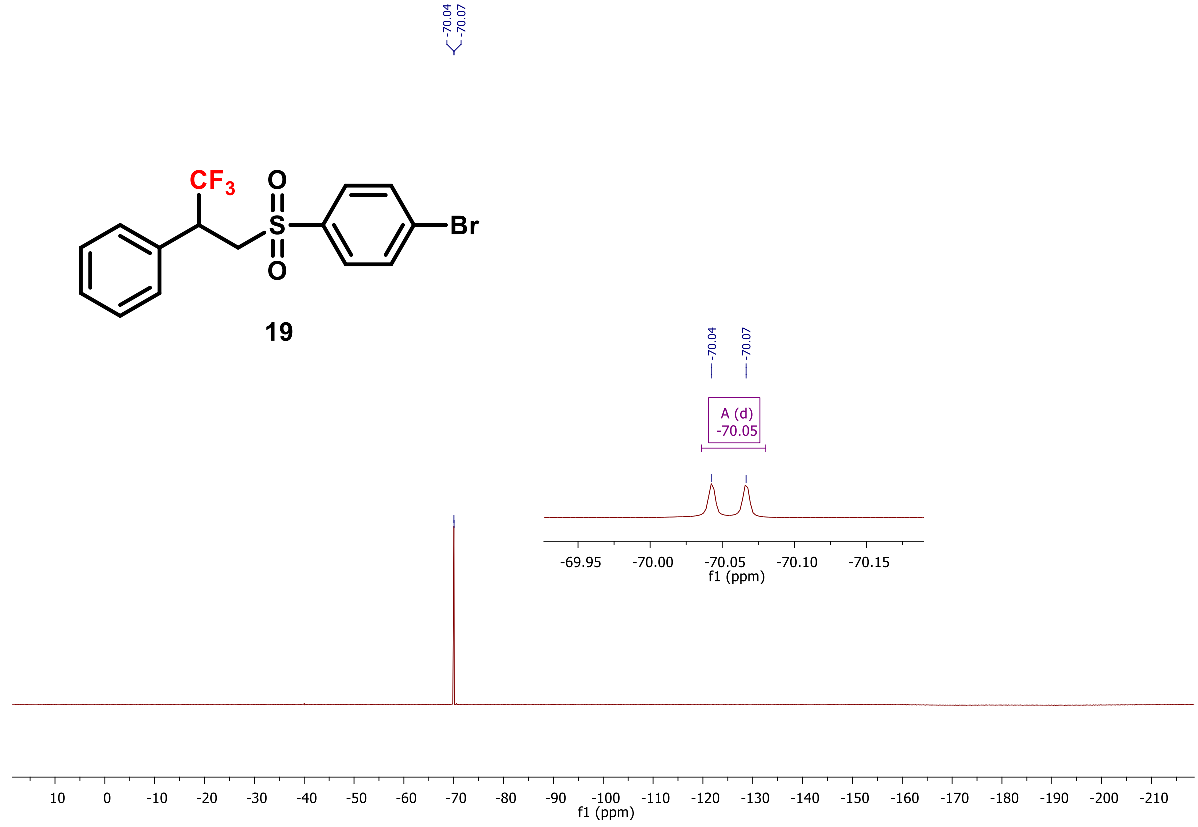

^{13}C NMR (101 MHz, $CDCl_3$) of **19**

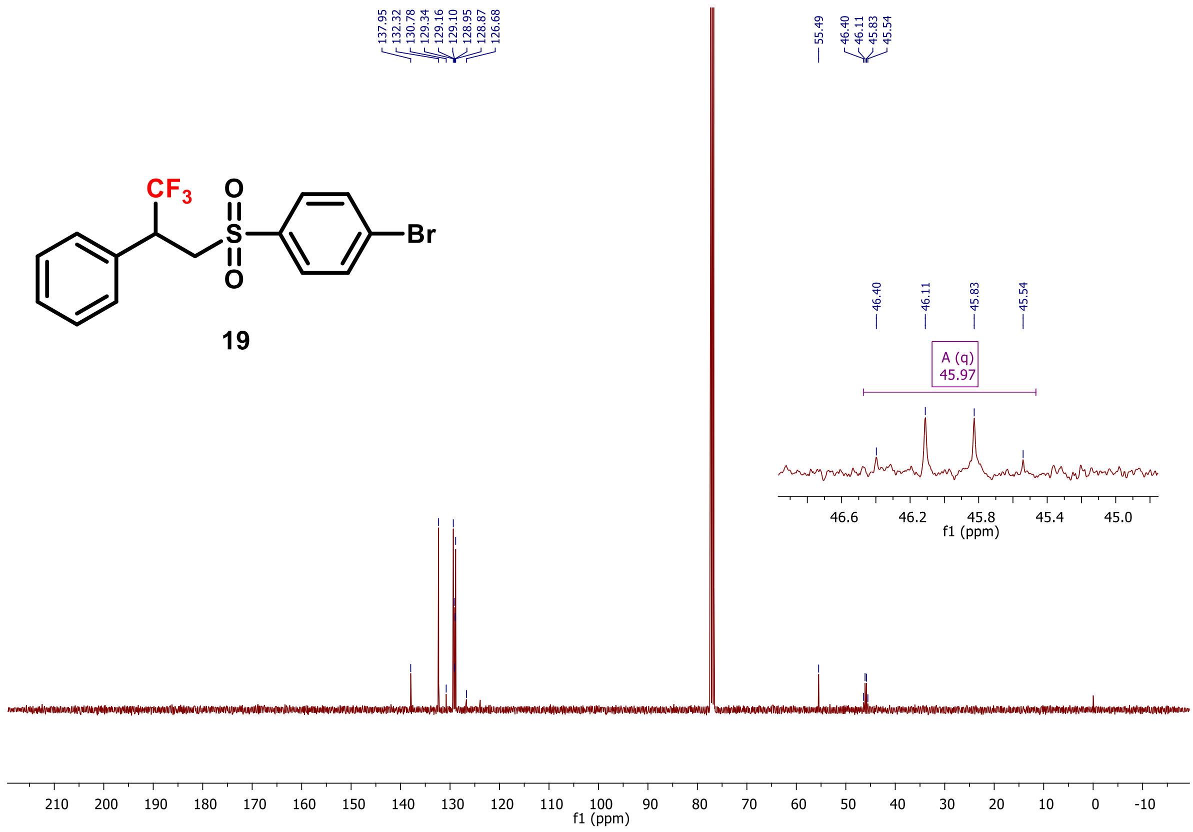

^{1}H NMR (400 MHz, $CDCl_3$) of **20**

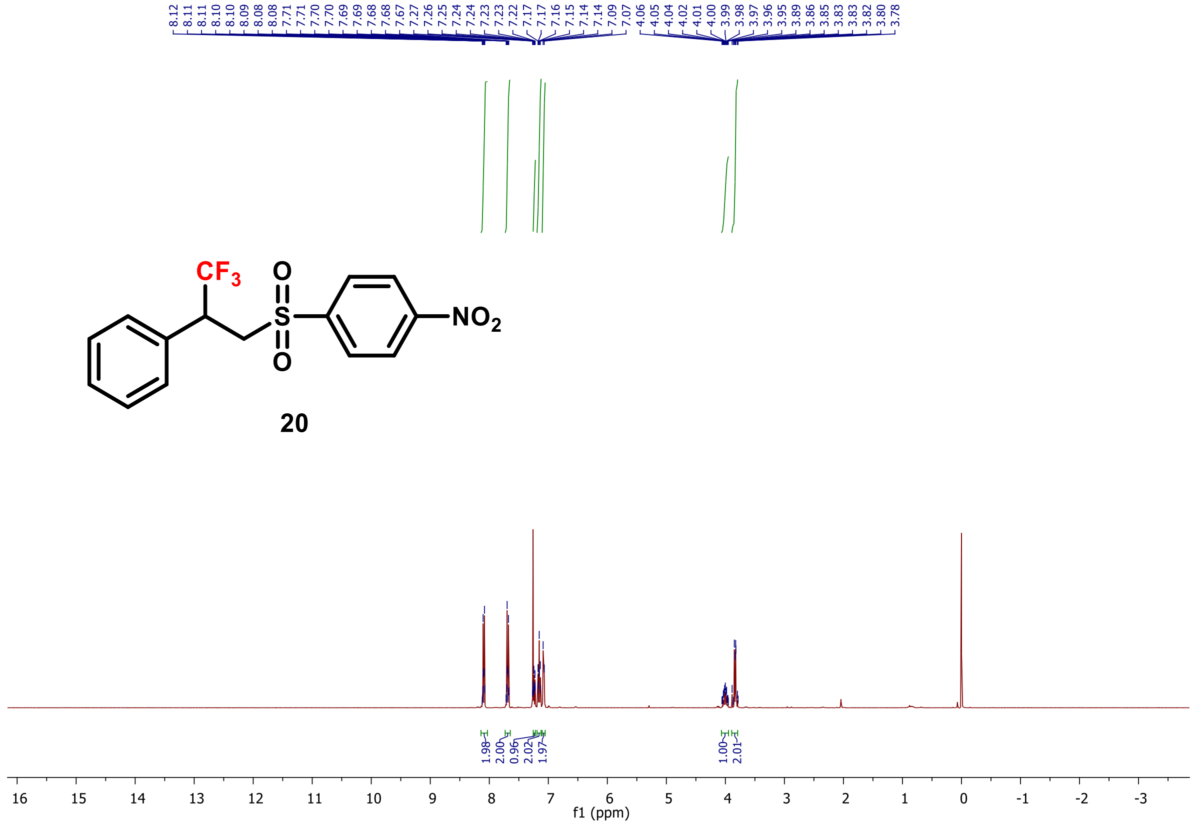

^{19}F NMR (376 MHz, $CDCl_3$) of **20**

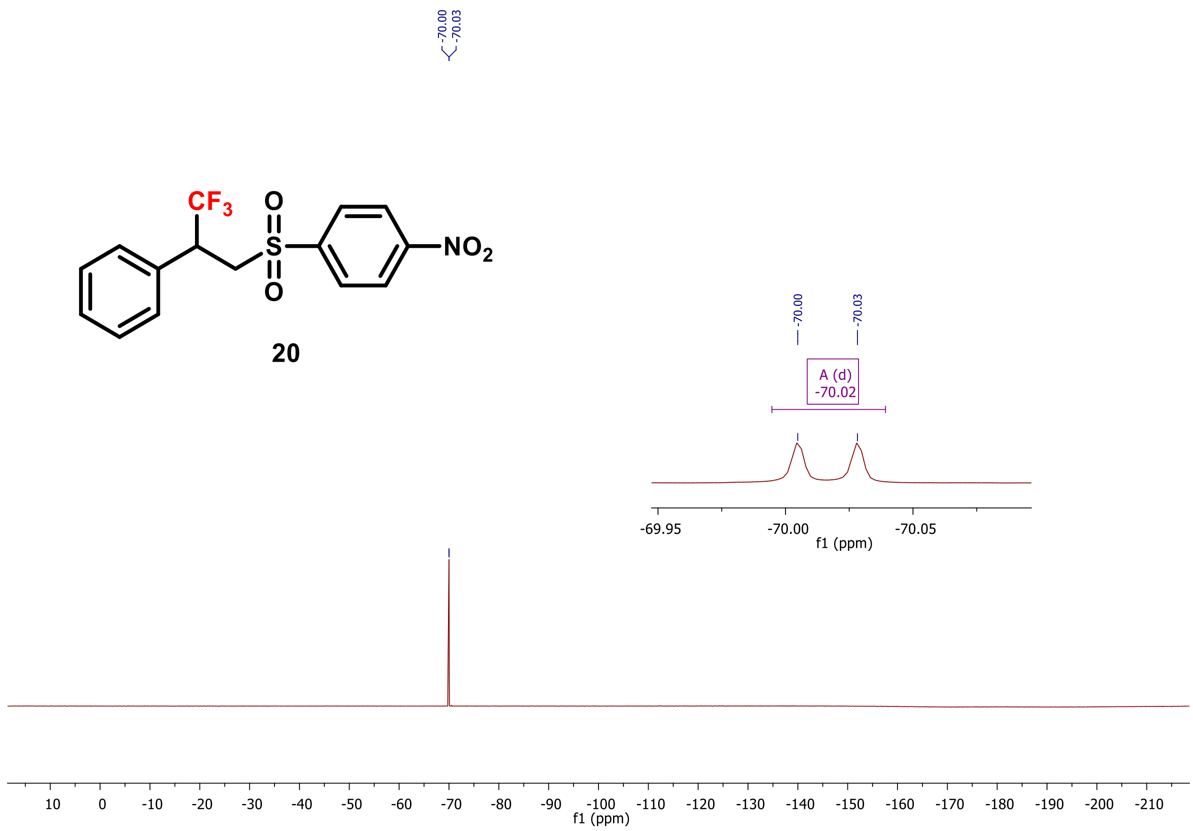

^{13}C NMR (101 MHz, $CDCl_3$) of **20**

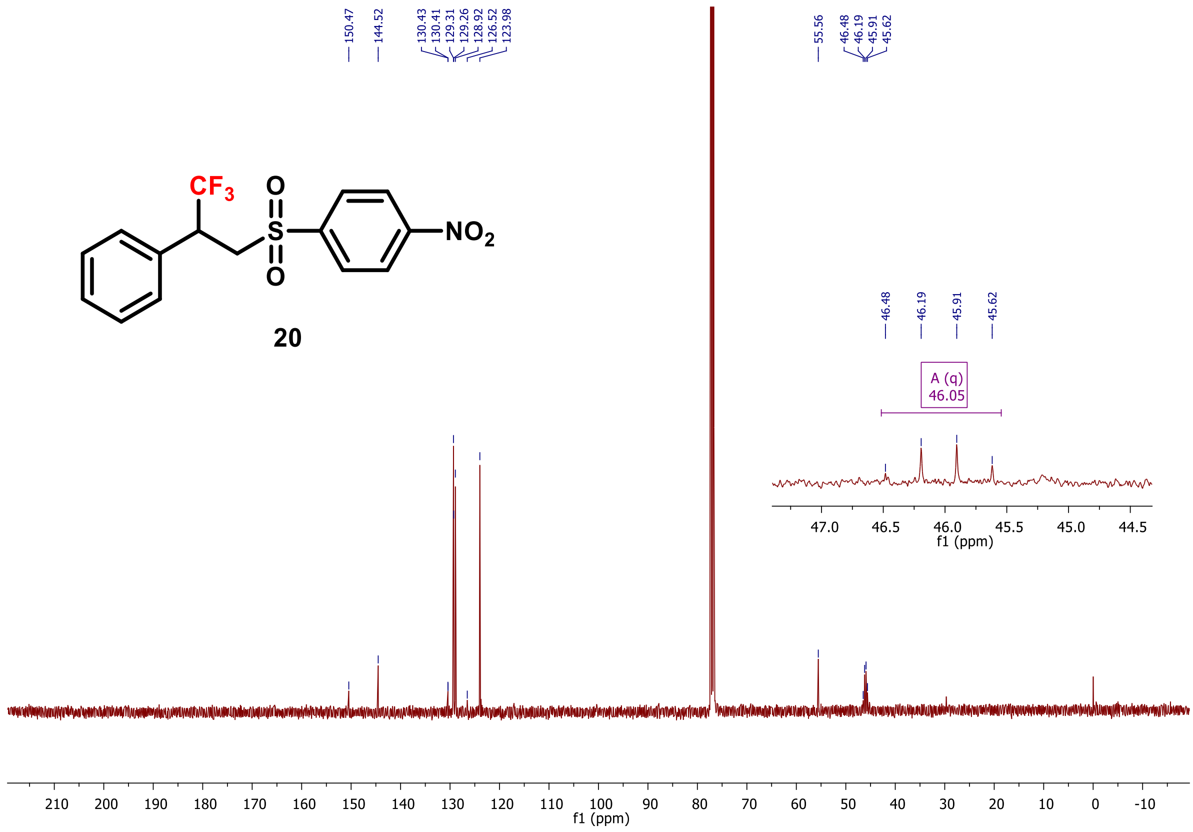

^{1}H NMR (400 MHz, $CDCl_3$) of **21**

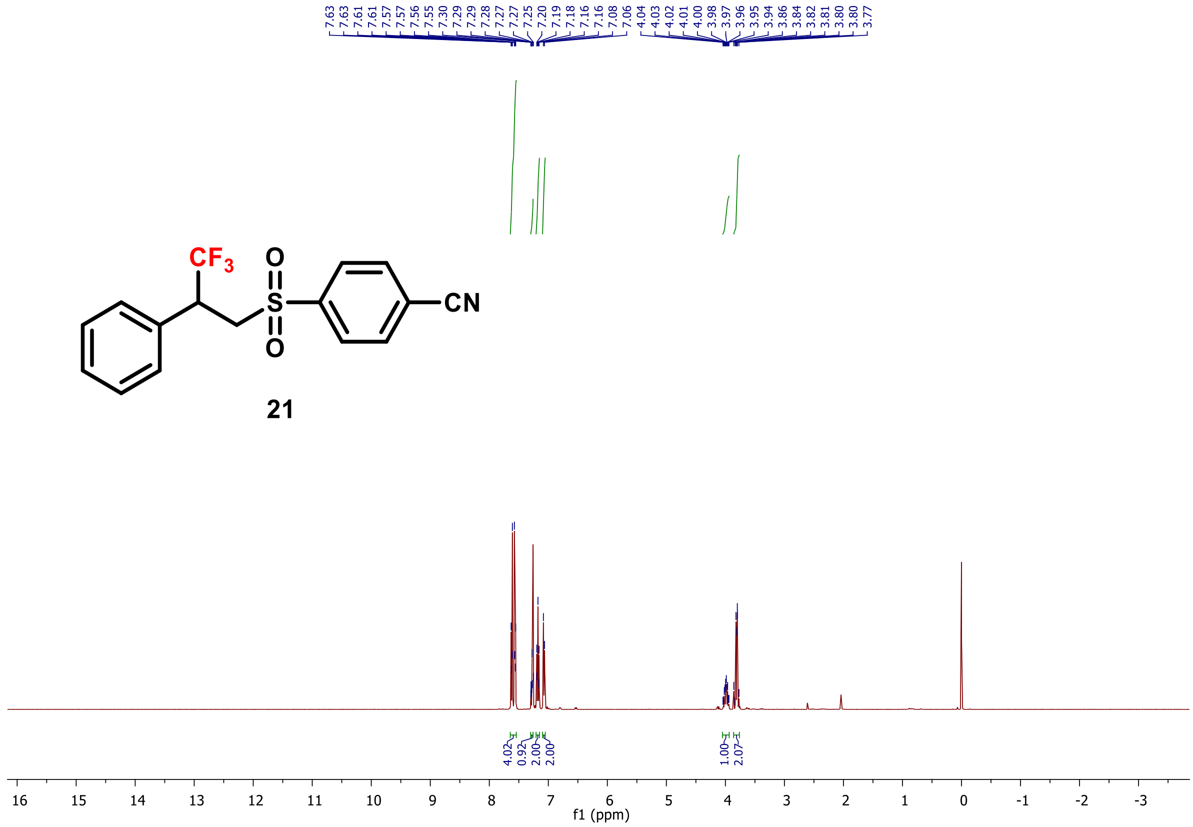

^{19}F NMR (376 MHz, $CDCl_3$) of **21**

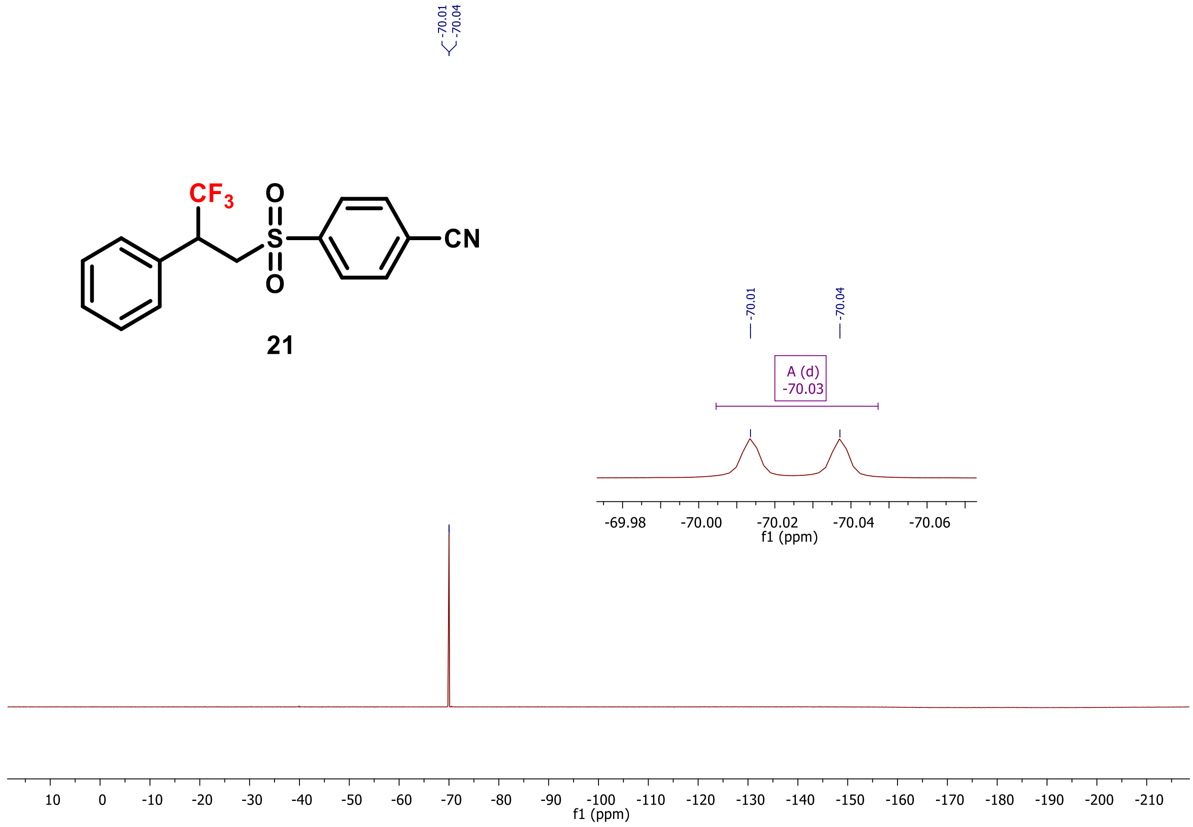

^{13}C NMR (101 MHz, $CDCl_3$) of **21**

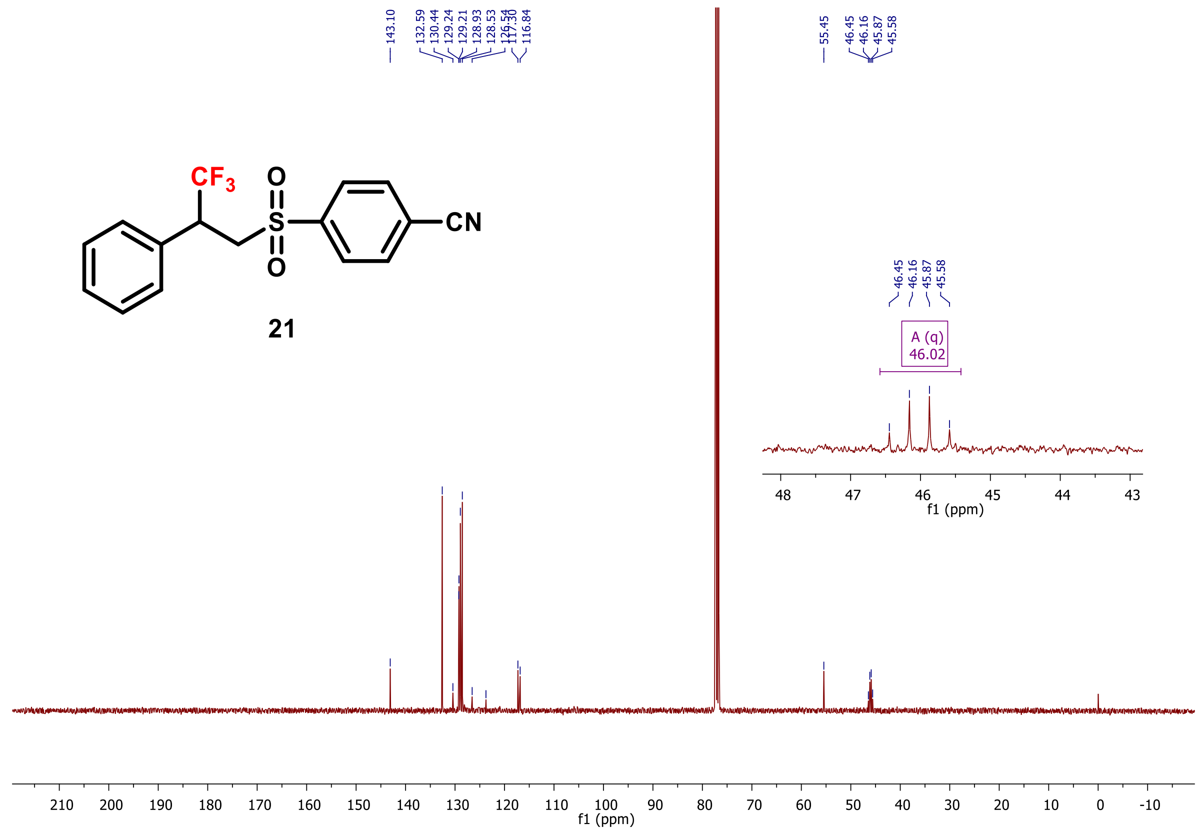

^{1}H NMR (400 MHz, CDCl$_3$) of **22**

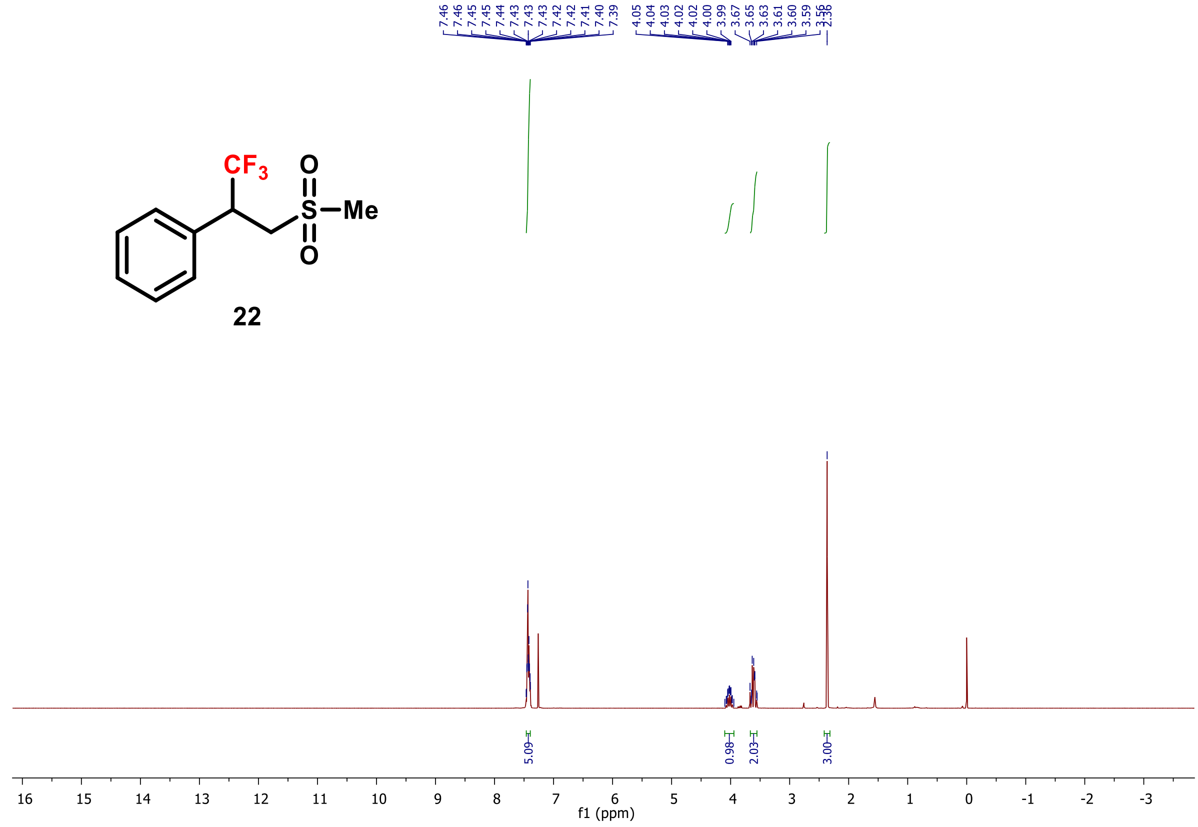

^{19}F NMR (376 MHz, CDCl$_3$) of **22**

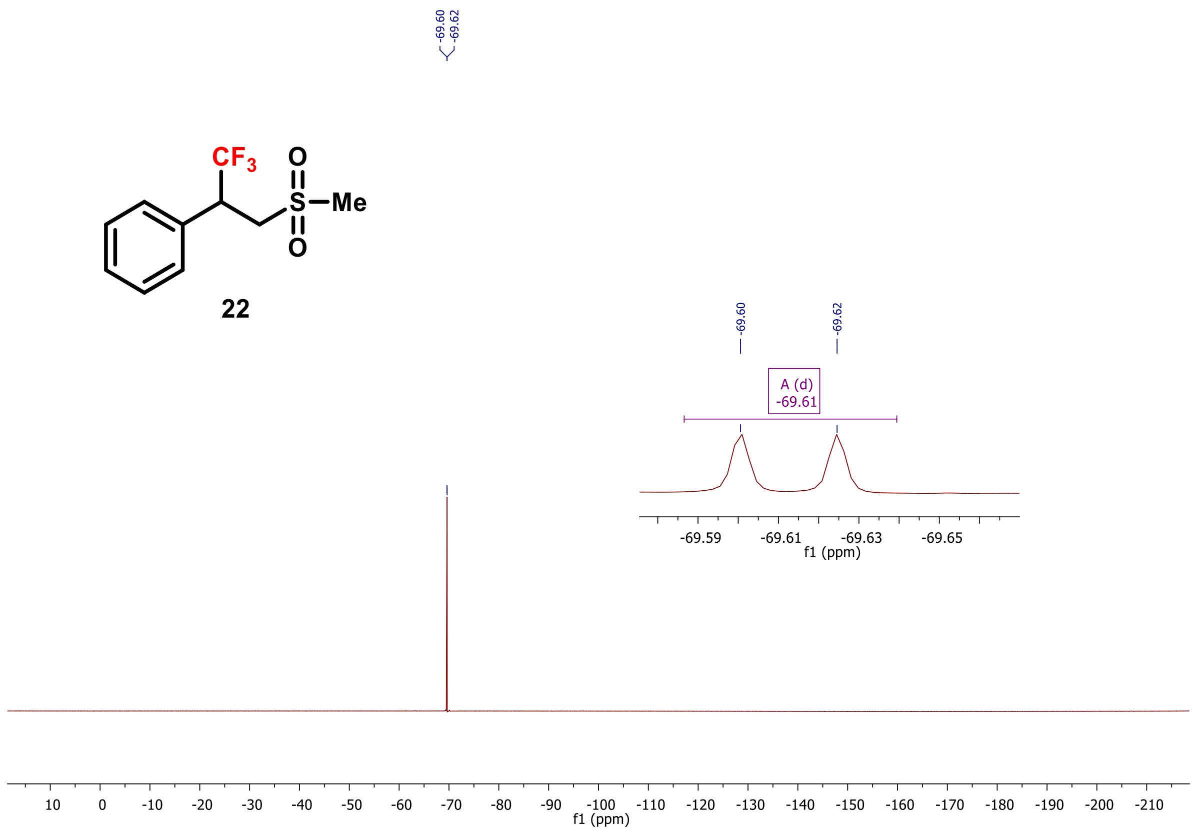

^{13}C NMR (101 MHz, $CDCl_3$) of **22**

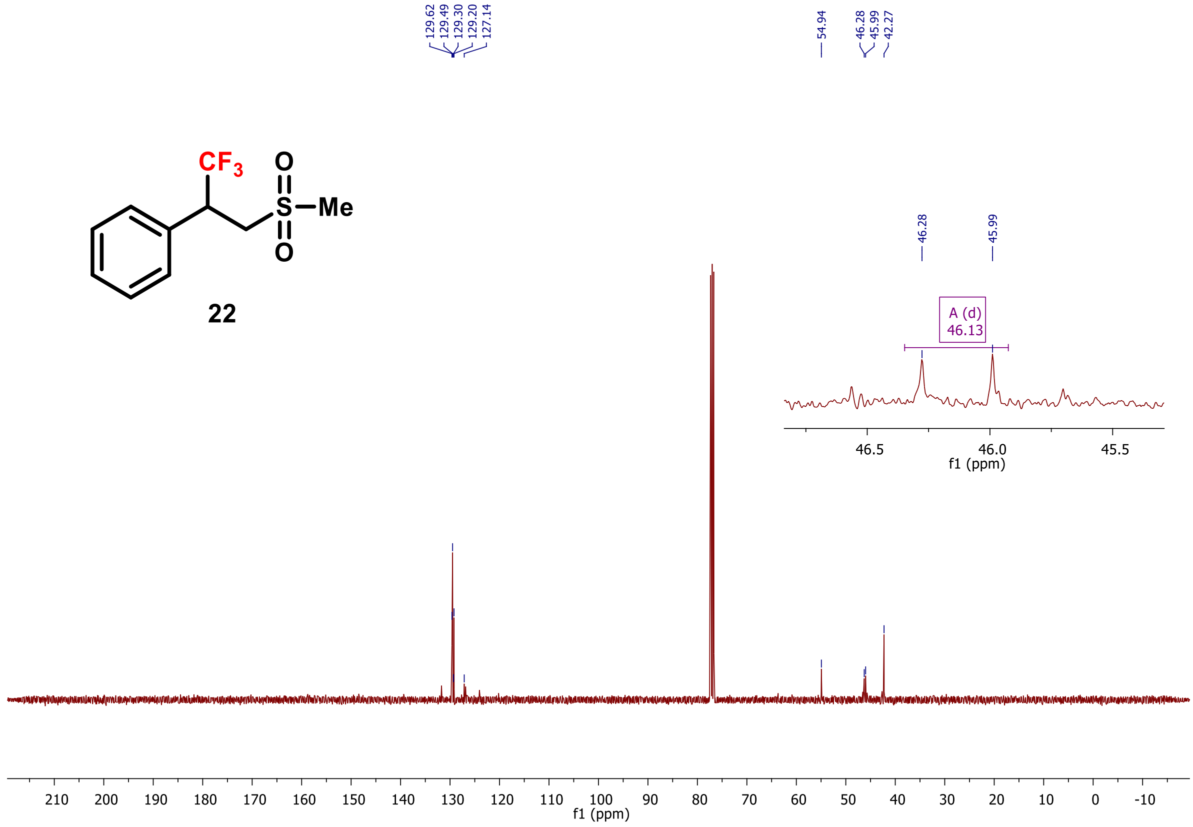

^{1}H NMR (400 MHz, $CDCl_3$) of **23**

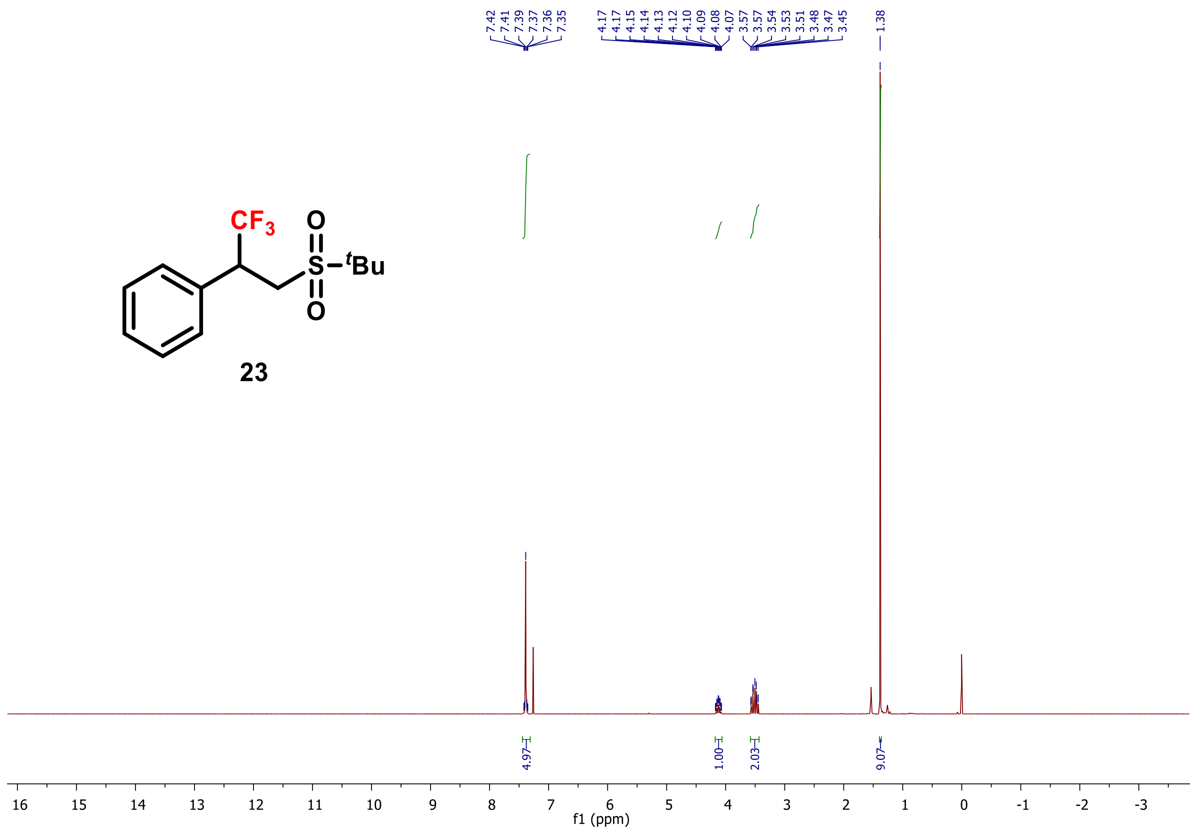

^{19}F NMR (376 MHz, $CDCl_3$) of **23**

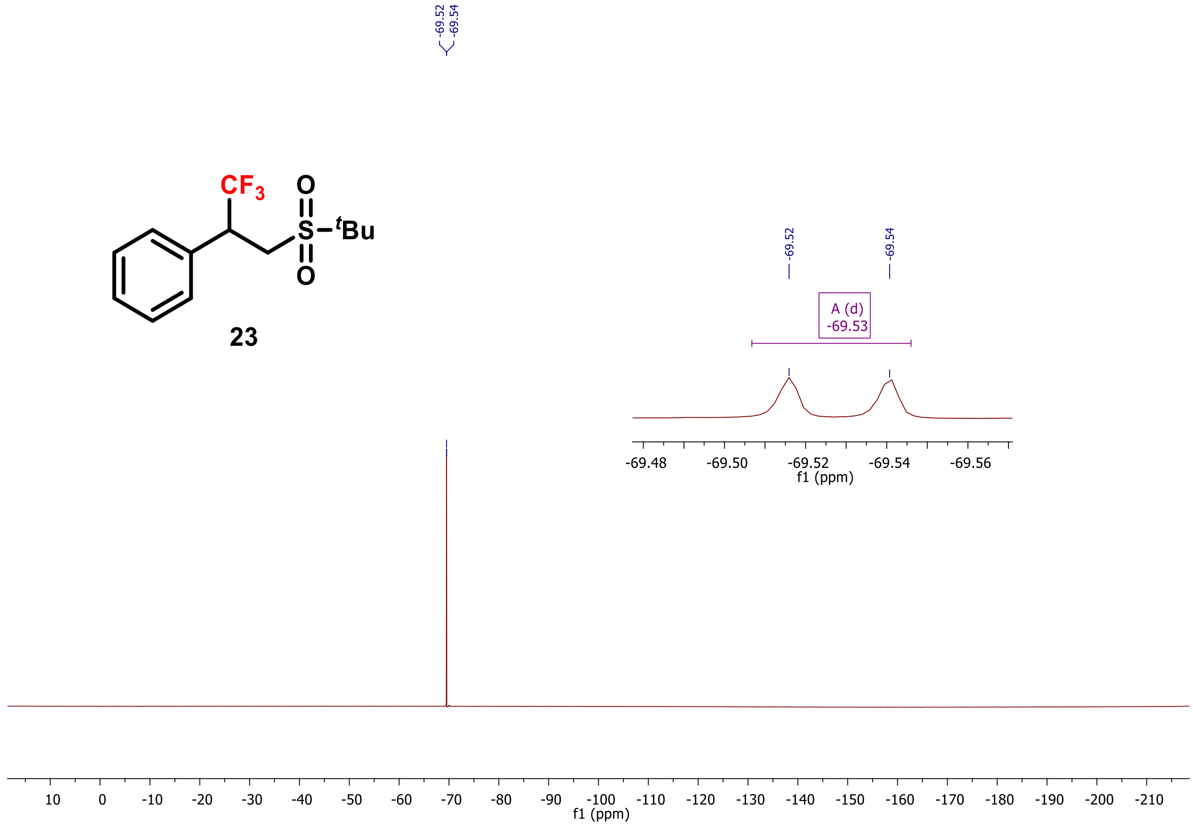

^{13}C NMR (101 MHz, $CDCl_3$) of **23**

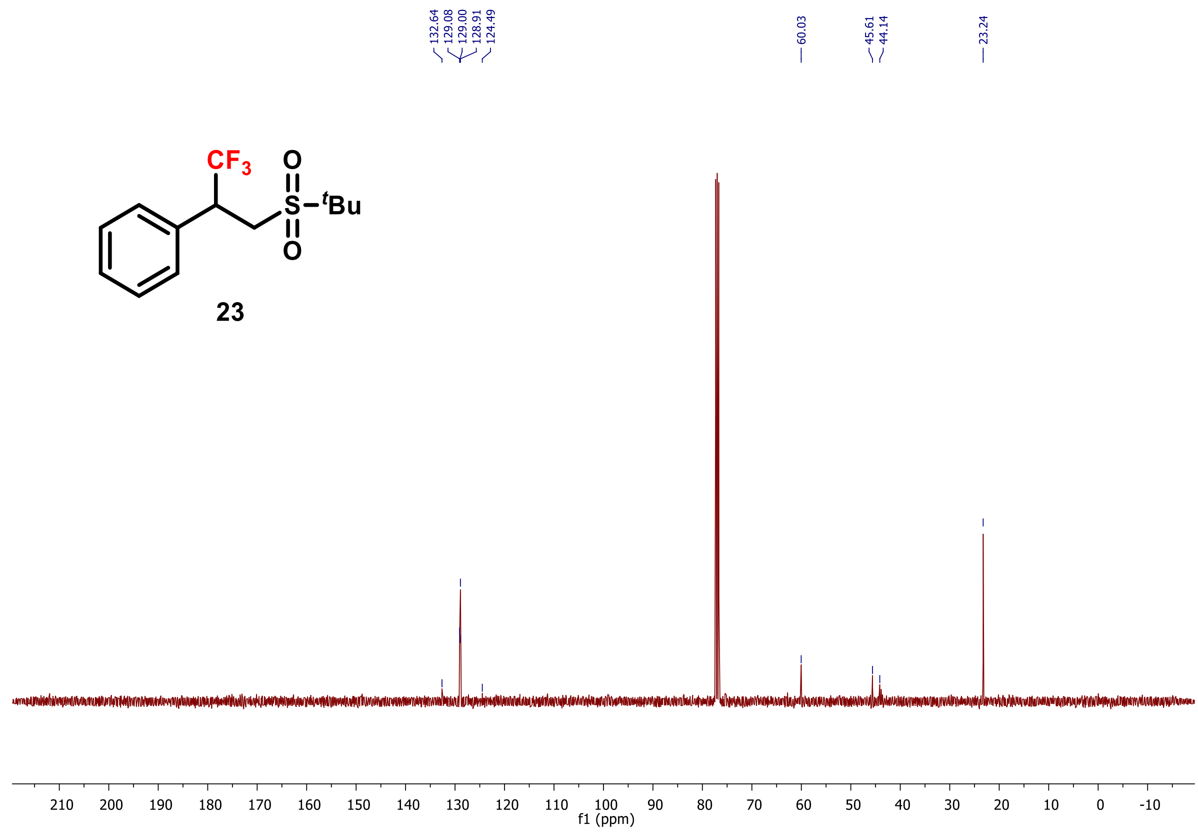

^{1}H NMR (400 MHz, $CDCl_3$) of **24**

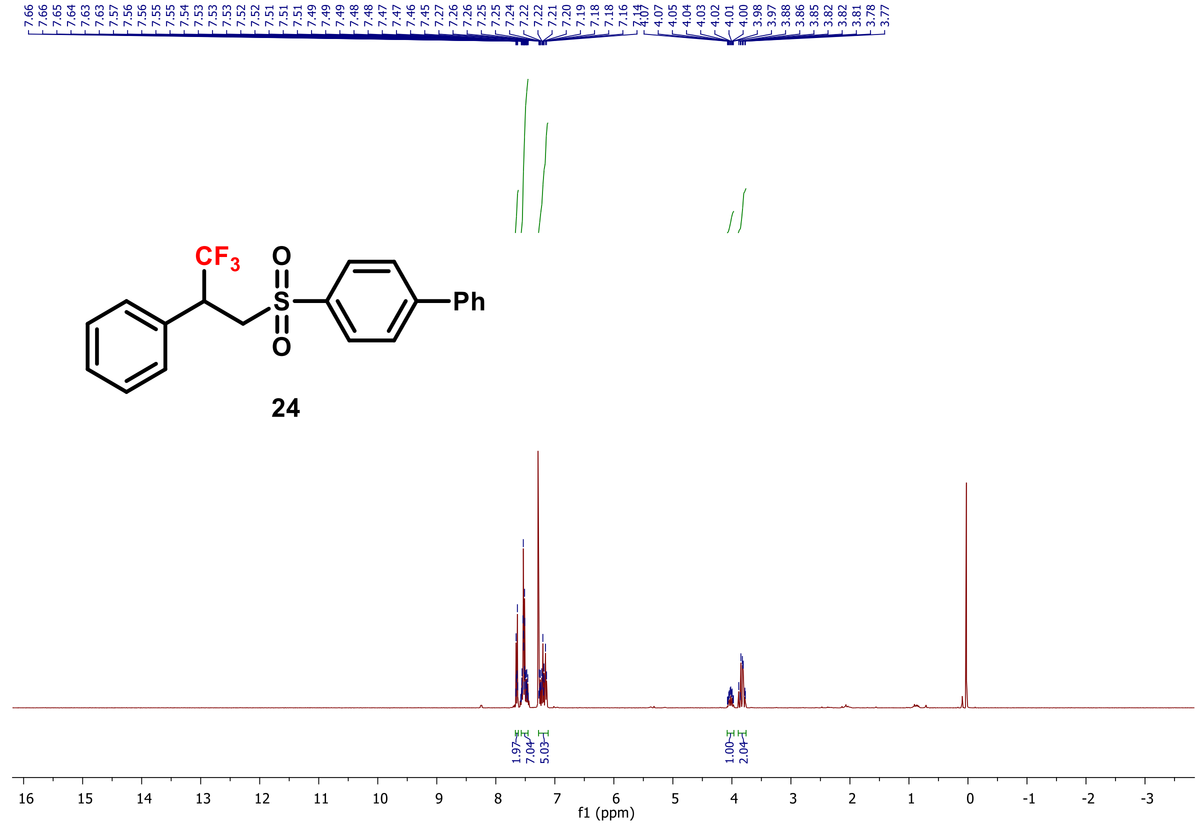

^{19}F NMR (376 MHz, $CDCl_3$) of **24**

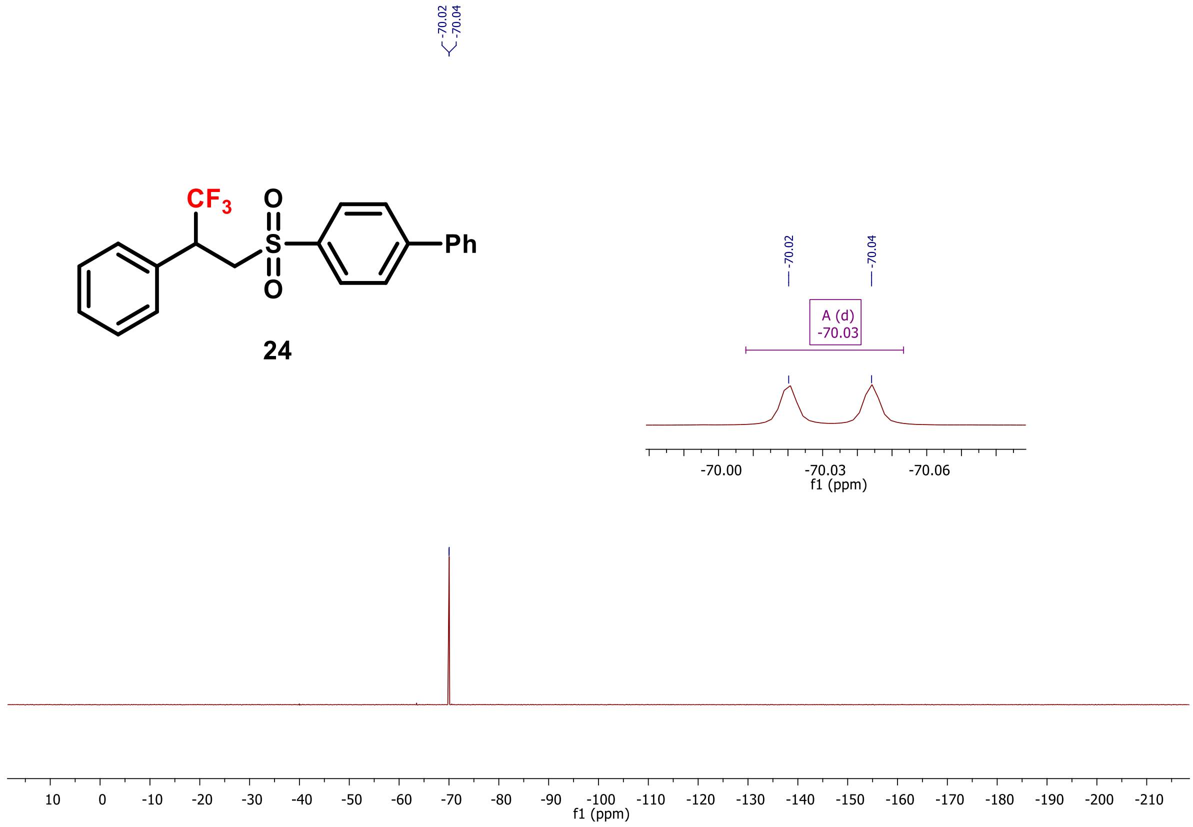

^{13}C NMR (101 MHz, $CDCl_3$) of **24**

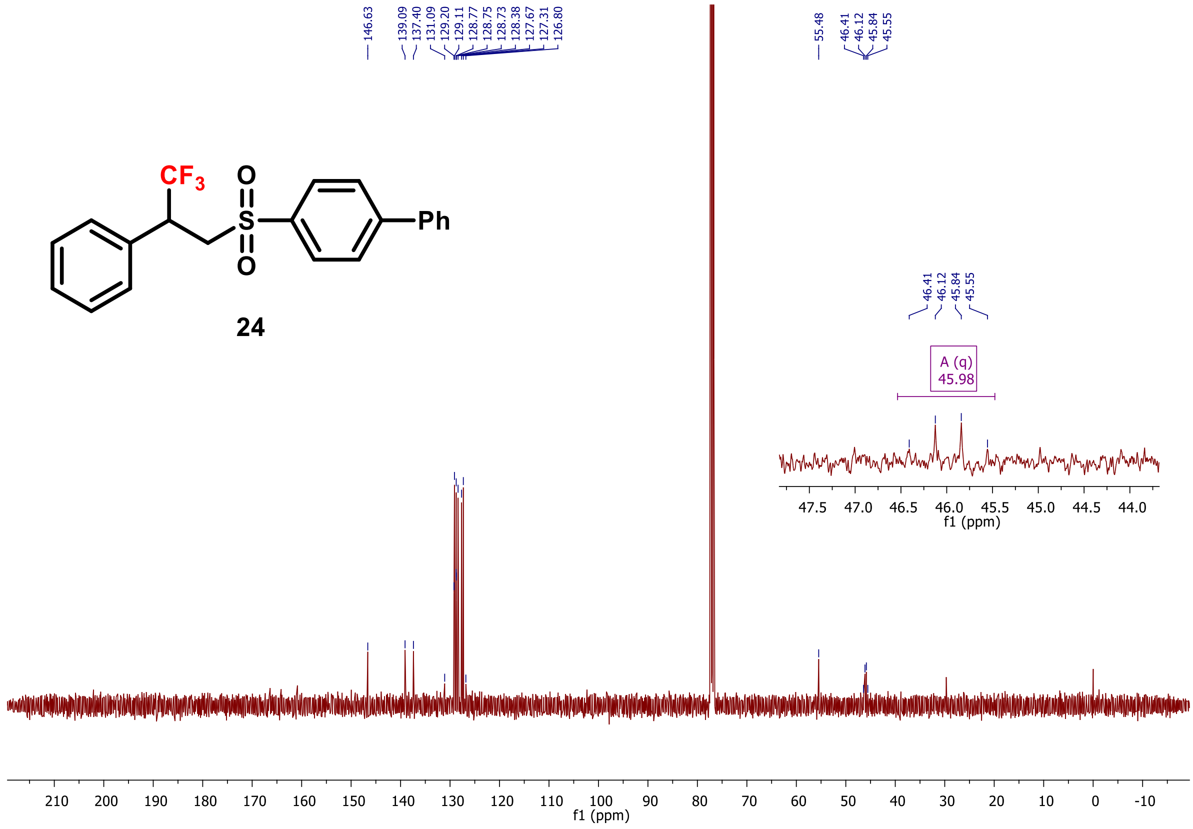

^{1}H NMR (400 MHz, $CDCl_3$) of **25**

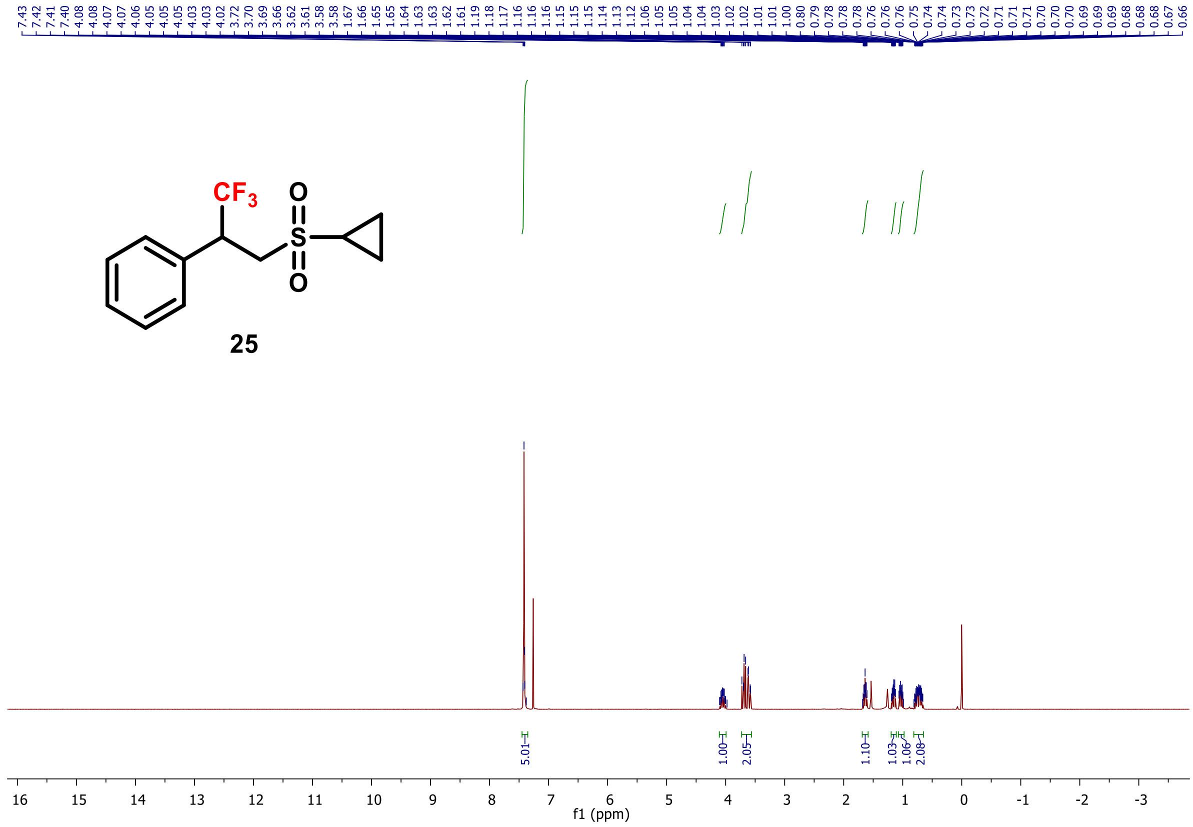

^{19}F NMR (376 MHz, $CDCl_3$) of **25**

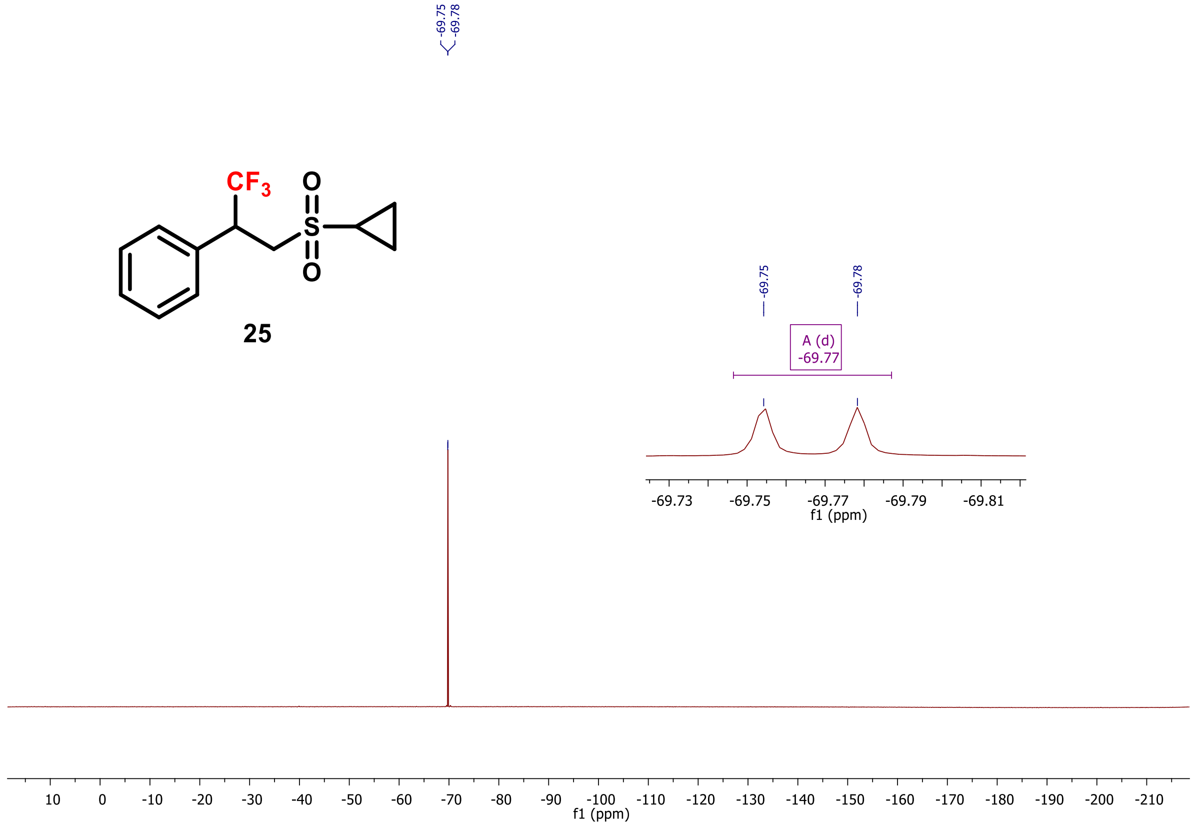

^{13}C NMR (101 MHz, $CDCl_3$) of **25**

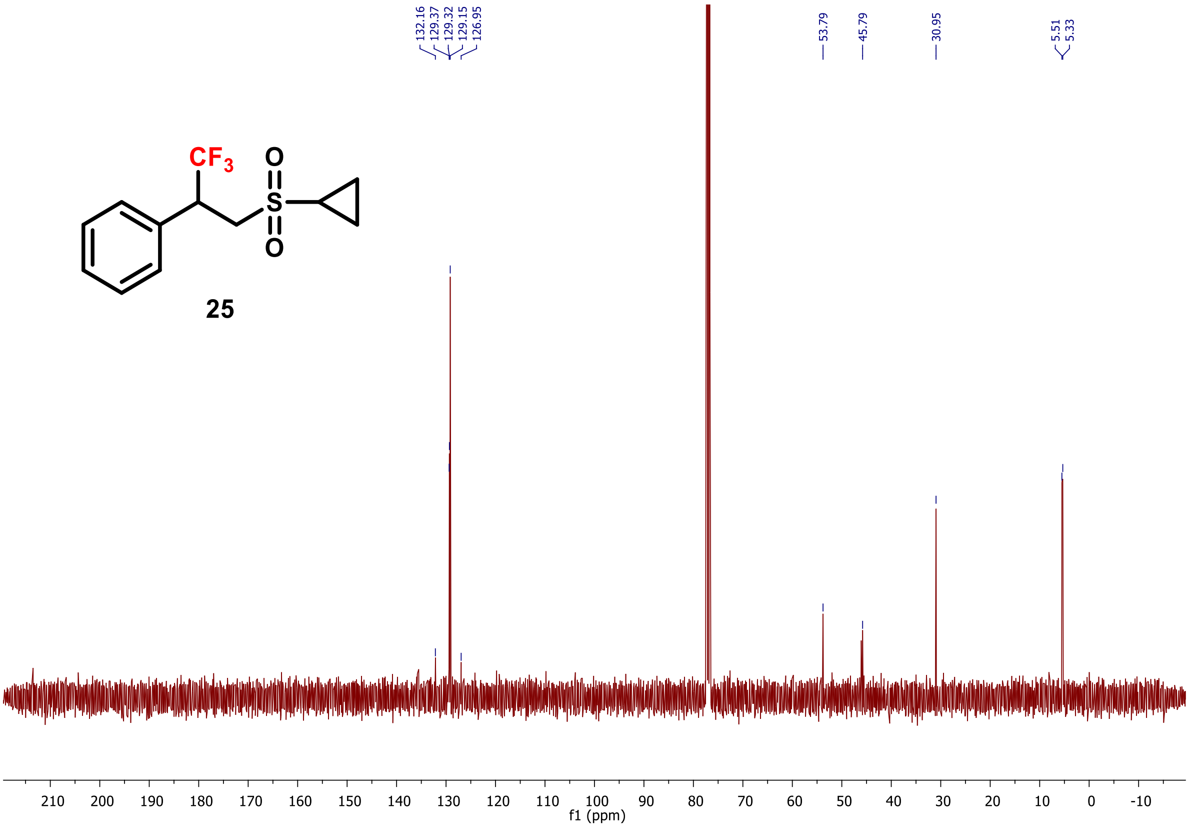

^{1}H NMR (400 MHz, CDCl$_3$) of **26**

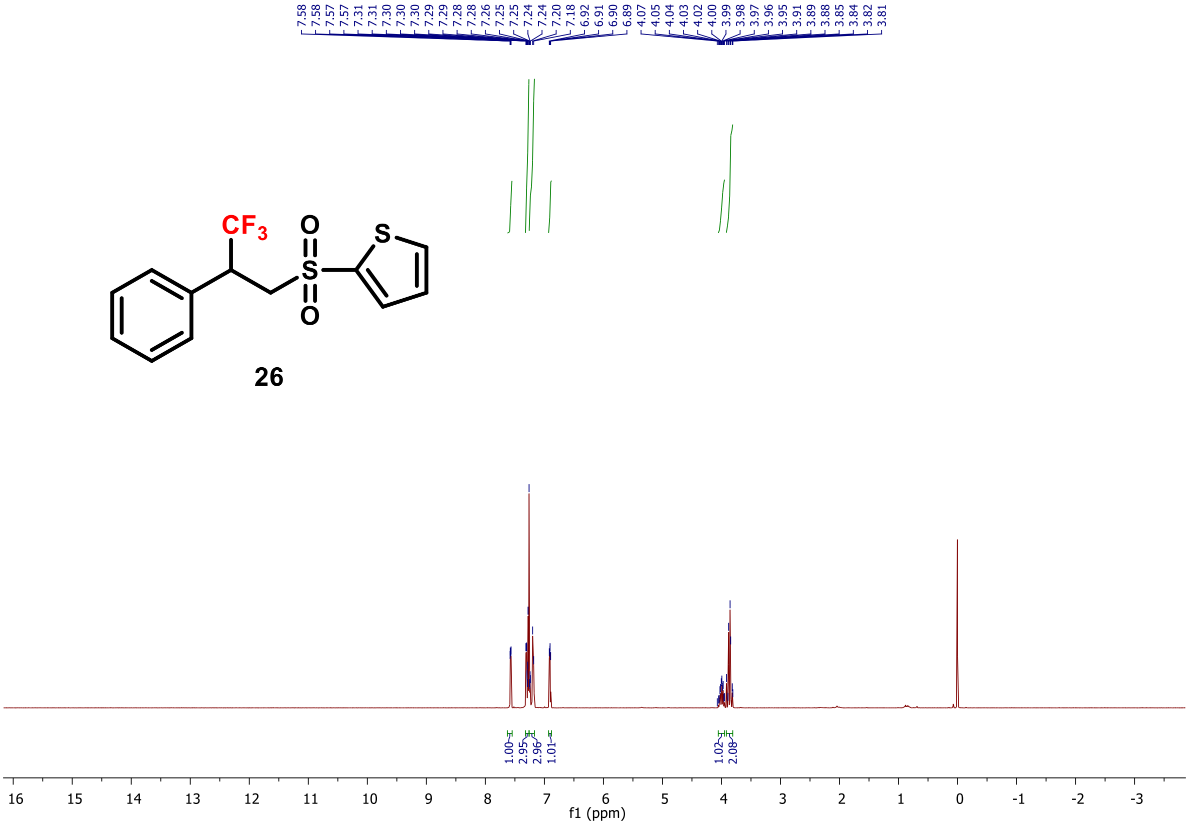

^{19}F NMR (376 MHz, CDCl$_3$) of **26**

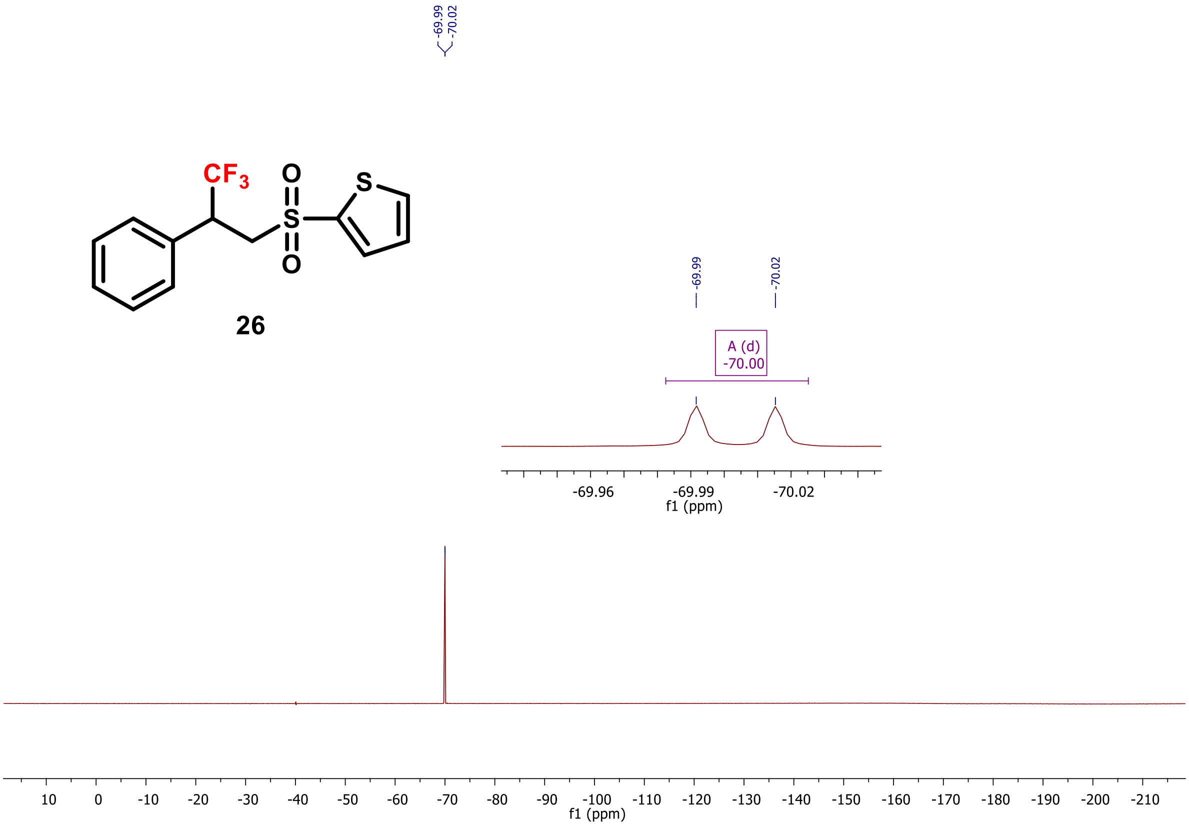

^{13}C NMR (101 MHz, $CDCl_3$) of **26**

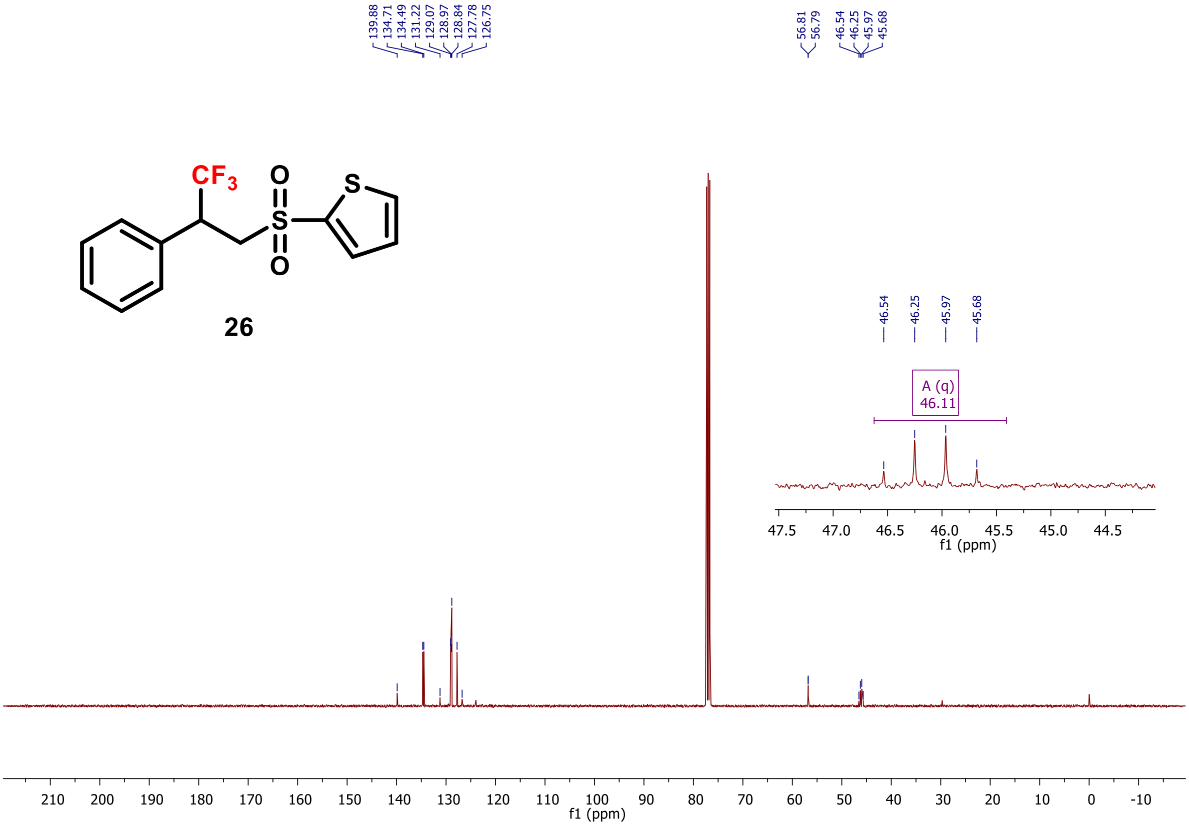

^{1}H NMR (400 MHz, $CDCl_3$) of **27**

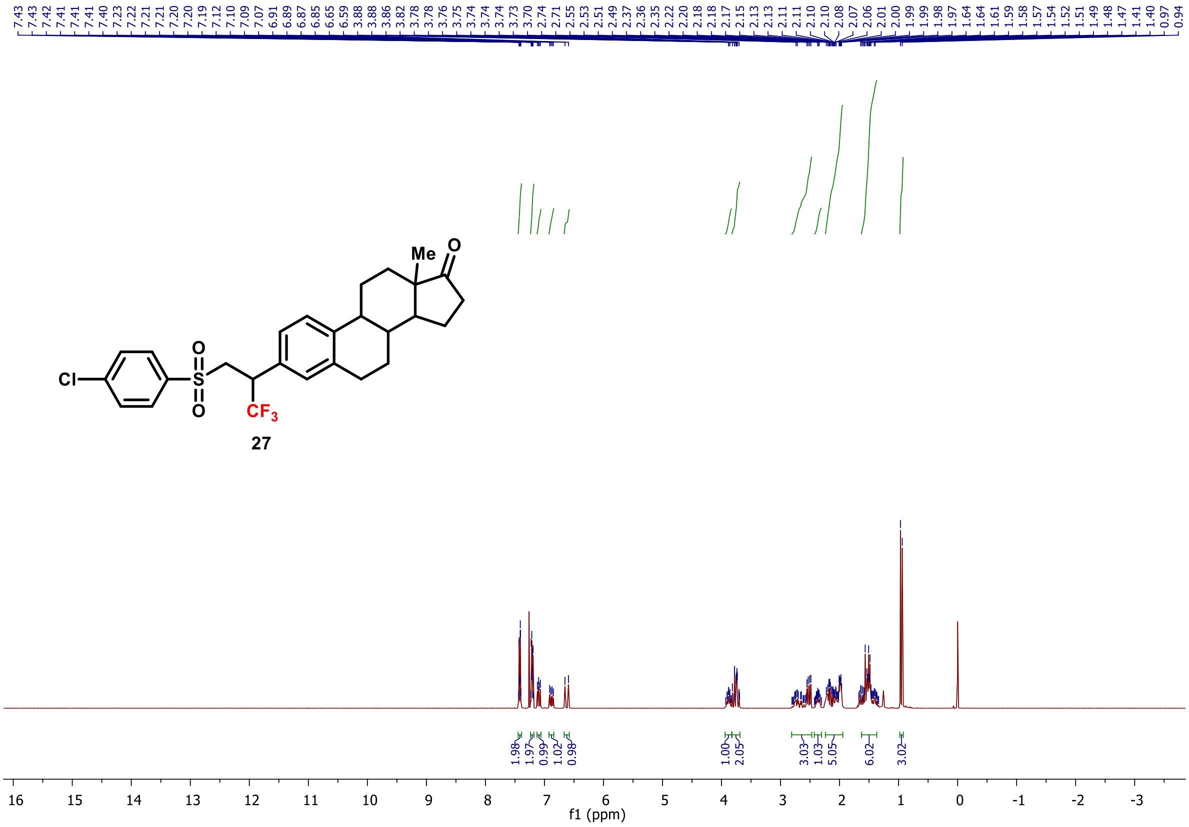

^{19}F NMR (376 MHz, $CDCl_3$) of **27**

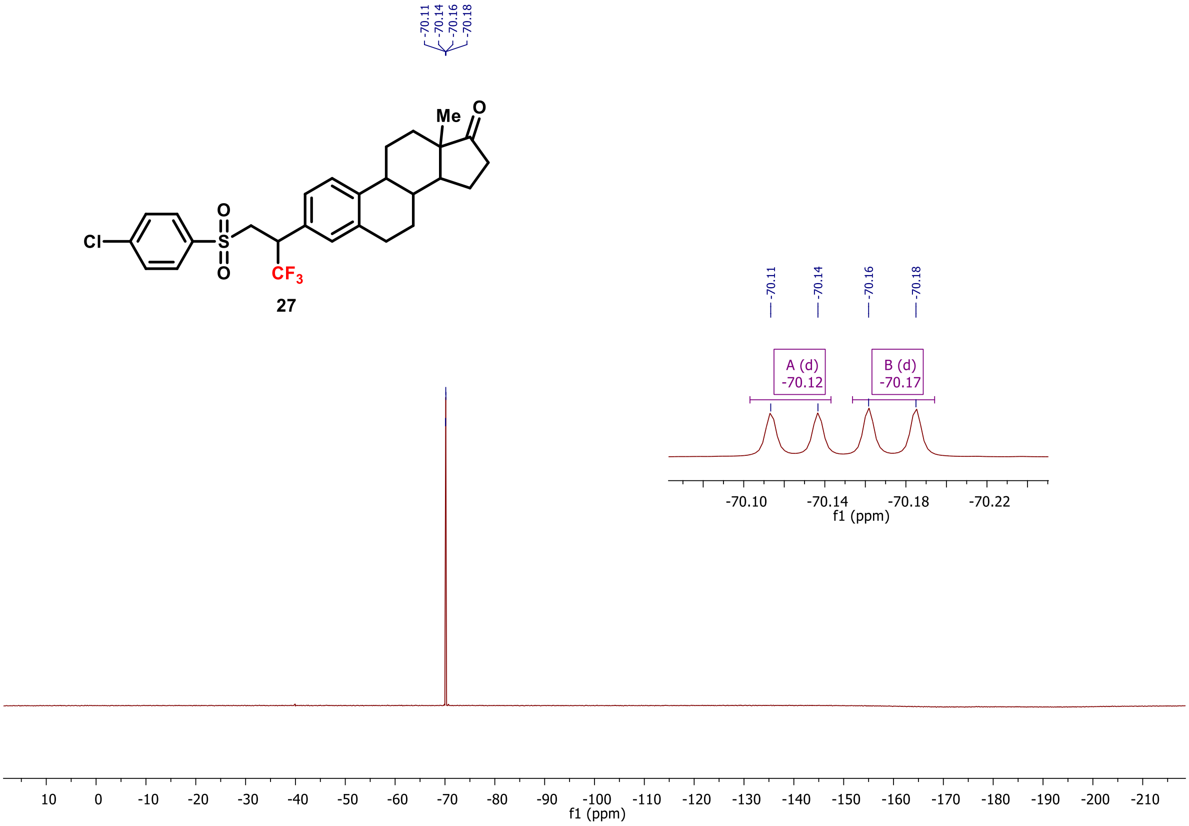

^{13}C NMR (101 MHz, $CDCl_3$) of **27**

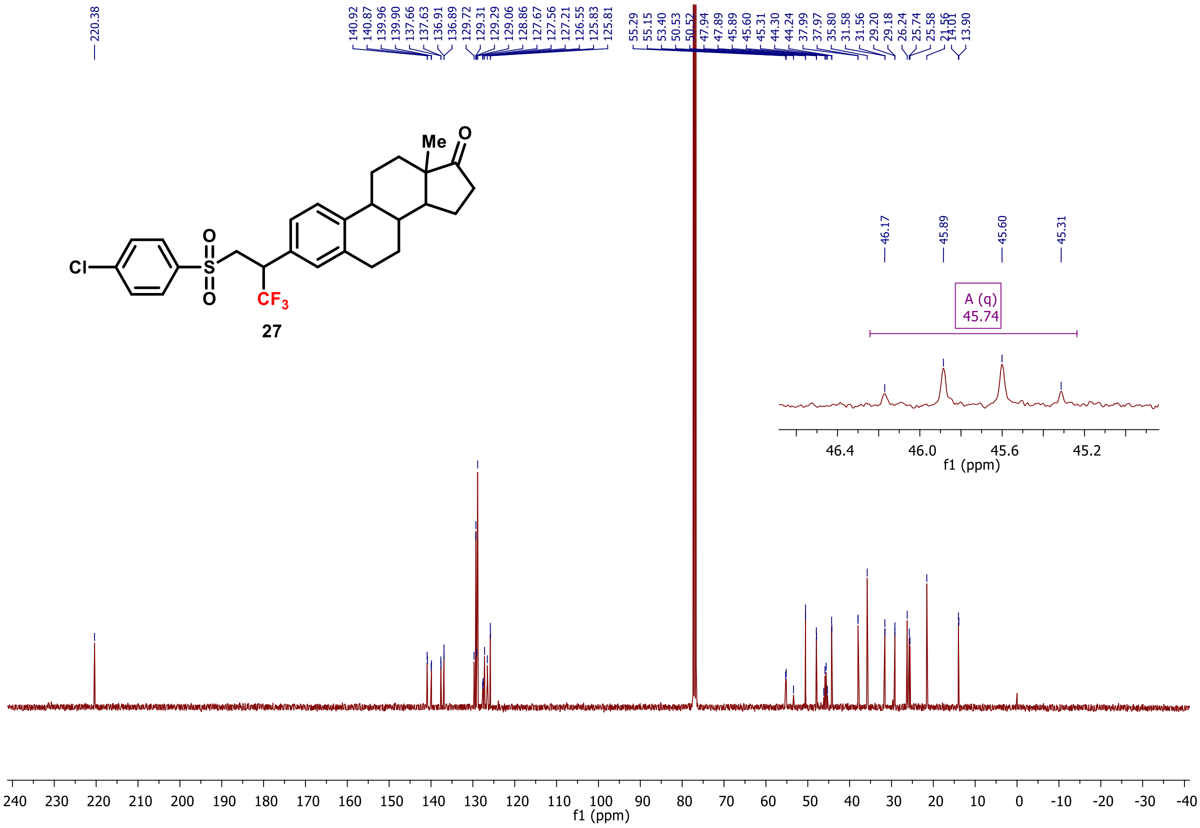

^{1}H NMR (400 MHz, $CDCl_3$) of **28**

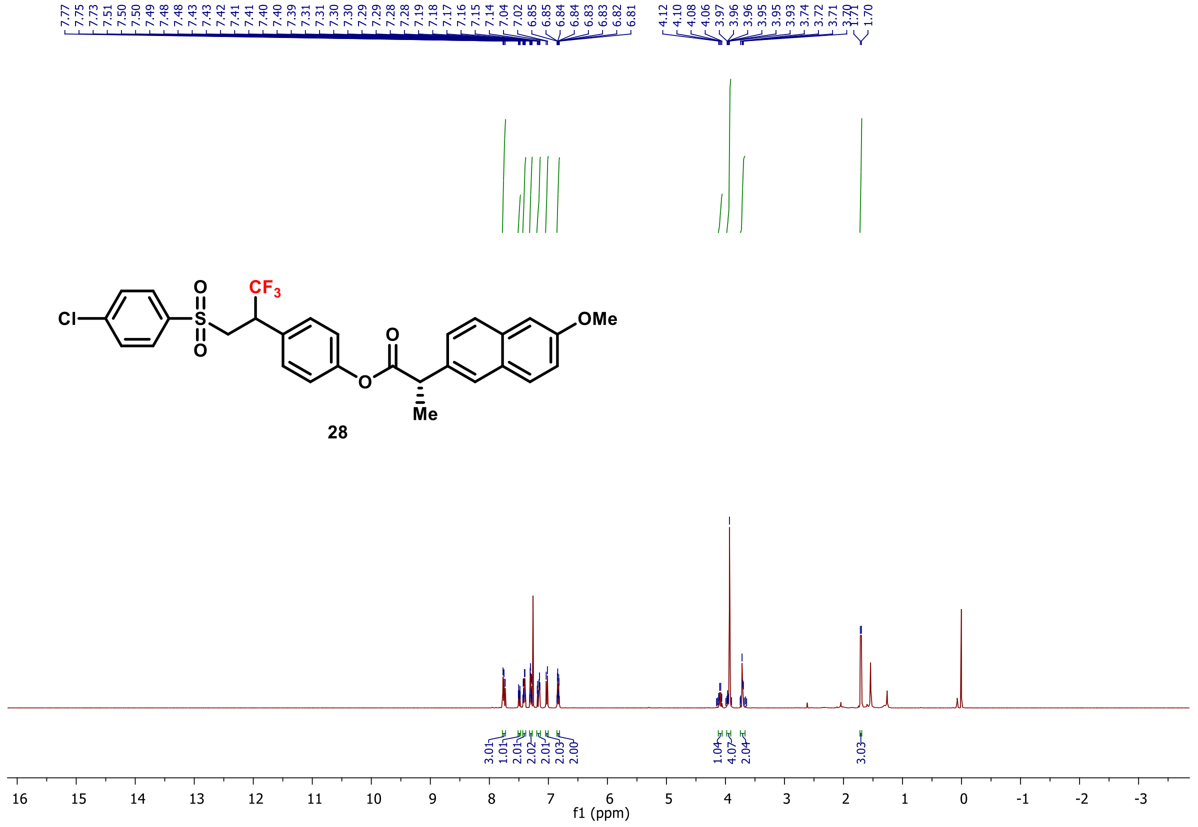

^{19}F NMR (376 MHz, $CDCl_3$) of **28**

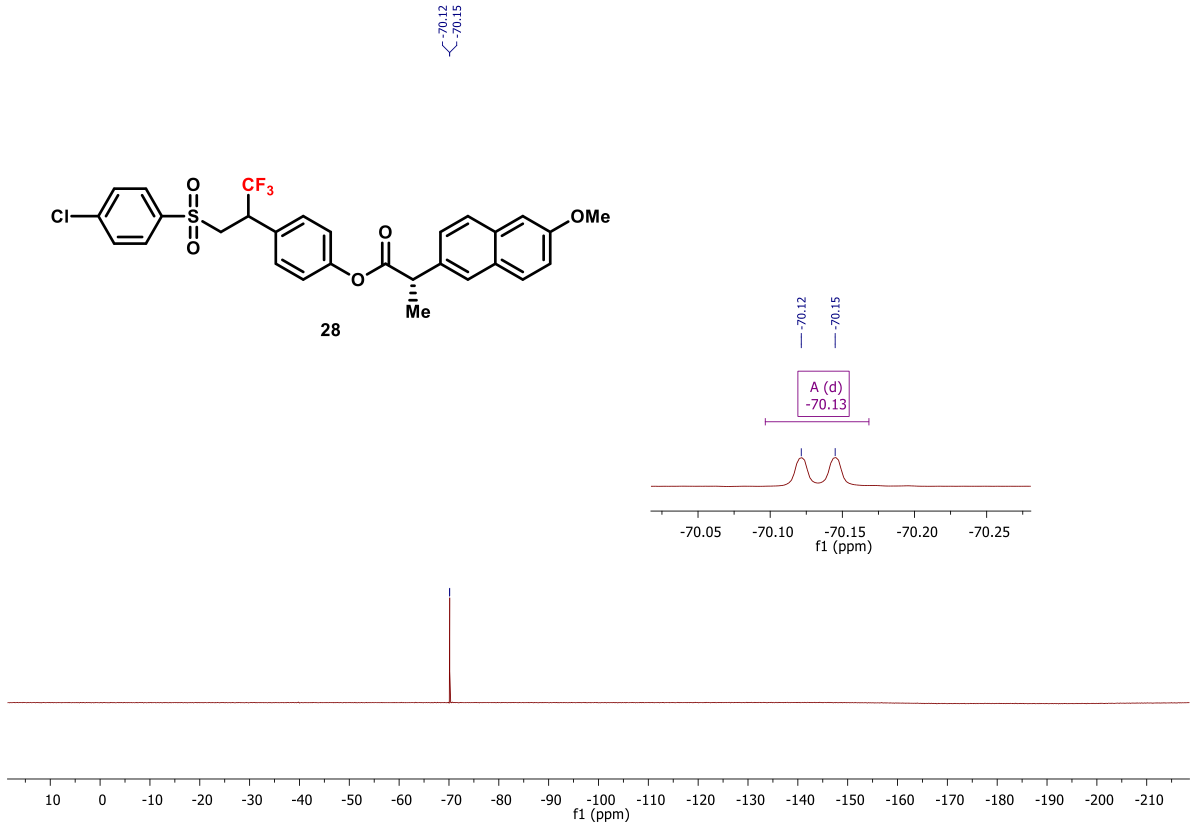

^{13}C NMR (101 MHz, $CDCl_3$) of **28**

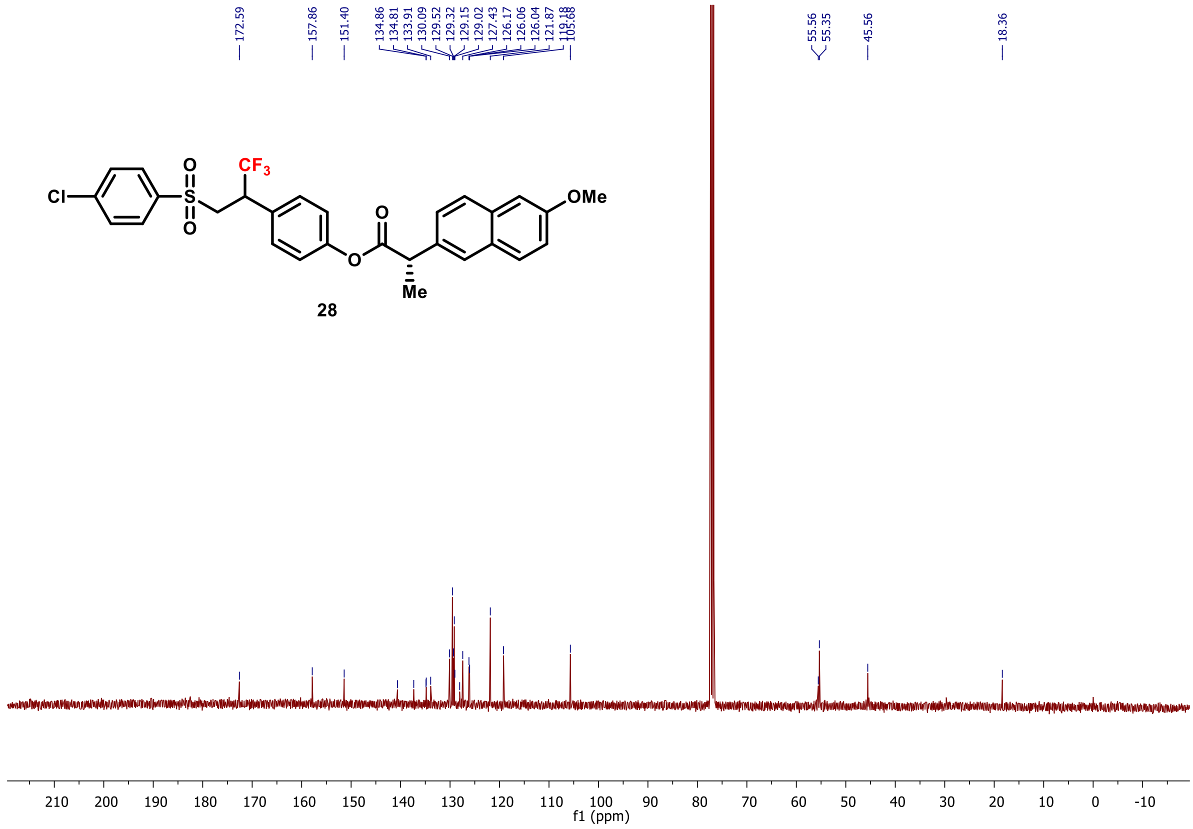

^{1}H NMR (400 MHz, $CDCl_3$) of **29**

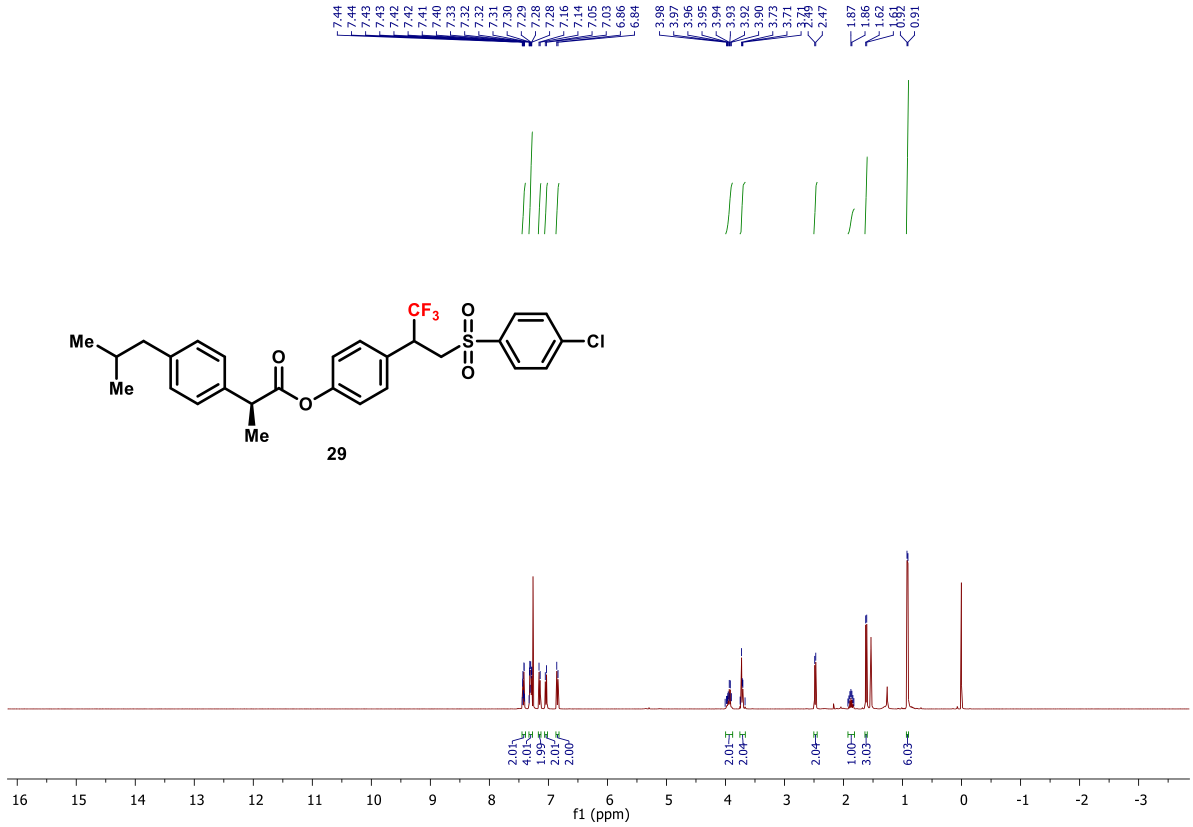

^{19}F NMR (376 MHz, $CDCl_3$) of **29**

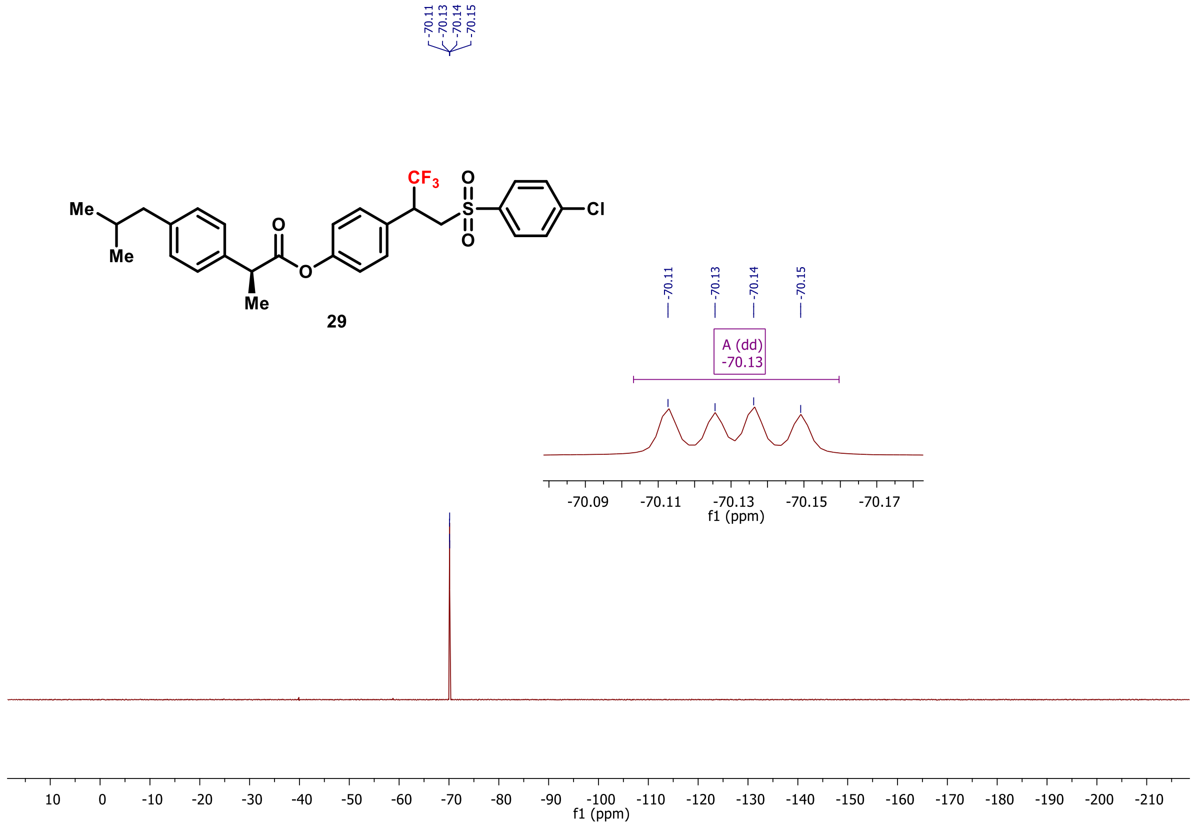

^{13}C NMR (101 MHz, $CDCl_3$) of **29**

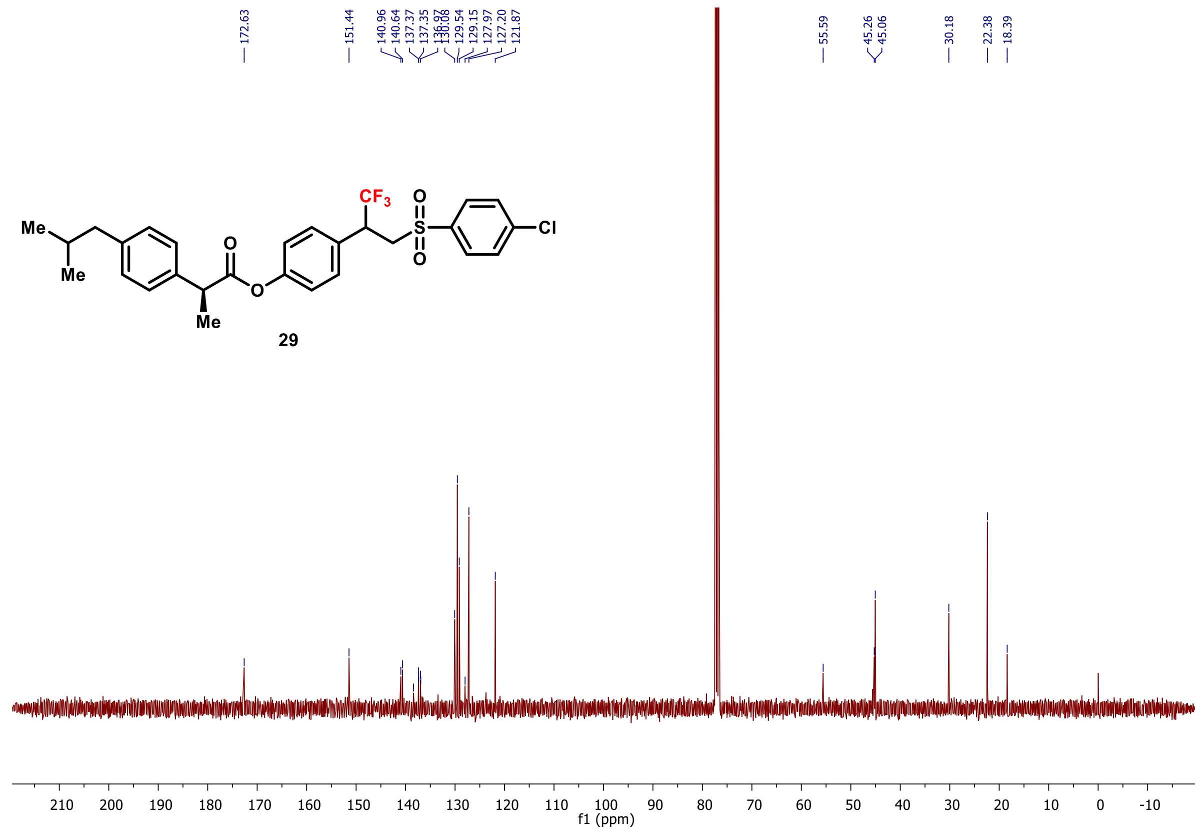

^{1}H NMR (400 MHz, $CDCl_3$) of **30**

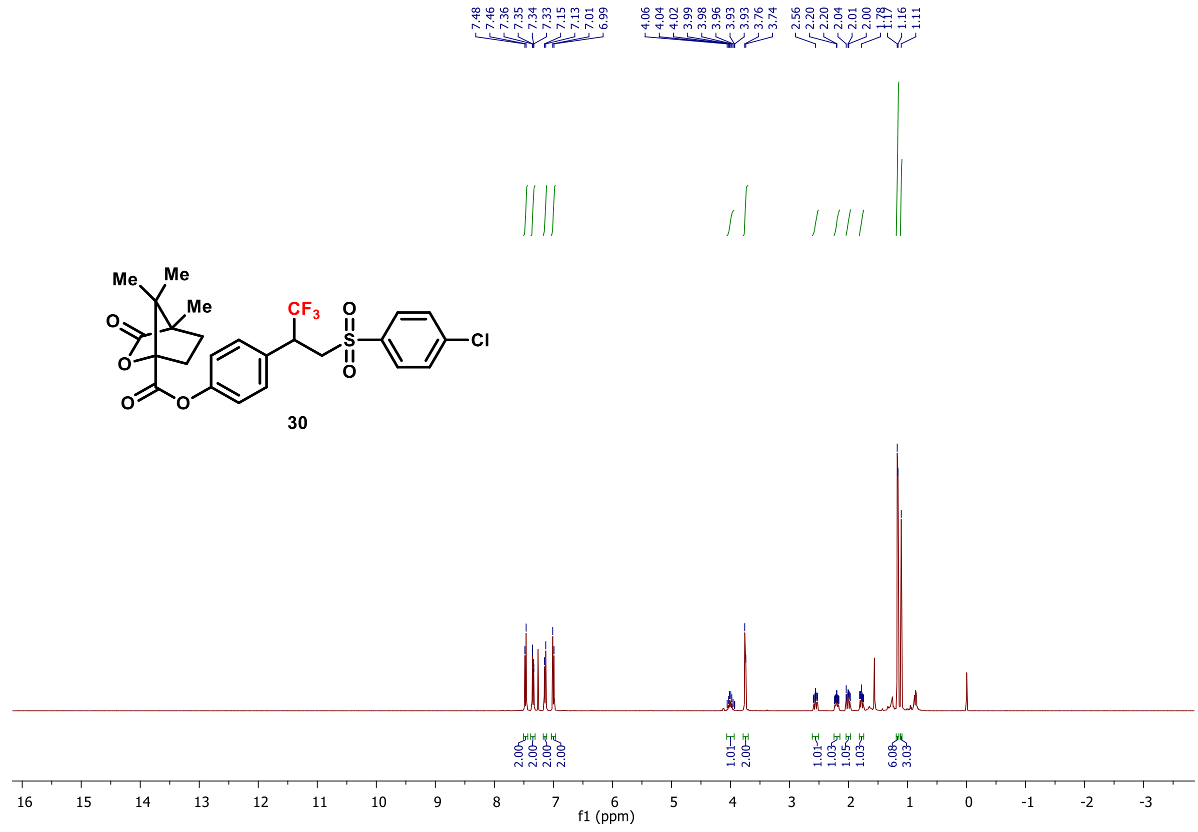

^{19}F NMR (376 MHz, $CDCl_3$) of **30**

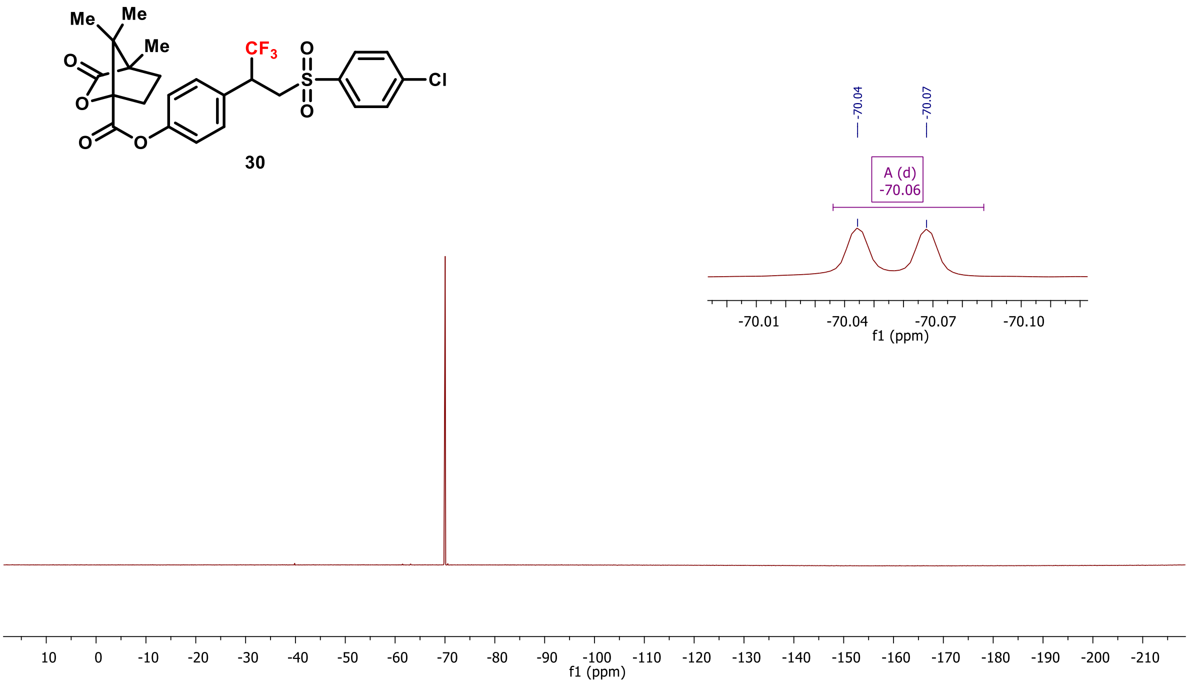

^{13}C NMR (101 MHz, $CDCl_3$) of **30**

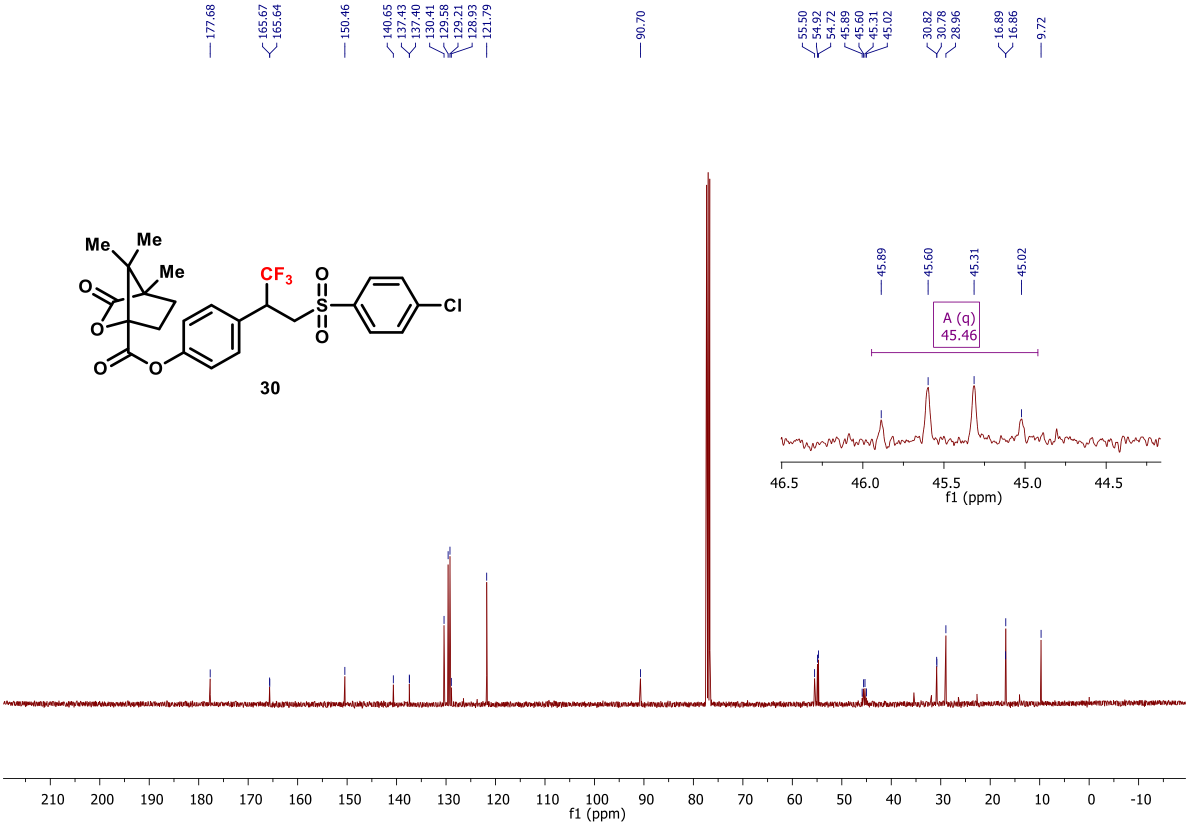

^{1}H NMR (400 MHz, $CDCl_3$) of **31**

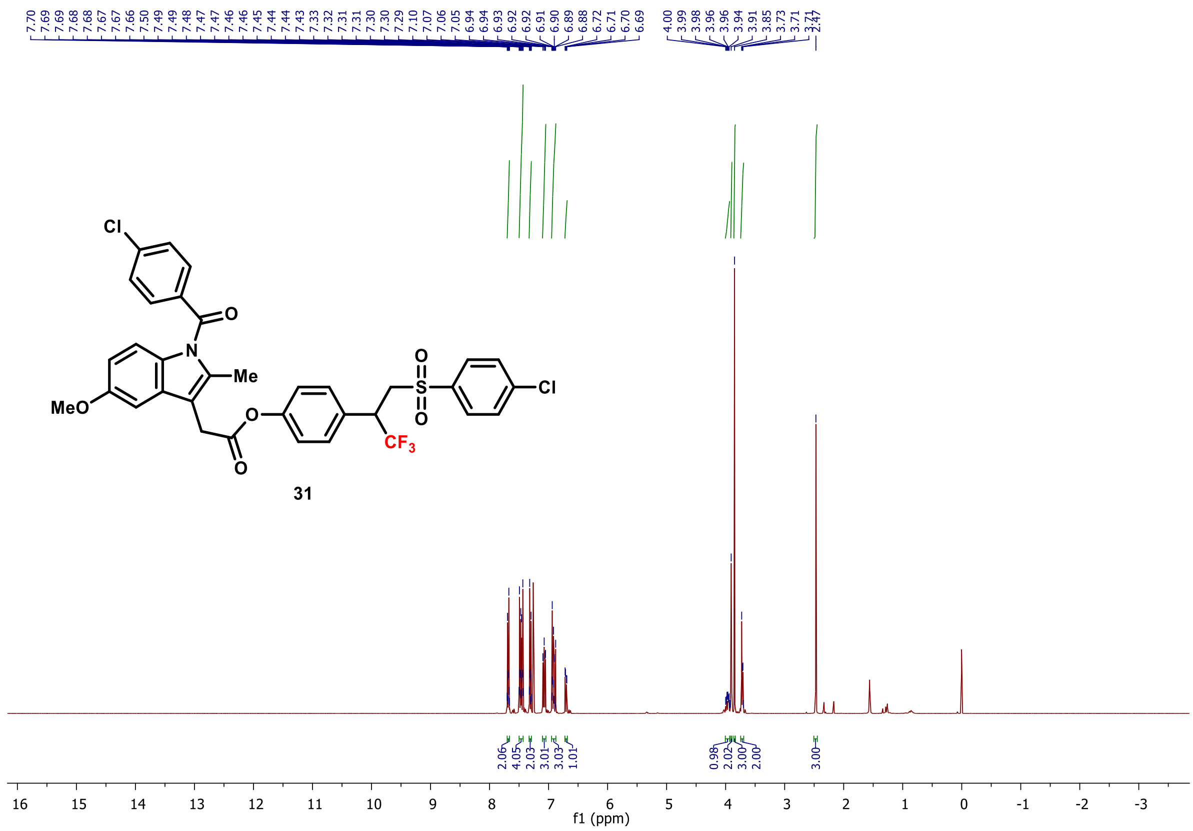

^{19}**F NMR** (376 MHz, $CDCl_3$) of **31**

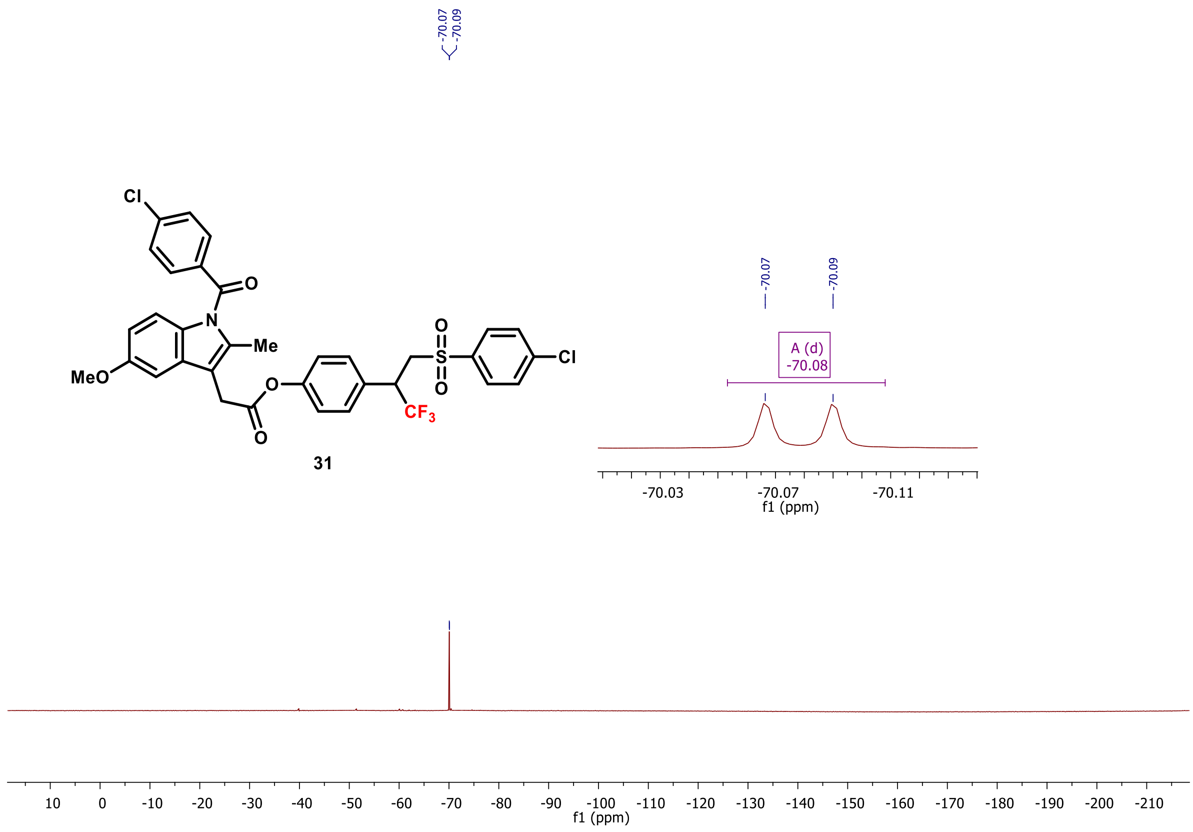

^{13}**C NMR** (101 MHz, $CDCl_3$) of **31**

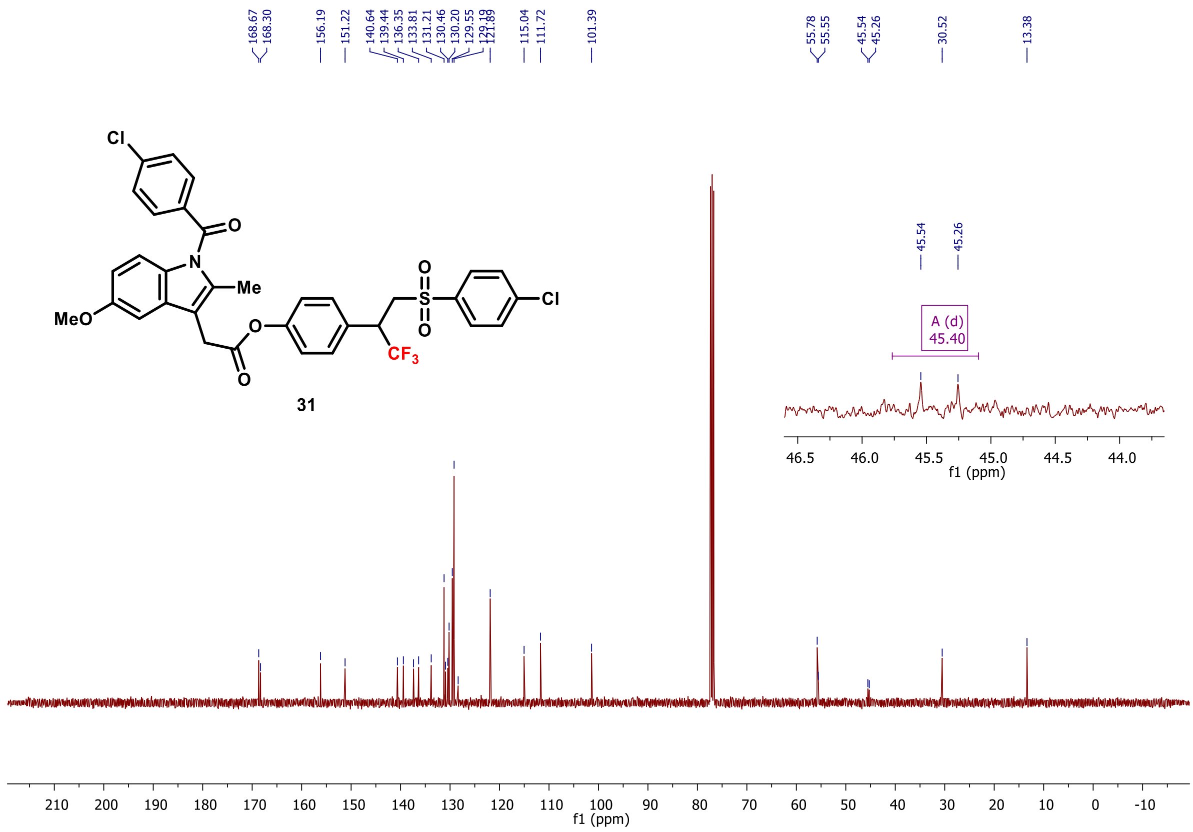

^{1}H NMR (400 MHz, $CDCl_3$) of **32**

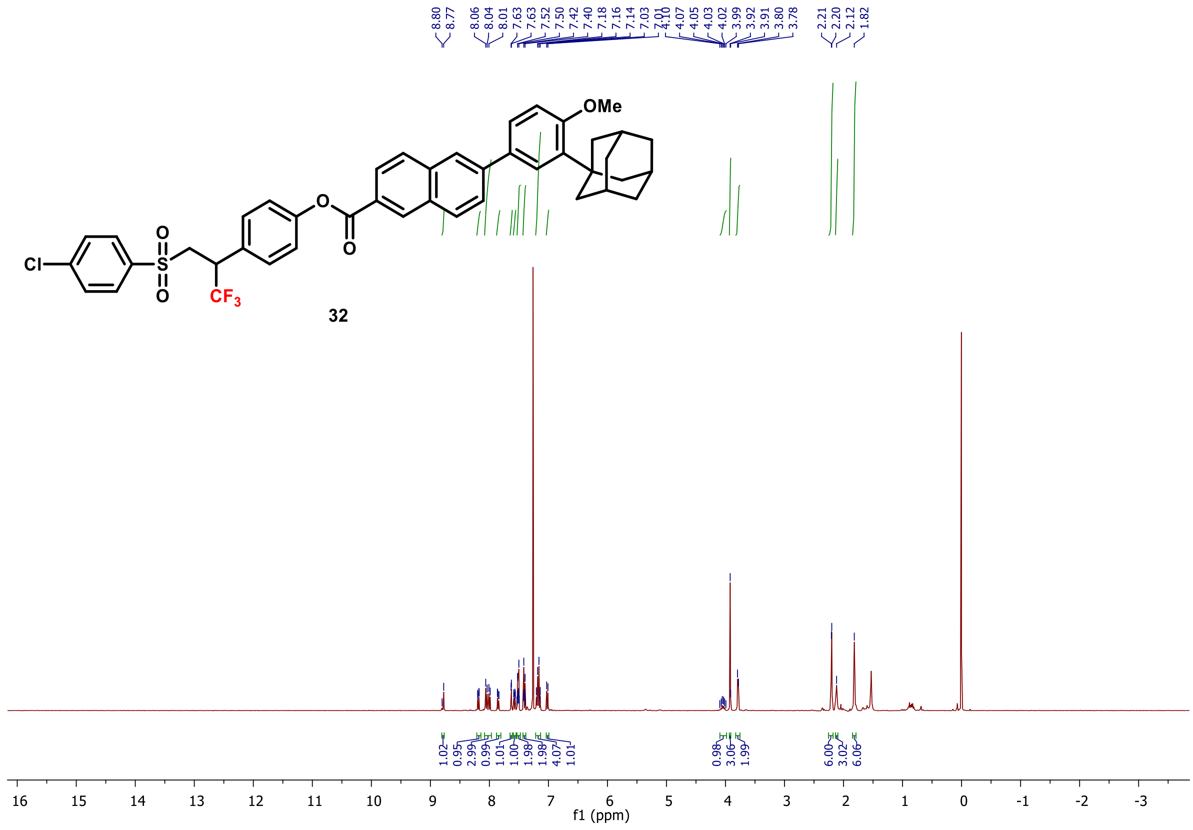

^{19}F NMR (376 MHz, $CDCl_3$) of **32**

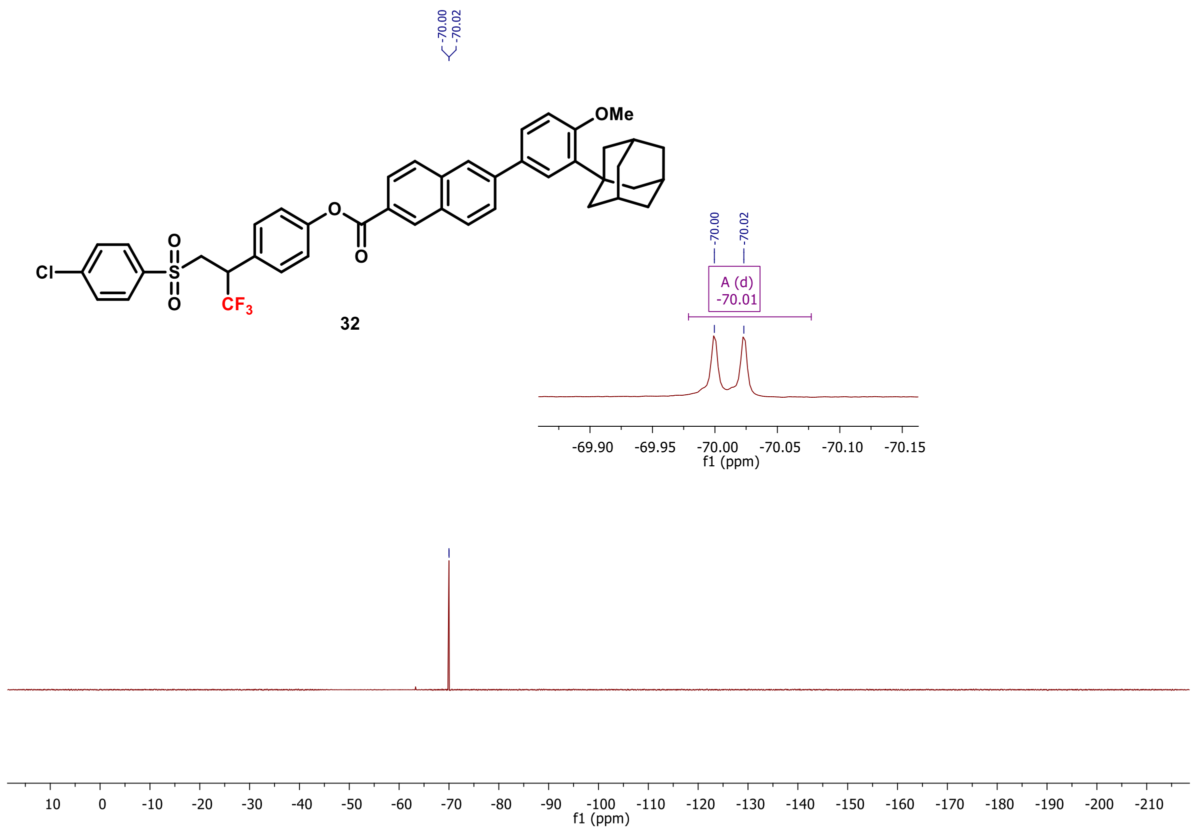

^{13}C NMR (101 MHz, CDCl3) of **32**

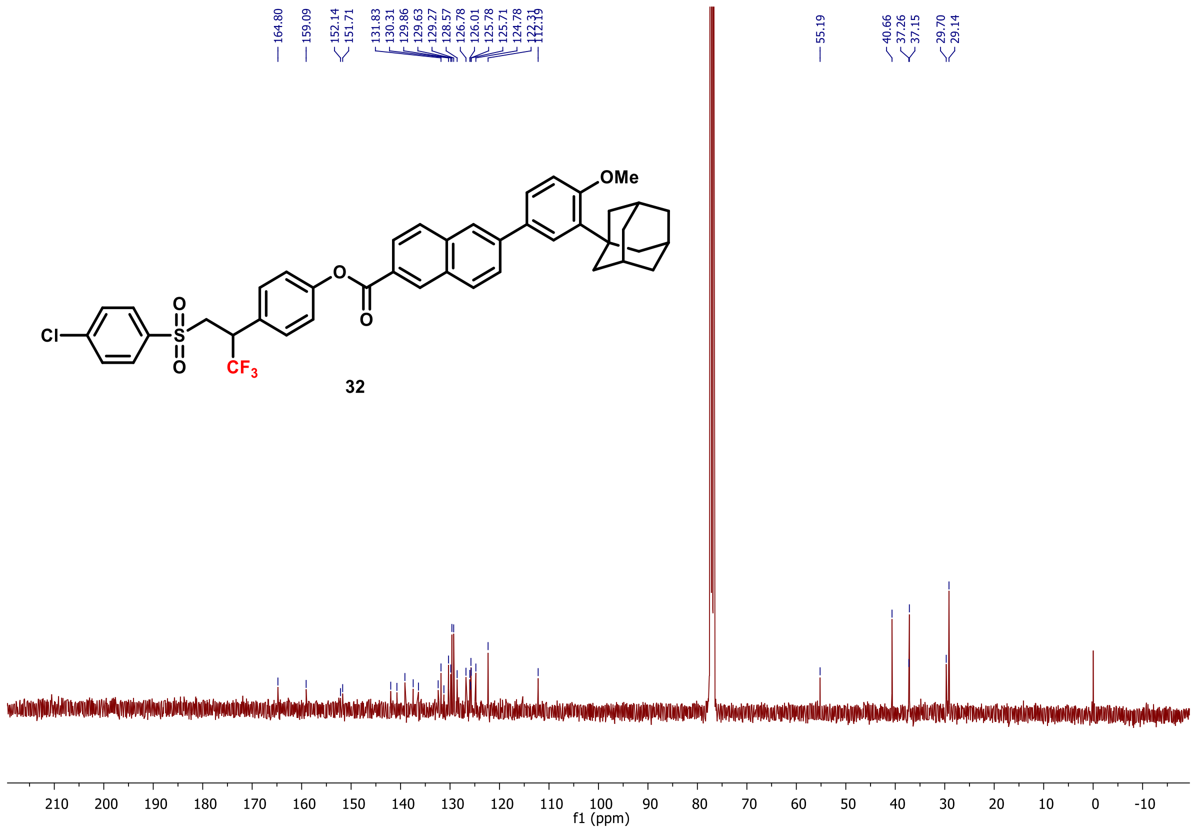

^{1}H NMR (400 MHz, CDCl3) of **33**

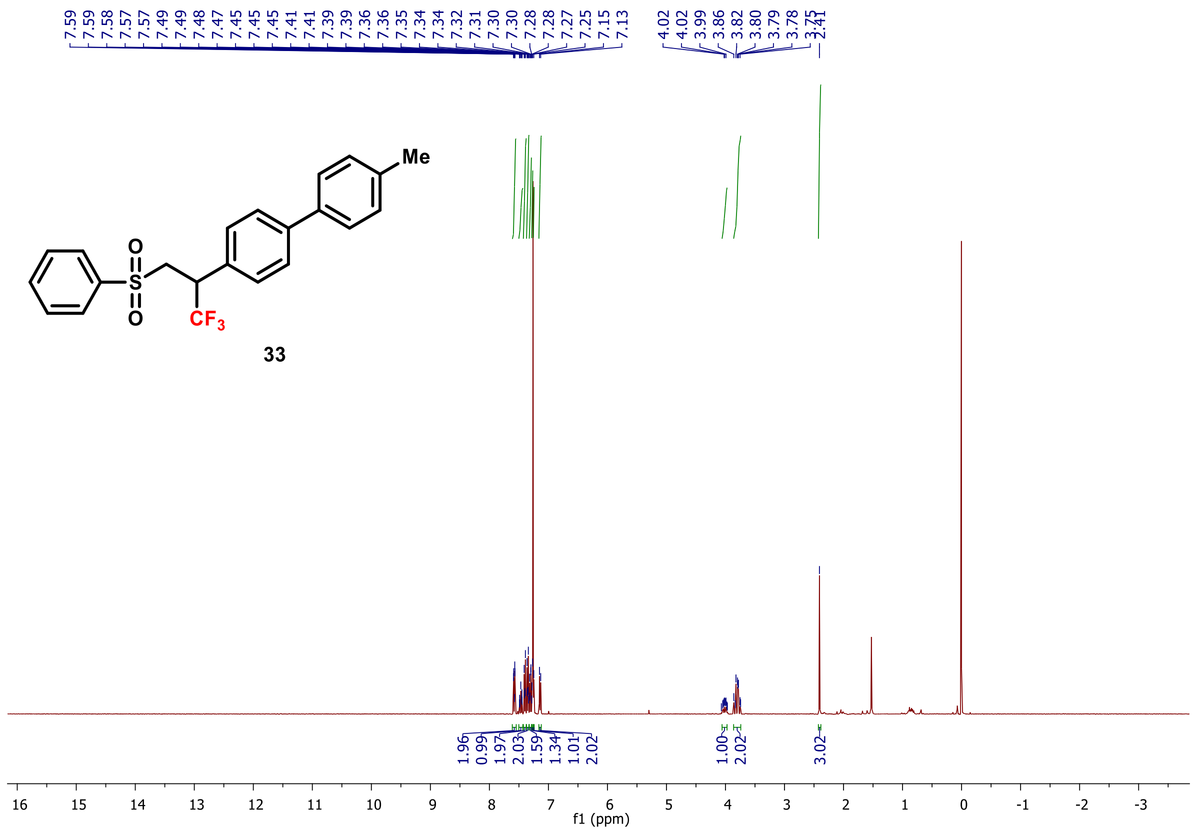

^{19}F NMR (376 MHz, $CDCl_3$) of **33**

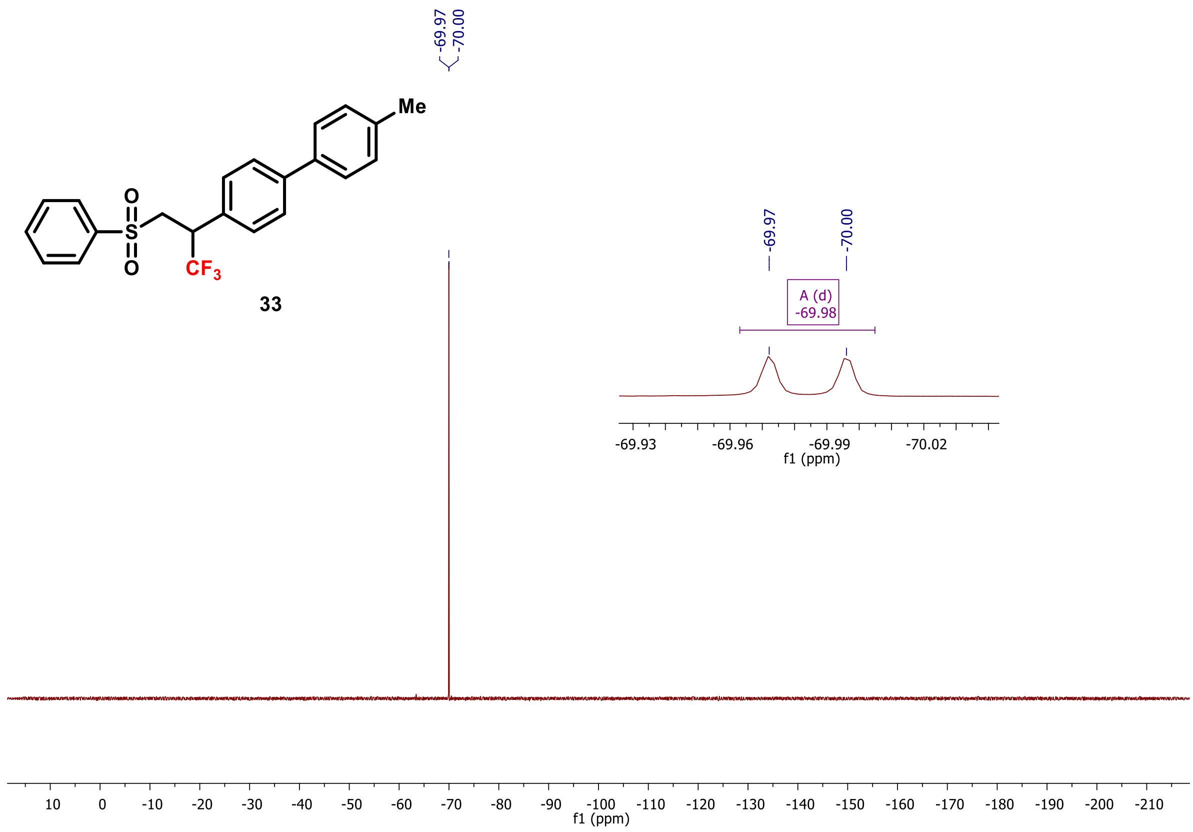

^{13}C NMR (101 MHz, $CDCl_3$) of **33**

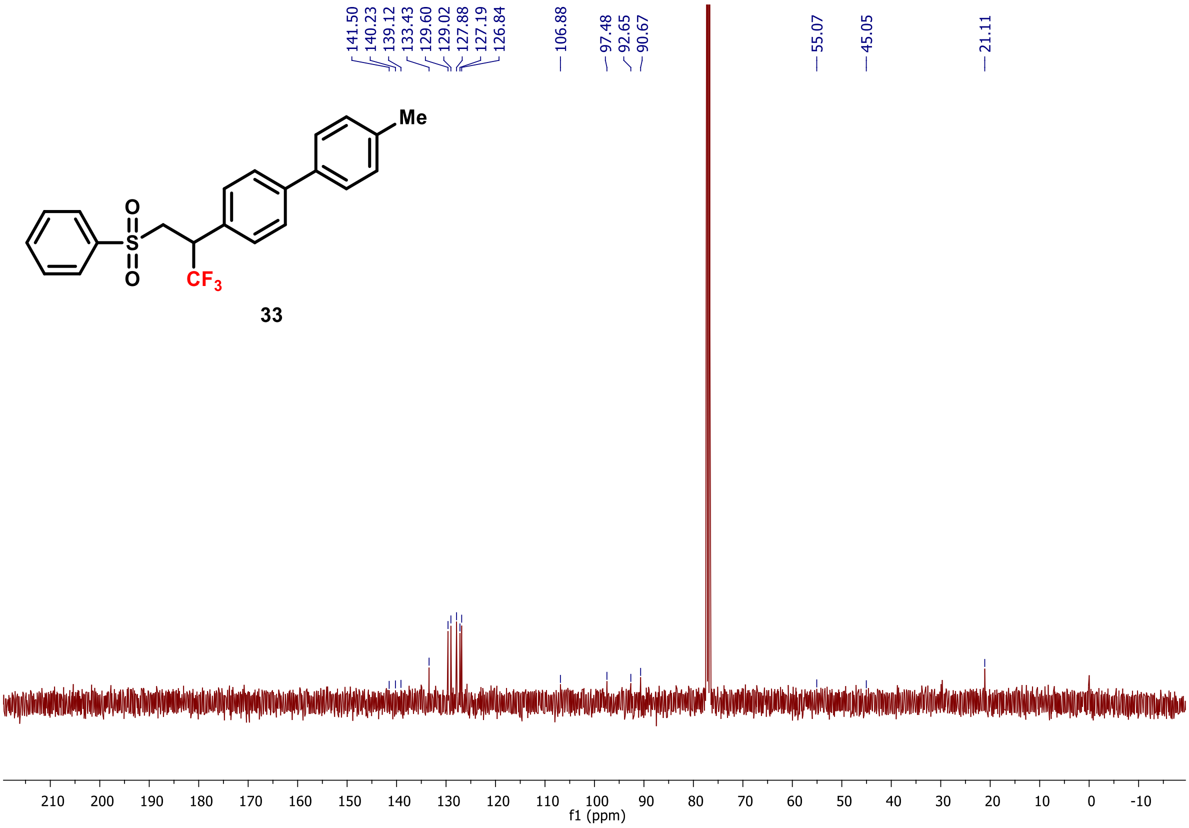

^{1}H NMR (400 MHz, $CDCl_3$) of **34**

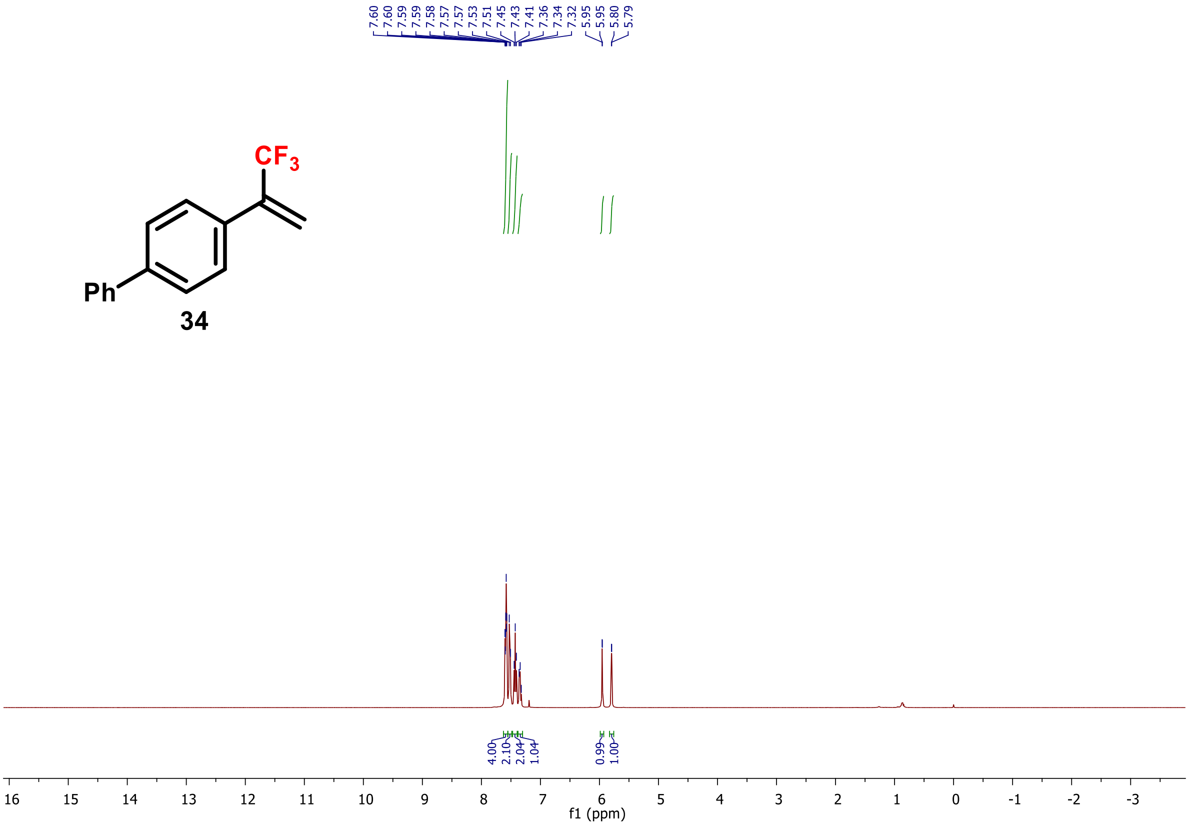

^{19}F NMR (376 MHz, $CDCl_3$) of **34**

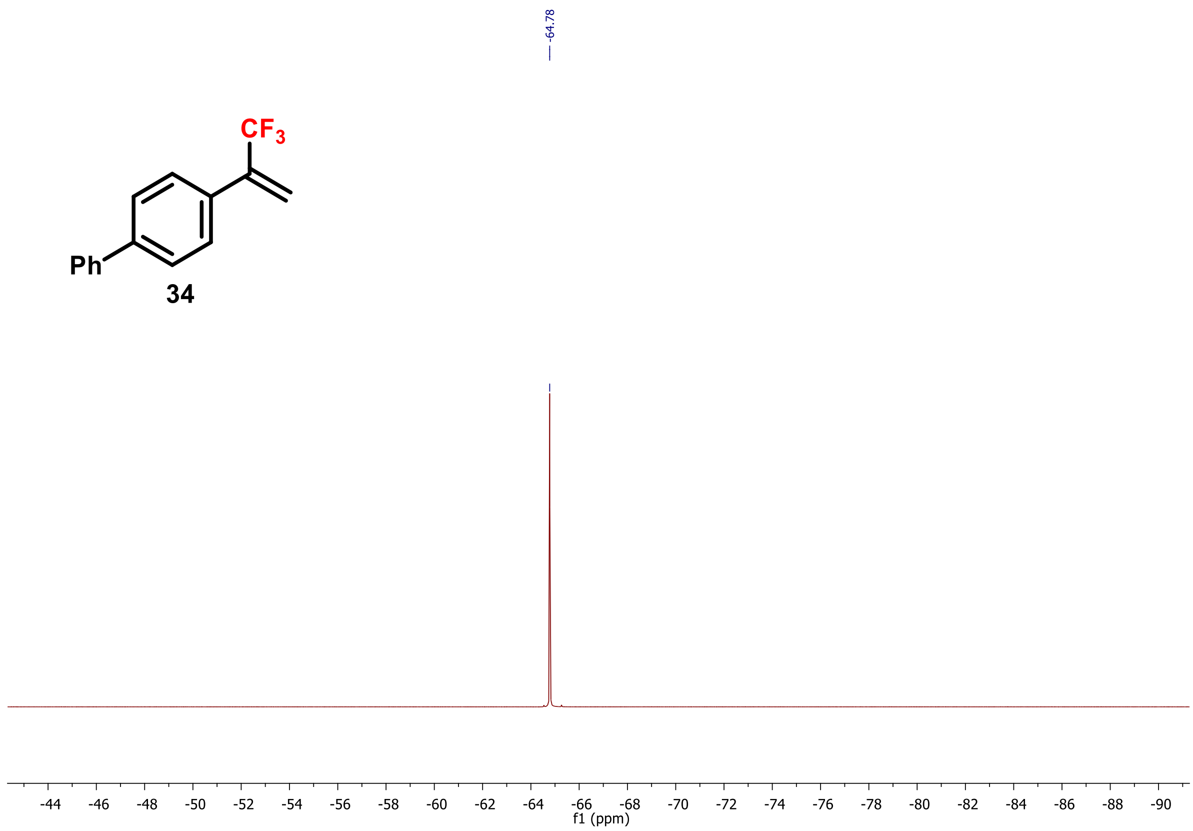

^{13}C NMR (101 MHz, $CDCl_3$) of **34**

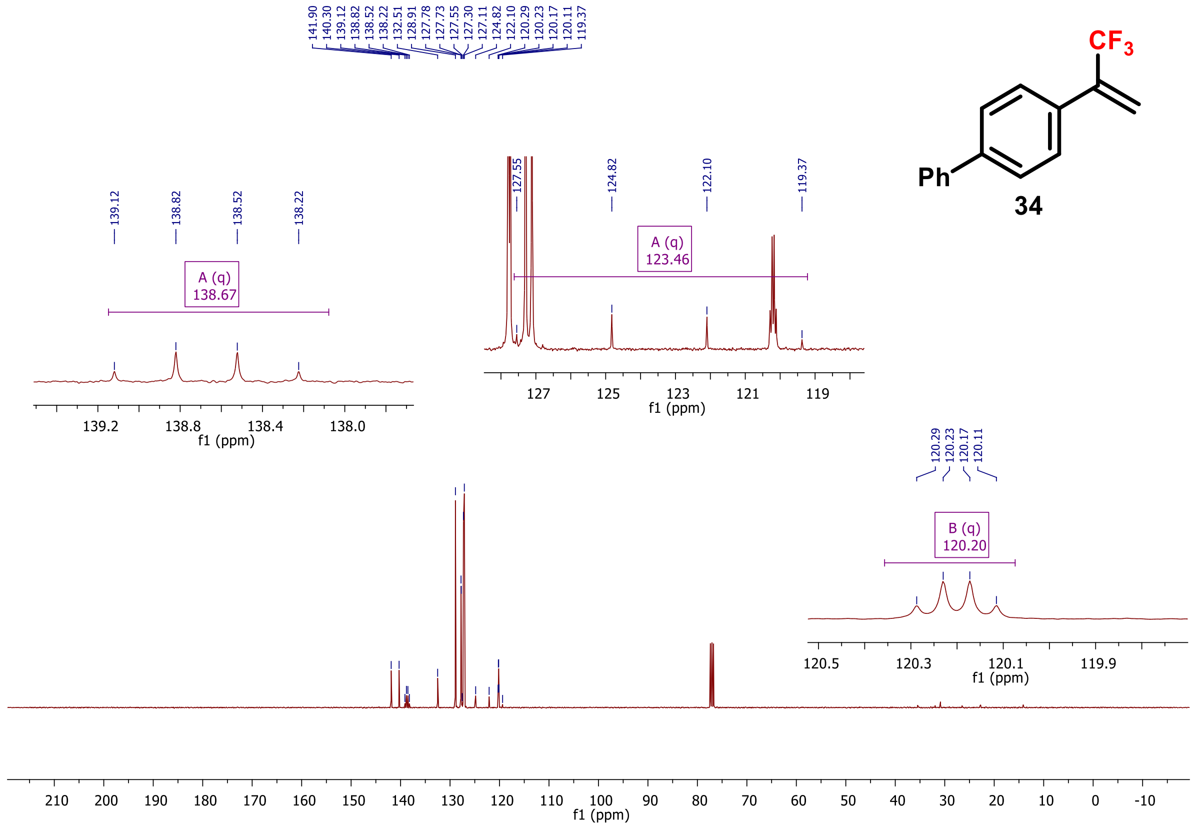

^{1}H NMR (400 MHz, $CDCl_3$) of **35**

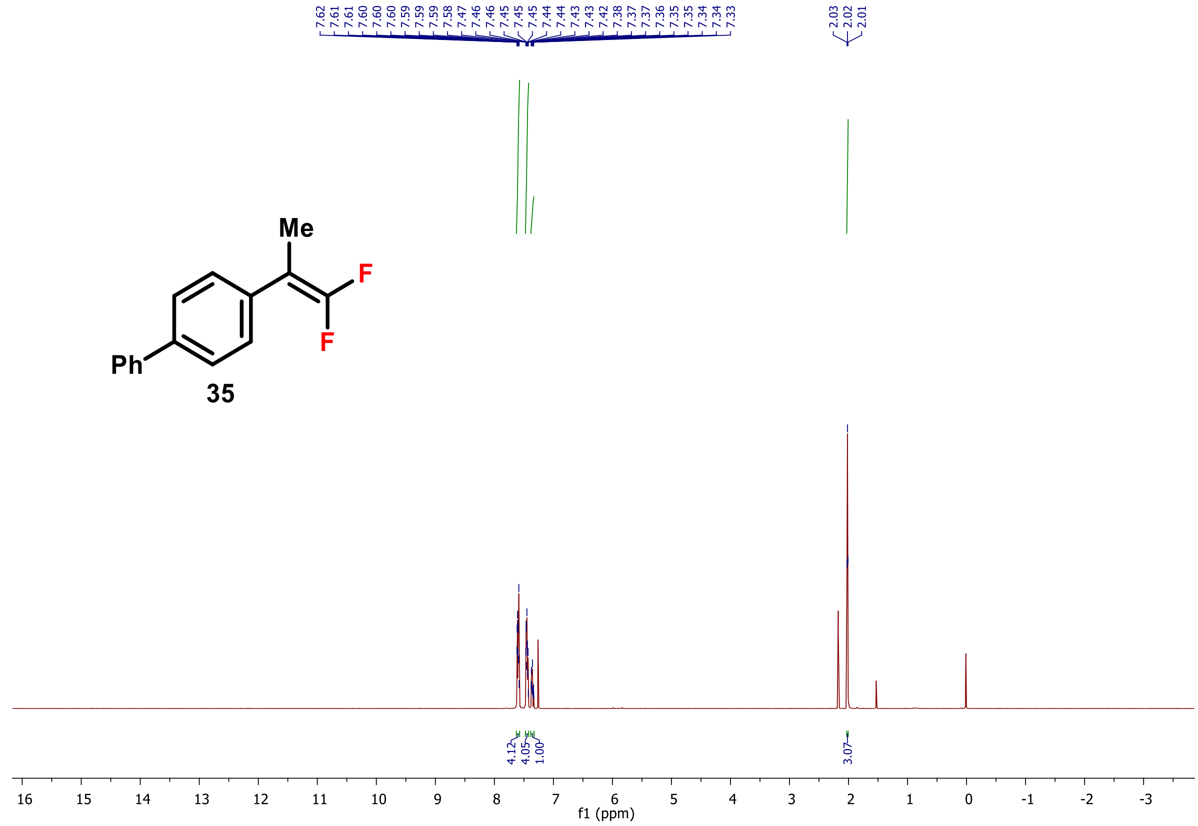

^{19}F NMR (376 MHz, $CDCl_3$) of **35**

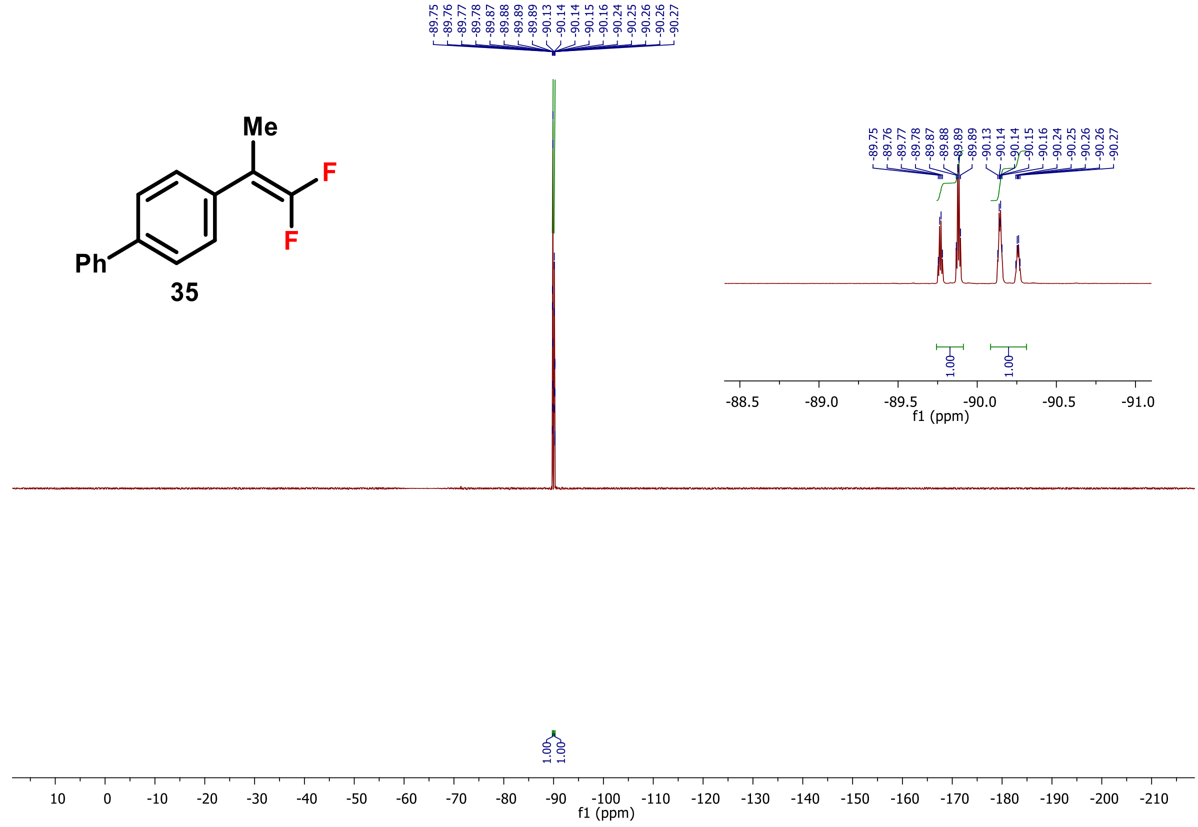

^{13}C NMR (101 MHz, $CDCl_3$) of **35**

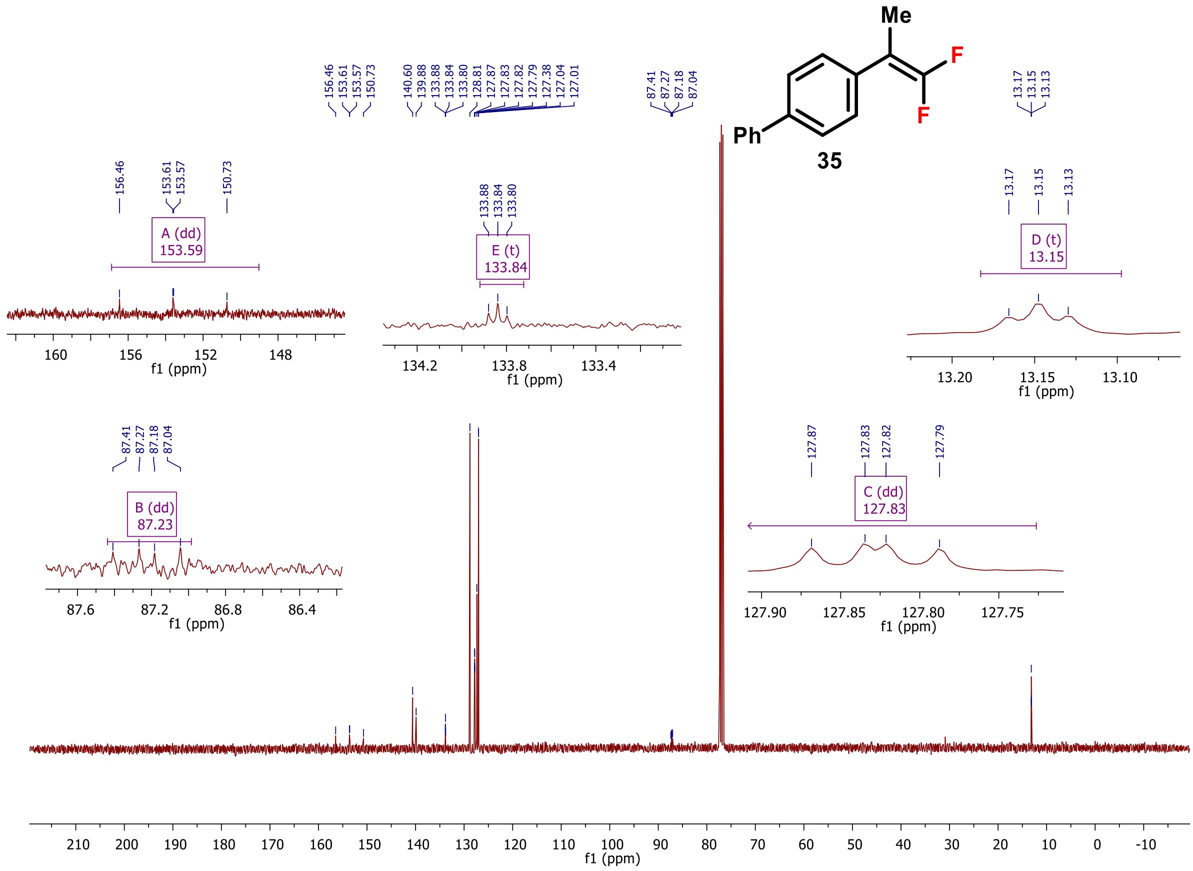

^{1}H NMR (400 MHz, $CDCl_3$) of **36**

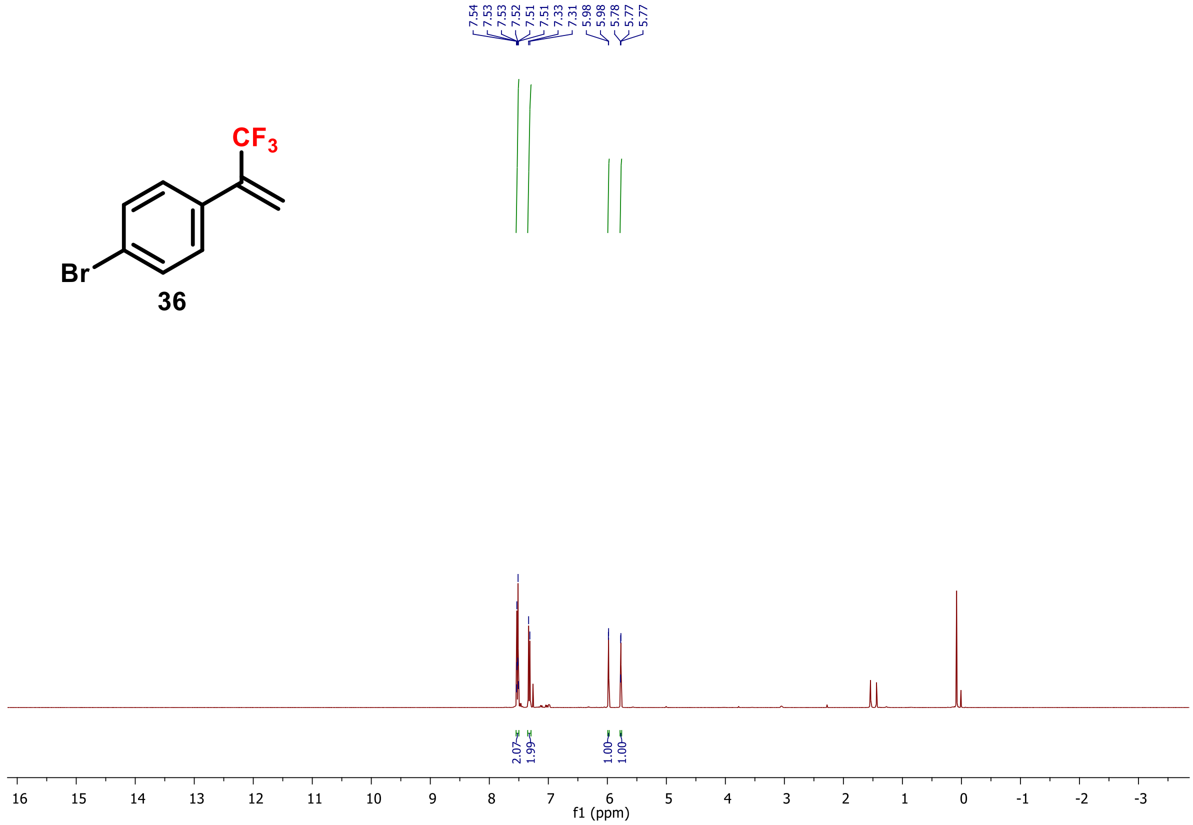

^{19}F NMR (376 MHz, $CDCl_3$) of **36**

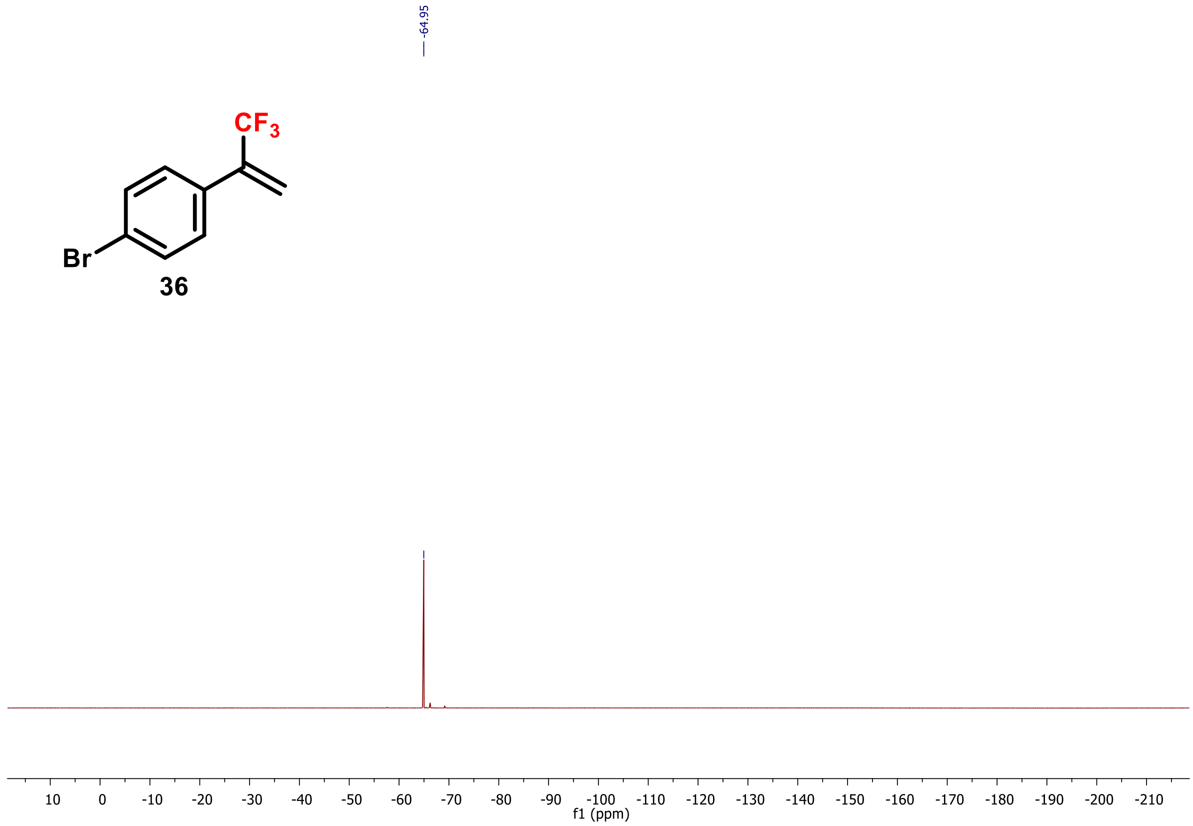

^{13}C NMR (101 MHz, $CDCl_3$) of **36**

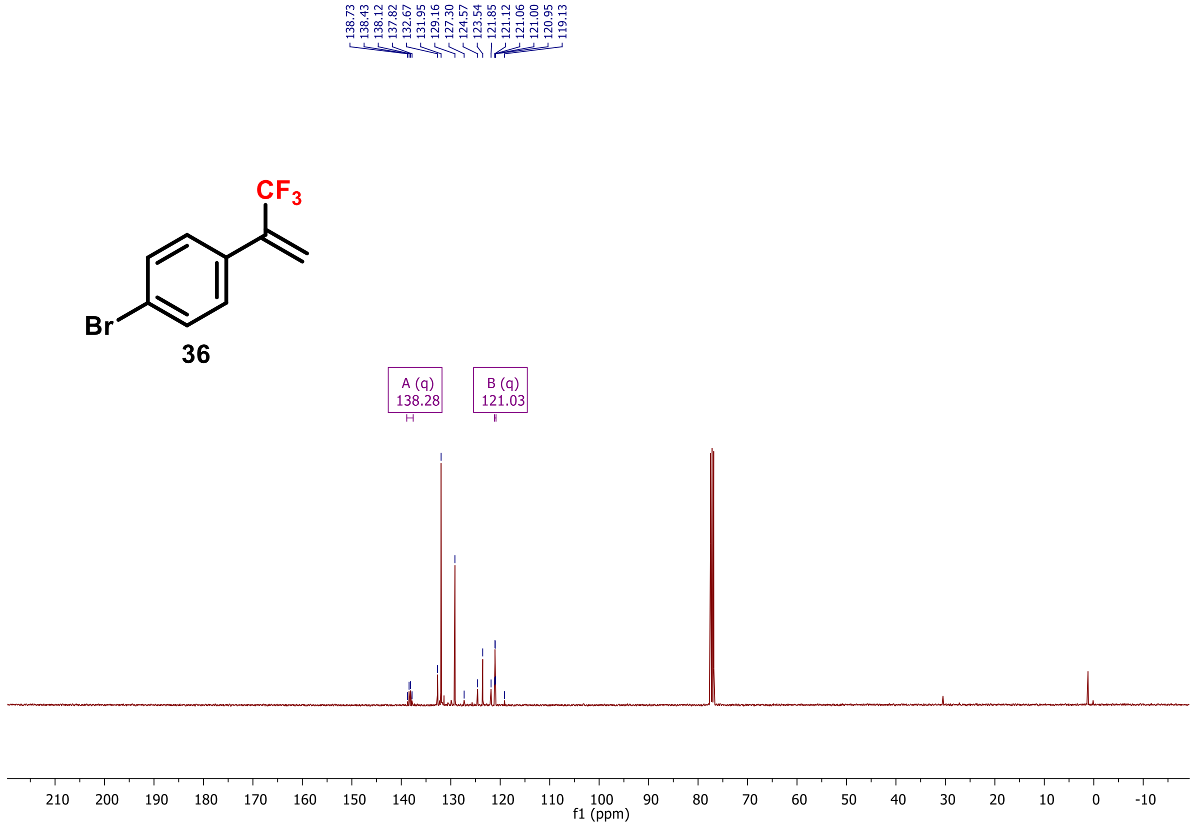

^{1}H NMR (400 MHz, $CDCl_3$) of **41**

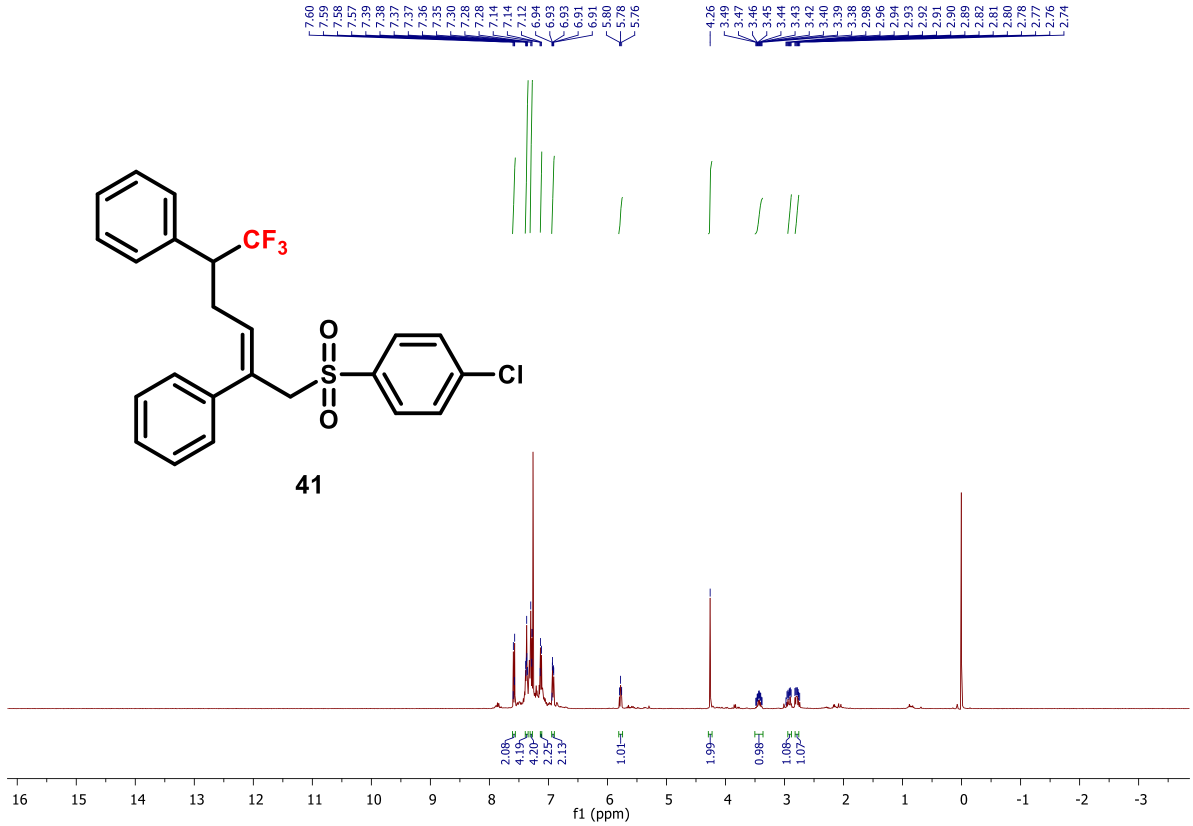

^{19}F NMR (376 MHz, $CDCl_3$) of **41**

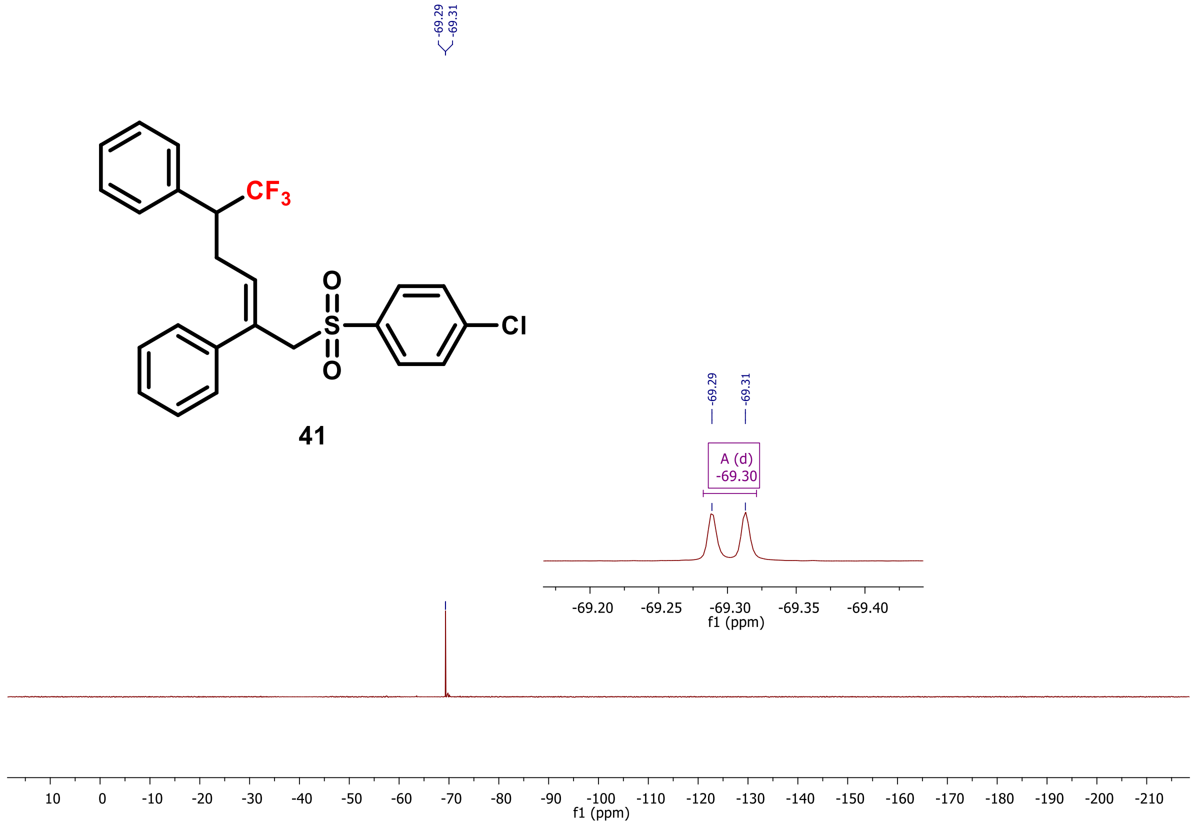

^{13}C NMR (101 MHz, $CDCl_3$) of **41**

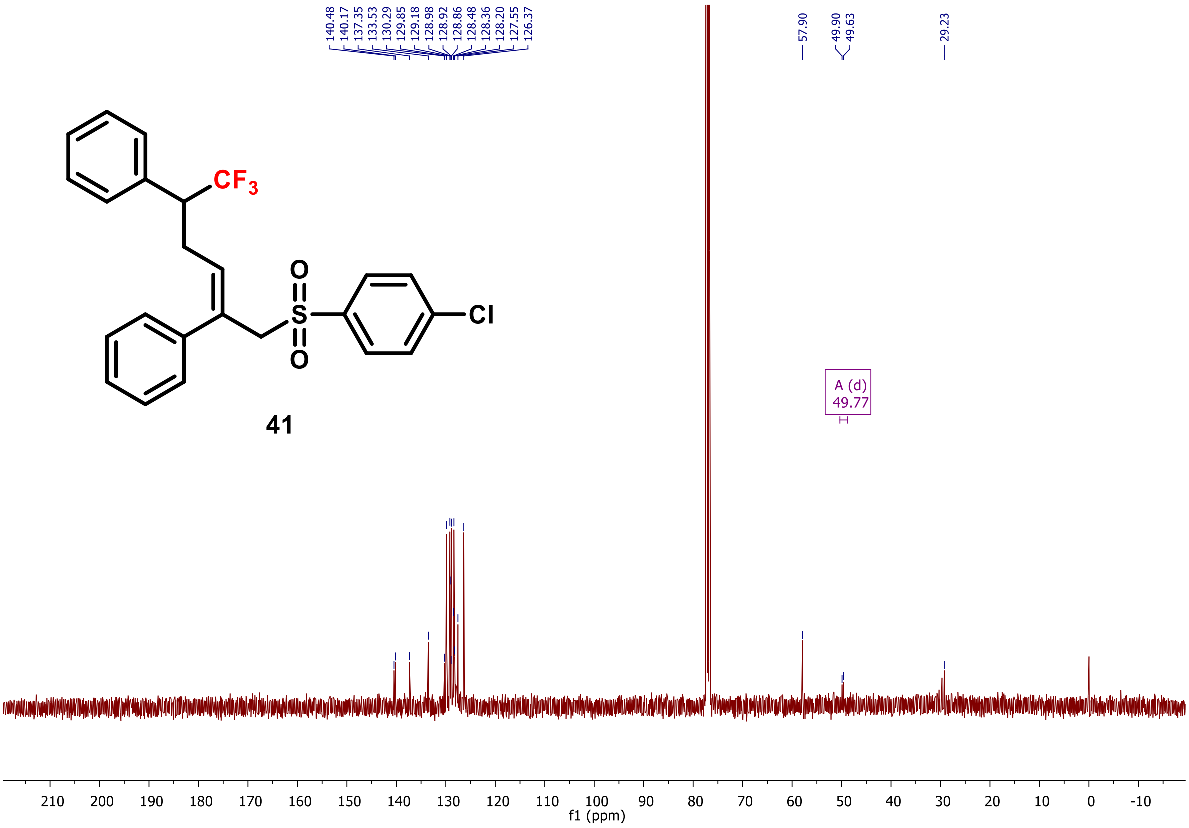
CF3
O
S
O
Cl
140.48
140.17
137.35
133.53
130.29
129.85
129.18
128.98
128.92
128.86
128.48
128.36
128.20
127.55
126.37
57.90
49.90
49.63
29.23
A (d)
49.77
210 200 190 180 170 160 150 140 130 120 110 100 90 80 70 60 50 40 30 20 10 0 -10
f1 (ppm)

41

Supplementary References

1.8 Reference

1. Ravetz, B. D., Tay, N. E. S., Joe, C. L., Sezen-Edmonds, M., Schmidt, M. A., Tan, Y., Janey, J. M., Eastgate, M. D., Rovis, T. Development of a Platform for Near-Infrared Photoredox Catalysis. *ACS Cent. Sci.* **6**, 2053–2059 (2020).
2. Romine, A. M., Nebra, N., Konovalov, A. I., Martin, E., Benet-Buchholz, J., Grushin, V. V. Easy Access to the Copper(III) Anion $[Cu(CF_3)_4]^-$. *Angew. Chem. Int. Ed.* **54**, 2745–2749 (2015).
3. Sarver, P. J., Bacauanu, V., Schultz, D. M., DiRocco, D. A., Lam, Y.-h., Sherer, E. C., MacMillan, D. W. C. The merger of decatungstate and copper catalysis to enable aliphatic $C(sp^3)$–H trifluoromethylation. *Nat. Chem.* **12**, 459–467 (2020).
4. Zhang, T., Schilling, W., Khan, S. U., Ching, H. Y. V., Lu, C., Chen, J., Jaworski, A., Barcaro, G., Monti, S., De Wael, K., Slabon, A., Das, S. Atomic-Level Understanding for the Enhanced Generation of Hydrogen Peroxide by the Introduction of an Aryl Amino Group in Polymeric Carbon Nitrides. *ACS Catal.* **11**, 14087–14101 (2021).
5. Kärkäs, M. D., Matsuura, B. S., Stephenson, C. R. J. Enchained by visible light-mediated photoredox catalysis. *Science*, **349**,1285–1286 (2015).
6. Jia, H., Häring, A. P., Berger, F., Zhang, L., Ritter, T. Trifluoromethyl Thianthrenium Triflate: A Readily Available Trifluoromethylating Reagent with Formal CF_3^+, $CF_3\bullet$, and CF_3^- Reactivity. *J. Am. Chem. Soc.* **143**, 7623–7628 (2021).
7. Ratushnyy, M., Kamenova, M., Gevorgyan, V. A mild light-induced cleavage of the S–O bond of aryl sulfonate esters enables efficient sulfonylation of vinylarenes. *Chem. Sci.* **9**, 7193–7197 (2018).
8. Tan, G., Das, M., Kleinmans, R., Katzenburg, F., Daniliuc, C., Glorius, F. Energy transfer-enabled unsymmetrical diamination using bifunctional nitrogen-radical precursors. *Nat. Catal.* **5**, 1120–1130 (2022).
